# *Mycobacterium leprae* infection, Hansen’s disease, and helminth infections: A cross-sectional study in southeastern Brazil

**DOI:** 10.1371/journal.pntd.0013285

**Published:** 2026-10-01

**Authors:** Audra Bass, Heloine Leite, Lorena B. P. Oliveira, Emma Nedell, Pedro Marçal, Julie A. Clennon, Jeffrey M. Collins, Thomas R. Ziegler, Erica Magueta Silva, Maisa P. Vieira, Marcos D. S. Pinheiro, Alexandre C. Branco, José A. Ferreira, Lance Waller, Lucia A. O. Fraga, Jessica K. Fairley

**Affiliations:** 1 Department of Medicine, Division of Infectious Diseases, Emory University School of Medicine, Atlanta, Georgia, United States of America; 2 Programa Multicêntrico de Bioquímica e Biologia Molecular/PMBqBM, Universidade Federal de Juiz De Fora-Campus Governador Valadares, Governador Valadares, Minas Gerais, Brazil; 3 Department of Health Sciences, Universidade Vale do Rio Doce (UNIVALE), Governador Valadares, Minas Gerais, Brazil; 4 Hubert Department of Global Health, Rollins School of Public Health, Emory University, Atlanta, Georgia, United States of America; 5 Coulter Department of Biomedical Engineering, Georgia Institute of Technology, Atlanta, Georgia, United States of America; 6 Department of Environmental Sciences, Emory University, Atlanta, Georgia, United States of America; 7 Department of Medicine, Division of Endocrinology, Metabolism and Lipids, Emory University School of Medicine, Atlanta, Georgia, United States of America; 8 Centro de Referência em Doenças Endêmicas e Programas Especiais, Governador Valadares, Minas Gerais, Brazil; 9 Unifenas Universidade, Belo Horizonte, Minas Gerais, Brazil; 10 Department of Biostatistics and Bioinformatics, Rollins School of Public health, Emory University, Atlanta, Georgia, United States of America; 11 Department of Epidemiology, Rollins School of Public Health, Emory University, Atlanta, Georgia, United States of America; Federal University of Ceará, Fortaleza, Brazil, BRAZIL

## Abstract

**Background:**

Neglected tropical diseases are often co-endemic in affected areas but how they interact is less clear, especially in the case of Hansen’s Disease (HD), commonly referred to as leprosy. Prior studies have demonstrated associations between helminths and HD, but our understanding of the epidemiology of these infections is limited. Our goal was to assess the prevalence and potential associations of *Mycobacterium leprae* infection and parasitic infections in a sample of community members in a highly endemic area for HD in Minas Gerais, Brazil.

**Methods:**

Adults and children ages 3 years and older were enrolled from communities in Governador Valadares, Minas Gerais, Brazil, and nearby municipalities. Questionnaires on demographics and infection history were administered. Serological reactivity against *M. leprae*, *Schistosoma mansoni,* and *Strongyloides stercoralis* was assessed by multiplexed bead assay (MBA). Descriptive, univariate, and multivariate statistics were performed to describe the outcomes and potential associations.

**Results:**

Of 1,311 participants, 72 (5.5%) reported a prior history of HD, 94 (7.2%) tested positive for anti-LID-1, 836 (63.8%) reported having one or more parasitic diseases in the past, 153 (11.7%) tested positive for antibodies to schistosoma egg antigen (SEA), and 69 (5.3%) for antibodies to the *Strongyloides stercoralis* antigen, NIE. Rural residence and anti-LID-1 showed a positive association (aOR 1.79, CI: 1.07- 3.38) as did rural residence and history of HD (aOR, 1.97, CI: 1.14 – 3.38). While not statistically significant, there was a positive association between anti-LID-1 and NIE antibodies (aOR 1.57, CI: 0.69-3.57), and a negative association between anti-LID-1 and SEA antibodies (aOR 0.79, CI: 0.38 - 1.61).

**Conclusion:**

We simultaneously measured seroreactivity among various pathogens. While we did not find that a history of HD and anti-LID-1 positivity were associated with seropositivity to NIE or SEA, our study found a high burden of several neglected tropical diseases (NTDs). There was also a strong association with rural residence and LID-1 positivity warranting investigation into the prevalence and epidemiology of *M. leprae* infection, as well as potential spatial and environmental risk factors.

## Author summary

Hansen’s disease, leprosy, continues to affect populations globally with Brazil reporting the second highest number of cases after India. Helminth infections are often co-endemic with HD and have been found to be associated with leprosy in prior research. Therefore, in order to better understand the epidemiology of *Mycobacterium leprae* infection and helminths in a hyperendemic area Brazil, we sought to describe the overlap of these neglected tropical diseases. We measured antibody reactivity to *M. leprae*, *Schistosoma mansoni*, and *Strongyloides* *stercoralis* in a sample of 1311 adults and children in eastern Minas Gerais, Brazil using a multiplexed beaded assay. In addition, we collected data on prior history of HD and parasitic infections. We found that 7% of the participants tested positive for IgGagainst the leprosy bacteria (anti-LID-1) and 6% had a prior history of HD. *Schistosoma mansoni* antibodies and *Strongyloides stercoralis* antibodies were present in 12% and 5% respectively and 2/3 of individuals self-reported a parasitic infection in the past. We did not find statistically significant associations between the different infections; however, the proportion of individuals who were either seropositive or who self-reported prior infections demonstrated a large burden of HD and other neglected tropical infections in these communities. In addition, rural residence was associated with HD, highlighting future area of study to home in on potential associated risk factors.

## Introduction

Hansen’s disease (HD), also commonly known as leprosy, is an ancient disease of humans [1] and is caused by the bacteria *Mycobacterium leprae* and less commonly *Mycobacterium lepromatosis* [2]. Each year there are approximately 200,000 new cases worldwide, with Brazil reporting the 2^nd^ highest number after India, 22,773 in 2023, despite significant improvements after multi-drug treatment was introduced in the 1980s [2,3]. The exact mode of transmission is not fully understood; however, the most widely accepted theory is person-to-person through nasal secretions with zoonotic and environmental reservoirs also thought to play a role [2,4–7]. We do not fully understand why some areas in Brazil continue to have high transmission when other areas do not. This may be due to specific unique risk factors present in those areas. Risk factors that may aggravate or facilitate transmission and / or the development of disease include poverty, undernutrition, water and sanitation practices, armadillo contact, and other environmental exposures [4,8–11]. Concomitant infections, like helminths, could also predispose someone to either acquire infection or develop disease once infected due to their immunomodulatory properties, and have been a subject of increased investigation [12,13].

Helminth infections affect up to a quarter of the world’s population, in primarily impoverished communities, sometimes leading to people being infected by multiple pathogens over the course of their life [14,15] Schistosomiasis and intestinal helminths elicit a strong systemic T-helper cell (Th)-2 response which up-regulates regulatory T-cell activity, leading to a weakened pro-inflammatory Th1 response [12,16]. Since host control of *M. leprae* relies on a robust Th1 response, this may be a mechanism of risk for the development of HD. One study previously demonstrated that prevalence of multibacillary HD was statistically significantly higher among patients with soil-transmitted helminth infections than those with no helminth infections [12]. In this situation, Th2 cytokines were more prominent in the co-infected individuals. Studies have also demonstrated an association between HD and helminths, in Brazil, Nepal, and Indonesia [12,13,17,18]. These associations led us to investigate potential overlap between *M. leprae* and two helminths using a multiplexed beaded assay (MBA) that can be used to simultaneously measure seroreactivity to several pathogens at once [19,20].

To better understand the epidemiology of HD and select parasitic infections in eastern Minas Gerais, we measured seroreactivity to *M. leprae*, *Schistosoma mansoni*, and *Strongyloides stercoralis*. *M. leprae* serological assays, including anti-LID-1 (IDRI Diagnostic protein) and anti-PGL-I (phenolic glycolipid-I) can predict which individuals are at higher risk for developing HD, and presumably represent latent infection or exposure to infection (i.e., resolved infection) in those without symptoms [21,22]. We used serology as a marker of infection, not necessarily as an indicator of HD, so we could thus capture those that have not yet or may never develop disease. While the sensitivity and specificity of these tools for latent infection are not known, they can still give us an epidemiological snapshot of HD and allow us to describe associations between seroreactivity and other factors, such as helminth infection, something that has not been fully investigated in the literature. We also wanted to compare participants with a history of HD to those without such history to determine how exposures might differ among these groups. In other words, we aim to describe cross-sectional associations around prevalence estimations. The goal, ultimately, was to get a better understanding of the factors that may be prevalent in those either with current or past *M. leprae* infection to inform larger-scale research and public health interventions. While we understand that we can not determine causality, we hypothesized that those seropositive *for M. leprae* or who had HD in the past would be more likely to be seropositive for a helminth infection, given what we know about potential immune dysregulation of helminth infections versus common environmental reservoirs.

## Methods

### Ethics statement

This study was approved by the institutional review boards of Emory University and Universidade Federal de Juiz de Fora. All participants provided written informed consent with parents providing written consent for their children. In addition, Children ages 7–17 years old provided assent as per IRB guidelines. CDC staff were determined to be non-engaged in human subjects research as they did not interact with study participants or have access to identifying information.

### Study design and population

The study took place in four municipalities of eastern Minas Gerais, Brazil, in and near the city of Governador Valadares (Governador Valadares [GV], Inhapim, Teófilo Otoni, and Mantena) (Fig 1). The neighborhoods in each municipality were chosen due to relatively high rates of reported HD, with some areas considered hyperendemic (incidence rate of >40 / 100,000 annually) [2,24]. We partnered with local family health centers of each neighborhood to assist with recruitment of community members. For GV, Dr. Fraga chose the neighborhoods based on high incidence of HD and prior work in these neighborhoods. The health centers in the other municipalities were chosen by the local secretary of health after consulting with them. In GV, the neighborhoods were divided by block, and houses on each street were selected randomly, with approximately 4–5 houses per street. To approach the families, the Community Health Agents (ACSs), with our study team, rang the doorbells to check if anyone was home. If someone answered, the team briefly explained the project and gave the invitation to the resident. To broaden the coverage area, the team avoided approaching houses that were very close to each other, trying not to approach the 2 or 3 houses that were immediately adjacent to the house where the invitation was delivered. Approaching a neighboring house was only done when no resident of the initially selected house answered. Invitations were given for one individual per household. The household was allowed to choose the person to be included. For the other municipalities, the local community health centers invited families in a similar manner. Eligible participants included adults and children ages 3 years and older currently living in the communities. To have a representative sample for each neighborhood, households were chosen randomly across the neighborhoods with the assistance of the community health worker, ensuring geographic separation (i.e., no contiguous households and on average 1–3 households per block). Invitations were given for one individual per household to attend the recruitment and enrollment day later that week. While the goal was only one individual per household, rarely, more than one individual per household was enrolled.

**Fig 1:**
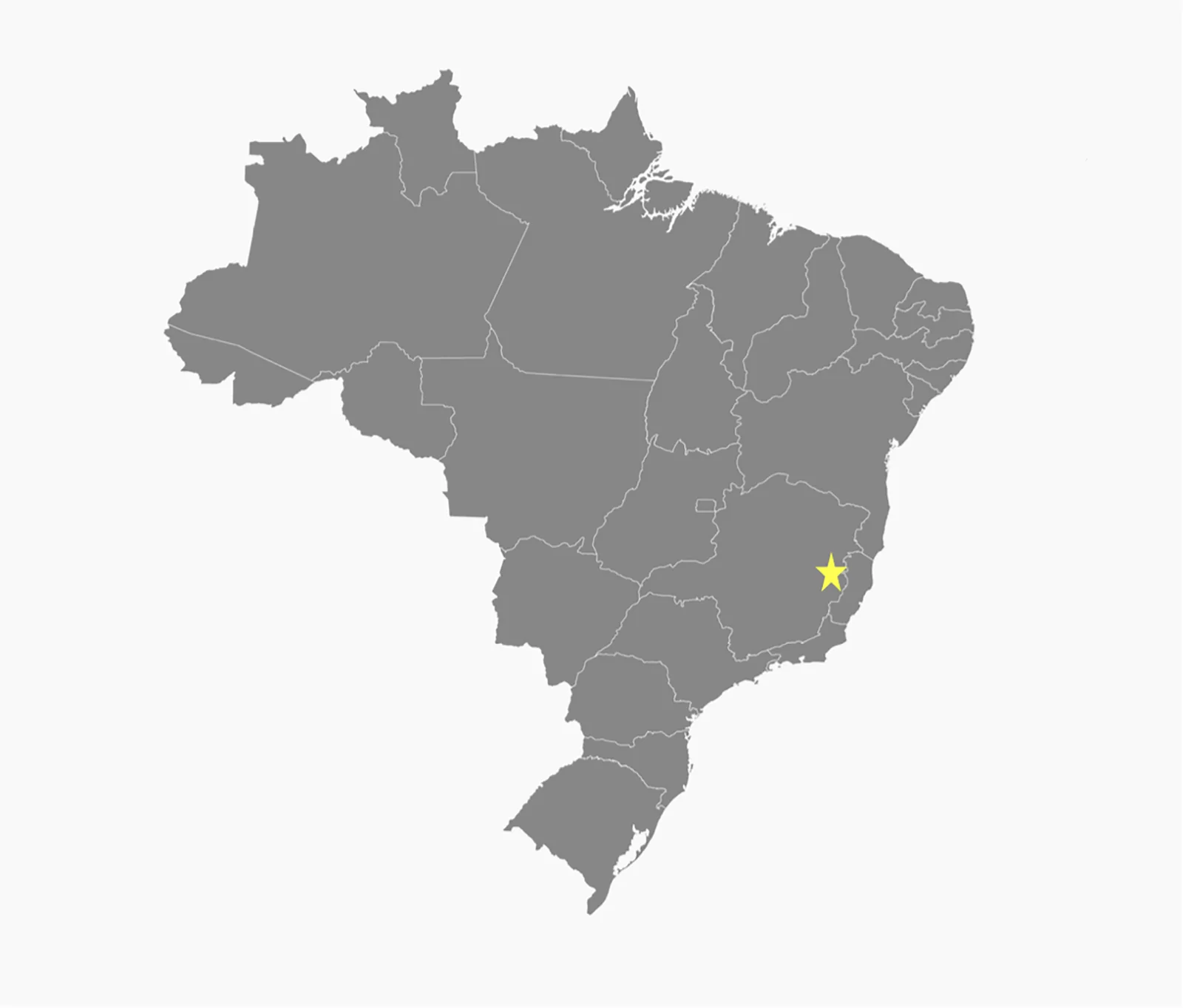
Map of Minas Gerais state, Brazil, with star marking general area of the 4 municipalities included in the study. Adapted from FreeVectorMaps.com [23].

### Data collection

Enrollment occurred from September 2021 to December 2022 with study activities conducted at local family health centers. Data collection sessions took place on a Saturday and worked around the farming/harvesting schedules to ensure community members’ availability. A questionnaire was administered and included questions on demographics (i.e., sex, age, race, residence, monthly salary, household factors) and self-reported history of the following infections: roundworm, schistosomiasis, hookworm, tapeworm, pinworm, giardia, amoebiasis, and HD. In addition, individuals were asked if they had a scar from the BCG (bacille Calmette-Guérin) vaccine, which is given to household contacts (HHC) of individuals with HD because it has been shown to reduce the risk of the HD in HHC [25] in addition to being mandatory for childhood vaccination schedule in Brazil. However, coverage of BCG has declined in recent years [26].

A fingerstick blood draw was taken for a dried blood spot which was transported to the Centers for Disease Control and Prevention in Atlanta, GA. Serum was eluted from the dried blood spots [27–32] and tested for antibodies to LID-1 from *M. leprae*, a pan-*Schistosoma* soluble egg antigen (SEA), and NIE, a recombinant antigen from *Strongyloides* stercoralis [27]. Using a multiplex bead serological assay (MBA) SEA and NIE were coupled to MagPlex beads (Luminex Corp, Austin, TX) as previously described [19,20,28]. LID-1 was coupled with the same procedure using 15 µg of antigen per 12.5 × 10^6^ beads in PBS pH7.2. Seropositivity cutoffs for LID-1 and SEA were determined using the mean plus three standard deviations of readings from 85 individuals from North America. The cutoff for NIE was generated using receiver operator characteristic (ROC) curve analysis of 94 sera from North American individuals and 53 from individuals whose stool was positive for *S. stercoralis* at the time of sera collection [27]. Anti-SEA antibodies are generally thought to represent recent or ongoing infection as studies show reduction in all IgG subtypes against SEA after treatment with “high decay” of IgG in early studies [29–31]. However, this is not universal, and positive anti-SEA still has to be interpreted with caution. In terms of strongyloidiasis, serological responses to NIE are highly sensitive and specific for infection. While they decrease after treatment and following antibody levels is widely used to assess cure [32], anti-NIE IgG may not completely disappear and therefore can represent recent or active infection.

All positive participants received their results and those positive for anti-LID-1 IgG were evaluated by physicians at CREDENPS (Centro de Referência de Doenças Especiais e Programas) for signs and symptoms of HD. These individuals, along with the same number of anti-LID-1 negative controls, were then enrolled in a longitudinal study, which is outside the scope of this analysis.

### Statistical analysis


$$\begin{array}{c} {\text{n}}_{\text{1}}\;=\;\frac{{\left({\text{Z}}_{\alpha /\text{2}}+{\text{Z}}_{\text{1}-\beta }\right)}^{\text{2}}\overset{―}{\text{p}}\overset{―}{\text{q}}(\text{r}+\text{1})}{{\text{r}({\text{p}}_{\text{1}}-{\text{p}}_{\text{2}})}^{\text{2}}} \end{array}$$

All data were maintained on the data management platform RedCAP [33] and analyses were carried out in R4.3.0 using packages: ‘readxl’, ‘janitor’, ‘tidyverse’, ‘AICcmodavg’, ‘gmodels’, and ‘stargazer’ [34]. Descriptive statistics were performed using frequency and measures of central tendency as appropriate. Univariate analyses with t-test for continuous exposure variable(s) or Chi-square test for categorical variable(s) were conducted to determine associations with anti-LID-1 seropositivity or history of HD. For the purpose of further analyses, all categorical variables in the questionnaire were transformed to binary indicators, as follows: Sex as ‘female’ or ‘male’ (no one identified as non-binary), Race as ‘non-white’ (black, mixed, indigenous, Asian descent, other) or ‘white’, Residency as ‘rural’ or ‘urban’, Salary as ‘<1 minimum’ (1 x the minimum wage is a standard measure of monthly salary) or ‘>1 x minimum’. Age was used as both a continuous variable and as a binary variable to create categories of participants less than the age of 15 (regarded as a minor) and all others who were 15 years of age or older (regarded as an adult). Sample size calculations done apriori used an estimated odds ratio of 3 for the association of anti-LID-1 and anti-SEA (based on prior reports of associations between HD and helminth infection of 4 or more) [12,13], and an estimated 10% seropositivity for *schistosoma* infection [35]. The sample size required to attain a power of 0.8 and significance of 0.05 was 45 individuals positive for anti-LID-1 and 446 negative. The anti-LID-1 analyses were also stratified by age (<15 vs 15 or greater) to determine if any associations differed based on age. Sample size calculation was done using OpenEpi (v3.01) (https://www.openepi.com/SampleSize/SSCohort.htm), using the Kelsey method, which uses the above formula where n_1_ = number exposed, n_2_ = number of unexposed, Z_α/2_ = standard normal deviate for two-tailed test based on alpha levels, Z_β_ = standard normal deviate for two-tailed test based on beta level, r = ratio of unexposed to exposed, p_1_ = proportion of exposed with disease and q_1_ = 1-p_1;_ p_2_ = proportion of unexposed with disease and q_2_ = 1-p_2_.

Multivariate logistic regression models were built to explore associations between anti-LID-1 positivity and helminth serology (anti-SEA positivity and anti-NIE positivity). We also built separate models with history of HD as the outcome instead of anti-LID-1 positivity. We then incorporated the following other measures of infectious parasites in three separate models: 1) individual infectious parasites that were self-reported from the questionnaire; 2) all individual infectious parasites into categories as follows: self-reported schistosomiasis, self-reported non-schistosomiasis helminths (comprising roundworm, hookworm, tapeworm, pinworm), and self-reported protozoa (comprising giardia, amoeba, and other); and 3) model that included only ‘yes’ or ‘no’ to whether someone had any history of parasitic disease. Each of these models incorporated two different outcomes of interest: 1) a participant resulting ‘positive’ for anti-LID-1 antibodies and 2) a participant having had a history of HD. Model diagnostics with backwards selection and Akaike information criterion (AIC) were used to determine which variables were used for the final models. The crude forms of the logistic regression models included the following variables: ‘age’, ‘sex’, ‘race’, ‘residence’, ‘monthly salary’, ‘BCG scar’, ‘past parasitic infection’, self-reported parasites (‘Roundworm’, ‘Schistosomiasis’, ‘Hookworm’, ‘Tapeworm’, ‘Pinworm’, ‘Giardia’, ‘Amoeba’, and ‘Other’), ‘Strongyloides antibody (Anti-NIE)’, and ‘Schistosomiasis antibody (Anti-SEA)’. We reached our final models through backwards selection by dropping 1) ‘age’, ‘sex’, ‘monthly salary’, ‘past parasitic infection’, and ‘bcg scar’ for the Anti-LID-1 model and 2) ‘race’, ‘monthly salary’, ‘past parasitic infection’, and ‘bcg scar’ for the Reported History of HD model.

## Results

### Demographics

A total of 1,315 participants were enrolled with 1,311 having sufficient data to include in the study. Those four participants were removed for either having no results for the MBA assay or having not completed the surveys. The median age was 52 years (range 4–92) with 64.1% of participants female (840/1311), 54.2% (710/1311) identifying as mixed race and the majority, (83.1% (1,089/1311)) living in an urban setting. Ninety-four (out of 1311) individuals (7.2%) tested positive for anti-LID-1 antibody. Over a quarter (34.3% (450/1311)) made less than minimum wage for Brazil (Table 1). **Table 1** shows demographic variables stratified by anti-LID-1 status. Only residence differed across serological status with 25/92 (26.6%) reporting rural residence compared to their LID-1 negative counterparts (195/1215, 16%), p < 0.01.

**Table 1:** Demographic data comparing those who tested positive for anti-LID-1 antibody and those who tested negative.

Variable	Positive Anti-LID-1-antibody (n = 94)	Negative Anti-LID-1-antibody (n = 1217)	Total (n = 1311)	P-value
Age (mean, SD)	50.1 (20.5)	48.6 (20)	48.8 (20)	0.69
Age Categorical (n, %)				0.69
<15 years of age	7 (7.4%)	105 (8.6%)	112 (8.5%)	
Sex (n, %)				0.54
Female	63 (67%)	777 (63.8%)	840 (64.1%)	
Male	31 (33%)	440 (36.2%)	471 (35.9%)	
Race (n, %)				0.67
Black	20 (21.3%)	277 (22.8%)	297 (22.7%)	
White	16 (17%)	242 (19.9%)	258 (19.7%)	
Mixed	54 (57.4%)	656 (53.9%)	710 (54.2%)	
Asian	0 (0%)	NA	0 (0%)	
Indigenous	0 (0%)	6 (4.9%)	6 (0.5%)	
Other	1 (1.1%)	4 (3.3%)	5 (0.4%)	
*Missing = 35*	*3 (0.32%)*	*32 (2.6%)*	*35 (2.7%)*	
*Residence (n, %)*				***<0.01***
Urban	69 (73.4%)	1020 (83.8%)	1089 (83.1%)	
Rural	25 (26.6%)	195 (16%)	220 (16.8%)	
*Missing = 2*	*0 (0%)*	*2 (0.2%)*	*2 (0.1%)*	
Monthly Salary (n, %)				0.48
< 1 x Minimum Salary	32 (34%)	418 (34.3%)	450 (34.3%)	
1-3 x Minimum Salary	59 (62.7%)	687 (56.5%)	746 (56.9%)	
3-5 x Minimum Salary	1 (1.1%)	49 (4%)	50 (3.8%)	
> 5 x Minimum Salary	1 (1.1%)	11 (0.9%)	12 (0.9%)	
*Missing = 53*	*1 (1.1%)*	*52 (4.3%)*	*53 (4%)*	

Comparisons done using t-test for continuous variables and chi-square or univariate logistic regression for categorical values. Bolded p-values are considered significant with a p-value of 0.05 or less. ‘Missing’ refers to participants responding ‘don’t know’, ‘refused to answer’, or if this question was left blank by their interviewer.

### Infection history / status

Table 2 demonstrates the infectious diseases data by anti-LID-1 status. Of the 1017 we had data on for HD history, seventy-two (72/1017, 7.1%), had a history of HD. This question was initially left off the survey, but all anti-LID-1 positive individuals were asked, retrospectively if needed. Of those reporting a history of HD, 15 (20.8%) were anti-LID-1 positive. For *S. mansoni* reactivity, 153 / 1311 individuals (11.7%) tested positive, of which 9 individuals (9.6%) were anti-LID-1 positive. Likewise, a total of 69 /1311 (5.3%) tested positive for *S. stercoralis* IgG, with 7 (7.4%) of those individuals also reacting seropositive for anti-LID-1. Seropositivity for both helminths did not differ significantly across anti-LID-1 positivity. The point estimates remained essentially the same when the anti-LID-1 analyses were stratified by age < 15 and 15 and older.

**Table 2:** Infectious disease data comparing those who tested positive for anti-LID-1 antibody and those who tested negative.

Variable	Positive Anti-LID-1-antibody (n = 94)	Negative Anti-LID-1-antibody (n = 1217)	Total (n = 1311)	P-value
BCG scar (n, %)	64 (68.1%)	831 (68.3%)	895 (68.3%)	0.49
*Missing = 92*	*3 (3.2%)*	*89 (7.3%)*	*92 (7%)*	
Reported history of HD (n, %)	15 (16%)	57 (6.2%)	72 (5.5%)	**<0.01**
*Missing = 294*	*0 (0%)*	*294 (100%)*	*294 (22.4%)*	
*Schistosoma mansoni* (anti-SEA) ab positive (n, %)	9 (9.6%)	144 (11.8%)	153 (11.7%)	0.51
*Strongyloides stercoralis* ab pos (anti-NIE) (n, %)	7 (7.4%)	62 (5.1%)	69 (5.3%)	0.33
Self-reported History of Parasitic Infection (n, %)				0.89
Yes	65 (69.2%)	771 (63.3%)	836 (63.8%)	
No	27 (28.7%)	331 (27.2%)	358 (27.3%)	
*Missing = 117*	*2 (2.1%)*	*115 (9.5%)*	*117 (8.9%)*	
Which self-reported Parasitic infection (n, %)*				
Roundworm	36 (38.3)	410 (33.7)	446 (34.0)	0.71
Schistosomiasis	25 (26.6)	273 (22.4)	298 (22.7)	0.61
Hookworm	3 (3.2)	10 (0.8)	13 (1)	***0.03***
Tapeworm	9 (9.6)	62 (5.1)	71 (5.4)	0.10
Pinworm	6 (6.4)	105 (8.6)	111 (8.5)	0.34
Giardia	4 (4.3)	50 (4.1)	54 (4.1)	0.93
Amoeba	8 (8.5)	114 (9.4)	122 (9.3)	0.62
Other	0 (0%)	5 (0.4%)	5 (0.4%)	0.52

*Self-reported refers to participants who answered ‘Yes’ or ‘No’ to the following question – “Have you ever been diagnosed with a parasitic infection?”. If a participant selected ‘Yes’ they were then prompted with a list of possible parasites and to indicate which of the following they had been infected by and/or provide an option for ‘Other’ if a parasite was not listed. ‘Missing’ refers to participants responding ‘don’t know’, ‘refused to answer’, or if this question was not answered

NOTE: Comparisons done using t-test for continuous variables and chi-square or univariate logistic regression for categorical values. Bolded p-values are considered significant with a p-value of 0.05 or less.

In terms of self-reported history, almost two-thirds of individuals reported a prior parasitic infection (63.8%), with roundworm (34%) and schistosomiasis (22.7%) being the most commonly reported. Interestingly, only 47 (15.8%) of those reporting a history of schistosomiasis tested positive for anti-SEA antibodies, possibly signifying a more remote history and clearance of antibodies due to treatment. There was a positive association between self-reported hookworm and LID-1 seropositivity (X^2^ = 4.37; p = 0.03). For the option ‘other diseases’ five people responded, which included the following answers: 2 for leishmaniasis, 1 for toxoplasma, 1 for urinary infection, and 1 response that was unknown. In this study, 117 participants had missing data for reported past parasitic infection because they marked ‘don’t know’(97), ‘refuse to answer’ (15), or had not answered the question at all (5).

### Multivariate analysis

Final logistic regression models are shown in Table 3 and use the categorized form of the self-reported parasites. Two other models for each outcome were run and can be found in supplementary S1 and S2 Tables, comprising the forms of each individual parasite and all parasites summarized into the singular variable ‘parasitic infection’. The final variables for each model type were those not dropped during backwards selection and the exposures kept a priori (‘Strongyloides antibody [Anti-NIE]’, ‘Schistosomiasis antibody [Anti-SEA]’, and self-reported parasites). Since the anti-SEA variable and self-reported parasite variable for schistosomiasis had the potential for multi-collinearity, we ran additional permutations of the selected models. This included the original form (with both anti-SEA antibody and the self-reported schistosomiasis), a version of the model with only the anti-SEA antibody, and a version with only the schistosomiasis self-report variable. There was little to no effective change, so we determined that there was no concern for collinearity with the originally selected model. **S3** and S4 Tables show these permutations and can be found in the Supplementary Section.

**Table 3:** Multivariate Logistic Regression with POSITIVE ANTI-LID-1 and HISTORY OF HD as outcomes (serological results and categorized parasites).

	Pos Anti-LID-1	History of HD
	aOR (95% CI)	aOR (95% CI)
*S. mansoni* antibody positive (anti-SEA)	0.79 (0.38, 1.61)	1.26 (0.63, 2.50)
*S. stercoralis* antibody positive (anti-NIE)	1.57 (0.69, 3.57)	0.40 (0.09, 1.70)
Self-reported schistosomiasis	1.02 (0.61, 1.70)	0.88 (0.50, 1.53)
Self-reported helminth infection (other than *S. mansoni*)	1.15 (0.74, 1.78)	**1.77 (1.08, 2.90)**
Self-reported protozoa	0.76 (0.37, 1.57)	0.75 (0.35, 1.62)
Race (non-white)	1.20 (0.68, 2.10)	
Adult age (15 or older)		**7.80 (1.05, 57.64)**
Sex as female	.	0.64 (0.39, 1.051)
Rural Residence	**1.79 (1.07, 2.99)**	**1.97 (1.14, 3.38)**

NOTE: Adjusted odds ratio listed with confidence intervals (in parentheses) of exposures involving positive serological results of helminth infections, categorized self-reported past or present infections, and best fit demographic variables. Categorization of parasites was as follows: self-reported S.mansoni infection, self-reported Helminth Infections other than *S.mansoni* (Roundworm, Hookworm, Pinworm, Tapeworm), and self-reported Protozoa Infections (Giardia, Amoeba, Other). * p < 0.05

Neither helminth serological markers showed a statistically significant association with anti-LID-1 seropositivity. Anti-NIE positivity (aOR 1.57; 95% CI:0.69 - 3.57) had a positive point estimate while anti-SEA positivity had a negative point estimate (aOR 0.79, 95% CI: 0.38 - 1.61). Self-reported history of parasitic infections (categorized by type) was not associated with anti-LID-1 positivity. Finally, participants with positive anti-LID-1 (aOR 1.79, 95% CI: 1.07, 2.99) were more likely to live in a rural locale than an urban area (**Table 3**).

For the outcome, History of HD, there were no statistically significant associations with either anti-SEA or anti-NIE. The point estimate was positive for (aOR 1.26, 95% CI: 0.63 - 2.50) *S. mansoni* seropositivity and negative for *S. stercoralis* (aOR 0.40, 95% CI: 0.09 - 1.70) positivity. On the other hand, self-reported history of non-*Schistosoma* helminth infection was associated with prior HD (aOR 1.77, 95% CI: 1.08 - 2.90), as was older age (15 and greater) (aOR: 7.80, 95% CI 1.06 - 57.6). Participants with prior history of HD (aOR 1.97, 95% CI: 1.14 - 3.38) were more likely to reside in rural areas than urban settings (Table 3).

## Discussion

Our study showed a substantial burden of HD and other NTDs in eastern Minas Gerais with more than 7% of a random sample having had prior HD and 7% with seroreactivity to *Mycobacterium leprae* through anti-LID-1 antibody. Seroreactivity *to S. mansoni* and *S. stercoralis* was observed in 5–12% of participants, and almost two-thirds of participants reported history of parasitic infection (both NTDs and others). Studies measuring anti-LID-1 reactivity in community samples are limited in the literature and none have used a multiplexed beaded assay (MBA) to identify seroreactivity of multiple infections simultaneously. Therefore, our study was a unique snapshot in these communities of Minas Gerais of serological response coupled with historical reports, allowing us to document overlap between the infections and identify epidemiologic trends that can guide more mechanistic studies.

In terms of anti-LID-1 seropositivity, one other study in Brazil found over 20% of a community population seropositive for anti-LID-1 [21]. This study appeared to be an outlier with our study more in line with a study on household contacts in Goiás, Brazil, which found 7% seropositivity [36]. Regarding *S.mansoni*, eastern Minas Gerais remains endemic for schistosomiasis [13,35], and was not only highly self-reported in our study at 23% but 12% of individuals had a positive anti-SEA IgG, signifying recently resolved or active *S. mansoni* infections. *S. stercoralis*, a soil-transmitted helminth found mostly in tropical/subtropical zones with moist soil and poor sanitation, was the other directly measured helminth infection in this study [37]. A positive serological result typically reflects a recent or active infection, as anti-NIE IgG disappears over time after treatment, so presumably seropositivity is occurring prior to treatment [32]. Five percent of our study sample were anti-NIE seropositive showing ongoing transmission in the community.

Co-infections with helminths, particularly soil-transmitted infections and schistosomiasis, have been shown to have epidemiologic and immunologic associations with HD at the time of diagnosis in prior research [12,13,17,18]. Our prior work in this region demonstrated a strong association between active *S. mansoni* infection and HD (aOR 8.69, 95% CI: 1.50 – 50.51) when comparing cases to household contacts [13]. Diniz et al. showed an association with soil-transmitted helminths and leprosy, especially evident in multibacillary cases with a shift to the Th2 cytokines in co-infected individuals [12]. And, while the results were marginal and not statistically significant a longitudinal study in India did find an increased likelihood of HD among HHC who tested positive for soil-transmitted helminths compared to the HHC who were negative [38]. They also found that co-infected individuals had an immune profile consistent with a Th2 shift, suggesting a modulation from the helminth.

However, none of the analyses in this manuscript showed a statistically significant overlap with serological markers of helminth infection and either past HD or anti-LID-1. This could reflect the limitations of the cross-sectional nature of our study, the fact that seropositive individuals may represent treated helminth infection in the past, and in the case of prior HD, the fact that we cannot assess temporality of the infections. The lack of association between anti-LID-1 positivity and helminth positivity may also suggest that helminth infections are not more likely in *M. leprae* infected individuals but may be more likely to occur in those who develop disease as shown in prior studies. While Brazil has both a schistosomiasis control program and a soil-transmitted helminth control program with guidance based on prevalence in the community, Minas Gerais does not qualify for preventative chemotherapy due to lower endemicity.Therefore, unless the participants were tested by stool exam, it is unlikely that they were treated for either schistosomiasis or soil-transmitted helminths [39,40].

However, it was interesting that the point-estimates for anti-NIE, when compared to either anti-LID-1 positivity or past HD, had opposite findings. Anti-NIE seroreactivity was positively associated with anti-LID-1 (aOR 1.57, 95% CI: 0.69-3.57) and negatively associated with history of HD (aOR 0.40, 95% CI: 0.09 – 1.70). While this could have been purely by chance as evidenced by the aOR crossing 1, it may be related to the fact that those with HD were likely treated with ivermectin due to the risk of *Strongyloides* hyperinfection syndrome in the setting of prednisone use for either Type 1 or Type 2 reaction [41,42].

To have a better idea of prior exposures in addition to the measured antibodies, we asked participants to report any prior diagnoses of parasitic infections. Interestingly, self-reported worm infections (excluding schistosomiasis) had a statistically significant positive association with history of HD (aOR 1.77, 95% CI: 1.08 – 2.90), while hookworm alone had a statistically significant positive association with anti-LID-1 seropositivity (OR 3.97, 95% CI: 1.08-14.7; p = 0.03). However, with very small numbers, specifically only 13 participants reporting a history of hookworm infection and with 3 of those individuals being anti-LID-1 positive, this limits interpretability of this difference.

We did not find an association with the category self-reported history of protozoa to either outcome of interest. There are few studies related to co-infection between HD and protozoa, one being that *Toxoplasma gondii* could potentially increase the chances of Type 2 HD reaction [43]. More direct measurement of intestinal protozoa at the time of HD diagnosis could better determine an association. Lastly, the BCG vaccine has been shown to reduce the likelihood of HD in household contacts [25]. However, the presence of a BCG scar, indicating vaccination, did not differ across anti-LID-1 positive or negative individuals, suggesting that past vaccination was not associated with reduced risk of *M. leprae* infection.

In terms of non-infectious variables, there was a consistent significant association across all the models demonstrating a positive relationship between rural residence and both anti-LID-1 positivity and past HD. This result could be reflective of the endemicity in the rural areas and/or another risk factor such as environmental exposures like armadillos which are a known zoonotic reservoir [4,44–46].

There were several limitations of this study. Firstly, this analysis is a cross-sectional study, therefore findings may not reflect biologic associations. The variables related to having a history of parasites as well as history of past HD diagnosis involved recall bias due to the nature of self-reported data. Additionally, the question for the history of HD was missing nearly 300 responses, which may have impacted the power of the analysis for this outcome. There may have been some bias in participant recruitment since the households were allowed to choose the family member to participate, and may have been the most willing member.

## Conclusion

This study utilized a novel multiplex bead assay to simultaneously measure seroreactivity to *M. leprae*, schistosomiasis, and strongyloidiasis in a community sample and to examine associations between the infections. While we did not find strong associations, the degree of overlap of infections and baseline seropositivity of three NTDs in this region are notable. Also important to note was the ease of collecting a blood spot with the MBA tool and how it effectively and quickly provided information about multiple diseases. This could provide a way to integrate leprosy into other NTD control strategies globally. The strong association with rural residence also warrants further investigation by geospatial techniques to identify spatial risks or environmental risk factors. These epidemiologic tools, thus, hold promise for wider use in other areas to understand the prevalence of *M. leprae* infection and other infections that may impact disease epidemiology.

## Supporting information

S1 Table Multivariate Logistic Regression with POSITIVE ANTI-LID-1 and HISTORY OF HANSEN’S DISEASE against co-infection (*serological results and past parasitic infection*) and demographic risk factors. (DOCX)

S2 Table Multivariable logistic regression (MLR) with POSITIVE ANTI-LID-1 and HISTORY OF HANSEN’S DISEASE against co-infection (serological results and *all individual parasites*) and demographic risk factors. (DOCX)

S3 Table Checking effects size change among different model permutations for the outcome of POSITIVE ANTI-LID-1 (aOR). (DOCX)

S4 Table Checking effects size change among different model permutations for the outcome of HISTORY OF HANSEN’S DISEASE (aOR). (DOCX)

S1. Data Deidentified database used in the statistical analyses. (PDF)
