# Supplementary material for: Mycobacterium leprae infection, Hansen’s disease, and helminth infections: A cross-sectional study in southeastern Brazil
Source: PLoS Negl Trop Dis. 2026 Oct 1;20(10):e0013285. doi: 10.1371/journal.pntd.0013285 (PMC13693981; doi:10.1371/journal.pntd.0013285)
Supplement: Supplementary file 1 [file pntd.0013285.s005.pdf]

Study ID	Esta área é ru	Quantos	Qual é o seu sex	Como você d	Outros: Othe	Quantas pess
HMM0448	Urbana	55	Feminino	Branco		2
HMM0342	Urbana	41	Masculino	Branco		3
HMM0321	Urbana	47	Feminino	Pardo		3
HMM0322	Urbana	85	Feminino	Negro		1
HMM0323	Urbana	45	Feminino	Pardo		2
HMM0324	Urbana	39	Masculino	Branco		6
HMM0325	Urbana	17	Feminino	Pardo		2
HMM0326	Urbana	7	Masculino	Pardo		6
HMM0327	Urbana	4	Masculino	Pardo		6
HMM0328	Urbana	32	Feminino	Negro		6
HMM0329	Urbana	81	Masculino	Pardo		2
HMM0330	Urbana	47	Feminino	Pardo		6
HMM0331	Urbana	53	Feminino	Pardo		3
HMM0335	Urbana	6	Masculino	Negro		3
HMM0336	Urbana	20	Feminino	Negro		6
HMM0337	Urbana	7	Feminino	Pardo		6
HMM0338	Urbana	51	Feminino	Pardo		6
HMM0339	Urbana	44	Feminino	Negro		4
HMM0340	Urbana	21	Masculino	Pardo		6
HMM0341	Urbana	83	Masculino	Pardo		2
HMM0244	Urbana	88	Feminino	Pardo		2
HMM0245	Urbana	36	Feminino	Pardo		2
HMM0246	Urbana	75	Masculino	Negro		2
HMM0247	Urbana	45	Feminino	Pardo		5
HMM0248	Urbana	46	Feminino	Pardo		2
HMM0249	Urbana	69	Feminino	Branco		2
HMM0250	Urbana	55	Feminino	Pardo		3
HMM0251	Urbana	63	Feminino	Negro		5
HMM0252	Urbana	61	Feminino	Pardo		3
HMM0253	Urbana	79	Masculino	Pardo		1
HMM0254	Urbana	49	Feminino	Pardo		5
HMM0255	Urbana	40	Masculino	Pardo		6
HMM0256	Urbana	68	Masculino	Pardo		2
HMM0257	Urbana	69	Masculino	Pardo		2
HMM0258	Urbana	15	Masculino	Branco		3
HMM0259	Urbana	64	Feminino	Negro		2
HMM0260	Urbana	70	Masculino	Negro		2
HMM0261	Urbana	33	Feminino	Pardo		3
HMM0262	Urbana	42	Masculino	Negro		3
HMM0263	Urbana	74	Feminino	Negro		2
HMM0264	Urbana	50	Feminino	Pardo		2
HMM0265	Urbana	66	Feminino	Negro		2

HMM0266	Urbana	64	Feminino	Negro	2
HMM0267	Urbana	18	Masculino	Pardo	3
HMM0268	Urbana	42	Feminino	Pardo	3
HMM0269	Urbana	65	Feminino	Negro	2
HMM0270	Urbana	20	Masculino	Negro	3
HMM0271	Urbana	82	Masculino	Pardo	2
HMM0272	Urbana	59	Feminino	Negro	5
HMM0273	Urbana	54	Feminino	Pardo	3
HMM0274	Urbana	39	Feminino	Negro	4
HMM0275	Urbana	62	Feminino	Branco	3
HMM0276	Urbana	34	Feminino	Pardo	6
HMM0277	Urbana	9	Masculino	Negro	6
HMM0278	Urbana	51	Feminino	Pardo	3
HMM0279	Urbana	61	Feminino	Pardo	3
HMM0280	Urbana	43	Feminino	Negro	3
HMM0281	Urbana	52	Feminino	Negro	2
HMM0282	Urbana	50	Feminino	Negro	6
HMM0283	Urbana	51	Feminino	Pardo	3
HMM0284	Urbana	71	Masculino	Negro	3
HMM0285	Urbana	42	Masculino	Negro	3
HMM0286	Urbana	54	Feminino	Negro	3
HMM0287	Urbana	91	Feminino	Indigeno	2
HMM0288	Urbana	54	Feminino	Negro	4
HMM0289	Urbana	67	Feminino	Pardo	7
HMM0290	Urbana	74	Feminino	Pardo	1
HMM0291	Urbana	66	Feminino	Pardo	2
HMM0292	Urbana	38	Masculino	Negro	2
HMM0293	Urbana	66	Feminino	Negro	1
HMM0294	Urbana	15	Feminino	Pardo	6
HMM0295	Urbana	12	Feminino	Pardo	5
HMM0296	Urbana	71	Feminino	Pardo	2
HMM0297	Urbana	51	Feminino	Negro	2
HMM0298	Urbana	27	Feminino	Pardo	4
HMM0299	Urbana	58	Feminino	Branco	4
HMM0300	Urbana	37	Feminino	Negro	3
HMM0301	Urbana	67	Feminino	Pardo	3
HMM0302	Urbana	56	Feminino	Branco	3
HMM0303	Urbana	74	Masculino	Branco	2
HMM0304	Urbana	30	Masculino	Negro	4
HMM0305	Urbana	32	Feminino	Pardo	4
HMM0306	Urbana	52	Feminino	Pardo	2
HMM0307	Urbana	63	Masculino	Pardo	1
HMM0308	Urbana	72	Feminino	Branco	1

HMM0309	Urbana	50	Feminino	Pardo	3
HMM0310	Urbana	69	Feminino	Branco	2
HMM0311	Urbana	49	Masculino	Pardo	2
HMM0312	Urbana	67	Masculino	Pardo	2
HMM0313	Urbana	12	Feminino	Negro	5
HMM0314	Urbana	42	Feminino	Pardo	4
HMM0315	Urbana	92	Masculino	Pardo	2
HMM0316	Urbana	68	Feminino	Negro	2
HMM0317	Urbana	57	Masculino	Pardo	3
HMM0318	Urbana	73	Feminino	Pardo	2
HMM0319	Urbana	56	Feminino	Negro	2
HMM0320	Urbana	65	Feminino	Pardo	3
HMM0001	Urbana	70	Masculino	Pardo	3
HMM0002	Urbana	30	Feminino	Pardo	2
HMM0003	Urbana	20	Feminino	Pardo	2
HMM0004	Urbana	53	Masculino	Negro	3
HMM0005	Urbana	28	Masculino	Negro	3
HMM0006	Urbana	71	Masculino	Negro	3
HMM0007	Urbana	41	Masculino	Negro	4
HMM0008	Urbana	58	Masculino	Pardo	2
HMM0009	Urbana	63	Feminino	Negro	11
HMM0010	Urbana	9	Masculino	Pardo	5
HMM0011	Urbana	39	Masculino	Pardo	4
HMM0012	Urbana	44	Masculino	Pardo	4
HMM0013	Urbana	68	Feminino	Pardo	4
HMM0014	Urbana	62	Feminino	Negro	2
HMM0015	Urbana	59	Feminino	Pardo	2
HMM0016	Urbana	65	Feminino	Pardo	1
HMM0017	Urbana	75	Feminino	Branco	1
HMM0018	Urbana	75	Masculino	Pardo	3
HMM0019	Urbana	59	Feminino	Pardo	2
HMM0020	Urbana	51	Feminino	Pardo	1
HMM0021	Urbana	48	Feminino	Pardo	4
HMM0022	Urbana	56	Masculino	Pardo	2
HMM0023	Urbana	40	Masculino	Pardo	1
HMM0024	Urbana	59	Feminino	Pardo	1
HMM0450	Urbana	69	Feminino	Pardo	1
HMM0449	Urbana	63	Feminino	Pardo	2
HMM0451	Urbana	62	Feminino	Negro	1
HMM0452	Urbana	45	Feminino	Pardo	2
HMM0453	Urbana	26	Masculino	Negro	2
HMM0456	Urbana	54	Feminino	Negro	4
HMM0457	Urbana	78	Feminino	Pardo	1

HMM0458	Urbana	75	Feminino	Pardo	1
HMM0460	Urbana	70	Feminino	Pardo	4
HMM0459	Urbana	58	Feminino	Pardo	2
HMM0461	Urbana	53	Feminino	Branco	2
HMM0464	Urbana	47	Feminino	Branco	
HMM0465	Urbana	51	Masculino	Pardo	3
HMM0466	Urbana	64	Feminino	Negro	5
HMM0467	Urbana	67	Masculino	Negro	
HMM0468	Urbana	56	Masculino	Pardo	4
HMM0469	Urbana	33	Feminino	Negro	5
HMM0470	Urbana	39	Feminino	Negro	3
HMM0471	Urbana	57	Feminino	Pardo	6
HMM0472	Urbana	54	Feminino	Branco	2
HMM0473	Urbana	45	Feminino	Negro	4
HMM0474	Urbana	48	Feminino	Negro	2
HMM0475	Urbana	64	Masculino	Pardo	2
HMM0476	Urbana	72	Feminino	Pardo	2
HMM0477	Urbana	48	Feminino	Pardo	2
HMM0478	Urbana	57	Masculino	Branco	4
HMM0479	Urbana	75	Feminino	Pardo	3
HMM0480	Urbana	45	Feminino	Pardo	4
HMM0481	Urbana	60	Feminino	Pardo	2
HMM0482	Urbana	51	Masculino	Pardo	2
HMM0483	Urbana	56	Feminino	Pardo	1
HMM0484	Urbana	50	Feminino	Pardo	3
HMM0485	Urbana	60	Feminino	Pardo	1
HMM0486	Urbana	58	Feminino	Pardo	3
HMM0487	Urbana	67	Feminino	Pardo	4
HMM0488	Urbana	37	Feminino	Negro	4
HMM0489	Rural	77	Feminino	Pardo	2
HMM0490	Urbana	54	Masculino	Pardo	2
HMM0491	Urbana	36	Feminino	Branco	4
HMM0492	Urbana	73	Feminino	Pardo	8
HMM0493	Urbana	38	Feminino	Pardo	3
HMM0494	Urbana	23	Feminino	Pardo	3
HMM0495	Urbana	61	Masculino	Negro	2
HMM0496	Urbana	60	Feminino	Negro	1
HMM0497	Urbana	53	Masculino	Branco	2
HMM0498	Urbana	49	Feminino	Pardo	1
HMM0499	Urbana	60	Feminino	Negro	4
HMM0518	Urbana	53	Masculino	Negro	2
HMM0519	Urbana	49	Feminino	Pardo	4
HMM0520	Urbana	45	Feminino	Negro	4

HMM0521	Urbana	56	Feminino	Pardo	2
HMM0522	Urbana	69	Masculino	Pardo	4
HMM0523	Urbana	53	Feminino	Negro	4
HMM0524	Urbana	62	Feminino	Pardo	1
HMM0525	Rural	37	Masculino	Pardo	4
HMM0526	Urbana	43	Feminino	Negro	3
HMM0527	Urbana	54	Feminino	Pardo	3
HMM0528	Urbana	21	Feminino	Pardo	4
HMM0529	Urbana	35	Feminino	Pardo	7
HMM0530	Urbana	60	Feminino	Negro	3
HMM0425	Rural	79	Masculino	Pardo	2
HMM0426	Rural	48	Masculino	Pardo	1
HMM0427	Rural	24	Masculino	Pardo	3
HMM0428	Rural	41	Masculino	Pardo	4
HMM0429	Urbana	56	Feminino	Negro	3
HMM0389	Urbana	35	Feminino	Pardo	3
HMM0390	Urbana	62	Feminino	Negro	3
HMM0391	Rural	32	Feminino	Pardo	2
HMM0392	Urbana	53	Feminino	Branco	4
HMM0393	Urbana	55	Masculino	Negro	1
HMM0394	Urbana	65	Feminino	Pardo	1
HMM0395	Urbana	74	Feminino	Pardo	3
HMM0396	Rural	10	Masculino	Indigeno	6
HMM0397	Urbana	30	Feminino	Pardo	3
HMM0398	Rural	12	Masculino	Negro	3
HMM0399	Urbana	75	Masculino	Negro	2
HMM0430	Rural	44	Feminino	Pardo	3
HMM0431	Rural	68	Masculino	Não sei	2
HMM0432	Rural	40	Masculino	Pardo	4
HMM0433	Rural	53	Feminino	Pardo	3
HMM0434	Urbana	36	Masculino	Negro	4
HMM0400	Urbana	83	Masculino	Pardo	1
HMM0435	Urbana	57	Feminino	Pardo	2
HMM0401	Urbana	43	Masculino	Pardo	1
HMM0436	Urbana	55	Feminino	Branco	3
HMM0402	Urbana	57	Feminino	Negro	4
HMM0437	Urbana	62	Feminino	Branco	1
HMM0403	Urbana	30	Feminino	Branco	4
HMM0438	Urbana	44	Masculino	Negro	3
HMM0404	Urbana	8	Masculino	Pardo	4
HMM0405	Urbana	69	Masculino	Pardo	1
HMM0406	Urbana	7	Feminino	Negro	4
HMM0407	Urbana	42	Feminino	Negro	4

HMM0408	Rural	74	Feminino	Pardo	3
HMM0409	Urbana	54	Masculino	Pardo	2
HMM0410	Urbana	5	Feminino	Pardo	3
HMM0411	Rural	43	Masculino	Negro	5
HMM0500	Rural	69	Feminino	Branco	1
HMM0412	Urbana	41	Feminino	Negro	5
HMM0501	Rural	57	Feminino	Negro	2
HMM0413	Urbana	70	Feminino	Pardo	2
HMM0414	Urbana	61	Masculino	Branco	1
HMM0415	Rural	45	Feminino	Pardo	2
HMM0416	Rural	46	Feminino	Branco	3
HMM0417	Urbana	68	Feminino	Branco	2
HMM0418	Urbana	38	Feminino	Pardo	3
HMM0419	Urbana	50	Feminino	Negro	5
HMM0420	Urbana	40	Feminino	Pardo	3
HMM0421	Urbana	6	Feminino	Pardo	3
HMM0422	Rural	63	Feminino	Pardo	2
HMM0423	Urbana	55	Feminino	Negro	2
HMM0424	Urbana	49	Feminino	Branco	4
HMM0502	Rural	62	Feminino	Negro	2
HMM0503	Rural	65	Feminino	Branco	2
HMM0119	Urbana	63	Feminino	Branco	2
HMM0120	Urbana	72	Feminino	Pardo	3
HMM0121	Urbana	71	Feminino	Pardo	2
HMM0122	Urbana	65	Feminino	Branco	4
HMM0123	Urbana	69	Feminino	Pardo	2
HMM0124	Urbana	53	Masculino	Pardo	1
HMM0125	Urbana	7	Masculino	Pardo	5
HMM0126	Urbana	49	Feminino	Pardo	5
HMM0127	Urbana	76	Masculino	Pardo	1
HMM0128	Urbana	73	Feminino	Negro	2
HMM0129	Urbana	57	Masculino	Branco	1
HMM0130	Urbana	59	Feminino	Negro	4
HMM0131	Urbana	12	Feminino	Branco	2
HMM0132	Urbana	9	Feminino	Pardo	3
HMM0133	Urbana	65	Feminino	Pardo	3
HMM0134	Urbana	74	Masculino	Negro	2
HMM0135	Urbana	55	Feminino	Negro	4
HMM0136	Urbana	11	Feminino	Branco	3
HMM0137	Urbana	49	Feminino	Branco	2
HMM0138	Urbana	44	Masculino	Pardo	1
HMM0139	Urbana	42	Masculino	Negro	2
HMM0140	Urbana	50	Feminino	Pardo	3

HMM0141	Urbana	70	Feminino	Pardo	3
HMM0142	Urbana	60	Masculino	Pardo	1
HMM0143	Urbana	70	Feminino	Branco	2
HMM0144	Urbana	57	Feminino	Pardo	3
HMM0145	Urbana	31	Feminino	Negro	4
HMM0146	Urbana	69	Feminino	Negro	2
HMM0147	Urbana	64	Feminino	Pardo	1
HMM0148	Urbana	47	Feminino	Pardo	4
HMM0149	Urbana	47	Masculino	Pardo	3
HMM0150	Urbana	83	Feminino	Branco	1
HMM0151	Urbana	60	Masculino	Branco	1
HMM0152	Urbana	52	Feminino	Negro	3
HMM0153	Urbana	62	Feminino	Branco	3
HMM0154	Urbana	75	Feminino	Pardo	3
HMM0504	Rural	40	Feminino	Pardo	4
HMM0514	Rural	8	Feminino	Pardo	9
HMM0721	Rural	19	Feminino	Pardo	2
HMM0720	Rural	53	Masculino	Pardo	3
HMM0505	Rural	88	Feminino	Pardo	9
HMM0723	Rural	54	Masculino	Pardo	3
HMM0507	Rural	66	Masculino	Negro	2
HMM0506	Rural	50	Feminino	Pardo	8
HMM0722	Rural	53	Masculino	Pardo	2
HMM0725	Rural	72	Feminino	Pardo	2
HMM0509	Rural	64	Feminino	Pardo	3
HMM0724	Rural	52	Masculino	Pardo	5
HMM0508	Rural	51	Masculino	Pardo	4
HMM0727	Rural	44	Feminino	Branco	2
HMM0511	Rural	72	Feminino	Branco	2
HMM0726	Rural	70	Masculino	Pardo	3
HMM0729	Rural	45	Masculino	Pardo	4
HMM0510	Rural	71	Masculino	Pardo	2
HMM0728	Rural	26	Feminino	Pardo	2
HMM0512	Rural	56	Feminino	Pardo	3
HMM0730	Rural	10	Feminino	Pardo	7
HMM0515	Rural	50	Feminino	Pardo	2
HMM0513	Rural	59	Feminino	Pardo	9
HMM0360	Urbana	57	Masculino	Branco	3
HMM0439	Urbana	80	Masculino	Branco	1
HMM0440	Urbana	48	Feminino	Branco	6
HMM0441	Urbana	59	Masculino	Branco	2
HMM0442	Urbana	43	Masculino	Pardo	3
HMM0443	Urbana	49	Feminino	Pardo	2

HMM0444	Urbana	71	Feminino	Branco	2
HMM0447	Urbana	62	Feminino	Pardo	3
HMM0445	Urbana	38	Masculino	Negro	1
HMM0446	Urbana	67	Feminino	Branco	3
HMM0334	Urbana	7	Masculino	Branco	4
HMM0343	Urbana	72	Feminino	Branco	2
HMM0155	Urbana	56	Masculino	Não sei	5
HMM0156	Urbana	40	Feminino	Pardo	7
HMM0157	Urbana	19	Feminino	Pardo	3
HMM0516	Rural	54	Feminino	Negro	3
HMM0517	Rural	71	Masculino	Pardo	2
HMM0601	Rural	24	Masculino	Pardo	4
HMM0600	Rural	42	Masculino	Branco	4
HMM0603	Rural	63	Feminino	Branco	3
HMM0602	Rural	67	Feminino	Negro	2
HMM0604	Rural	68	Feminino	Pardo	1
HMM0701	Rural	62	Feminino	Branco	2
HMM0700	Rural	85	Feminino	Branco	2
HMM0703	Rural	56	Masculino	Branco	4
HMM0702	Rural	19	Feminino	Pardo	3
HMM0344	Urbana	70	Feminino	Negro	3
HMM0345	Rural	75	Feminino	Pardo	5
HMM0346	Urbana	67	Feminino	Branco	2
HMM0347	Rural	67	Feminino	Pardo	3
HMM0348	Urbana	70	Feminino	Negro	2
HMM0349	Urbana	73	Feminino	Branco	1
HMM0361	Urbana	56	Masculino	Branco	2
HMM0362	Urbana	51	Feminino	Branco	1
HMM0363	Urbana	62	Masculino	Pardo	1
HMM0158	Urbana	55	Feminino	Pardo	2
HMM0350	Urbana	51	Feminino	Negro	3
HMM0159	Urbana	76	Feminino	Branco	2
HMM0351	Urbana	68	Feminino	Negro	3
HMM0160	Urbana	66	Feminino	Branco	1
HMM0352	Urbana	80	Masculino	Branco	3
HMM0161	Urbana	39	Feminino	Negro	3
HMM0353	Urbana	43	Feminino	Negro	3
HMM0162	Urbana	62	Feminino	Negro	2
HMM0163	Urbana	53	Feminino	Pardo	5
HMM0164	Urbana	66	Feminino	Pardo	2
HMM0165	Urbana	28	Masculino	Branco	28
HMM0166	Urbana	39	Feminino	Pardo	4
HMM0167	Urbana	52	Masculino	Negro	4

HMM0364	Urbana	77	Feminino	Pardo	2
HMM0168	Urbana	75	Feminino	Pardo	2
HMM0365	Urbana	65	Feminino	Pardo	3
HMM0169	Urbana	78	Masculino	Pardo	9
HMM0366	Urbana	65	Feminino	Pardo	3
HMM0170	Urbana	55	Masculino	Negro	2
HMM0171	Urbana	72	Masculino	Negro	2
HMM0367	Urbana	11	Masculino	Pardo	4
HMM0368	Urbana	45	Masculino	Pardo	1
HMM0704	Rural	59	Masculino	Pardo	2
HMM0172	Urbana	34	Feminino	Pardo	3
HMM0369	Urbana	41	Feminino	Pardo	2
HMM0705	Rural	34	Masculino	Pardo	6
HMM0173	Urbana	69	Masculino	Branco	2
HMM0174	Urbana	64	Masculino	Pardo	2
HMM0706	Rural	60	Masculino	Branco	3
HMM0175	Urbana	65	Feminino	Branco	2
HMM0707	Rural	52	Masculino	Pardo	2
HMM0176	Urbana	38	Feminino	Negro	4
HMM0708	Rural	70	Masculino	Pardo	1
HMM0709	Rural	58	Masculino	Pardo	1
HMM0710	Rural	71	Masculino	Pardo	2
HMM0711	Rural	57	Feminino	Pardo	3
HMM0712	Rural	46	Masculino	Pardo	2
HMM0713	Urbana	68	Masculino	Pardo	3
HMM0714	Rural	46	Feminino	Pardo	3
HMM0715	Rural	56	Masculino	Pardo	3
HMM0716	Rural	56	Feminino	Pardo	3
HMM0717	Rural	66	Masculino	Branco	4
HMM0718	Rural	50	Feminino	Pardo	3
HMM0719	Rural	42	Feminino	Negro	6
HMM0177	Urbana	7	Masculino	Pardo	3
HMM0178	Urbana	27	Feminino	Pardo	3
HMM0179	Urbana	37	Feminino	Pardo	4
HMM0180	Urbana	34	Feminino	Pardo	4
HMM0181	Urbana	59	Feminino	Pardo	4
HMM0182	Urbana	69	Feminino	Pardo	5
HMM0183	Urbana	14	Masculino	Negro	3
HMM0184	Urbana	58	Feminino	Pardo	3
HMM0185	Urbana	48	Feminino	Branco	3
HMM0186	Urbana	41	Feminino	Pardo	3
HMM0187	Urbana	51	Masculino	Pardo	2
HMM0188	Urbana	39	Masculino	Pardo	1

HMM0099	Urbana	59	Feminino	Pardo	6
HMM0100	Urbana	35	Feminino	Negro	4
HMM0101	Urbana	8	Masculino	Negro	4
HMM0102	Urbana	69	Feminino	Pardo	3
HMM0370	Urbana	68	Masculino	Pardo	2
HMM0371	Urbana	69	Masculino	Negro	2
HMM0372	Urbana	82	Masculino	Pardo	4
HMM0373	Urbana	67	Feminino	Branco	2
HMM0374	Urbana	65	Feminino	Pardo	3
HMM0375	Urbana	73	Feminino	Pardo	2
HMM0376	Urbana	75	Masculino	Branco	2
HMM0377	Rural	52	Feminino	Pardo	5
HMM0378	Rural	91	Masculino	Não sei	2
HMM0354	Urbana	32	Feminino	Pardo	3
HMM0189	Urbana	46	Feminino	Pardo	4
HMM0355	Urbana	77	Masculino	Pardo	4
HMM0356	Rural	58	Feminino	Negro	2
HMM0357	Urbana	42	Feminino	Branco	5
HMM0358	Recusou a re	43	Feminino	Negro	4
HMM0359	Não sei	34	Masculino	Negro	2
HMM0190	Urbana	26	Masculino	Negro	2
HMM0191	Urbana	75	Feminino	Branco	3
HMM0192	Urbana	44	Masculino	Negro	2
HMM0193	Urbana	47	Feminino	Branco	2
HMM0194	Urbana	81	Masculino	Branco	3
HMM0195	Urbana	69	Feminino	Negro	3
HMM0196	Urbana	9	Masculino	Pardo	4
HMM0197	Urbana	82	Feminino	Pardo	2
HMM0198	Urbana	13	Feminino	Pardo	4
HMM0379	Rural	50	Feminino	Branco	2
HMM0380	Urbana	68	Feminino	Branco	4
HMM0199	Urbana	27	Feminino	Pardo	4
HMM0200	Urbana	56	Masculino	Branco	4
HMM0201	Urbana	48	Masculino	Pardo	6
HMM0381	Urbana	58	Masculino	Pardo	2
HMM0382	Urbana	72	Masculino	Pardo	2
HMM0383	Urbana	54	Feminino	Negro	10
HMM0384	Rural	30	Feminino	Pardo	5
HMM0385	Urbana	71	Feminino	Pardo	2
HMM0386	Urbana	83	Feminino	Pardo	3
HMM0387	Urbana	8	Feminino	Pardo	3
HMM0388	Urbana	8	Feminino	Pardo	3
HMM0202	Urbana	59	Masculino	Branco	3

HMM0203	Urbana	62	Feminino	Negro	2
HMM0204	Urbana	72	Feminino	Negro	3
HMM0205	Urbana	51	Feminino	Pardo	4
HMM0206	Urbana	49	Feminino	Branco	3
HMM0207	Urbana	73	Feminino	Branco	2
HMM0208	Urbana	48	Masculino	Branco	Não informac
HMM0209	Urbana	61	Masculino	Branco	2
HMM0210	Urbana	20	Masculino	Branco	2
HMM0211	Urbana	50	Feminino	Branco	5
HMM0212	Urbana	52	Feminino	Pardo	4
HMM0213	Urbana	51	Masculino	Branco	2
HMM0214	Urbana	66	Masculino	Negro	1
HMM0215	Urbana	76	Feminino	Branco	4
HMM0103	Urbana	5	Feminino	Branco	4
HMM0105	Urbana	63	Feminino	Pardo	2
HMM0106	Urbana	62	Feminino	Pardo	2
HMM0531	Urbana	72	Masculino	Pardo	3
HMM0107	Urbana	58	Feminino	Branco	3
HMM0108	Urbana	50	Masculino	Pardo	2
HMM0109	Urbana	70	Feminino	Branco	1
HMM0532	Urbana	26	Feminino	Branco	2
HMM0110	Urbana	51	Masculino	Pardo	1
HMM0533	Urbana	30	Masculino	Pardo	3
HMM0534	Rural	19	Feminino	Pardo	5
HMM0111	Urbana	64	Feminino	Pardo	4
HMM0535	Urbana	56	Feminino	Negro	5
HMM0536	Urbana	27	Feminino	Pardo	4
HMM0112	Urbana	67	Feminino	Pardo	6
HMM0113	Urbana	58	Feminino	Branco	2
HMM0114	Urbana	53	Feminino	Pardo	4
HMM0115	Urbana	77	Feminino	Branco	2
HMM0116	Urbana	76	Masculino	Negro	2
HMM0117	Urbana	61	Masculino	Branco	1
HMM0118	Urbana	87	Feminino	Negro	3
HMM0025	Urbana	47	Feminino	Branco	5
HMM0027	Urbana	65	Feminino	Pardo	1
HMM0537	Urbana	58	Feminino	Branco	7
HMM0538	Urbana	20	Feminino	Pardo	5
HMM0539	Urbana	49	Feminino	Pardo	4
HMM0540	Urbana	56	Masculino	Negro	3
HMM0216	Urbana	36	Feminino	Negro	6
HMM0218	Urbana	52	Masculino	Branco	5
HMM0219	Urbana	38	Masculino	Pardo	2

HMM0220	Urbana	46	Feminino	Pardo	6
HMM0028	Urbana	48	Masculino	Negro	4
HMM0029	Urbana	58	Masculino	Pardo	2
HMM0030	Urbana	52	Feminino	Pardo	2
HMM0031	Urbana	61	Masculino	Negro	2
HMM0032	Urbana	63	Feminino	Negro	1
HMM0033	Urbana	41	Feminino	Negro	3
HMM0034	Urbana	63	Feminino	Pardo	2
HMM0035	Urbana	64	Masculino	Negro	4
HMM0036	Urbana	45	Feminino	Pardo	5
HMM0037	Urbana	46	Masculino	Pardo	3
HMM0038	Urbana	47	Feminino	Branco	2
HMM0039	Urbana	48	Feminino	Negro	2
HMM0040	Urbana	29	Feminino	Pardo	4
HMM0041	Urbana	63	Masculino	Negro	2
HMM0042	Urbana	58	Feminino	Pardo	1
HMM0043	Urbana	51	Feminino	Negro	6
HMM0044	Urbana	64	Feminino	Branco	2
HMM0045	Urbana	60	Feminino	Pardo	1
HMM0046	Urbana	17	Feminino	Pardo	3
HMM0047	Urbana	52	Masculino	Pardo	2
HMM0048	Urbana	34	Feminino	Negro	5
HMM0049	Urbana	58	Masculino	Branco	2
HMM0221	Urbana	63	Feminino	Branco	2
HMM0222	Urbana	43	Feminino	Branco	3
HMM0223	Urbana	61	Feminino	Branco	3
HMM0224	Urbana	60	Feminino	Branco	5
HMM0225	Urbana	64	Feminino	Branco	2
HMM0226	Urbana	58	Feminino	Negro	2
HMM0541	Urbana	66	Masculino	Pardo	2
HMM0542	Urbana	36	Feminino	Negro	4
HMM0543	Urbana	67	Masculino	Branco	4
HMM0544	Urbana	34	Feminino	Pardo	2
HMM0545	Urbana	47	Feminino	Pardo	2
HMM0546	Urbana	10	Feminino	Branco	2
HMM0547	Urbana	35	Feminino	Branco	2
HMM0548	Urbana	51	Feminino	Pardo	3
HMM0050	Urbana	71	Feminino	Branco	2
HMM0051	Urbana	64	Feminino	Pardo	2
HMM0052	Urbana	63	Feminino	Branco	4
HMM0053	Urbana	71	Feminino	Pardo	1
HMM0054	Urbana	85	Feminino	Pardo	3
HMM0055	Urbana	53	Feminino	Pardo	4

HMM0056	Urbana	69	Feminino	Negro	2
HMM0057	Urbana	30	Masculino	Pardo	4
HMM0058	Urbana	68	Feminino	Negro	1
HMM0059	Urbana	19	Feminino	Branco	5
HMM0060	Urbana	61	Feminino	Branco	2
HMM0061	Urbana	74	Masculino	Pardo	3
HMM0062	Urbana	61	Masculino	Branco	2
HMM0063	Urbana	59	Feminino	Pardo	2
HMM0065	Urbana	20	Feminino	Negro	3
HMM0066	Urbana	40	Feminino	Pardo	3
HMM0067	Urbana	56	Masculino	Branco	4
HMM0068	Urbana	62	Feminino	Branco	3
HMM0069	Urbana	63	Feminino	Branco	2
HMM0070	Urbana	12	Feminino	Branco	4
HMM0071	Urbana	7	Feminino	Branco	4
HMM0072	Urbana	37	Feminino	Branco	4
HMM0073	Urbana	11	Masculino	Pardo	4
HMM0074	Urbana	32	Feminino	Pardo	4
HMM0075	Urbana	61	Feminino	Negro	3
HMM0076	Urbana	65	Feminino	Pardo	3
HMM0078	Urbana	53	Feminino	Pardo	5
HMM0079	Urbana	63	Feminino	Pardo	2
HMM0080	Urbana	41	Masculino	Branco	1
HMM0081	Urbana	35	Masculino	Pardo	4
HMM0082	Urbana	84	Masculino	Branco	4
HMM0083	Urbana	52	Feminino	Pardo	4
HMM0084	Urbana	68	Feminino	Pardo	3
HMM0085	Urbana	42	Feminino	Branco	4
HMM0086	Urbana	70	Feminino	Pardo	2
HMM0087	Urbana	61	Feminino	Pardo	2
HMM0088	Urbana	23	Masculino	Pardo	3
HMM0089	Urbana	7	Masculino	Negro	4
HMM0090	Urbana	49	Feminino	Pardo	4
HMM0091	Urbana	14	Feminino	Pardo	4
HMM0092	Urbana	59	Feminino	Branco	3
HMM0093	Urbana	13	Feminino	Pardo	13
HMM0094	Urbana	31	Feminino	Pardo	4
HMM0549	Urbana	56	Masculino	Negro	4
HMM0550	Urbana	41	Feminino	Branco	4
HMM0551	Urbana	50	Feminino	Pardo	2
HMM0552	Urbana	48	Feminino	Pardo	2
HMM0553	Rural	48	Feminino	Pardo	3
HMM0554	Urbana	41	Feminino	Pardo	2

HMM0555	Urbana	44	Feminino	Pardo	2
HMM0095	Urbana	41	Feminino	Pardo	5
HMM0096	Urbana	16	Feminino	Negro	4
HMM0097	Urbana	49	Masculino	Branco	4
HMM0098	Urbana	39	Feminino	Branco	4
HMM0556	Urbana	32	Feminino	Pardo	2
HMM0557	Urbana	46	Masculino	Pardo	4
HMM0558	Urbana	53	Masculino	Pardo	2
HMM0559	Urbana	55	Feminino	Negro	3
HMM0560	Urbana	38	Feminino	Branco	4
HMM0561	Urbana	27	Feminino	Pardo	3
HMM0562	Urbana	34	Masculino	Pardo	2
HMM0563	Urbana	9	Feminino	Pardo	4
HMM0564	Urbana	36	Feminino	Pardo	4
HMM0565	Urbana	31	Masculino	Branco	4
HMM0566	Urbana	61	Masculino	Branco	3
HMM0567	Urbana	76	Feminino	Negro	2
HMM0568	Urbana	54	Feminino	Pardo	4
HMM0569	Urbana	10	Feminino	Pardo	4
HMM0570	Urbana	61	Feminino	Pardo	6
HMM0571	Urbana	67	Feminino	Pardo	4
HMM0572	Urbana	84	Masculino	Negro	4
HMM0573	Urbana	51	Feminino	Branco	3
HMM0574	Urbana	52	Masculino	Negro	1
HMM0575	Urbana	10	Feminino	Negro	2
HMM0576	Urbana	26	Feminino	Negro	2
HMM0577	Urbana	55	Masculino	Pardo	4
HMM0578	Urbana	46	Masculino	Pardo	2
HMM0579	Urbana	39	Masculino	Negro	4
HMM0580	Urbana	50	Masculino	Pardo	3
HMM0581	Urbana	37	Masculino	Pardo	2
HMM0582	Urbana	58	Feminino	Pardo	3
HMM0583	Urbana	39	Feminino	Pardo	4
HMM0584	Urbana	47	Masculino	Pardo	4
HMM0454	Urbana	75	Feminino	Pardo	3
HMM0455	Urbana	57	Masculino	Pardo	4
HMM0462	Urbana	43	Masculino	Pardo	4
HMM0463	Urbana	40	Feminino	Indigeno	4
HMM0235	Urbana	59	Feminino	Branco	2
HMM0237	Urbana	66	Feminino	Pardo	3
HMM0238	Urbana	72	Masculino	Pardo	3
HMM0812	Urbana	29	Masculino	Pardo	5
HMM0811	Urbana	75	Feminino	Negro	2

HMM0810	Urbana	18	Masculino	Pardo	2
HMM0809	Urbana	19	Masculino	Pardo	3
HMM0747	Urbana	72	Feminino	Branco	2
HMM0744	Urbana	48	Feminino	Branco	2
HMM0806	Urbana	27	Masculino	Pardo	2
HMM0743	Urbana	53	Feminino	Pardo	5
HMM0745	Urbana	77	Feminino	Pardo	1
HMM0751	Urbana	57	Masculino	Pardo	6
HMM0807	Urbana	13	Masculino	Branco	4
HMM0808	Urbana	19	Feminino	Pardo	4
HMM0805	Urbana	15	Masculino	Pardo	3
HMM0804	Urbana	8	Masculino	Pardo	5
HMM0803	Urbana	24	Feminino	Pardo	5
HMM0802	Urbana	5	Masculino	Pardo	4
HMM0801	Urbana	40	Masculino	Branco	5
HMM0800	Urbana	55	Feminino	Pardo	2
HMM0799	Urbana	12	Feminino	Pardo	2
HMM0798	Urbana	17	Feminino	Pardo	3
HMM0797	Urbana	76	Feminino	Pardo	1
HMM0796	Urbana	35	Masculino	Pardo	5
HMM0788	Urbana	36	Feminino	Pardo	5
HMM0684	Urbana	44	Masculino	Pardo	1
HMM0585	Urbana	62	Feminino	Pardo	1
HMM0586	Urbana	55	Feminino	Negro	2
HMM0587	Rural	47	Masculino	Negro	3
HMM0681	Urbana	75	Feminino	Branco	1
HMM0682	Urbana	27	Masculino	Pardo	3
HMM0683	Urbana	34	Feminino	Pardo	3
HMM0685	Urbana	37	Masculino	Pardo	2
HMM0659	Urbana	73	Feminino	Branco	2
HMM0660	Urbana	46	Feminino	Negro	5
HMM0661	Rural	58	Masculino	Negro	4
HMM0662	Urbana	70	Feminino	Negro	2
HMM0663	Urbana	72	Feminino	Pardo	3
HMM0664	Urbana	30	Masculino	Branco	4
HMM0665	Urbana	73	Masculino	Pardo	3
HMM0666	Urbana	77	Masculino	Branco	2
HMM0667	Urbana	66	Feminino	Pardo	3
HMM0668	Urbana	9	Feminino	Pardo	3
HMM0669	Urbana	34	Feminino	Branco	4
HMM0795	Urbana	77	Feminino	Pardo	4
HMM0794	Urbana	54	Masculino	Pardo	5
HMM0789	Urbana	19	Feminino	Pardo	4

HMM0793	Urbana	70	Feminino	Pardo	1
HMM0792	Urbana	52	Feminino	Negro	7
HMM0791	Urbana	33	Feminino	Negro	4
HMM0790	Urbana	53	Masculino	Branco	1
HMM0919	Urbana	28	Feminino	Pardo	6
HMM0948	Urbana	5	Feminino	Pardo	3
HMM0787	Urbana	56	Feminino	Pardo	2
HMM0786	Urbana	54	Feminino	Pardo	2
HMM0785	Urbana	70	Feminino	Negro	5
HMM0670	Urbana	40	Masculino	Branco	4
HMM0671	Urbana	82	Masculino	Pardo	2
HMM0672	Urbana	82	Feminino	Branco	2
HMM0673	Urbana	56	Masculino	Negro	2
HMM0674	Urbana	73	Feminino	Pardo	2
HMM0675	Urbana	47	Feminino	Negro	4
HMM0676	Rural	68	Feminino	Branco	2
HMM0677	Rural	68	Masculino	Negro	2
HMM0678	Urbana	36	Feminino	Branco	7
HMM0784	Urbana	68	Feminino	Negro	3
HMM0783	Urbana	51	Feminino	Pardo	3
HMM0782	Urbana	53	Feminino	Negro	2
HMM0781	Urbana	58	Feminino	Branco	3
HMM0780	Urbana	17	Masculino	Pardo	3
HMM0779	Urbana	16	Feminino	Branco	3
HMM0778	Urbana	77	Feminino	Negro	1
HMM0777	Urbana	62	Feminino	Negro	2
HMM0776	Urbana	12	Masculino	Negro	2
HMM0775	Urbana	8	Masculino	Pardo	3
HMM0918	Urbana	36	Feminino	Pardo	3
HMM0917	Urbana	50	Feminino	Pardo	5
HMM0679	Urbana	68	Feminino	Negro	3
HMM0680	Urbana	54	Masculino	Negro	3
HMM0772	Urbana	49	Feminino	Pardo	1
HMM0771	Urbana	70	Masculino	Negro	2
HMM0770	Urbana	17	Masculino	Negro	5
HMM0769	Urbana	8	Feminino	Branco	4
HMM0766	Urbana	51	Feminino	Não sei	2
HMM0768	Urbana	60	Masculino	Pardo	3
HMM0767	Urbana	67	Feminino	Branco	2
HMM0765	Urbana	67	Feminino	Pardo	2
HMM0619	Urbana	54	Feminino	Pardo	1
HMM0620	Urbana	72	Masculino	Branco	2
HMM0621	Urbana	58	Masculino	Pardo	4

HMM0622	Rural	66	Masculino	Pardo		1
HMM0588	Urbana	66	Feminino	Branco		2
HMM0589	Rural	62	Masculino	Pardo		6
HMM0590	Urbana	76	Feminino	Negro		2
HMM0591	Rural	45	Feminino	Pardo		2
HMM0592	Urbana	75	Masculino	Pardo		3
HMM0593	Urbana	78	Feminino	Branco		4
HMM0594	Urbana	45	Masculino	Branco		4
HMM0596	Urbana	83	Masculino	Branco		1
HMM0595	Urbana	58	Feminino	Pardo		2
HMM0597	Urbana	72	Feminino	Pardo		1
HMM0598	Urbana	79	Masculino	Branco		1
HMM0599	Rural	19	Masculino	Negro		6
HMM0605	Rural	57	Masculino	Negro		3
HMM0606	Urbana	63	Feminino	Pardo		7
HMM0607	Urbana	54	Feminino	Pardo		5
HMM0608	Urbana	59	Feminino	Pardo		3
HMM0609	Rural	70	Feminino	Pardo		3
HMM0610	Urbana	65	Masculino	Pardo		3
HMM0611	Urbana	32	Masculino	Negro		1
HMM0612	Rural	51	Feminino	Negro		3
HMM0613	Urbana	86	Feminino	Branco		2
HMM0614	Urbana	69	Feminino	Pardo		3
HMM0615	Urbana	70	Feminino	Branco		2
HMM0616	Rural	54	Feminino	Pardo		3
HMM0617	Urbana	55	Masculino	Pardo		3
HMM0618	Rural	44	Masculino	Pardo		4
HMM0837	Urbana	18	Feminino	Branco		3
HMM0835	Urbana	28	Masculino	Pardo		4
HMM0836	Urbana	59	Feminino	Branco		3
HMM0839	Urbana	57	Feminino	Branco		2
HMM0840	Urbana	58	Masculino	Branco		2
HMM0841	Urbana	53	Feminino	Negro		4
HMM0842	Urbana	9	Feminino	Pardo		3
HMM0843	Urbana	57	Feminino	Pardo		4
HMM0844	Urbana	52	Feminino	Pardo		3
HMM0845	Urbana	53	Feminino	Outro	MORENA	3
HMM0846	Urbana	74	Feminino	Pardo		1
HMM0855	Urbana	67	Masculino	Recusou a responder		2
HMM0856	Urbana	60	Masculino	Pardo		1
HMM0857	Urbana	55	Masculino	Pardo		3
HMM0858	Urbana	8	Feminino	Negro		4
HMM0859	Urbana	28	Feminino	Negro		4

HMM0860	Urbana	62	Masculino	Branco	2
HMM0861	Urbana	10	Masculino	Pardo	4
HMM0862	Urbana	37	Feminino	Pardo	4
HMM0863	Urbana	37	Masculino	Pardo	4
HMM0864	Urbana	59	Masculino	Branco	2
HMM0865	Urbana	57	Feminino	Pardo	2
HMM0866	Urbana	61	Feminino	Negro	2
HMM0847	Urbana	52	Feminino	Negro	4
HMM0848	Urbana	57	Feminino	Branco	3
HMM0849	Urbana	54	Masculino	Pardo	4
HMM0850	Urbana	41	Feminino	Branco	4
HMM0851	Urbana	10	Masculino	Pardo	4
HMM0852	Urbana	17	Masculino	Negro	4
HMM0853	Urbana	33	Feminino	Pardo	6
HMM0854	Urbana	6	Masculino	Pardo	6
HMM0916	Urbana	53	Masculino	Pardo	3
HMM0914	Urbana	31	Feminino	Pardo	3
HMM0913	Urbana	35	Feminino	Pardo	4
HMM0912	Urbana	11	Feminino	Pardo	4
HMM0911	Urbana	6	Feminino	Pardo	4
HMM0915	Urbana	5	Feminino	Pardo	3
HMM0952	Urbana	24	Masculino	Branco	3
HMM0953	Rural	63	Feminino	Branco	2
HMM0954	Rural	64	Feminino	Pardo	3
HMM0955	Urbana	19	Feminino	Pardo	4
HMM0956	Rural	39	Masculino	Branco	3
HMM0957	Rural	61	Masculino	Branco	2
HMM0958	Rural	65	Feminino	Pardo	1
HMM0959	Rural	46	Feminino	Branco	3
HMM0960	Rural	16	Feminino	Pardo	3
HMM0961	Rural	69	Masculino	Pardo	3
HMM0962	Rural	57	Masculino	Branco	3
HMM0963	Rural	41	Feminino	Pardo	3
HMM0964	Rural	45	Feminino	Pardo	3
HMM0965	Rural	44	Feminino	Pardo	3
HMM0966	Rural	77	Masculino	Pardo	3
HMM0967	Rural	45	Masculino	Pardo	4
HMM0898	Urbana	7	Masculino	Pardo	2
HMM0899	Urbana	7	Masculino	Pardo	2
HMM0891	Urbana	39	Feminino	Pardo	2
HMM0947	Urbana	53	Feminino	Pardo	2
HMM0946	Urbana	55	Feminino	Pardo	4
HMM0968	Rural	32	Feminino	Pardo	4

HMM0813	Urbana	63	Feminino	Pardo	2
HMM0969	Rural	34	Feminino	Negro	5
HMM0970	Rural	17	Feminino	Pardo	5
HMM0814	Urbana	60	Feminino	Pardo	1
HMM0971	Rural	12	Feminino	Pardo	5
HMM0972	Rural	49	Feminino	Branco	2
HMM0816	Urbana	63	Masculino	Branco	2
HMM0973	Rural	66	Feminino	Pardo	1
HMM0974	Rural	59	Feminino	Negro	2
HMM0975	Rural	17	Masculino	Negro	2
HMM0976	Rural	44	Feminino	Pardo	2
HMM0977	Rural	19	Feminino	Branco	2
HMM0978	Rural	61	Masculino	Pardo	2
HMM0979	Rural	22	Feminino	Pardo	1
HMM0980	Rural	58	Feminino	Pardo	5
HMM0815	Urbana	49	Feminino	Pardo	2
HMM0981	Rural	32	Feminino	Pardo	5
HMM0982	Rural	60	Masculino	Pardo	5
HMM0817	Urbana	55	Masculino	Pardo	4
HMM0983	Rural	47	Feminino	Pardo	4
HMM0984	Rural	59	Feminino	Branco	3
HMM0818	Urbana	64	Feminino	Negro	4
HMM0819	Urbana	62	Feminino	Pardo	2
HMM0820	Urbana	44	Feminino	Negro	3
HMM0821	Urbana	55	Feminino	Branco	6
HMM0822	Urbana	54	Masculino	Negro	1
HMM0823	Urbana	81	Masculino	Negro	3
HMM0824	Urbana	56	Masculino	Pardo	2
HMM0825	Urbana	5	Feminino	Pardo	2
HMM0826	Urbana	60	Feminino	Pardo	1
HMM0827	Urbana	52	Feminino	Branco	2
HMM0828	Urbana	71	Masculino	Pardo	4
HMM0829	Urbana	8	Feminino	Negro	3
HMM0830	Urbana	39	Feminino	Pardo	3
HMM0831	Urbana	61	Feminino	Pardo	5
HMM0832	Urbana	59	Feminino	Pardo	2
HMM0833	Urbana	66	Feminino	Negro	1
HMM0834	Urbana	50	Masculino	Pardo	6
HMM0838	Urbana	10	Feminino	Pardo	4
HMM0873	Urbana	50	Masculino	Pardo	1
HMM0937	Urbana	61	Feminino	Pardo	3
HMM0936	Urbana	85	Masculino	Branco	1
HMM0935	Urbana	24	Feminino	Pardo	3

HMM0925	Urbana	57	Masculino	Branco	1
HMM0926	Urbana	65	Feminino	Não sei	2
HMM0934	Urbana	53	Feminino	Pardo	3
HMM0933	Urbana	18	Masculino	Pardo	3
HMM0932	Urbana	39	Feminino	Pardo	4
HMM0931	Urbana	16	Masculino	Pardo	4
HMM0930	Urbana	10	Masculino	Pardo	4
HMM0928	Urbana	7	Masculino	Pardo	2
HMM0929	Urbana	14	Masculino	Pardo	3
HMM0985A	Rural	71	Masculino	Pardo	2
HMM0985B	Rural	57	Masculino	Pardo	3
HMM0986	Rural	56	Masculino	Pardo	1
HMM0987	Rural	29	Feminino	Pardo	2
HMM0988	Rural	65	Feminino	Pardo	4
HMM0989	Rural	64	Feminino	Pardo	2
HMM0990	Rural	51	Masculino	Pardo	2
HMM0991	Rural	27	Masculino	Pardo	3
HMM0992	Rural	6	Masculino	Pardo	5
HMM0993	Rural	74	Masculino	Não sei	2
HMM0994	Rural	31	Masculino	Pardo	3
HMM0995	Rural	32	Masculino	Pardo	3
HMM0996	Rural	41	Feminino	Branco	4
HMM0997	Rural	57	Feminino	Negro	2
HMM0998	Rural	50	Feminino	Pardo	3
HMM0999	Rural	13	Feminino	Pardo	3
HMM1000	Rural	70	Feminino	Pardo	2
HMM1021	Rural	42	Masculino	Pardo	4
HMM1001	Rural	27	Feminino	Pardo	4
HMM1002	Rural	44	Feminino	Negro	2
HMM1003	Rural	48	Masculino	Branco	2
HMM1004	Rural	30	Masculino	Pardo	4
HMM1005	Rural	21	Masculino	Negro	4
HMM1006	Rural	56	Feminino	Pardo	2
HMM1007	Rural	68	Masculino	Pardo	2
HMM1008	Rural	53	Feminino	Pardo	4
HMM1009	Rural	16	Masculino	Pardo	5
HMM1010	Rural	49	Masculino	Negro	4
HMM1011	Rural	45	Masculino	Negro	2
HMM1012	Rural	19	Feminino	Negro	4
HMM1013	Rural	54	Masculino	Negro	4
HMM1014	Rural	39	Feminino	Negro	2
HMM1015	Rural	17	Feminino	Branco	4
HMM1016	Rural	38	Feminino	Pardo	5

HMM1017	Rural	38	Feminino	Negro	3
HMM1018	Rural	57	Masculino	Pardo	2
HMM1019	Rural	59	Feminino	Branco	2
HMM1020	Rural	33	Feminino	Pardo	5
HMM1022	Rural	73	Feminino	Pardo	1
HMM1023	Rural	89	Feminino	Pardo	2
HMM1024	Rural	35	Feminino	Pardo	2
HMM1025	Rural	65	Masculino	Pardo	2
HMM1026	Rural	60	Masculino	Pardo	2
HMM0874	Urbana	19	Feminino	Pardo	3
HMM0875	Urbana	7	Masculino	Pardo	3
HMM0876	Urbana	54	Feminino	Pardo	3
HMM1027	Rural	55	Feminino	Branco	2
HMM1028	Rural	63	Masculino	Pardo	2
HMM1029	Rural	67	Feminino	Pardo	1
HMM1030	Rural	32	Feminino	Pardo	4
HMM0872	Urbana	65	Masculino	Negro	1
HMM0869	Urbana	20	Feminino	Branco	4
HMM0868	Urbana	14	Feminino	Pardo	4
HMM1068	Urbana	46	Feminino	Branco	4
HMM0870	Urbana	47	Feminino	Pardo	4
HMM0871	Urbana	22	Feminino	Negro	4
HMM0877	Urbana	6	Feminino	Negro	4
HMM0878	Urbana	63	Feminino	Pardo	3
HMM0879	Urbana	45	Feminino	Negro	6
HMM0880	Urbana	15	Feminino	Negro	6
HMM0945	Urbana	36	Feminino	Pardo	3
HMM0881	Urbana	19	Masculino	Pardo	7
HMM0944	Urbana	11	Masculino	Pardo	4
HMM0943	Urbana	38	Feminino	Negro	4
HMM0942	Urbana	43	Feminino	Negro	5
HMM0941	Urbana	40	Masculino	Pardo	4
HMM0940	Urbana	9	Feminino	Negro	5
HMM0939	Urbana	11	Masculino	Negro	5
HMM0938	Urbana	15	Masculino	Negro	5
HMM0923	Urbana	80	Feminino	Pardo	4
HMM0924	Urbana	10	Feminino	Pardo	2
HMM0951	Urbana	51	Feminino	Pardo	3
HMM0950	Urbana	17	Feminino	Pardo	3
HMM0949	Urbana	19	Feminino	Pardo	3
HMM0922	Urbana	14	Feminino	Negro	3
HMM0920	Urbana	32	Masculino	Pardo	6
HMM0921	Urbana	37	Feminino	Negro	5

HMM0910	Urbana	67	Masculino	Pardo		2
HMM0909	Urbana	77	Feminino	Branco		2
HMM0908	Urbana	67	Feminino	Negro		5
HMM0907	Urbana	49	Feminino	Negro		4
HMM0906	Urbana	7	Feminino	Pardo		4
HMM0905	Urbana	13	Feminino	Pardo		5
HMM0904	Urbana	61	Feminino	Branco		5
HMM0902	Urbana	44	Feminino	Negro		4
HMM0903	Urbana	10	Feminino	Pardo		5
HMM0901	Urbana	12	Masculino	Negro		4
HMM0900	Urbana	10	Feminino	Negro		4
HMM0897	Urbana	40	Masculino	Negro		5
HMM0896	Urbana	59	Feminino	Negro		3
HMM0895	Urbana	49	Feminino	Pardo		2
HMM0894	Urbana	25	Masculino	Branco		2
HMM0893	Urbana	40	Masculino	Pardo		2
HMM0892	Urbana	63	Feminino	Pardo		2
HMM1031	Rural	22	Feminino	Pardo		6
HMM0890	Urbana	71	Feminino	Negro		1
HMM0889	Urbana	52	Feminino	Pardo		4
HMM0888	Urbana	8	Masculino	Branco		4
HMM1032	Rural	34	Feminino	Branco		2
HMM0887	Urbana	34	Feminino	Outro	Morena	3
HMM1033	Rural	67	Feminino	Branco		2
HMM0886	Urbana	50	Feminino	Pardo		4
HMM1034	Rural	71	Masculino	Branco		2
HMM0885	Urbana	68	Feminino	Indigeno		1
HMM0884	Urbana	67	Feminino	Pardo		1
HMM1035	Rural	78	Feminino	Branco		2
HMM0883	Urbana	47	Feminino	Pardo		5
HMM0882	Urbana	8	Feminino	Negro		5
HMM0623	Urbana	33	Feminino	Pardo		2
HMM0624	Urbana	58	Feminino	Pardo		2
HMM0625	Rural	51	Feminino	Pardo		2
HMM0626	Rural	45	Feminino	Negro		2
HMM0627	Rural	38	Feminino	Pardo		5
HMM0628	Rural	56	Feminino	Branco		2
HMM0631	Rural	42	Feminino	Pardo		4
HMM0629	Urbana	33	Feminino	Branco		2
HMM0632	Urbana	75	Feminino	Pardo		2
HMM0630	Urbana	56	Feminino	Pardo		2
HMM0633	Urbana	64	Feminino	Branco		3
HMM0635	Urbana	59	Masculino	Branco		2

HMM0634	Urbana	43	Feminino	Pardo	2
HMM0641	Urbana	82	Feminino	Negro	2
HMM0636	Urbana	65	Feminino	Pardo	4
HMM0642	Urbana	39	Feminino	Branco	7
HMM0637	Urbana	67	Feminino	Branco	2
HMM0643	Rural	46	Feminino	Pardo	5
HMM0638	Urbana	65	Feminino	Branco	2
HMM0644	Urbana	62	Feminino	Pardo	1
HMM0639	Urbana	66	Masculino	Branco	2
HMM0647	Urbana	63	Masculino	Pardo	3
HMM0645	Rural	39	Feminino	Pardo	4
HMM0646	Rural	77	Masculino	Branco	2
HMM0648	Rural	5	Feminino	Pardo	5
HMM0640	Urbana	47	Feminino	Pardo	3
HMM0651	Urbana	69	Feminino	Pardo	1
HMM0652	Urbana	82	Feminino	Pardo	1
HMM0649	Urbana	46	Feminino	Pardo	4
HMM0653	Urbana	29	Feminino	Pardo	5
HMM0650	Urbana	63	Feminino	Pardo	3
HMM0654	Urbana	69	Feminino	Pardo	3
HMM0655	Rural	50	Masculino	Branco	3
HMM0656	Rural	36	Masculino	Branco	2
HMM0657	Urbana	67	Feminino	Negro	4
HMM0658	Rural	15	Feminino	Pardo	5
HMM1036	Rural	59	Feminino	Pardo	1
HMM1037	Rural	13	Feminino	Pardo	3
HMM1038	Rural	14	Masculino	Pardo	5
HMM1039	Rural	69	Feminino	Pardo	3
HMM1040	Rural	20	Masculino	Branco	3
HMM1041	Rural	16	Feminino	Pardo	5
HMM1042	Rural	71	Feminino	Pardo	2
HMM1043	Rural	62	Feminino	Pardo	3
HMM1044	Rural	35	Masculino	Branco	5
HMM1045	Rural	13	Masculino	Pardo	5
HMM1046	Rural	12	Masculino	Branco	5
HMM1047	Urbana	68	Masculino	Branco	2
HMM1048	Rural	56	Masculino	Pardo	2
HMM1049	Rural	37	Feminino	Pardo	4
HMM1050	Rural	15	Feminino	Branco	5
HMM1051	Rural	18	Masculino	Pardo	4
HMM1052	Rural	11	Masculino	Pardo	4
HMM1053	Rural	51	Feminino	Negro	4
HMM1054	Rural	33	Masculino	Negro	2

HMM1055	Rural	38	Masculino	Negro		2
HMM1056	Rural	41	Masculino	Negro		4
HMM1057	Rural	60	Masculino	Branco		1
HMM1058	Rural	37	Feminino	Pardo		3
HMM1059	Rural	42	Masculino	Pardo		3
HMM1060	Rural	18	Feminino	Pardo		3
HMM1061	Rural	18	Masculino	Pardo		3
HMM1062	Rural	30	Feminino	Pardo		3
HMM1063	Rural	18	Masculino	Negro		3
HMM1064	Rural	43	Feminino	Pardo		3
HMM1065	Rural	19	Feminino	Pardo		3
HMM1066	Rural	18	Feminino	Pardo		3
HMM1067	Rural	18	Masculino	Pardo		3
HMM0693	Urbana	71	Masculino	Branco		5
HMM0694	Urbana	52	Feminino	Pardo		2
HMM0695	Urbana	13	Masculino	Negro		4
HMM0696	Urbana	64	Feminino	Branco		3
HMM0697	Urbana	60	Feminino	Pardo		2
HMM0698	Urbana	12	Masculino	Negro		5
HMM0699	Urbana	48	Masculino	Pardo		4
HMM0731	Urbana	49	Feminino	Negro		5
HMM0759	Urbana	7	Feminino	Pardo		3
HMM0732	Urbana	64	Feminino	Pardo		2
HMM0733	Urbana	60	Masculino	Pardo		3
HMM0734	Urbana	14	Masculino	Negro		4
HMM0735	Urbana	66	Feminino	Negro		1
HMM0736	Urbana	77	Feminino	Negro		5
HMM0739	Urbana	85	Masculino	Pardo		4
HMM0740	Urbana	89	Feminino	Não sei		1
HMM0757	Urbana	48	Feminino	Pardo		3
HMM0758	Urbana	62	Feminino	Pardo		1
HMM0756	Urbana	55	Feminino	Pardo		2
HMM0752	Urbana	42	Feminino	Outro	MORENA	2
HMM0750	Urbana	65	Feminino	Outro	MORENA	2
HMM0755	Urbana	26	Feminino	Pardo		3
HMM0749	Urbana	58	Feminino	Negro		2
HMM0746	Urbana	65	Feminino	Pardo		2
HMM0741	Urbana	57	Feminino	Pardo		5
HMM0742	Urbana	75	Feminino	Pardo		1
HMM0738	Urbana	17	Masculino	Negro		4
HMM0748	Urbana	21	Feminino	Pardo		1
HMM0753	Urbana	47	Feminino	Pardo		3
HMM0760	Urbana	65	Feminino	Pardo		2

HMM0761	Urbana	56	Feminino	Pardo	5
HMM0762	Urbana	46	Feminino	Branco	3
HMM0763	Urbana	66	Feminino	Pardo	2
HMM0764	Urbana	38	Feminino	Pardo	2
HMM0688	Urbana	47	Masculino	Negro	1
HMM0689	Urbana	45	Feminino	Negro	4
HMM0691	Urbana	63	Feminino	Branco	2
HMM0692	Urbana	50	Feminino	Pardo	1
HMM0690	Urbana	81	Feminino	Pardo	3
HMM1108	Urbana	50	Feminino	Negro	3
HMM1109	Urbana	60	Masculino	Pardo	2
HMM1110	Urbana	50	Feminino	Branco	4
HMM1111	Urbana	85	Feminino	Branco	4
HMM1112	Urbana	50	Feminino	Pardo	2
HMM1113	Urbana	37	Feminino	Pardo	3
HMM1114	Urbana	18	Feminino	Pardo	4
HMM1115	Urbana	63	Masculino	Pardo	3
HMM1116	Urbana	57	Feminino	Pardo	4
HMM1117	Urbana	17	Feminino	Pardo	4
HMM1140	Urbana	52	Feminino	Negro	2
HMM1118	Urbana	44	Feminino	Pardo	4
HMM1141	Urbana	65	Masculino	Pardo	1
HMM1119	Urbana	66	Masculino	Pardo	2
HMM1142	Urbana	57	Feminino	Negro	3
HMM1120	Urbana	69	Masculino	Branco	3
HMM1143	Urbana	73	Feminino	Pardo	2
HMM1121	Urbana	60	Feminino	Pardo	2
HMM1144	Urbana	34	Masculino	Pardo	4
HMM1145	Urbana	46	Feminino	Branco	3
HMM1146	Urbana	38	Feminino	Pardo	2
HMM1147	Urbana	69	Feminino	Pardo	2
HMM1148	Urbana	13	Masculino	Pardo	3
HMM1122	Urbana	31	Masculino	Negro	5
HMM1149	Urbana	66	Feminino	Branco	3
HMM1150	Urbana	50	Masculino	Branco	4
HMM1123	Urbana	28	Feminino	Negro	5
HMM1124	Urbana	56	Feminino	Pardo	3
HMM1125	Urbana	12	Masculino	Pardo	3
HMM1126	Urbana	10	Feminino	Pardo	4
HMM1127	Urbana	55	Feminino	Negro	3
HMM1128	Urbana	65	Feminino	Branco	5
HMM1129	Urbana	71	Feminino	Branco	2
HMM1130	Urbana	29	Feminino	Pardo	4

HMM1151	Urbana	41	Feminino	Pardo	NA	
HMM1131	Urbana	46	Feminino	Pardo		2
HMM1152	Urbana	14	Feminino	Pardo		4
HMM1153	Urbana	65	Masculino	Pardo		2
HMM1154	Urbana	49	Feminino	Pardo		2
HMM1155	Urbana	59	Masculino	Pardo		2
HMM1156	Urbana	51	Masculino	Pardo		4
HMM1157	Urbana	57	Masculino	Pardo		5
HMM1158	Urbana	53	Masculino	Negro		1
HMM1159	Urbana	55	Masculino	Pardo		2
HMM1160	Urbana	45	Masculino	Pardo		7
HMM1161	Urbana	23	Feminino	Branco		2
HMM1162	Urbana	29	Feminino	Negro		5
HMM1163	Urbana	12	Masculino	Negro		5
HMM1164	Urbana	8	Feminino	Negro		5
HMM1165	Urbana	47	Feminino	Pardo		4
HMM1166	Urbana	76	Masculino	Branco		1
HMM1167	Urbana	49	Masculino	Branco		2
HMM1168	Urbana	38	Feminino	Pardo		4
HMM1169	Urbana	13	Masculino	Pardo		2
HMM1086	Urbana	57	Feminino	Branco		1
HMM1170	Urbana	52	Masculino	Pardo		4
HMM1171	Urbana	61	Masculino	Negro		3
HMM1172	Urbana	51	Feminino	Pardo		2
HMM1173	Urbana	27	Feminino	Pardo		2
HMM1174	Urbana	55	Masculino	Negro		3
HMM1132	Urbana	58	Feminino	Negro		2
HMM1133	Urbana	44	Masculino	Branco		1
HMM1134	Urbana	25	Feminino	Pardo		3
HMM1175	Urbana	51	Masculino	Pardo		
HMM1135	Urbana	52	Feminino	Branco		2
HMM1136	Urbana	62	Masculino	Pardo		3
HMM1137	Urbana	15	Masculino	Pardo		4
HMM1138	Urbana	55	Masculino	Pardo		2
HMM1139	Urbana	56	Feminino	Pardo		4
HMM1176	Urbana	77	Feminino	Branco		1
HMM1177	Urbana	52	Masculino	Branco		3
HMM1191	Urbana	27	Masculino	Pardo		3
HMM1178	Urbana	52	Feminino	Negro		3
HMM1192	Urbana	9	Masculino	Negro		3
HMM1179	Urbana	21	Masculino	Branco		3
HMM1180	Urbana	18	Masculino	Negro		6
HMM1193	Urbana	32	Masculino	Negro		1

HMM1181	Urbana	49	Feminino	Pardo		4
HMM1182	Urbana	18	Masculino	Pardo		6
HMM1183	Urbana	22	Masculino	Branco		4
HMM1194	Urbana	44	Feminino	Pardo		6
HMM1185	Urbana	51	Feminino	Pardo		4
HMM1195	Urbana	7	Feminino	Pardo		2
HMM1186	Urbana	47	Feminino	Pardo		2
HMM1196	Urbana	29	Feminino	Pardo		2
HMM1084	Urbana	52	Feminino	Pardo		3
HMM1083	Urbana	58	Feminino	Branco		3
HMM1082	Urbana	62	Feminino	Pardo		2
HMM1081	Urbana	46	Feminino	Negro		2
HMM1080	Urbana	60	Feminino	Negro		2
HMM1079	Urbana	70	Masculino	Negro		3
HMM1077	Urbana	58	Masculino	Branco		4
HMM1085	Urbana	76	Masculino	Outro	Moreno	5
HMM1078	Urbana	23	Feminino	Branco		3
HMM1076	Urbana	38	Feminino	Pardo		4
HMM1075	Urbana	61	Masculino	Negro		4
HMM1074	Urbana	76	Feminino	Pardo		5
HMM1073	Urbana	53	Feminino	Negro		4
HMM1072	Urbana	9	Feminino	Pardo		6
HMM1071	Urbana	39	Feminino	Pardo		6
HMM1070	Urbana	49	Feminino	Negro		4
HMM1069	Urbana	27	Feminino	Branco		6
HMM1187	Urbana	10	Feminino	Branco		2
HMM1188	Urbana	65	Feminino	Branco		2
HMM1189	Urbana	60	Feminino	Pardo		3
HMM1190	Urbana	59	Masculino	Negro		4
HMM1197	Urbana	48	Masculino	Pardo		1
HMM1198	Urbana	21	Feminino	Negro		3
HMM1199	Urbana	25	Feminino	Negro		6
HMM1200	Urbana	54	Feminino	Pardo		5
HMM1201	Urbana	65	Feminino	Pardo		4
HMM1202	Urbana	55	Feminino	Negro		3
HMM1203	Urbana	59	Feminino	Pardo		2
HMM1204	Urbana	42	Feminino	Negro		2
HMM1205	Urbana	65	Feminino	Negro		2
HMM1206	Urbana	67	Masculino	Pardo		2
HMM1207	Urbana	65	Feminino	Negro		5
HMM1208	Urbana	59	Feminino	Branco		2
HMM1210	Urbana	47	Feminino	Negro		5
HMM1184	Urbana	59	Masculino	Negro		2

HMM1211	Urbana	73	Masculino	Pardo	
HMM1212	Urbana		Feminino	Pardo	3
HMM1213	Urbana	72	Masculino	Pardo	2
HMM1214	Urbana	29	Masculino	Pardo	3
HMM1215	Urbana	65	Masculino	Pardo	4
HMM1216	Urbana	46	Feminino	Pardo	2
HMM1218	Urbana	44	Feminino	Pardo	3
HMM1219	Urbana	41	Masculino	Negro	5
HMM1220	Urbana	16	Masculino	Pardo	4
HMM1221	Urbana	29	Feminino	Branco	2
HMM1222	Urbana	45	Masculino	Branco	1
HMM1223	Urbana	75	Masculino	Negro	3
HMM1224	Urbana	64	Feminino	Negro	5
HMM0898	Urbana	7	Masculino	Pardo	2
HMM0927	Urbana	42	Feminino	Pardo	2
HMM0737	Urbana	69	Masculino	Negro	2
HMM0754	Urbana	66	Masculino	Branco	2
HMM1225	Urbana	40	Feminino	Negro	4
HMM1226	Urbana	77	Masculino	Pardo	2
HMM1227	Urbana	65	Feminino	Pardo	5
HMM1228	Urbana	63	Masculino	Branco	3
HMM1229	Urbana	44	Masculino	Pardo	1
HMM1230	Urbana	65	Masculino	Negro	3
HMM1231	Urbana	89	Masculino	Pardo	6
HMM1232	Urbana	72	Feminino	Pardo	2
HMM1233	Urbana	72	Masculino	Não sei	1
HMM0773	Urbana	23	Feminino	Pardo	3
HMM0774	Urbana	20	Feminino	Negro	3
HMM1234	Urbana	50	Feminino	Negro	4
HMM1235	Urbana	8	Masculino	Pardo	7
HMM1236	Urbana	57	Masculino	Pardo	7
HMM1237	Urbana	75	Feminino	Pardo	3
HMM1238	Urbana	47	Feminino	Negro	6
HMM1239	Urbana	70	Feminino	Pardo	3
HMM1240	Urbana	58	Feminino	Pardo	3
HMM1241	Urbana	58	Masculino	Negro	1
HMM1242	Urbana	28	Feminino	Pardo	6
HMM1243	Urbana	54	Masculino	Pardo	1
HMM1245	Urbana	59	Masculino	Negro	3
HMM1244	Urbana	65	Feminino	Negro	3
HMM1246	Urbana	72	Feminino	Indigeno	1
HMM1247	Urbana	11	Feminino	Negro	4
HMM1248	Urbana	20	Feminino	Negro	2

HMM1249	Urbana	20	Feminino	Pardo	2
HMM1250	Urbana	48	Feminino	Branco	4
HMM1251	Urbana	52	Feminino	Pardo	3
HMM1252	Urbana	56	Feminino	Pardo	3
HMM1253	Urbana	72	Masculino	Pardo	3
HMM1254	Urbana	40	Feminino	Pardo	4
HMM1255	Urbana	45	Feminino	Pardo	5
HMM1256	Urbana	19	Masculino	Pardo	5
HMM1257	Urbana	64	Feminino	Indigeno	1
HMM1258	Urbana	50	Masculino	Negro	1
HMM1259	Urbana	60	Feminino	Branco	1
HMM1282	Urbana	28	Feminino	Pardo	4
HMM1283	Urbana	39	Feminino	Branco	6
HMM1284	Urbana	21	Feminino	Branco	3
HMM1285	Urbana	27	Masculino	Pardo	5
HMM1286	Urbana	54	Masculino	Negro	8
HMM1287	Urbana	14	Masculino	Negro	8
HMM1288	Urbana	39	Feminino	Pardo	4
HMM1289	Urbana	65	Feminino	Pardo	3
HMM1290	Urbana	81	Masculino	Negro	1
HMM1291	Urbana	66	Feminino	Pardo	3
HMM1297	Urbana	77	Feminino	Branco	2
HMM1260	Urbana	65	Feminino	Branco	2
HMM1261	Urbana	67	Feminino	Pardo	2
HMM1262	Urbana	66	Masculino	Negro	4
HMM1263	Urbana	54	Feminino	Pardo	1
HMM1264	Urbana	36	Masculino	Pardo	5
HMM1265	Urbana	83	Masculino	Branco	3
HMM1266	Urbana	17	Feminino	Pardo	3
HMM1267	Urbana	64	Feminino	Negro	1
HMM1268	Urbana	57	Masculino	Pardo	2
HMM1269	Urbana	58	Masculino	Negro	2
HMM1270	Urbana	53	Masculino	Pardo	3
HMM1271	Urbana	52	Feminino	Pardo	2
HMM1272	Urbana	53	Feminino	Pardo	4
HMM1273	Urbana	42	Feminino	Pardo	2
HMM1298	Urbana	42	Masculino	Negro	1
HMM1299	Urbana	56	Masculino	Pardo	7
HMM1300	Urbana	14	Feminino	Pardo	4
HMM1301	Urbana	79	Feminino	Pardo	1
HMM1302	Urbana	59	Feminino	Pardo	4
HMM1303	Urbana	23	Masculino	Negro	3
HMM1304	Urbana	43	Feminino	Negro	2

HMM1305	Urbana	65	Masculino	Pardo	1
HMM1306	Urbana	27	Feminino	Negro	3
HMM1274	Urbana	68	Masculino	Pardo	3
HMM1275	Urbana	65	Masculino	Branco	11
HMM1276	Urbana	35	Masculino	Pardo	1
HMM1277	Urbana	58	Masculino	Negro	2
HMM1279	Urbana	55	Feminino	Negro	2
HMM1278	Urbana	48	Masculino	Pardo	7
HMM1280	Urbana	70	Masculino	Pardo	2
HMM1281	Urbana	51	Masculino	Pardo	2
HMM1292	Urbana	47	Masculino	Pardo	5
HMM1293	Urbana	53	Masculino	Branco	1
HMM1294	Urbana	18	Masculino	Branco	1
HMM1295	Urbana	49	Feminino	Negro	4
HMM1296	Urbana	27	Masculino	Pardo	1
HMM1087	Urbana	64	Feminino	Pardo	2
HMM1088	Urbana	65	Feminino	Pardo	4
HMM1089	Urbana	45	Masculino	Negro	2
HMM1090	Urbana	74	Masculino	Branco	2
HMM1091	Urbana	70	Masculino	Negro	3
HMM1092	Urbana	64	Feminino	Branco	3
HMM1093	Urbana	71	Masculino	Branco	2
HMM1094	Rural	70	Feminino	Pardo	3
HMM1095	Urbana	67	Masculino	Negro	3
HMM1096	Urbana	49	Feminino	Pardo	2
HMM1099	Urbana	41	Feminino	Pardo	2
HMM1107	Urbana	74	Feminino	Branco	2
HMM1106	Urbana	63	Feminino	Negro	4
HMM1103	Urbana	24	Feminino	Branco	2
HMM1104	Urbana	46	Feminino	Pardo	3
HMM1105	Urbana	67	Feminino	Negro	4
HMM1102	Urbana	24	Feminino	Não sei	6
HMM1101	Urbana	38	Feminino	Pardo	4
HMM1100	Urbana	30	Feminino	Pardo	2
HMM1098	Urbana	63	Feminino	Negro	3
HMM1097	Urbana	91	Feminino	Pardo	3
HMM1307	Urbana	53	Masculino	Negro	4
HMM1308	Urbana	43	Masculino	Pardo	1
HMM1309	Urbana	65	Masculino	Negro	2
HMM0217	Urbana	78	Masculino	Recusou a responder	
HMM1310	Urbana	65	Masculino	Branco	1
HMM1311	Urbana	37	Feminino	Pardo	4
HMM0227	Urbana	77	Feminino	Recusou a responder	

HMM0228	Urbana	73	Feminino	Recusou a responder	2
HMM0229	Urbana	39	Feminino	Recusou a responder	
HMM0230	Urbana	18	Masculino	Recusou a responder	
HMM0231	Urbana	64	Feminino	Recusou a responder	
HMM0232	Urbana	58	Masculino	Recusou a responder	
HMM0233	Urbana	56	Masculino	Recusou a responder	
HMM0234	Urbana	66	Masculino	Recusou a responder	
HMM0239	Urbana	27	Feminino	Não sei	5
HMM0240	Urbana	8	Masculino	Não sei	5
HMM0241	Urbana	26	Feminino	Recusou a responder	5
HMM0242	Urbana	50	Masculino	Recusou a responder	5
HMM0243	Urbana	28	Masculino	Não sei	5
HMM0026	Urbana	60	Feminino	Pardo	
HMM0236	Urbana	46	Feminino	Recusou a responder	
HMM0077	Urbana	12	Masculino	Recusou a responder	
HMM0104	Urbana	40	Feminino	Branco	4
HMM0867	Urbana	21	Feminino	Pardo	
HMM1313	Urbana	21	Feminino	Recusou a responder	
HMM1314	Urbana	20	Feminino	Recusou a responder	
HMM1315	Urbana	65	Feminino	Recusou a responder	
HMM1317	Urbana	59	Masculino	Recusou a responder	
HMM1318	Urbana	59	Masculino	Recusou a responder	
HMM1319	Urbana	64	Feminino	Recusou a responder	
HMM1320	Urbana	22	Masculino	Recusou a responder	
HMM0686	Urbana	9	Masculino	Recusou a responder	
HMM0687	Urbana	39	Feminino	Recusou a responder	

Você está trabalhando a	Se sim, qual	Se você não	Outros: Other:	Se você não	Qual é o seu
Não		Other	PEDIU DEMISSÃO E NÃ	AJUDANTE D	< 1 salário mí
Sim, dia inteiro	REPOSITOR DE ARMAZÉM				3-5 salários r
Sim, meio-dia	DOMÉSTICA				1-3 salários r
Não		Retired		LAVADEIRA	< 1 salário mí
Não		Health reasons		DOMÉSTICA	< 1 salário mí
Sim, dia inteiro	TRABALHA COM ROÇADEIRA ELÉTRICA				1-3 salários r
Não		Other	MENOR DE IDADE	NÃO SE APLI	Não sei
Não		Other	MENOR DE IDADE	MENOR DE I	Não sei
Não		Other	MENOR DE IDADE	MENOR DE I	Não sei
Sim, dia inteiro	SERVIÇOS GERAIS (LIMPEZA EM LOJA DE AGROPECUÁRIA)				1-3 salários r
Não		Retired		MILITAR E OB	< 1 salário mí
Não		Taking care of children and/or househ		TRABALHOU	3-5 salários r
Não		Recently laid off		DOMÉSTICA	1-3 salários r
Não		Other	MENOR DE IDADE	MENOR DE I	< 1 salário mí
Não		Recently laid off		GARÇONETE	1-3 salários r
Não		Other	MENOR DE IDADE	MENOR DE I	< 1 salário mí
Não		Health reasons		TRABALHAVA	< 1 salário mí
Sim, dia inteiro	DOMÉSTICA				1-3 salários r
Sim, dia inteiro	PEDREIRO				1-3 salários r
Não		Retired		VAQUEIRO	1-3 salários r
Não		Retired		TRABALHAD	1-3 salários r
Sim, dia inteiro	TÉCNICA DE ENFERMAGEM				1-3 salários r
Não		Retired		PEDREIRO	1-3 salários r
Sim, dia inteiro	CUIDADORA DE IDOSOS				1-3 salários r
Não		Recently laid off		TRABALHAD	1-3 salários r
Não		Retired		DIARISTA/LIM	1-3 salários r
Não		Recently laid off		FAXINEIRA	< 1 salário mí
Não		Health reasons		COZINHEIRA	1-3 salários r
Não		Recently laid off		DOMÉSTICA	1-3 salários r
Não		Retired		MOTORISTA	1-3 salários r
Sim, dia inteiro	DOMÉSTICA				1-3 salários r
Sim, dia inteiro	PEDREIRO				1-3 salários r
Sim, dia inteiro	MOTORISTA				1-3 salários r
Sim, dia inteiro	PEDREIRO				1-3 salários r
Não		Other	MENOR DE IDADE	MENOR DE I	< 1 salário mí
Não		Taking care of children and/or househ		FAXINEIRA	1-3 salários r
Não		Retired		CONSTRUÇÃ	1-3 salários r
Sim, dia inteiro	CUIDADORA				1-3 salários r
Não		Retired		NÃO RESPON	1-3 salários r
Não		Other	PENSIONISTA	DOMÉSTICA	1-3 salários r
Sim, dia inteiro	DOMÉSTICA				< 1 salário mí
Sim, dia inteiro	COSTUREIRA				< 1 salário mí

Não		Health reasons		NÃO RESPON	1-3 salários r
Sim, meio-dia	TRABALHA EM PIZZARIA				< 1 salário mí
Não		Health reasons		DOMÉSTICA	1-3 salários r
Não		Taking care of children and/or househ		SECRETARIA	1-3 salários r
Não		Recently laid off		SEGURANÇA	< 1 salário mí
Não		Retired		VIGIA	1-3 salários r
Sim, meio-dia	DIARISTA				< 1 salário mí
Não		Retired		NÃO RESPON	1-3 salários r
Sim, dia inteiro	BABÁ				1-3 salários r
Não		Health reasons		LIMPEZA URB	1-3 salários r
Não		Taking care of children and/or househ		LIMPEZA URB	< 1 salário mí
Não		Other	MENOR DE IDADE	MENOR DE ID	1-3 salários r
Sim, dia inteiro	DIARISTA				1-3 salários r
Sim, dia inteiro	FAXINEIRA				< 1 salário mí
Não		Taking care of children and/or househ		FAXINA	< 1 salário mí
Não		Recently laid off		NÃO RESPON	1-3 salários r
Não		Health reasons		NÃO RESPON	1-3 salários r
Não		Temporarily at home		DOMÉSTICA	< 1 salário mí
Não		Retired		FUNCIONÁRI	1-3 salários r
Sim, dia inteiro	PEDREIRO				1-3 salários r
Sim, dia inteiro	DOMÉSTICA				1-3 salários r
Não		Retired		NAÕ RESPON	1-3 salários r
Sim, meio-dia	DIARISTA				< 1 salário mí
Sim, dia inteiro	DOMÉSTICA				1-3 salários r
Não		Retired		DOMÉSTICA	1-3 salários r
Não		Retired		LAVADEIRA /	< 1 salário mí
Sim, dia inteiro	OPERADOR DE MÁQUINA				< 1 salário mí
Não		Retired		NÃO RESPON	1-3 salários r
Não		Other	MENOR DE IDADE	MENOR DE ID	1-3 salários r
Não		Other	MENOR DE IDADE	MENOR DE ID	< 1 salário mí
Não		Retired		FAXINEIRA	1-3 salários r
Sim, dia inteiro	VENDEDORA AMBULANTE LIMPEZA				1-3 salários r
Sim, dia inteiro	VENDEDORA				< 1 salário mí
Sim, dia inteiro	DOMÉSTICA				1-3 salários r
Não		Recusou a responder		NÃO RESPON	1-3 salários r
Não		Taking care of children and/or househ		NUNCA TRAB	1-3 salários r
Não		Taking care of children and/or househ		DIARISTA	< 1 salário mí
Sim, dia inteiro	TRABALHA EM UM BAR E É PEDREIRO TAMBÉM				1-3 salários r
Sim, dia inteiro	ATENDENTE DE LOJA				1-3 salários r
Não		Taking care of children and/or househ		VENDEDORA	3-5 salários r
Não		Retired		DOMÉSTICA	1-3 salários r
Não		Retired		PEDREIRO	1-3 salários r
Não		Retired		COZINHEIRA	< 1 salário mí

Não		Recently laid off		NÃO TRABALI	< 1 salário mí
Não		Retired		DONA DE CA	1-3 salários r
Sim, dia inteiro	REPOSITOR DE MERCADORIAS				1-3 salários r
Não		Retired		TRABALHAVA	1-3 salários r
Não		Other	MENOR DE IDADE	MENOR DE II	< 1 salário mí
Sim, meio-dia	ORGANIZAÇÃO DE EVENTOS				1-3 salários r
Não		Retired		PEDREIRO	1-3 salários r
Não		Retired		CF	1-3 salários r
Não		Retired		MECÂNICO	1-3 salários r
Não		Health reasons		COSTUREIRA	1-3 salários r
Não		Retired		FUNCIONÁRI	1-3 salários r
Não		Retired		LAVADEIRA	1-3 salários r
Não		Health reasons		AJUDANTE DI	< 1 salário mí
Sim, dia inteiro	ASSESSORA DE VENDAS				1-3 salários r
Não		Temporarily at home		BABÁ	1-3 salários r
Não		Retired		AUXILIAR DE	1-3 salários r
Sim, dia inteiro	CABELEREIRO				1-3 salários r
Não		Retired		POLIDOR	1-3 salários r
Sim, meio-dia	TRABALHA EM EMPRESA DE LATICÍNIOS				1-3 salários r
Sim, dia inteiro	PEDREIRO				1-3 salários r
Sim, dia inteiro	DIARISTA				3-5 salários r
Não		Other	MENOR DE IDADE	MENOR DE II	1-3 salários r
Sim, dia inteiro	PEDREIRO				1-3 salários r
Não		Health reasons		OPERADOR D	1-3 salários r
Não		Retired		AUXILIAR DE	1-3 salários r
Não		Health reasons		COZINHEIRA	1-3 salários r
Não		Taking care of children and/or househ		DOMÉSTICA	1-3 salários r
Não		Retired		RECEPCIONI	1-3 salários r
Não		Retired		NÃO RESPON	1-3 salários r
Não		Retired		SEGURANÇA	1-3 salários r
Não		Health reasons		DOMÉSTICA	< 1 salário mí
Sim, meio-dia	VENDEDORA AUTÔNOMA				1-3 salários r
Não		Health reasons		DOMÉSTICA	1-3 salários r
Não		Health reasons		NÃO RESPOD	< 1 salário mí
Sim, dia inteiro	CAIXA DE POSTO DE GASOLINA				1-3 salários r
Sim, meio-dia	ASSISTENTE ADMINISTRATIVO				1-3 salários r
Não		Health reasons		comerciante	1-3 salários r
Não		Health reasons		Faxineira	1-3 salários r
Sim, dia inteiro	Doméstica				< 1 salário mí
Sim, dia inteiro	Cuidadora de Idosos				< 1 salário mí
Sim, dia inteiro	Técnico em fibra óptica				1-3 salários r
Não		Recently laid off		Serviços gera	1-3 salários r
Não		Retired		Auxiliar de se	1-3 salários r

Não		Retired			1-3 salários r
Não		Health reasons		Costureira	1-3 salários r
Não		Retired		Trabalhadora	1-3 salários r
Não		Health reasons		Vendedora	< 1 salário mí
Sim, dia inteiro	Doméstica				< 1 salário mí
Sim, dia inteiro	Gesseiro				3-5 salários r
Não		Health reasons		Doméstica	1-3 salários r
Não		Retired			1-3 salários r
Sim, dia inteiro	Pedreiro				1-3 salários r
Não		Recently laid off		Doméstica	1-3 salários r
Sim, dia inteiro	Cozinheira				< 1 salário mí
Não		Recently laid off		Cozinheira	1-3 salários r
Não		Other	cuidando da mãe	Doméstica, C	< 1 salário mí
Não		Health reasons		Doméstica	< 1 salário mí
Sim, dia inteiro	Diarista				1-3 salários r
Não		Other	pensionista	ajudante de p	1-3 salários r
Não		Other	Pensionista	Doméstica	1-3 salários r
Não		Health reasons		diarista	< 1 salário mí
Não		Health reasons			< 1 salário mí
Não		Retired		Cozinheira	1-3 salários r
Não		Retired		Atendente de	1-3 salários r
Não		Retired		Doméstica	1-3 salários r
Sim, dia inteiro	Marcineiro				3-5 salários n
Não		Recently laid off		gerente de hc	< 1 salário mí
Não		Recently laid off		Domestica	1-3 salários r
Não		Recently laid off		cuidador	< 1 salário mí
Não		Retired		caixa de pada	< 1 salário mí
Não		Retired		Padaria	< 1 salário mí
Não		Taking care of children and/or househ		Sempre foi dc	1-3 salários r
Não		Retired		lavadeira	1-3 salários n
Não		Recently laid off		Motorista de	< 1 salário mí
Não		Taking care of children and/or househ		Doméstica	< 1 salário mí
Não		Retired		Funcionária I	1-3 salários r
Não		Recently laid off		Doméstica	1-3 salários r
Não		Recently laid off		Não teve	1-3 salários r
Não		Retired		asfalto	3-5 salários r
Não		Health reasons		Doméstica	< 1 salário mí
Sim, meio-dia	caminhoneiro				>5 salários m
Não		Health reasons		Vendedora	< 1 salário mí
Não		Health reasons		Doméstica	< 1 salário mí
Não		Health reasons		Trabalha na r	1-3 salários r
Não		Retired		Doméstica	1-3 salários r
Não		Recently laid off		Cuidadora de	< 1 salário mí

Não		Taking care of children and/or househ		Telemarkting	< 1 salário mí
Não		Retired		Pedreiro	< 1 salário mí
Não		Taking care of children and/or househ		Não trabalho	1-3 salários r
Não		Retired		Babá	< 1 salário mí
Sim, dia inteiro	Ajudante de Pedreiro				1-3 salários r
Sim, dia inteiro	Agente Comunitário de Saúde.				< 1 salário mí
Não		Taking care of children and/or househ		Nunca trabal	1-3 salários r
Não		Recently laid off		Caixa de sup	< 1 salário mí
Não		Recently laid off		Doméstica	1-3 salários r
Sim, meio-dia	Doméstica				< 1 salário mí
Não		Retired		Atendente de	1-3 salários r
Não		Retired		Chapa	< 1 salário mí
Sim, dia inteiro	Trabalhador rural				1-3 salários r
Sim, dia inteiro	Lavrador				1-3 salários r
Não		Taking care of children and/or househ		Empregada d	1-3 salários r
Não		Taking care of children and/or househ		COZINHEIRA	< 1 salário mí
Não		Health reasons		TRABALHOU	< 1 salário mí
Sim, meio-dia	FAXINA				< 1 salário mí
Não		Taking care of children and/or household			1-3 salários r
Sim, dia inteiro	TRABALHA EM OBRAS				1-3 salários r
Não		Retired		DIARISTA	< 1 salário mí
Não		Retired		TRABALHOU	3-5 salários r
Não		Other	MENOR DE IDADE	MENOR DE I[	Não sei
Não		Recently laid off		CUIDADOR D	1-3 salários r
Não		Other	MENOR DE IDADE	MENOR DE I[	< 1 salário mí
Sim, dia inteiro	CARROCEIRO				1-3 salários r
Sim, dia inteiro	Colhedora de café				< 1 salário mí
Não		Retired		Marceneiro	3-5 salários r
Sim, dia inteiro	Lavrador				< 1 salário mí
Sim, dia inteiro	LAVOURA DE CAFÉ				< 1 salário mí
Sim, dia inteiro	EMPRESÁRIO				1-3 salários r
Não		Retired		AUXILIAR	1-3 salários r
Não		Retired		LAVRADOR	1-3 salários r
Sim, dia inteiro	LAVRADOR				1-3 salários r
Não		Retired		NÃO RESPON	< 1 salário mí
Sim, dia inteiro	ORIENTADORA SOCIAL				1-3 salários r
Não		Retired		LAVRADORA	1-3 salários r
Não		Recently laid off		FAXINEIRA	< 1 salário mí
Não		Retired		LAVRADOR	1-3 salários r
Não		Other	MENOR DE IDADE	MENOR DE I[	1-3 salários r
Não		Retired		SERVENTE DI	1-3 salários r
Não		Other	MENOR DE IDADE	MENOR DE I[	Não sei
Sim, dia inteiro	LAVRADORA				1-3 salários n

Não		Retired		NÃO RESPON	< 1 salário mí
Sim, dia inteiro	VIGIA GARI (PREFEITURA)				1-3 salários r
Não		Other	MENOR DE IDADE	MENOR DE II	< 1 salário mí
Sim, dia inteiro	FOTÓGRAFO	CORRETOR DE IMÓVEL			3-5 salários r
Não		Retired		Faxineira	1-3 salários r
Não		Health reasons		COSTUREIRA	1-3 salários r
Não		Taking care of children and/or househ		Trabalhou em	< 1 salário mí
Não		Retired		DONA DE CA	1-3 salários r
Sim, dia inteiro	CASEIRO				1-3 salários r
Não		Temporarily at home		APANHADOR	1-3 salários r
Sim, dia inteiro	LAVADEIRA				Não sei
Não		Retired		DIARISTA	1-3 salários r
Não		Recently laid off		DIARISTA	1-3 salários r
Não		Retired		EMPREGADA	3-5 salários r
Não		Taking care of children and/or househ		DOMÉSTICA	1-3 salários r
Não		Other	MENOR DE IDADE	MENOR DE II	< 1 salário mí
Sim, dia inteiro	LAVOURA				< 1 salário mí
Não		Health reasons		LAVADEIRA	< 1 salário mí
Não		Temporarily at home		LAVOURA DE	< 1 salário mí
Não		Retired		NAO HA	1-3 salários r
Não		Retired		Lavradora	1-3 salários r
Não		Taking care of children and/or househ		SEMPRE FOI I	< 1 salário mí
Não		Health reasons		COZINHEIRA	< 1 salário mí
Não		Taking care of children and/or househ		SEMPRE FOI I	3-5 salários r
Não		Retired		NÃO RESPON	1-3 salários r
Não		Health reasons		SEMPRE FOI I	1-3 salários r
Sim, dia inteiro	PEDREIRO				< 1 salário mí
Não		Other	MENOR DE IDADE	MENOR DE II	< 1 salário mí
Não		Recently laid off		FAXINEIRA	1-3 salários r
Não		Retired		MANUTENÇÃ	1-3 salários r
Não		Retired		SERVIÇOS GI	1-3 salários r
Sim, dia inteiro	PEDREIRO				1-3 salários r
Não		Taking care of children and/or househ		DOMÉSTICA	< 1 salário mí
Não		Other	MENOR DE IDADE	MENOR DE II	Não sei
Não		Other	MENOR DE IDADE	MENOR DE II	< 1 salário mí
Não		Retired		DONA DE CA	< 1 salário mí
Não		Retired		CARPINTEIR(	< 1 salário mí
Não		Temporarily at home		SACOLEIRA ('	1-3 salários r
Não		Other	MENOR DE IDADE	MENOR DE II	1-3 salários r
Não		Temporarily at home		NÃO RESPON	1-3 salários r
Sim, dia inteiro	OBRA CIVIL				1-3 salários r
Não		Health reasons		MECÂNICO	1-3 salários r
Sim, meio-dia	BABÁ				1-3 salários r

Não		Temporarily at home		DOMÉSTICA	1-3 salários r
Sim, dia inteiro	OPERADOR DE MÁQUINA				< 1 salário mí
Não		Health reasons		TRABALHAD(	1-3 salários r
Sim, meio-dia	TRABALHA EM LOJA DE AÇAI				1-3 salários r
Não		Taking care of children and/or househ		MANICURE	1-3 salários r
Não		Retired		NÃO RESPON	1-3 salários r
Não		Health reasons		DIARISTA / CU	< 1 salário mí
Não		Recently laid off		AUXILIAR DE :	1-3 salários r
Não		Recently laid off		VENDEDOR	< 1 salário mí
Não		Retired		LAVADEIRA	< 1 salário mí
Não		Recently laid off		SERVENTE DI	< 1 salário mí
Não		Health reasons		AUXILIAR DE (	1-3 salários r
Não		Taking care of children and/or househ		SEMPRE FOI I	1-3 salários r
Não		Other	PENSIONISTA	COSTUREIRA	< 1 salário mí
Não		Other	Devido à pandemia	NAO HA	1-3 salários r
Não		Other	CRIANÇA	NAO HA	1-3 salários r
Não		Recently laid off		DONA DE CA	< 1 salário mí
Sim, meio-dia	LAVRADOR				< 1 salário mí
Não		Retired		NAO HA	1-3 salários r
Sim, dia inteiro	PEDREIRO				1-3 salários r
Não		Retired		NAO HA	1-3 salários r
Sim, dia inteiro	LAVRADORA				1-3 salários r
Sim, dia inteiro	LAVRADOR				1-3 salários r
Não		Retired		LAVRADOR	1-3 salários r
Não		Retired		NAO HA	1-3 salários r
Não		Recusou a responder		LAVRADOR	< 1 salário mí
Sim, dia inteiro	LAVRADOR				1-3 salários r
Não		Não sei		MANICURE	< 1 salário mí
Não		Retired		LAVRADORA	1-3 salários r
Não		Retired			1-3 salários r
Sim, dia inteiro	MARCENEIRO				1-3 salários r
Não		Retired		NAO CONSTA	1-3 salários r
Sim, dia inteiro	SECRETARIA				1-3 salários r
Sim, dia inteiro	FAXINEIRA				1-3 salários r
Não		Other	ESTUDANTE	ESTUDANTE	Não sei
Sim, dia inteiro	DOMESTICA				< 1 salário mí
Sim, meio-dia	AUXILIAR DE SERVIÇOS GERAIS				1-3 salários r
Não		Other	Cuida da mãe	Trabalhava co	1-3 salários r
Não		Health reasons		LAVRADOR	< 1 salário mí
Sim, meio-dia	DIARISTA				1-3 salários r
Sim, dia inteiro	LAVRADOR				1-3 salários r
Sim, dia inteiro	LAVRADOR				< 1 salário mí
Não		Taking care of children and/or househ		LAVRADORA	< 1 salário mí

Não		Retired		LAVRADORA	< 1 salário mí
Não		Retired		LAVOURA, AU	< 1 salário mí
Não		Recently laid off		PADARIA, VEI	Recusou a re:
Não		Retired		LAVRADORA	1-3 salários r
Não		Other	NÃO SE APLICA (CRIAN(	NÃO SE APLI(	1-3 salários r
Não		Retired		DONA DE CA	1-3 salários r
Sim, dia inteiro	PASTOR				1-3 salários r
Não		Health reasons		TRABALHOU I	< 1 salário mí
Não		Recently laid off		NÃO RESPON	< 1 salário mí
Sim, dia inteiro	PROFESSORA				1-3 salários r
Não		Retired		NAO ESPECIF	1-3 salários r
Sim, dia inteiro	LAVRADOR				1-3 salários r
Sim, dia inteiro	LAVRADOR				1-3 salários r
Não		Recusou a responder		DO LAR	3-5 salários r
Não		Retired		LAVRADOR	1-3 salários r
Não		Retired		LAVRADOR	1-3 salários r
Não		Não sei		LAVRADOR	1-3 salários r
Não		Retired		LAVRADOR	< 1 salário mí
Sim, dia inteiro	LAVRADOR				< 1 salário mí
Não		Recently laid off		NÃO ESPECIF	< 1 salário mí
Sim, dia inteiro	CUIDADORA DE IDOSO				1-3 salários r
Não		Retired		EMPREGADA	1-3 salários r
Não		Taking care of children and/or househ		NÃO HÁ	>5 salários m
Não		Health reasons		NÃO INFORM	< 1 salário mí
Não		Retired		LANCHONET	< 1 salário mí
Não		Other	PENSIONISTA	TRABALHAD(	1-3 salários r
Sim, meio-dia	Bar				1-3 salários r
Não		Temporarily at home		Trabalho na r	< 1 salário mí
Sim, dia inteiro	Lavrador				1-3 salários r
Sim, dia inteiro	DOMÉSTICA				< 1 salário mí
Sim, dia inteiro	LAVRADORA				< 1 salário mí
Não		Retired		DONA DE CA	1-3 salários r
Não		Retired		ENFERMAGE	1-3 salários r
Não		Health reasons		COSTUREIRA	< 1 salário mí
Não		Retired		NÃO HPA	3-5 salários r
Não		Temporarily at home		VENDEDORA	1-3 salários r
Sim, dia inteiro	LAVRADORA				< 1 salário mí
Não		Taking care of children and/or househ		CHAPELEIRA	< 1 salário mí
Não		Health reasons		VENDEDOR A	< 1 salário mí
Não		Recently laid off		FAXINA E CO:	1-3 salários r
Não		Recently laid off		NÃO TEM	1-3 salários r
Não		Taking care of children and/or househ		DOMÉSTICA	1-3 salários r
Não		Health reasons		MONTADOR I	< 1 salário mí

Não		Retired		Serviços gera	< 1 salário mí
Não		Health reasons		ZELADORA (F	1-3 salários r
Não		Retired		Professora	1-3 salários r
Não		Retired		MOTORISTA	1-3 salários r
Não		Retired		Lavoura	1-3 salários r
Não		Health reasons		PINTOR	< 1 salário mí
Não		Retired		SERVIÇOS GI	< 1 salário mí
Não		Other	Menor de Idade	Menor de Ida	Recusou a res
Sim, meio-dia	Fazendo Bicos				< 1 salário mí
Sim, dia inteiro	Trabalha na Roça - Roçadeiro				< 1 salário mí
Não		Health reasons		CHEFE DE DI	1-3 salários r
Não		Taking care of children and/or househ		Trabalhou na	1-3 salários r
Sim, dia inteiro	Soldador				1-3 salários r
Não		Retired		MOTORISTA	1-3 salários r
Não		Recently laid off		VIGIA NOTUR	< 1 salário mí
Sim, meio-dia	LAVRADOR				1-3 salários r
Não		Health reasons		DOMÉSTICA	< 1 salário mí
Não		Health reasons		LAVRADOR	< 1 salário mí
Não		Taking care of children and/or househ		VENDEDORA	< 1 salário mí
Não		Retired		LAVRADOR	< 1 salário mí
Sim, dia inteiro	LAVRADOR				< 1 salário mí
Não		Retired		LAVRADOR	1-3 salários n
Não		Retired		LAVRADOR	< 1 salário mí
Sim, dia inteiro	LAVRADOR				1-3 salários r
Sim, meio-dia	PEDREIRO				1-3 salários r
Sim, dia inteiro	PROFESSORA				3-5 salários r
Não		Health reasons		PEDREIRO	< 1 salário mí
Sim, meio-dia	PROFESSORA				1-3 salários r
Sim, dia inteiro	LAVRADOR				1-3 salários r
Sim, dia inteiro	ESCOLA				< 1 salário mí
Sim, meio-dia	ASB - ESTABULO				< 1 salário mí
Não		Other	MENOR DE IDADE	MENOR DE II	Não sei
Não		Recently laid off		OPERADORA	1-3 salários r
Sim, dia inteiro	FAXINEIRA				1-3 salários r
Não		Recently laid off		COSTUREIRA	< 1 salário mí
Não		Taking care of children and/or househ		NÃO RESPON	< 1 salário mí
Não		Other	PENSIONISTA	DOMÉSTICA	1-3 salários r
Não		Other	MENOR DE IDADE	MENOR DE II	1-3 salários r
Não		Health reasons		DOMÉSTICA	< 1 salário mí
Não		Taking care of children and/or househ		COSTUREIRA	< 1 salário mí
Não		Recently laid off		VENDEDORA	1-3 salários r
Sim, dia inteiro	COMERCIANTE				1-3 salários r
Sim, dia inteiro	PEDREIRO				1-3 salários r

Não		Other	PENSIONISTA	LAVADEIRA E	1-3 salários r
Sim, dia inteiro	SALGADEIRA	BABÁ			< 1 salário mí
Não		Other	MENOR DE IDADE	MENOR DE II	< 1 salário mí
Sim, meio-dia	SACOLEIRA (VENDEDORA AUTÔNOMA)				1-3 salários r
Não		Retired		Vigia	1-3 salários r
Não		Retired		Trabalhador r	< 1 salário mí
Não		Retired		Lavoura de ca	< 1 salário mí
Não		Retired		Zona rural	< 1 salário mí
Sim, meio-dia	Bazar, CLAS (artesanato)				1-3 salários r
Não		Retired		Lavoura (café	1-3 salários r
Não		Retired		Lavrador	1-3 salários n
Sim, dia inteiro	Comerciante				1-3 salários r
Não		Retired			1-3 salários r
Não		Health reasons		EM ROÇA	1-3 salários r
Não				Caixa em lan	< 1 salário mí
Não		Retired		LAVRADOR	1-3 salários r
Sim, meio-dia	LAVRADORA				< 1 salário mí
Não		Taking care of children and/or househ		DIARISTA	< 1 salário mí
Sim, dia inteiro	AUXILIAR DE SERVIÇOS GERAIS				1-3 salários r
Não		Health reasons		AJUDANTE DI	1-3 salários r
Sim, dia inteiro	Atendente				1-3 salários r
Não		Retired		Costureira	1-3 salários r
Sim, dia inteiro	Empresário				3-5 salários r
Sim, dia inteiro	Diarista				< 1 salário mí
Não		Retired		Pedreiro	< 1 salário mí
Não		Health reasons		Doméstica	1-3 salários r
Não		Other	Menor	Não se aplica	1-3 salários r
Não		Retired		Dona de Casa	1-3 salários r
Não		Other	Menor	Não se aplica	1-3 salários r
Sim, meio-dia	Lavoura				< 1 salário mí
Não		Retired		Trabalhava na	1-3 salários r
Não		Recently laid off		Diarista	1-3 salários r
Sim, dia inteiro	Comércio				< 1 salário mí
Sim, dia inteiro	Comerciante, Mercearia				1-3 salários r
Sim, dia inteiro	Área da saúde (Agente). Já foi trabalhador rural.				1-3 salários r
Não		Retired		Lavoura	1-3 salários r
Não		Temporarily at home		Cuidadora	Recusou a res
Sim, dia inteiro	Doméstica				1-3 salários r
Não		Retired		Faxina	1-3 salários r
Não		Retired		Lavadeira	1-3 salários r
Não		Other	Menor de Idade	Menor de Ida	Recusou a res
Não		Other	Menor de Idade	Menor de Ida	Recusou a res
Não		Health reasons		Pedreiro	< 1 salário mí

Não		Retired		NÃO INFORM	1-3 salários n
Não		Retired		Lavanderia	
Não		Other	Cuidando da mãe	Recepcionist	< 1 salário mí
Não		Recently laid off		Monitora esc	3-5 salários r
Sim, dia inteiro	Trabalha no bar				< 1 salário mí
Não		Other	Afastado dor no braço	Soldador	1-3 salários r
Sim, dia inteiro	Comercio, Pedreiro				< 1 salário mí
Sim, dia inteiro	Comércio				< 1 salário mí
Não		Recently laid off		Comercio, co	< 1 salário mí
Não		Recently laid off		Serviços gera	< 1 salário mí
Não		Recently laid off		Transportado	< 1 salário mí
Não		Retired		NÃO INFORM	< 1 salário mí
Não		Taking care of children and/or househ		NÃO INFORMADO	
Não		Other	MENOR DE IDADE	MENOR DE II	1-3 salários r
Não		Health reasons		DOMÉSTICA	1-3 salários r
Não		Retired		NÃO RESPON	>5 salários m
Não		Retired		Ajudante de p	1-3 salários r
Não		Retired		COSTUREIRA	1-3 salários r
Não		Other	CUIDANDO DA MÃE DO	MOTO-TÁXI /	1-3 salários r
Não		Health reasons		COSTUREIRA	< 1 salário mí
Não		Recently laid off		Frentista	< 1 salário mí
Sim, dia inteiro	SOLDADOR				1-3 salários r
Sim, dia inteiro	Motorista de caminhão.				1-3 salários r
Não		Taking care of children and/or househ		Doméstica	1-3 salários r
Não		Retired		SERVIÇOS GI	1-3 salários r
Não		Health reasons		Auxiliar de co	< 1 salário mí
Não		Taking care of children and/or househ		Caixa do Mc	< 1 salário mí
Não		Recently laid off		DOMÉSTICA	1-3 salários r
Sim, dia inteiro	COSTUREIRA				>5 salários m
Não		Other	CUIDA DOS PAIS	COZINHEIRA	1-3 salários r
Não		Health reasons		ROCEIRA	< 1 salário mí
Não		Health reasons		ROCEIRO	< 1 salário mí
Não		Retired		ATIVIDADES I	1-3 salários r
Não		Recently laid off		NÃO TRABALI	< 1 salário mí
Não		Recusou a responder		COZINHEIRA	< 1 salário mí
Não		Other	AGUARDANDO A APOSE	DONA DO LAI	< 1 salário mí
Não		Health reasons		Comércio	1-3 salários r
Não		Recently laid off		Não teve.	1-3 salários r
Sim, dia inteiro	Auxiliar de Serviços Gerais				1-3 salários r
Sim, dia inteiro	Motorista				1-3 salários r
Não		Taking care of children and/or househ		Fábrica de co	1-3 salários r
Sim, dia inteiro	Roça				1-3 salários r
Não		Retired		NÃO INFORM	< 1 salário mí

Sim, dia inteiro	Faxina / limpeza de casa				1-3 salários r
Sim, dia inteiro	SERVENTE DE PEDREIRO				< 1 salário mí
Não		Recusou a responder		PINTOR	< 1 salário mí
Não		Taking care of children and/or househ		NÃO TEVE PR	< 1 salário mí
Sim, meio-dia	ATENDENTE				< 1 salário mí
Não		Retired		NÃO RESPON	< 1 salário mí
Não		Health reasons		DOMÉSTICA	1-3 salários r
Não		Health reasons		SECRETÁRIA	< 1 salário mí
Não		Retired		OPERADOR D	1-3 salários r
Não		Health reasons		VENDEDORA	< 1 salário mí
Sim, dia inteiro	EXAMINADOR				1-3 salários r
Não		Recently laid off		VENDAS	< 1 salário mí
Sim, dia inteiro	BABÁ				< 1 salário mí
Não		Taking care of children and/or househ		BABÁ	< 1 salário mí
Não		Retired		FRIGORÍFICO	1-3 salários r
Sim, dia inteiro	DOMÉSTICA				< 1 salário mí
Sim, dia inteiro	LAVANDERIA				1-3 salários r
Sim, dia inteiro	COSTUREIRA				1-3 salários r
Não		Retired		TRABALHAVA	< 1 salário mí
Sim, meio-dia	JOVEM APRENDIZ				1-3 salários r
Não		Recently laid off		NÃO RESPON	< 1 salário mí
Sim, dia inteiro	CABELEREIRA				< 1 salário mí
Sim, dia inteiro	PEDREIRO				1-3 salários r
Não		Health reasons			1-3 salários r
Sim, dia inteiro	Babá				< 1 salário mí
Não		Taking care of children and/or househ		Oficina de ca	1-3 salários r
Sim, dia inteiro	Comércio				< 1 salário mí
Não		Health reasons			< 1 salário mí
Não		Health reasons		Faxina	< 1 salário mí
Não		Retired		Montador Inc	1-3 salários r
Não		Taking care of children and/or househ		Doméstica	1-3 salários r
Não		Retired		Gerente de E	1-3 salários r
Não		Temporarily at home		Atendente de	1-3 salários r
Não		Health reasons		Diarista	1-3 salários r
Não		Other	criança	Criança	1-3 salários r
Sim, dia inteiro	Vendas				1-3 salários r
Não		Retired		Comerciante	1-3 salários r
Não		Other	PENSIONISTA	CUIDADOS G	1-3 salários r
Não		Temporarily at home		DOMÉSTICA	< 1 salário mí
Não		Taking care of children and/or househ		DIARISTA (FA	< 1 salário mí
Não		Other	PENSIONISTA	EMPREGADA	< 1 salário mí
Não		Health reasons		DOMÉSTICA /	Não sei
Não		Health reasons		SALGADEIRA	< 1 salário mí

Não		Retired		LIMPEZA URE	< 1 salário mí
Sim, dia inteiro	ENCARREGADO				1-3 salários r
Sim, meio-dia	COSTUREIRA				< 1 salário mí
Não		Temporarily at home		CARTÓRIO	1-3 salários r
Não		Health reasons		AUTÔNOMA	1-3 salários n
Não		Retired		PEDREIRO	1-3 salários r
Não		Other	DEVIDO À PANDEMIA	VENDEDOR /	< 1 salário mí
Não		Health reasons		AUXILIAR GEI	< 1 salário mí
Não		Other	ESTUDANTE	NÃO TRABALI	< 1 salário mí
Não		Recently laid off		DIARISTA	< 1 salário mí
Não		Retired		FUNCIONÁRI	1-3 salários r
Não		Taking care of children and/or househ		MANICURE	< 1 salário mí
Não		Health reasons		DIARISTA	< 1 salário mí
Não		Other	MENOR DE IDADE	MENOR DE II	< 1 salário mí
Não		Other	MENOR DE IDADE	MENOR DE II	< 1 salário mí
Não		Taking care of children and/or househ		NÃO RESPON	< 1 salário mí
Não		Other	MENOR DE IDADE	MENOR DE II	< 1 salário mí
Não		Recently laid off		NUNCA TRAE	< 1 salário mí
Não		Health reasons		DIARISTA	< 1 salário mí
Sim, dia inteiro	COZINHEIRA				< 1 salário mí
Sim, dia inteiro	COMERCIANTE				1-3 salários r
Não		Taking care of children and/or househ		DOMÉSTICA	1-3 salários r
Sim, dia inteiro	POLÍCIA PENAL				3-5 salários r
Não		Health reasons		SERVENTE DI	< 1 salário mí
Não		Retired		AGRICULTUR	1-3 salários r
Não		Other	NÃO TRABALHOU FORA	NÃO TRABALI	1-3 salários r
Não		Retired		DOMESTICA	1-3 salários r
Não		Recently laid off		BALCONISTA	< 1 salário mí
Não		Retired		NÃO RESPON	1-3 salários r
Não		Health reasons		DOMÉSTICA	< 1 salário mí
Sim, meio-dia	ESTAGIÁRIO				3-5 salários r
Não		Other	MENOR DE IDADE	MENOR DE II	< 1 salário mí
Não		Recently laid off		COZINHEIRA	< 1 salário mí
Não		Other	MENOR DE IDADE - EST	MENOR DE II	< 1 salário mí
Sim, dia inteiro	ARTESÃ				< 1 salário mí
Não		Other	MENOR DE IDADDE	MENOR DE II	Não sei
Sim, dia inteiro	BABY SITTER				< 1 salário mí
Não		Recently laid off		Encarregado	1-3 salários r
Não		Taking care of children and/or househ		promotora de	1-3 salários r
Não		Recently laid off		Doméstica	< 1 salário mí
Sim, dia inteiro	auxiliar de limpeza				1-3 salários r
Sim, dia inteiro	agricultora				1-3 salários n
Sim, dia inteiro	Auxiliar De Limpeza.				1-3 salários r

Sim, dia inteiro	auxiliar de limpeza				1-3 salários r
Sim, dia inteiro	AUXILIAR - SERVIDOR PÚBLICO				1-3 salários r
Não		Other	MENOR DE IDADE	MENOR DE II	>5 salários m
Sim, dia inteiro	MECÂNICO				1-3 salários r
Não		Taking care of children and/or househ		EMPREGADA	1-3 salários r
Sim, dia inteiro	babá				1-3 salários r
Sim, dia inteiro	Auxiliar de limpeza				1-3 salários r
Não		Health reasons		Pintor	< 1 salário mí
Sim, dia inteiro	Serviços Gerais				1-3 salários r
Sim, dia inteiro	agente de saúde				1-3 salários r
Sim, dia inteiro	ACS				1-3 salários r
Sim, dia inteiro	Agencia de turismo				1-3 salários r
Não		Other	criança.	criança	< 1 salário mí
Não		Taking care of children and/or househ		atendente e g	< 1 salário mí
Sim, dia inteiro	Serviços Gerais				1-3 salários r
Sim, dia inteiro	frigorifico				1-3 salários r
Não		Retired		Trabalhava na	< 1 salário mí
Sim, dia inteiro	diarista				< 1 salário mí
Não		Other	criança	criança	< 1 salário mí
Não		Other	pensionista	auxiliar de se	< 1 salário mí
Não		Retired		Lavanderia d	1-3 salários r
Não		Retired		Topografia	1-3 salários r
Sim, meio-dia	auxiliar administrativo				1-3 salários r
Não		Health reasons		Servente de p	< 1 salário mí
Não					< 1 salário mí
Sim, dia inteiro	frentista				< 1 salário mí
Não		Health reasons		atendente	1-3 salários r
Sim, dia inteiro	ajudante de pedreiro				< 1 salário mí
Sim, dia inteiro	vigilante noturno				1-3 salários r
Sim, dia inteiro	funcionário público				>5 salários m
Sim, dia inteiro	Técnico e m quimica				1-3 salários r
Sim, dia inteiro	coordenadora vigilância epidemiologica				>5 salários m
Sim, dia inteiro	diarista				1-3 salários r
Sim, dia inteiro	pedreiro				1-3 salários r
Não		Retired		Frigorifico	1-3 salários r
Sim, dia inteiro	Porteiro				1-3 salários r
Sim, dia inteiro	Motorista				1-3 salários r
Não		Recently laid off		cozinheira	< 1 salário mí
Sim, meio-dia	diarista				1-3 salários r
Não		Retired		Lavadeira	1-3 salários r
Não		Retired		Trabalhador I	< 1 salário mí
Sim, dia inteiro	Ferragens				< 1 salário mí
Não		Retired		Não há	< 1 salário mí

Não		Recently laid off		Colocava pis	1-3 salários r
Sim, dia inteiro	ajudante de pedreiro				1-3 salários r
Não		Retired		Do lar	1-3 salários r
Não		Health reasons		Doméstica	< 1 salário mí
Sim, dia inteiro	gesseiro				1-3 salários r
Sim, dia inteiro	vendedora				1-3 salários r
Não		Retired		Não há.	< 1 salário mí
Sim, dia inteiro	Pedreiro				1-3 salários r
Não		Other	criança	entregando e	Não sei
Não		Recently laid off		Babá	< 1 salário mí
Não		Other	adolescente	nunca trabal	Não sei
Não		Other	menor de idade, estuda	nenhuma cria	1-3 salários r
Não		Recently laid off		atendente	< 1 salário mí
Não		Other	criança	nenhum crian	1-3 salários r
Não		Health reasons		não há	Não sei
Não		Taking care of children and/or househ		Do lar	< 1 salário mí
Não		Other	Estudante	Estudante	1-3 salários r
Sim, meio-dia	Repositora de estoque				1-3 salários r
Não		Other	pensionista	ajudante de c	1-3 salários r
Sim, dia inteiro	Marceneiro				1-3 salários r
Não		Recently laid off		Faxineira	< 1 salário mí
Sim, dia inteiro	Locutor e Coach				1-3 salários r
Não		Retired		Auxiliar de cc	1-3 salários r
Não		Health reasons		Na roça e fax	1-3 salários r
Não		Health reasons		Vaqueiro	< 1 salário mí
Não		Retired		Fábrica de m	< 1 salário mí
Sim, dia inteiro	Agente Comunitário de Saúde				1-3 salários n
Sim, dia inteiro	Não informado				>5 salários m
Sim, dia inteiro	Funcionário Público				1-3 salários r
Não		Retired		Não informac	< 1 salário mí
Não		Taking care of children and/or househ		Trabalho braç	< 1 salário mí
Sim, dia inteiro	Lavrador				1-3 salários r
Não		Other	Do lar	Não informac	1-3 salários r
Não		Retired		Lavadeira	1-3 salários r
Sim, dia inteiro	Serviços gerais, pintura, etc.				1-3 salários r
Não		Retired		Gari	1-3 salários n
Sim, dia inteiro	Comércio				< 1 salário mí
Não		Retired		Doméstica	< 1 salário mí
Não		Other	Estudante/criança	Não se aplica	< 1 salário mí
Não		Taking care of children and/or househ		Empregada d	1-3 salários r
Não		Retired		Não há	< 1 salário mí
Não		Retired		manutenção	1-3 salários r
Não		Taking care of children and/or househ		Faxineira	1-3 salários r

Não		Retired		Serviços gera	1-3 salários r
Não		Taking care of children and/or househ		Não trabalha	< 1 salário mí
Não		Taking care of children and/or househ		Não teve	1-3 salários r
Não		Retired		servente de p	1-3 salários r
Sim, dia inteiro	Diarista				< 1 salário mí
Não		Other	CRIANÇA	NAO HA	< 1 salário mí
Não		Taking care of children and/or househ		Não tem	1-3 salários r
Não		Health reasons		operadora de	< 1 salário mí
Não		Retired		diarista e cap	1-3 salários r
Sim, dia inteiro	Torneiro mecânico				1-3 salários r
Não		Retired		Pedreiro	1-3 salários r
Não		Retired		Cuida do lar	
Sim, dia inteiro	Construção Civil (pedreiro, pintor, eletricista)				1-3 salários r
Não		Retired		Costureira	< 1 salário mí
Não		Health reasons		Cuidadora de	1-3 salários r
Não		Retired		Do lar	1-3 salários r
Não		Retired		Na roça com	1-3 salários r
Não		Taking care of children and/or househ		Padaria/Rece	1-3 salários r
Não		Retired		Doméstica	1-3 salários r
Não		Health reasons		Babá e domé	1-3 salários r
Não		Health reasons		Doméstica	< 1 salário mí
Não		Health reasons		costureira	< 1 salário mí
Não		Other	é menor de idade	Estudante	1-3 salários r
Não		Other	Estudante	EStudante	1-3 salários r
Não		Retired		Doméstica	< 1 salário mí
Não		Taking care of children and/or househ		cabelereira	1-3 salários r
Não		Other	adolescente estudante	estudante	< 1 salário mí
Não		Other	criança	criança	1-3 salários r
Não		Recently laid off		FAXINEIRA	< 1 salário mí
Sim, dia inteiro	BALCONISTA				1-3 salários r
Não		Retired		Serviços gera	1-3 salários r
Sim, dia inteiro	Concreteira (serviço braçal)				1-3 salários r
Sim, meio-dia	Sacoleira				< 1 salário mí
Não		Retired		Pedreiro	1-3 salários r
Não		Other	estudante	estudante	1-3 salários r
Não		Other	criança	criança	< 1 salário mí
Sim, dia inteiro	cozinheira				< 1 salário mí
Sim, dia inteiro	funileiro				1-3 salários r
Não		Taking care of children and/or househ		Do lar	1-3 salários r
Sim, meio-dia	faxina				< 1 salário mí
Não		Health reasons		Faxineira	< 1 salário mí
Não		Retired		lavrador	1-3 salários r
Não		Other	Está desempregado	Marceneiro	1-3 salários r

Não		Retired		Pedreiro, agri	1-3 salários r
Não		Retired			1-3 salários r
Sim, dia inteiro	lavrador				1-3 salários r
Não		Retired		Serviço domé	1-3 salários r
Sim, meio-dia	Colheita de Café				< 1 salário mí
Não		Retired		lavrador	3-5 salários r
Não		Retired		Auxiliar de Se	1-3 salários r
Sim, dia inteiro	pedreiro				3-5 salários r
Não		Retired		Vigia	1-3 salários r
Não		Other	Acidente	Funcionária p	< 1 salário mí
Não		Retired			< 1 salário mí
Não		Retired		Lavrador	1-3 salários r
Sim, dia inteiro	Lavrador				1-3 salários r
Sim, meio-dia	lavrador				1-3 salários r
Sim, dia inteiro	Diarista				< 1 salário mí
Não		Health reasons		costureira, ag	1-3 salários r
Não		Health reasons		Faxineira	< 1 salário mí
Não		Retired			< 1 salário mí
Não		Retired		caseiro	1-3 salários r
Sim, dia inteiro	Pintor automotivo				1-3 salários r
Sim, meio-dia	doméstica				1-3 salários r
Não		Retired		Vendedora de	1-3 salários r
Não		Retired		Prefeitura, re	1-3 salários r
Não		Retired		Zeladora / Lir	1-3 salários r
Não		Taking care of children and/or househ		trabalhador r	< 1 salário mí
Não		Health reasons			1-3 salários r
Não		Health reasons		Capinando	< 1 salário mí
Não		Health reasons		NÃO HÁ	1-3 salários r
Não		Health reasons		NÃO HÁ	1-3 salários n
Não		Taking care of children and/or househ		FÁBRICA DE	1-3 salários r
Sim, meio-dia	COZINHEIRA ESCOLAR				1-3 salários r
Não		Health reasons		PEDREIRO	1-3 salários r
Não		Recently laid off		COZINHEIRA	1-3 salários r
Não		Other	ESTUDANTE	NÃO HÁ	< 1 salário mí
Não		Taking care of children and/or househ		DIARISTA	1-3 salários r
Sim, dia inteiro	AUXILIAR DE LIMPEZA				< 1 salário mí
Não		Health reasons		DOMESTICA	1-3 salários r
Não		Retired		DOMÉSTICA	1-3 salários r
Não		Retired		PEDREIRO, A	< 1 salário mí
Não		Retired		AUXILIAR DE	1-3 salários r
Sim, dia inteiro	PEDREIRO				1-3 salários r
Não		Other	NÃO SE APLICA	NÃO SE APLIC	1-3 salários r
Não		Taking care of children and/or househ		VENDEDORA	1-3 salários r

Sim, dia inteiro	VENDEDOR AUTÔNOMO				< 1 salário mí
Não		Other	NÃO SE APLICA	NÃO SE APLIC	3-5 salários r
Sim, dia inteiro	AUXILIAR DE CONTAS MÉDICAS				3-5 salários r
Sim, dia inteiro	CONFERENTE				3-5 salários r
Não		Other	PESCADOR/SAMARCO		Recusou a res
Não		Retired			1-3 salários r
Não		Taking care of children and/or househ		COBRADOR/	< 1 salário mí
Não		Taking care of children and/or househ		DOMÉSTICA	1-3 salários r
Não		Recently laid off		DIARISTA	1-3 salários r
Sim, dia inteiro	AUTÔNOMO				1-3 salários r
Sim, dia inteiro	AUXILIAR DE SERVIÇOS GERAIS				1-3 salários r
Não		Other	NÃO SE APLICA	NÃO SE APLIC	1-3 salários r
Não		Other	ESTUDANTE	NÃO SE APLIC	1-3 salários r
Não		Taking care of children and/or househ		DOMÉSTICA	1-3 salários r
Não		Other	NÃO SE APLICA	NÃO SE APLIC	1-3 salários r
Não		Retired		EMBALADOR	1-3 salários r
Não		Recently laid off		FAXINEIRA	1-3 salários r
Sim, dia inteiro	FAXINEIRA EM EDIFÍCIO				1-3 salários r
Não		Other	CRIANÇA	NÃO HÁ	1-3 salários r
Não		Other	CRIANÇA	NÃO SE APLIC	1-3 salários r
Não		Other	CRIANÇA	MENOR DE II	1-3 salários r
Não		Other	NUNCA TRABALHOU	NUNCA TRAB	1-3 salários r
Não		Retired		LAVRADORA	1-3 salários r
Não		Retired		TRABALHAD(	1-3 salários r
Não		Recently laid off		BABÁ	1-3 salários r
Sim, dia inteiro	CONSTROI CERCA NA ZONA RURAL				1-3 salários r
Não		Retired		NÃO RESPON	1-3 salários r
Não		Retired		GARI	< 1 salário mí
Sim, dia inteiro	LAVRADORA				1-3 salários r
Sim, meio-dia	COSTUREIRA				< 1 salário mí
Sim, meio-dia	LAVRADOR				< 1 salário mí
Sim, dia inteiro	AGRICULTOR				< 1 salário mí
Não		Health reasons		TRABALHAD(	1-3 salários r
Não		Taking care of children and/or househ		NÃO RESPON	< 1 salário mí
Sim, dia inteiro	LAVRADORA				< 1 salário mí
Sim, dia inteiro	LAVOURA				1-3 salários r
Sim, dia inteiro	LAVRADOR E VIGIA				< 1 salário mí
Não		Other	CRIANÇA	NÃO SE APLIC	< 1 salário mí
Não		Other	CRIANÇA	NÃO SE APLIC	< 1 salário mí
Sim, dia inteiro	VENDEDORA DE PICOLÉ E TRABALHA COM RECICLAGEM				< 1 salário mí
Não		Taking care of children and/or househ		MANICURE	1-3 salários r
Sim, dia inteiro	TRIAGEM DE MATERIAL RECICLADO				1-3 salários r
Não		Taking care of children and/or househ		COSTUREIRA	< 1 salário mí

Não		Retired		Vendedora de	1-3 salários r
Não		Taking care of children and/or househ		DONA DE CA	< 1 salário mí
Não		Other	ESTUDANTE - MENOR D	MENOR DE ID	< 1 salário mí
Não		Health reasons		Doméstica	< 1 salário mí
Não		Other	ESTUDANTE - MENOR D	ESTUDANTE -	< 1 salário mí
Não		Taking care of children and/or househ		ATENDENTE I	1-3 salários r
Sim, dia inteiro	Técnico de Enfermagem				1-3 salários r
Não		Retired		NÃO RESPON	1-3 salários r
Sim, dia inteiro	COZINHEIRA				3-5 salários r
Não		Other	MENOR DE IDADE	MENOR DE ID	1-3 salários r
Sim, meio-dia	PROFESSORA				1-3 salários r
Sim, dia inteiro	AUXILIAR DE DENTISTA				< 1 salário mí
Não		Retired		LAVRADOR	1-3 salários r
Sim, dia inteiro	AGENTE DE PORTARIA - POSTO DE SAÚDE				1-3 salários r
Não		Taking care of children and/or househ		NÃO TRABALI	< 1 salário mí
Não		Retired		Vendedora au	1-3 salários r
Não		Taking care of children and/or househ		FAXINEIRA	1-3 salários r
Não		Retired		TRABALHADO	< 1 salário mí
Não		Retired		Serviços gera	1-3 salários r
Sim, dia inteiro	TRABALHADORA RURAL				< 1 salário mí
Não		Taking care of children and/or househ		NÃO RESPON	1-3 salários r
Não		Health reasons		Faxineira e c	1-3 salários r
Não		Health reasons		Verdureira	< 1 salário mí
Não		Recently laid off		Cuidadora de	< 1 salário mí
Não		Taking care of children and/or househ		Zeladora de p	1-3 salários r
Sim, dia inteiro	Pedreiro				< 1 salário mí
Não		Retired		Manutenção	1-3 salários r
Sim, dia inteiro	Obras				1-3 salários r
Não		Other	Menor de idade	Menor de ida	1-3 salários r
Não		Retired		Doméstica e	1-3 salários r
Não		Temporarily at home		Faxineira	< 1 salário mí
Não		Retired		Operador de	1-3 salários r
Não		Other	Estudante / Criança		< 1 salário mí
Não		Recently laid off		Não informac	< 1 salário mí
Sim, meio-dia	Ajudante de cozinha				1-3 salários r
Não		Other	Pensionista	Auxiliar de se	1-3 salários r
Sim, dia inteiro	Doméstica				1-3 salários n
Sim, dia inteiro	Manutenção de obras em igreja				1-3 salários r
					1-3 salários r
Sim, dia inteiro	Serviços gerais				< 1 salário mí
Não		Health reasons		FAXINEIRA	1-3 salários r
Não		Retired		AGRICULTOR	1-3 salários r
Sim, dia inteiro	MONITORA DE EDUCAÇÃO				1-3 salários r

Não		Recently laid off		TRABALHOU (	< 1 salário mí
Não		Health reasons		COZINHEIRA	< 1 salário mí
Não		Retired		DOMESTICA	1-3 salários r
Não		Recently laid off		SERVIÇOS GI	1-3 salários r
Sim, dia inteiro	CABELELEIRA				1-3 salários r
Não		Other	ESTUDANTE	MENOR DE II	1-3 salários r
Não		Other	ESTUDANTE		1-3 salários r
Não		Other	ESTUDANTE		1-3 salários r
Não		Other	ESTUDANTE	FALTA DE OP(	< 1 salário mí
Não		Retired		TRABALHAD(	1-3 salários r
Sim, dia inteiro	MOTORISTA				1-3 salários r
Não		Temporarily at home		LAVRADOR	< 1 salário mí
Sim, dia inteiro	AGENTE DE SAÚDE				3-5 salários r
Não		Retired		TRABALHAD(	1-3 salários r
Sim, dia inteiro	ENFERMEIRA				>5 salários m
Sim, dia inteiro	EDUCADOR				1-3 salários r
Sim, dia inteiro	AGENTE COMUNITÁRIO DE SAÚDE				< 1 salário mí
Não		Other	MENOR DE IDADE	MENOR DE II	1-3 salários r
Sim, meio-dia	LAVRADOR				1-3 salários r
Sim, dia inteiro	AGENTE COMUNITÁRIO DE SAÚDE				1-3 salários r
Sim, dia inteiro	AGENTE COMUNITÁRIO SAÚDE				1-3 salários r
Não		Other	PENSIONISTA	SEMPRE FOI I	1-3 salários r
Não		Retired		COZINHEIRA	1-3 salários r
Não		Taking care of children and/or househ		NÃO RESPON	< 1 salário mí
Não		Other	MENOR DE IDADE	MENOR DE II	< 1 salário mí
Não		Retired		TRABALHAD(	1-3 salários r
Sim, dia inteiro	CONSTRUTOR CIVIL				1-3 salários r
Não		Taking care of children and/or househ		NÃO RESPON	1-3 salários r
Sim, meio-dia	LAVRADORA				< 1 salário mí
Sim, dia inteiro	LAVRADOR				< 1 salário mí
Sim, dia inteiro	TRABALHADOR RURAL				< 1 salário mí
Sim, dia inteiro	TRABALHADOR RURAL				1-3 salários r
Não		Taking care of children and/or househ		NÃO RESPON	1-3 salários r
Não		Retired		LAVRADOR	1-3 salários r
Sim, dia inteiro	TRABALHADORA RURAL				< 1 salário mí
Não		Other	MENOR DE IDADE	MENOR DE II	Não sei
Sim, dia inteiro	TRABALHADOR RURAL				< 1 salário mí
Sim, dia inteiro	TRABALHADOR RURAL				< 1 salário mí
Não		Temporarily at home		NÃO RESPON	< 1 salário mí
Sim, dia inteiro	TRABALHADOR RURAL				< 1 salário mí
Não		Taking care of children and/or househ		NÃO RESPON	< 1 salário mí
Não		Other	MENOR DE IDADE	MENOR DE II	1-3 salários r
Sim, dia inteiro	TRABALHADORA RURAL				< 1 salário mí

Não		Taking care of children and/or househ		NÃO RESPON	< 1 salário mí
Sim, dia inteiro	LAVRADOR				< 1 salário mí
Sim, dia inteiro	LAVRADORA				< 1 salário mí
Sim, dia inteiro	DOMÉSTICA				< 1 salário mí
Não		Retired		NÃO RESPON	1-3 salários r
Não		Retired		Trabalhadora	1-3 salários r
Não		Taking care of children and/or househ		FAXINEIRA	< 1 salário mí
Não		Retired		LAVRADOR	< 1 salário mí
Não		Health reasons		LAVRADOR	< 1 salário mí
Sim, dia inteiro	Vendedora de telemarketing				< 1 salário mí
Não		Other	Menor de idade (estudante)		< 1 salário mí
Não		Health reasons		Doméstica	< 1 salário mí
Não		Other	CUIDANDO DA MÃE	NUNCA TRAB	1-3 salários r
Não		Retired		NÃO RESPON	1-3 salários r
Não		Retired		TRABALHOU	1-3 salários r
Sim, dia inteiro	AUXILIAR DE SERVIÇOS GERAIS				1-3 salários r
Não		Retired		TRABALHADO	1-3 salários r
Não		Taking care of children and/or househ		NÃO HÁ	1-3 salários r
Não		Other	CRIANÇA	NÃO SE APLIC	1-3 salários r
Sim, dia inteiro	DIARISTA				1-3 salários r
Não		Health reasons		ATENDENTE	1-3 salários r
Não		Recently laid off		GARÇONETE	< 1 salário mí
Não		Other	Estudante		< 1 salário mí
Não		Other	Irá procurar	Não informac	< 1 salário mí
Não		Taking care of children and/or househ		Doméstica, p	3-5 salários r
Não		Other	Estudante		3-5 salários r
Não		Retired		ENTREGAND	1-3 salários r
Não		Recently laid off		Vendedor	1-3 salários r
Não		Other	ESTUDANTE	ESTUDANTE	1-3 salários r
Sim, dia inteiro	OPERADOR DE CAIXA				1-3 salários r
Não		Taking care of children and/or household			1-3 salários r
Sim, dia inteiro	AUXILIAR MECANICO				1-3 salários r
Não		Other	CRIANÇA	CRIANÇA	1-3 salários r
Não		Other	CRIANÇA	CRIANÇA	1-3 salários r
Não		Other	ESTUDANTE	ESTUDANTE	1-3 salários r
Não		Retired		CARPINTEIR	3-5 salários r
Não		Other	ESTUDANTE		1-3 salários r
Sim, dia inteiro	Babá				< 1 salário mí
Não		Temporarily at home		Cuidava de c	< 1 salário mí
Não		Recently laid off		Babá	< 1 salário mí
Sim, meio-dia	Vendedora de loja				1-3 salários r
Não		Recently laid off		Montador de	< 1 salário mí
Sim, meio-dia	Acompanhante de idosa				1-3 salários r

Não		Retired		Distribuidora	3-5 salários r
Não		Retired		Costureira, d	3-5 salários r
Não		Health reasons		Lavadeira	< 1 salário mí
Não		Health reasons		Babá	< 1 salário mí
Não		Other	estudante		< 1 salário mí
Não		Other	Estudante/adolescente	Não há	1-3 salários r
Sim, meio-dia	Vende verduras de folha				1-3 salários r
Não		Taking care of children and/or household			1-3 salários r
Não		Other	Estudante/criança	Não há	1-3 salários r
Não		Other	criança	não se aplica	1-3 salários r
Não		Other	criança		1-3 salários r
Sim, dia inteiro	Borracheiro				1-3 salários r
Não		Taking care of children and/or househ		do lar, faxina	1-3 salários r
Sim, dia inteiro	Faxineira				< 1 salário mí
Sim, meio-dia	Programador.				1-3 salários r
Não		Other	deficiência		1-3 salários r
Não		Retired		Faxineira	1-3 salários r
Sim, dia inteiro	VENDENDORA				1-3 salários r
Não		Retired		Diarista	< 1 salário mí
Sim, dia inteiro	Pedagoga				3-5 salários r
Não		Other	menor de idade		3-5 salários r
Não		Health reasons		FAXINEIRA	< 1 salário mí
Não		Taking care of children and/or househ		Trabalho em	1-3 salários r
Não		Retired		TRABALHADO	1-3 salários r
Sim, dia inteiro	Malote				1-3 salários r
Não		Retired		TRABALHADO	1-3 salários r
Não		Retired		Diarista	1-3 salários r
Não		Retired		Cuidadora, c	1-3 salários r
Não		Retired		LAVRADORA	1-3 salários r
Sim, dia inteiro	AGENTE DE LIMPEZA				1-3 salários r
Sim, meio-dia	ARTESÃ				< 1 salário mí
Não		Retired		LAVADEIRA	1-3 salários r
Sim, meio-dia	DIARISTA				< 1 salário mí
Não		Health reasons		LAVADORA	1-3 salários r
Não		Taking care of children and/or househ		DIARISTA	< 1 salário mí
Não		Retired			< 1 salário mí
Não		Taking care of children and/or househ		AUXILIAR DE	1-3 salários r
Recusou a responder					
Não		Retired		DO LAR	1-3 salários r
Não		Retired		AUXILIAR DE	1-3 salários r
Sim, dia inteiro	AUXILIAR DE SERVIÇOS GERAIS				1-3 salários r
Não		Other	DO LAR		1-3 salários r

Não		Temporarily at home		MANICURE/ I	< 1 salário mí
Não		Retired		DO LAR	1-3 salários r
Não		Taking care of children and/or househ		DOMÉSTICA	1-3 salários r
Não		Temporarily at home		DIARISTA	< 1 salário mí
Não		Retired		AUXILIAR DE	1-3 salários r
Não		Health reasons		LAVRADORA	1-3 salários r
Não		Retired		AUXILIAR DE	1-3 salários r
Não		Health reasons		COMERCIO	< 1 salário mí
Não		Retired		PEDREIRO	1-3 salários n
Não		Health reasons		LAVRADOR	< 1 salário mí
Não		Taking care of children and/or househ		AGRICULTOR	< 1 salário mí
Não		Retired		PEDREIRO E	1-3 salários r
Não		Other	NÃO SE APLICA - CRIAN	NÃO SE APLIC	1-3 salários r
Não		Taking care of children and/or household			1-3 salários r
Não		Retired		COZINHEIRA	1-3 salários r
Não		Retired		VENDEDORA	< 1 salário mí
Sim, dia inteiro	COMÉRCIO				< 1 salário mí
Não		Other	GESTANTE	DO LAR	1-3 salários r
Não		Retired		LAVADORA	1-3 salários r
Não		Retired		SECRETÁRIA	1-3 salários r
Sim, dia inteiro	FEIRANTE				< 1 salário mí
Não		Recently laid off		SOLDADOR	< 1 salário mí
Não		Retired		LAVRADORA/	1-3 salários r
Não		Other	ESTUDANTE	NÃO SE APLIC	1-3 salários r
Não		Taking care of children and/or househ		LAVADEIRA	< 1 salário mí
Não		Other	MENOR DE IDADE	MENOR DE II	1-3 salários r
Não		Other	MENOR DE IDADE	MENOR DE II	< 1 salário mí
Não		Retired		NÃO RESPON	1-3 salários r
Sim, dia inteiro	FÁBRICA DE COSTURA				1-3 salários r
Não		Other	MENOR DE IDADE	MENOR DE II	1-3 salários r
Não		Retired		DOMÉSTICA	1-3 salários r
Não		Health reasons		TRABALHADC	1-3 salários r
Sim, dia inteiro	LAVRADOR				< 1 salário mí
Não		Other	MENOR DE IDADE - EST	MENOR DE II	< 1 salário mí
Não		Other	MENOR DE IDADE - EST	MENOR DE II	< 1 salário mí
Não		Retired		COMERCIAN	>5 salários m
Sim, dia inteiro	LAVRADOR				1-3 salários r
Sim, dia inteiro	Doméstica/Dona de casa				< 1 salário mí
Não		Other	Estudante	Estudante	1-3 salários r
Não		Other	Estudante	Não informac	< 1 salário mí
Não		Other	Estudante	Não informac	< 1 salário mí
Sim, dia inteiro	Domestica				< 1 salário mí
Sim, dia inteiro	Lavrador				< 1 salário mí

Sim, dia inteiro	Lavrador				< 1 salário mí
Sim, dia inteiro	Lavrador				< 1 salário mí
Não		Retired		Lavrador	< 1 salário mí
Sim, dia inteiro	Lavrador				< 1 salário mí
Sim, dia inteiro	Lavrador				< 1 salário mí
Sim, dia inteiro	Lavrador				< 1 salário mí
Sim, dia inteiro	Lavrador = Lavoura de café				< 1 salário mí
Sim, dia inteiro	Lavrador/ lavoura				< 1 salário mí
Sim, dia inteiro	Lavrador				< 1 salário mí
Sim, dia inteiro	Lavrador				< 1 salário mí
Sim, dia inteiro	Fábrica de roupas				< 1 salário mí
Sim, dia inteiro	Fábrica de roupas				< 1 salário mí
Sim, dia inteiro	Fábrica de roupas				< 1 salário mí
Não		Retired		Mecânico	< 1 salário mí
Não		Taking care of children and/or househ		Carpinteira	1-3 salários r
Não					< 1 salário mí
Não		Other	Do lar	Trabalhou em	1-3 salários r
Não		Recently laid off		Faxina	< 1 salário mí
Não					< 1 salário mí
Não		Health reasons		Atendente dc	1-3 salários r
Não		Taking care of children and/or househ		Doméstica; v	< 1 salário mí
Não		Other	CRIANÇA	NÃO SE APLIC	1-3 salários r
Não		Health reasons		Auxiliar de cc	1-3 salários r
Sim, dia inteiro	Pedreiro				1-3 salários r
Não		Other	Menor de idade		< 1 salário mí
Não		Retired		Do lar	1-3 salários r
Não		Other	Do lar	Não informac	1-3 salários r
Não		Retired		Lavroura	< 1 salário mí
Não		Retired		Trabalho rura	< 1 salário mí
Sim, meio-dia	CONFEITEIRA				1-3 salários r
Não		Retired		DOMÉSTICA	< 1 salário mí
Não		Recently laid off		FAXINEIRA E /	< 1 salário mí
Não		Health reasons		NAO HA	1-3 salários r
Não		Health reasons		DOMÉSTICA	1-3 salários r
Sim, dia inteiro	CUIDADORA DE IDOSOS				1-3 salários r
Não		Health reasons		DOMÉSTICA	< 1 salário mí
Sim, dia inteiro	COSTUREIRA				1-3 salários r
Não		Health reasons		NUNCA TRAB	1-3 salários r
Não		Retired		DOMESTICA	1-3 salários r
Não		Other	ADOLESCENTE	ESTUDANTE	< 1 salário mí
Sim, dia inteiro	Babá				< 1 salário mí
Não		Other	Não relatou		< 1 salário mí
Não		Taking care of children and/or househ		Trabalhou em	1-3 salários r

Sim, dia inteiro	Doméstica				< 1 salário mí
Não		Temporarily at home		Do lar	1-3 salários r
Não		Retired		Gari	1-3 salários r
Não		Other	Beneficiada		1-3 salários r
Não		Retired		Nunca trabal	1-3 salários r
Não		Taking care of children and/or househ		Diarista, Sec	< 1 salário mí
Não		Other	Desempregada	Sempre foi do	1-3 salários r
Não		Health reasons		Doméstica	< 1 salário mí
Não		Retired		Do lar	1-3 salários r
Não		Health reasons		NÃO RESPON	< 1 salário mí
Não		Health reasons		Motorista de	1-3 salários r
Sim, dia inteiro	Cabelereira/ representante				3-5 salários r
Não		Retired		Trabalhou em	3-5 salários r
Sim, dia inteiro	Laticinio				1-3 salários r
Sim, dia inteiro	Cuidadora de idoso				1-3 salários r
Sim, dia inteiro	Caixa de supermercado				1-3 salários r
Não		Retired		Mecanico ind	1-3 salários r
Não		Health reasons		do lar	< 1 salário mí
Sim, meio-dia	Jovem aprendiz				1-3 salários r
Não		Health reasons		Auxiliar limpe	1-3 salários r
Sim, meio-dia	Limpeza				1-3 salários r
Não		Retired		Motorista de	1-3 salários r
Não		Retired		Recuso a res	1-3 salários r
Não		Recently laid off		Restaurante	1-3 salários r
Não		Recently laid off		Eletricista de	1-3 salários r
Não		Retired		lavadeira	1-3 salários r
Não		Health reasons		secretaria de	< 1 salário mí
Sim, dia inteiro	JPP (EMPRESA)				1-3 salários r
Sim, dia inteiro	Auxiliar (escola)				1-3 salários r
Não		Taking care of children and/or househ		De lar	1-3 salários r
Não		Retired		Passadeira	1-3 salários r
Não		Other	Estudanto	NA	1-3 salários r
Sim, dia inteiro	restaurante				1-3 salários r
Sim, dia inteiro	Lavadeira				1-3 salários r
Sim, dia inteiro	Const Civil				1-3 salários r
Sim, dia inteiro	Secretaria de lar				1-3 salários r
Não		Health reasons		Faxineira	< 1 salário mí
Não		Other	Estudante, criança	Estudante, cr	< 1 salário mí
Não		Other	Estudante/criança	NA	1-3 salários r
Não		Retired		do lar	< 1 salário mí
Não		Health reasons		Faxineira	1-3 salários r
Não		Retired		NA	1-3 salários r
Não		Taking care of children and/or househ		manicure	1-3 salários r

Sim, dia inteiro	Auxilial de cozinha				< 1 salário mí
Sim, dia inteiro	manicure				< 1 salário mí
Não		Other	Estudanta		< 1 salário mí
Sim, dia inteiro	Vendedor				1-3 salários r
Não		Other	em casa	administrativ	1-3 salários r
Sim, dia inteiro	trabalha fazendo de tudo em doca				1-3 salários r
Sim, dia inteiro	vigia noturno				1-3 salários r
Não		Health reasons		Carpinteiro	1-3 salários r
Sim, dia inteiro	borracheiro				1-3 salários r
Não		Recently laid off		lapidação	1-3 salários r
Sim, dia inteiro	funcionário de prefeitura				1-3 salários r
Sim, dia inteiro	op. de caixa				1-3 salários r
Sim, dia inteiro	agente comunitária de saúde				1-3 salários r
Não		Other	estudante	NA	1-3 salários r
Não		Other	estudante	NA	1-3 salários r
Sim, dia inteiro	recepcionista				1-3 salários r
Não		Retired		metalúrgico	>5 salários m
Sim, dia inteiro	coordenador de vendas				3-5 salários r
Sim, dia inteiro	manicure				1-3 salários r
					1-3 salários r
Não		Health reasons		faxineira	1-3 salários r
Sim, dia inteiro	limpeza				< 1 salário mí
Sim, dia inteiro	motorista e operador de máquinas				1-3 salários r
Sim, dia inteiro	Saladeira / Big Mais				1-3 salários r
Sim, dia inteiro	operadora de caixa em farmácia				1-3 salários r
Sim, dia inteiro	pedreiro				< 1 salário mí
Não		Recently laid off		comércio (ba	1-3 salários r
Sim, dia inteiro	Pedreiro				1-3 salários n
Não		Taking care of children and/or househ		Caixa	3-5 salários r
Não		Other	morador de rua	trabalhar na	< 1 salário mí
Sim, dia inteiro	Diarista				1-3 salários r
Não		Recently laid off		Pedreiro	< 1 salário mí
Não		Other	estudante	NA	1-3 salários r
Não		Retired		pintor	1-3 salários r
Sim, meio-dia	artesã				1-3 salários r
Não		Retired		doméstica	1-3 salários r
Sim, dia inteiro	pedreiro				1-3 salários r
Sim, dia inteiro	Cartorio				1-3 salários r
Sim, dia inteiro	salgadeira				1-3 salários r
Não		Other	NA criança	NA criança	1-3 salários r
Sim, dia inteiro	operador de caixa				1-3 salários r
Não		Recently laid off			< 1 salário mí
Sim, dia inteiro	Construção Civil				1-3 salários r

Sim, dia inteiro	doméstica			1-3 salários r
Não		Recently laid off		1-3 salários r
Sim, dia inteiro	auxiliar de impressão			3-5 salários r
Não		Temporarily at home	Garimpo	< 1 salário mí
Não		Taking care of children and/or househ	nunca trabal	3-5 salários r
Não		Other estudante	estudante	1-3 salários r
Sim, dia inteiro	diarista			1-3 salários r
Sim, dia inteiro	Doméstica			1-3 salários r
Sim, dia inteiro	Técnica em Enfermagem			1-3 salários r
Sim, dia inteiro	Agente Comunitária de Saúde e Recepcionista			1-3 salários r
Não		Health reasons	doméstica	< 1 salário mí
Sim, dia inteiro	Recepcionista do posto			1-3 salários r
Sim, dia inteiro	Dona de bar			3-5 salários r
Não		Retired	Eletricista	< 1 salário mí
Não		Retired	Porteiro, Vigi	1-3 salários r
Não		Retired	Pedreiro	1-3 salários r
Sim, dia inteiro	OPERADORA DE CAIXA			1-3 salários r
Não		Recently laid off	CONTABILID	1-3 salários r
Sim, dia inteiro	CAMINHONEIRO			3-5 salários r
Não		Retired	LAVADEIRA D	1-3 salários r
Não		Taking care of children and/or househ	CANTINEIRA,	3-5 salários n
Não		Other ESTUDANTE	CRIANÇA	1-3 salários r
Não		Recently laid off	FAXINEIRA	1-3 salários r
Não		Taking care of children and/or househ	AUXILIAR DE	1-3 salários r
Não		Recently laid off	CUIDADORA	< 1 salário mí
Não		Other MENOR DE IDADE	MENOR DE II	1-3 salários r
Não		Retired	EMPREGADA	1-3 salários r
Não		Recently laid off	AUXILIAR OPE	1-3 salários r
Não		Retired	PINTOR	< 1 salário mí
Não		Recently laid off	AJUDANTE DI	< 1 salário mí
Não		Taking care of children and/or househ	EMBALADOR	< 1 salário mí
Sim, dia inteiro	RECEPCIONISTA			3-5 salários r
Sim, dia inteiro	FAXINEIRA			1-3 salários r
Não		Health reasons	CUIDADORA	1-3 salários r
Não		Health reasons	ARTESANATO	< 1 salário mí
Não		Retired	NÃO RESPON	1-3 salários r
Sim, dia inteiro	DOMÉSTICA			< 1 salário mí
Não		Retired	FAXINEIRA	1-3 salários r
Não		Retired	METALÚRGIC	1-3 salários r
Sim, dia inteiro	MANICURE			< 1 salário mí
Não		Retired	SAPATEIRA	1-3 salários r
Não		Health reasons	VENDEDORA	1-3 salários r
Sim, dia inteiro	MECÂNICO			1-3 salários r

Não		Retired		NÃO RESPON	1-3 salários r
Sim, dia inteiro	PEDREIRO				1-3 salários r
Não		Retired		OPERADOR [	1-3 salários r
Não		Temporarily at home		NÃO RESPON	1-3 salários r
Sim, dia inteiro	MONITORA DA ADQF				1-3 salários r
Não		Recently laid off		ESTOQUISTA	1-3 salários r
Não		Other	MENOR DE IDADE	MENOR DE II	1-3 salários r
Não		Taking care of children and/or househ		DONA DE CA	< 1 salário mí
Sim, meio-dia	TRABALHADOR BRAÇAL				< 1 salário mí
Não		Retired		FUNCIONÁRI	1-3 salários r
Sim, meio-dia	TRABALHANDO COM CAMPANHAS POLÍTICAS				1-3 salários r
Não		Other	MENOR DE IDADE	MENOR DE II	< 1 salário mí
Sim, dia inteiro	VENDEDORA				1-3 salários r
Não		Retired		TRABALHAVA	< 1 salário mí
Não		Retired		AJUDANTE DI	< 1 salário mí
Sim, meio-dia	NÃO RESPONDEU				< 1 salário mí
Não		Retired		CARPINTEIR(	1-3 salários r
Não		Retired		NÃO RESPON	< 1 salário mí
Sim, dia inteiro	CONFEITEIRO				3-5 salários r
Sim, dia inteiro	AUTÔNOMO				< 1 salário mí
Sim, dia inteiro	VENDEDOR				3-5 salários r
Não		Retired		ARMADOR DI	1-3 salários r
Não		Retired		COZINHEIRA	1-3 salários r
Não		Retired		TORNEIRO, N	1-3 salários r
Não		Recently laid off		TELEMARKETI	1-3 salários r
Não		Recently laid off		DO LAR	1-3 salários r
Sim, dia inteiro	EMPREENDEDORA				< 1 salário mí
Não		Other	MENOR DE IDADE	MENOR DE II	< 1 salário mí
Sim, dia inteiro	PEDREIRO				3-5 salários r
Sim, meio-dia	DOMÉSTICA				1-3 salários r
Não		Taking care of children and/or househ		NÃO TRABALI	< 1 salário mí
Não		Recently laid off		DOMÉSTICA	1-3 salários r
Sim, meio-dia	DIARISTA				< 1 salário mí
Sim, meio-dia	PEDREIRO				< 1 salário mí
Sim, meio-dia	TRABALHANDO NA CAMPANHA POLÍTICA E COMERCIANTE				>5 salários m
Não		Recently laid off		SERVIÇOS GI	< 1 salário mí
Não		Health reasons		NÃO RESPON	< 1 salário mí
Não		Health reasons		DIARISTA	< 1 salário mí
Não		Retired		PROFESSOR/	1-3 salários r
Não		Other	MENOR DE IDADE	MENOR DE II	1-3 salários r
Não		Recently laid off		ATENDENTE	1-3 salários r

Não		Recently laid off		NÃO TRABALI	< 1 salário mí
Sim, meio-dia	CAMPANHA POLÍTICA				< 1 salário mí
Sim, dia inteiro	PROFESSORA				1-3 salários r
Não		Taking care of children and/or househ		CABELEREIR/	1-3 salários r
Não		Retired		MOTORISTA -	1-3 salários r
Sim, dia inteiro	DIARISTA				< 1 salário mí
Não		Taking care of children and/or househ		RECEPCIONI	< 1 salário mí
Sim, dia inteiro	AUXILIAR DE FRIOS				1-3 salários r
Não		Retired		TRABALHOU	1-3 salários r
Não		Retired		SERVENTE DI	1-3 salários r
Não		Health reasons		PASSADEIRA	< 1 salário mí
Sim, dia inteiro	TECNICO ENFERMAGEM				1-3 salários r
Não		Temporarily at home		FAXINEIRA	1-3 salários r
Não		Recently laid off		VENDEDORA	1-3 salários r
Não		Health reasons		CHURRASQL	3-5 salários r
Não		Health reasons		PEDREIRO	1-3 salários r
Não		Recently laid off		ESTUDANTE	1-3 salários r
Não		Taking care of children and/or househ		DIARISTA	1-3 salários r
Não		Recently laid off		FIRMA, REST/	1-3 salários r
Não		Retired		LAVRADOR	1-3 salários r
Não		Retired			1-3 salários r
Não		Retired		VARRER RUA,	1-3 salários r
Não		Health reasons		AJUDANTE DI	< 1 salário mí
Não		Taking care of children and/or househ		SERVIÇO DO	< 1 salário mí
Não		Retired		FREELANCE	1-3 salários r
Não		Recently laid off		COMERCIAN	1-3 salários r
Sim, dia inteiro	BARBEIRO				3-5 salários r
Não		Retired		SERRALHEIR	1-3 salários r
Sim, meio-dia	VENDEDORA				< 1 salário mí
Não		Health reasons		DOMÉSTICA	< 1 salário mí
Sim, meio-dia	AJUDANTE DE PEDREIRO				< 1 salário mí
Sim, dia inteiro	MECÂNICO				1-3 salários r
Sim, dia inteiro	VIGIA				1-3 salários r
Não		Recently laid off		CAIXA DE SUI	< 1 salário mí
Sim, dia inteiro	DIARISTA				< 1 salário mí
Sim, meio-dia	VENDEDORA				< 1 salário mí
Não		Health reasons		AJUDANTE EN	< 1 salário mí
Sim, dia inteiro	SERVIÇOS GERAIS				< 1 salário mí
Não		Other	ESTUDANTE	ESTUDANTE	1-3 salários r
Não		Retired		LAVADEIRA	1-3 salários r
Não		Taking care of children and/or househ		DOMÉSTICA	1-3 salários r
Não		Recently laid off		DESMONTAV	< 1 salário mí
Não		Taking care of children and/or househ		FAXINEIRA E	< 1 salário mí

Não		Recently laid off	POLICIAL, PE	< 1 salário mí
Não		Taking care of children and/or househ	BABÁ E NO G	1-3 salários r
Não		Retired	PORTEIRO, VI	1-3 salários r
Sim, dia inteiro	BARBEIRO			3-5 salários r
Sim, dia inteiro	VENDEDOR			1-3 salários r
Não		Retired	VAQUEIRO - T	1-3 salários r
Sim, meio-dia	VENDEDORA			1-3 salários r
Não		Retired	MECÂNICO	1-3 salários r
Não		Retired	SERVIÇO PÚE	1-3 salários r
Não		Recently laid off	LAPIDÁRIO	< 1 salário mí
Sim, dia inteiro	RECICLAGEM			1-3 salários r
Sim, dia inteiro	CATADOR DE LIXO			< 1 salário mí
Sim, dia inteiro	RECICLAGEM			1-3 salários r
Não		Recently laid off	DOMÉSTICA	1-3 salários r
Sim, dia inteiro	SELECIONA MATERIAL PARA RECICLAGEM PRENSISTA			1-3 salários r
Não		Retired	NA	1-3 salários r
Não		Retired	COZINHEIRA	1-3 salários r
Sim, dia inteiro	PROMOTOR DE VENDAS			< 1 salário mí
Não		Retired	GRAFICA E LA	< 1 salário mí
Não		Retired	FRIGORIFICO	1-3 salários r
Não		Recently laid off	DOMESTICA	1-3 salários r
Não		Retired		1-3 salários r
Não		Retired	DOMESTICA	1-3 salários r
Não		Retired	PEDREIRO	< 1 salário mí
Não		Retired	DOMESTICA	1-3 salários r
Não		Taking care of children and/or househ	TRABALHOU	< 1 salário mí
Não		Retired	EMPREGADA	< 1 salário mí
Não		Health reasons	AUXILIAR DE	1-3 salários r
Sim, dia inteiro	BARBEIRA			1-3 salários r
Sim, dia inteiro	ENFERMEIRA			3-5 salários r
Não		Retired	EMPREGADA	1-3 salários r
Sim, dia inteiro	AUXILIAR DE ESCRITÓRIO			1-3 salários r
Sim, dia inteiro	TÉCNICA EM ENFERMAGEM			1-3 salários r
Sim, dia inteiro	AGENTE ADMINISTRATIVA			1-3 salários r
Não		Taking care of children and/or househ	EMPREGADA	< 1 salário mí
Não		Taking care of children and/or househ	DIARISTA	1-3 salários r
Sim, meio-dia	VIGILANTE			1-3 salários r
Sim, dia inteiro	SERVENTE DE PEDREIRO			1-3 salários r
Sim, dia inteiro	TRABALHA COM CONSTRUÇÃO CIVIL			1-3 salários r
Recusou a responder				Recusou a re
Sim, meio-dia	RECICLAGEM			1-3 salários r
Não		Health reasons	AGENTE DE S	< 1 salário mí
Não		Retired	NÃO RESPON	Recusou a re

Não	Retired		NÃO RESPON	Recusou a res
Recusou a responder				Recusou a res
Recusou a responder				Recusou a res
Recusou a responder				Recusou a res
Recusou a responder				Recusou a res
Recusou a responder				Recusou a res
Recusou a responder				Recusou a res
Recusou a responder				Recusou a res
Não	Other	MENOR DE IDADE	MENOR DE II	Não sei
Recusou a responder				Recusou a res
Recusou a responder				Recusou a res
Recusou a responder				Recusou a res
Não	Retired		NÃO RESPON	Recusou a res
Recusou a responder				Recusou a res
Não	Other	MENOR DE IDADE	MENOR DE II	Não sei
Recusou a responder				1-3 salários r
Recusou a responder				Recusou a res
Recusou a responder				Recusou a res
Recusou a responder				Recusou a res
Recusou a responder				Recusou a res
Recusou a responder				Recusou a res
Recusou a responder				Recusou a res
Recusou a responder				Recusou a res
Recusou a responder				Recusou a res
Não	Other	MENOR DE IDADE	MENOR DE II	Recusou a res
Recusou a responder				Recusou a res

Quantas pess	Qual o seu ma	Você tem u	Você compar	Quantas pe	Você já foi dia	Se sim, você s	Se sim, você s
2	Ensino fundar	Sim		0-3	Sim	Checked	Unchecked
3	Ensino médic	Sim	Sim	0-3	Sim	Unchecked	Unchecked
3	Ensino médic	Sim	Sim	0-3	Sim	Unchecked	Unchecked
1	Não sei	Não	Não	0-3	Não sei	Unchecked	Unchecked
2	Ensino fundar	Sim	Sim	0-3	Sim	Checked	Unchecked
6	Ensino fundar	Sim	Sim	0-3	Sim	Unchecked	Unchecked
	Ensino médic	Sim	Sim	0-3	Sim	Checked	Unchecked
	Nenhum	Sim	Sim	0-3	Não	Unchecked	Unchecked
	Nenhum	Sim	Sim	0-3	Não	Unchecked	Unchecked
6	Ensino médic	Sim	Sim	0-3	Não	Unchecked	Unchecked
2	Ensino médic	Não	Sim	0-3	Sim	Checked	Unchecked
6	Ensino fundar	Sim	Sim	0-3	Sim	Checked	Unchecked
3	Ensino fundar	Sim	Sim	0-3	Sim	Unchecked	Unchecked
3	Nenhum	Sim	Sim	0-3	Não	Unchecked	Unchecked
10	Ensino médic	Sim	Sim	0-3	Sim	Checked	Unchecked
6	Ensino fundar	Sim	Não	0-3	Não	Unchecked	Unchecked
10	Ensino médic	Não	Sim	0-3	Sim	Checked	Unchecked
4	Ensino médic	Sim	Sim	0-3	Não	Unchecked	Unchecked
6	Ensino médic	Sim	Sim	0-3	Não	Unchecked	Unchecked
1	Nenhum	Não	Não	0-3	Sim	Checked	Unchecked
2	Nenhum	Não	Sim	0-3	Sim	Unchecked	Unchecked
2	Nível Técnico	Sim	Não	0-3	Sim	Unchecked	Unchecked
2	Ensino fundar	Não	Sim	0-3	Sim	Unchecked	Unchecked
5	Ensino médic	Não	Sim	0-3	Não	Unchecked	Unchecked
2	Ensino fundar	Não	Sim	0-3	Sim	Checked	Unchecked
2	Nenhum	Não	Sim	0-3	Sim	Checked	Unchecked
2	Ensino fundar	Sim	Sim	0-3	Sim	Checked	Unchecked
5	Ensino fundar	Sim	Sim	0-3	Sim	Checked	Unchecked
3	Ensino fundar	Não	Sim	0-3	Sim	Checked	Unchecked
1	Ensino fundar	Não	Não	0-3	Não	Unchecked	Unchecked
5	Ensino fundar	Sim	Não	0-3	Sim	Unchecked	Unchecked
6	Ensino fundar	Sim	Sim	0-3	Sim	Checked	Unchecked
2	Ensino fundar	Não	Sim	0-3	Não	Unchecked	Unchecked
2	Ensino fundar	Não	Sim	0-3	Sim	Unchecked	Unchecked
3	Ensino médic	Não	Não	0-3	Não	Unchecked	Unchecked
6	Ensino fundar	Sim	Sim	0-3	Sim	Checked	Checked
6	Ensino fundar	Não	Sim	0-3	Sim	Unchecked	Checked
3	Ensino médic	Sim	Sim	0-3	Sim	Unchecked	Unchecked
3	Ensino fundar	Não sei	Não	0-3	Não sei	Unchecked	Unchecked
2	Nenhum	Sim	Não	0-3	Não	Unchecked	Unchecked
2	Ensino fundar	Sim	Não	0-3	Sim	Unchecked	Checked
2	Nenhum	Sim	Não	0-3	Não	Unchecked	Unchecked

	2	Ensino funda	Não sei	Não	0-3	Sim	Unchecked	Unchecked
	2	Ensino médic	Não	Sim	0-3	Sim	Unchecked	Unchecked
	3	Ensino funda	Sim	Não	0-3	Sim	Unchecked	Checked
	2	Nenhum	Não	Sim	0-3	Sim	Checked	Unchecked
	3	Ensino médic	Sim	Não	0-3	Sim	Unchecked	Unchecked
	2	Ensino funda	Não	Não	0-3	Sim	Unchecked	Unchecked
	5	Ensino médic	Sim	Sim	4+	Sim	Unchecked	Checked
	3	Ensino funda	Sim	Sim	0-3	Não	Unchecked	Unchecked
	4	Ensino funda	Sim	Sim	0-3	Sim	Unchecked	Unchecked
	3	Ensino funda	Sim	Sim	0-3	Não	Unchecked	Unchecked
	6	Ensino médic	Sim	Sim	4+	Não	Unchecked	Unchecked
	6	Nenhum	Sim	Sim	0-3	Não	Unchecked	Unchecked
	3	Ensino funda	Sim	Sim	0-3	Sim	Checked	Unchecked
nimo		Ensino funda	Não	Não	0-3	Sim	Unchecked	Unchecked
	3	Ensino funda	Sim	Sim	0-3	Sim	Unchecked	Unchecked
	2	Ensino funda	Sim	Sim	0-3	Não	Unchecked	Unchecked
	6	Ensino funda	Sim	Não	0-3	Não	Unchecked	Unchecked
	2	Nenhum	Não	Sim	0-3	Não	Unchecked	Unchecked
	1	Ensino funda	Não	Sim	0-3	Não	Unchecked	Unchecked
	3	Ensino funda	Sim	Sim	0-3	Sim	Checked	Unchecked
	3	Ensino funda	Sim	Sim	0-3	Sim	Unchecked	Unchecked
	2	Nenhum	Não	Não	0-3	Sim	Unchecked	Unchecked
	3	Ensino funda	Sim	Sim	0-3	Não	Unchecked	Unchecked
	2	Nenhum	Não	Não	0-3	Não	Unchecked	Unchecked
	1	Nenhum	Não	Sim	0-3	Sim	Unchecked	Checked
	2	Ensino funda	Não	Sim	0-3	Não	Unchecked	Unchecked
	2	Ensino médic	Sim	Sim	0-3	Sim	Unchecked	Checked
	1	Nenhum	Sim	Não	0-3	Não	Unchecked	Unchecked
	6	Ensino médic	Sim	Não	0-3	Não	Unchecked	Unchecked
	5	Ensino funda	Sim	Não	4+	Não	Unchecked	Unchecked
	2	Ensino funda	Sim	Sim	0-3	Sim	Unchecked	Checked
	2	Ensino médic	Sim	Não	0-3	Sim	Unchecked	Unchecked
	4	Ensino médic	Sim	Sim	0-3	Não	Unchecked	Unchecked
	4	Ensino funda	Não	Sim	4+	Sim	Checked	Unchecked
	3	Nenhum	Sim	Não	0-3	Não	Unchecked	Unchecked
	2	Ensino funda	Sim	Não	0-3	Sim	Unchecked	Unchecked
	3	Ensino funda	Sim	Sim	0-3	Não	Unchecked	Unchecked
	2	Nenhum	Sim	Sim	0-3	Sim	Checked	Unchecked
	4	Ensino médic	Sim	Não	4+	Sim	Checked	Unchecked
	4	Ensino médic	Sim	Sim	0-3	Não	Unchecked	Unchecked
	2	Ensino médic	Sim	Sim	0-3	Não	Unchecked	Unchecked
	2	Ensino funda	Sim	Não	0-3	Não	Unchecked	Unchecked
	1	Ensino funda	Sim	Não	0-3	Sim	Unchecked	Checked

	3	Ensino funda	Sim	Sim	0-3	Não sei	Unchecked	Unchecked
	2	Nenhum	Não	Sim	0-3	Não	Unchecked	Unchecked
	2	Ensino médic	Sim	Sim	0-3	Sim	Checked	Unchecked
	2	Ensino funda	Sim	Sim	0-3	Sim	Unchecked	Unchecked
	5	Ensino funda	Sim	Sim	4+	Sim	Checked	Unchecked
	4	Ensino médic	Sim	Sim	0-3	Não	Unchecked	Unchecked
	2	Nenhum	Sim	Sim	0-3	Não	Unchecked	Unchecked
	2	Ensino funda	Sim	Sim	0-3	Sim	Unchecked	Unchecked
	3	Ensino funda	Sim	Sim	0-3	Sim	Unchecked	Unchecked
	2	Ensino funda	Não	Sim	0-3	Sim	Unchecked	Unchecked
	2	Ensino funda	Sim	Não	0-3	Não	Unchecked	Unchecked
	3	Ensino funda	Sim	Não	0-3	Sim	Unchecked	Unchecked
	3	Ensino funda	Sim	Não	0-3	Não	Unchecked	Unchecked
	2	Ensino médic	Sim	Sim	0-3	Sim	Unchecked	Unchecked
	2	Ensino funda	Sim	Sim	0-3	Sim	Checked	Unchecked
	3	Ensino funda	Sim	Não	0-3	Não	Unchecked	Unchecked
	3	Ensino médic	Sim	Não	0-3	Não	Unchecked	Unchecked
	3	Ensino funda	Não	Sim	0-3	Sim	Checked	Unchecked
	4	Ensino funda	Sim	Sim	0-3	Sim	Unchecked	Unchecked
	2	Ensino funda	Sim	Sim	0-3	Sim	Checked	Unchecked
	11	Ensino funda	Sim	Sim	0-3	Sim	Checked	Unchecked
	5	Ensino funda	Não	Sim	4+	Não sei	Unchecked	Unchecked
	4	Ensino funda	Sim	Sim	0-3	Sim	Checked	Checked
	4	Ensino funda	Não	Sim	0-3	Sim	Checked	Unchecked
	4	Ensino médic	Sim	Sim	0-3	Sim	Unchecked	Unchecked
	2	Ensino funda	Não	Sim	0-3	Sim	Unchecked	Checked
	2	Ensino funda	Sim	Sim	0-3	Sim	Unchecked	Unchecked
	1	Ensino funda	Não	Não	0-3	Sim	Checked	Unchecked
	1	Nível superio	Não	Não	0-3	Não	Unchecked	Unchecked
	3	Ensino funda	Não	Sim	0-3	Sim	Checked	Unchecked
	2	Ensino funda	Sim	Sim	0-3	Sim	Unchecked	Checked
	1	Ensino funda	Não	Sim	0-3	Sim	Unchecked	Unchecked
	4	Ensino funda	Sim	Sim	0-3	Não	Unchecked	Unchecked
	2	Ensino funda	Sim	Sim	0-3	Sim	Checked	Unchecked
	1	Ensino médic	Sim	Não	0-3	Sim	Unchecked	Checked
	1	Ensino médic	Sim	Não	0-3	Sim	Checked	Unchecked
	1	Ensino funda	Sim	Sim	0-3	Sim	Checked	Unchecked
	2	Ensino funda	Sim	Não	0-3	Sim	Checked	Unchecked
nimo		Ensino funda	Sim	Não	0-3	Não	Unchecked	Unchecked
	1	Ensino funda	Não	Não	4+	Sim	Checked	Checked
	2	Ensino médic	Sim	Não	0-3	Não	Unchecked	Unchecked
	4	Nenhum	Não	Não	0-3	Sim	Unchecked	Checked
	1	Nenhum	Sim	Não	0-3	Sim	Unchecked	Unchecked

	1	Ensino funda	Não	Não	0-3	Sim	Checked	Unchecked
	4	Ensino médic	Não	Não	0-3	Sim	Unchecked	Checked
	2	Ensino funda	Sim	Sim	0-3	Sim	Unchecked	Checked
	2	Ensino funda	Não	Sim	0-3	Sim	Unchecked	Checked
	3	Ensino funda	Não	Sim	0-3	Sim	Unchecked	Checked
	3	Ensino funda	Sim	Sim	0-3	Sim	Checked	Checked
	1	Ensino funda	Sim	Não	0-3	Sim	Checked	Checked
	2	Ensino funda	Sim	Não	0-3	Sim	Unchecked	Checked
	4	Ensino funda	Não	Sim	0-3	Sim	Checked	Checked
	5	Ensino médic	Sim	Sim	0-3	Sim	Checked	Unchecked
	2	Ensino funda	Sim	Sim	0-3	Sim	Checked	Checked
	6	Ensino funda	Sim	Não	0-3	Sim	Unchecked	Checked
	2	Ensino funda	Sim	Não	0-3	Não	Unchecked	Unchecked
	4	Ensino funda	Sim	Não	0-3	Sim	Checked	Checked
	2	Ensino médic	Sim	Sim	0-3	Sim	Unchecked	Checked
	2	Ensino funda	Sim	Não	0-3	Não	Unchecked	Unchecked
	2	Nenhum	Não	Não	0-3	Sim	Checked	Checked
	2	Ensino funda	Sim	Sim	0-3	Sim	Unchecked	Checked
	1	Ensino funda	Não	Sim	0-3	Sim	Checked	Checked
	3	Nenhum	Não	Sim	0-3	Não	Unchecked	Unchecked
	4	Ensino médic	Sim	Sim	0-3	Sim	Checked	Unchecked
	2	Não sei	Sim	Sim	0-3	Sim	Unchecked	Checked
nínimos		Ensino funda	Sim	Sim	0-3	Sim	Checked	Unchecked
	1	Ensino funda	Sim	Não	0-3	Sim	Checked	Unchecked
	3	Ensino funda	Sim	Não	0-3	Sim	Checked	Checked
	1	Ensino funda	Sim	Não	4+	Sim	Unchecked	Unchecked
	2	Ensino funda	Sim	Sim	0-3	Sim	Checked	Checked
	2	Ensino funda	Sim	Não	0-3	Sim	Unchecked	Checked
	4	Ensino funda	Sim	Sim	0-3	Sim	Unchecked	Unchecked
nínimos		Ensino funda	Sim	Não	0-3	Sim	Checked	Checked
	2	Ensino funda	Não	Sim	0-3	Sim	Checked	Checked
	1	Ensino médic	Sim	Sim	0-3	Sim	Unchecked	Checked
	8	Ensino funda	Não	Sim	0-3	Não	Unchecked	Unchecked
	3	Ensino funda	Sim	Não	0-3	Sim	Unchecked	Checked
	3	Ensino médic	Sim	Sim	0-3	Sim	Unchecked	Unchecked
	2	Ensino funda	Sim	Sim	0-3	Não	Unchecked	Unchecked
	1	Ensino funda	Sim	Sim	0-3	Sim	Unchecked	Checked
	2	Ensino funda	Sim	Sim	0-3	Não sei	Unchecked	Unchecked
	1	Ensino médic	Sim	Não	0-3	Sim	Checked	Unchecked
	2	Ensino médic	Sim	Sim	0-3	Sim	Unchecked	Checked
	2	Ensino funda	Sim	Sim	0-3	Sim	Checked	Checked
	4	Ensino funda	Sim	Sim	0-3	Sim	Unchecked	Unchecked
	4	Ensino médic	Sim	Sim	0-3	Sim	Checked	Unchecked

	2	Ensino médic	Sim	Não	0-3	Sim	Checked	Checked
	4	Nenhum	Sim	Sim	0-3	Sim	Unchecked	Checked
	4	Ensino funda	Sim	Não	0-3	Sim	Unchecked	Unchecked
	1	Ensino funda	Sim	Não	0-3	Sim	Checked	Unchecked
	4	Ensino funda	Sim	Sim	0-3	Sim	Checked	Unchecked
	3	Ensino médic	Sim	Sim	0-3	Sim	Unchecked	Unchecked
	3	Ensino funda	Sim	Sim	0-3	Sim	Checked	Unchecked
	1	Ensino médic	Sim	Sim	0-3	Sim	Unchecked	Unchecked
	7	Ensino médic	Sim	Sim	0-3	Sim	Checked	Checked
	3	Nenhum	Sim	Não	0-3	Sim	Unchecked	Checked
	2	Ensino funda	Não	Não	0-3	Sim	Checked	Checked
	1	Ensino funda	Não	Não	0-3	Sim	Unchecked	Checked
	3	Ensino funda	Sim	Sim	0-3	Não sei	Unchecked	Unchecked
	4	Ensino médic	Sim		0-3	Sim	Unchecked	Checked
	3	Ensino funda	Sim	Não	0-3	Sim	Unchecked	Unchecked
	3	Ensino médic	Recusou a	Sim	0-3	Sim	Checked	Unchecked
	3	Nenhum	Sim	Sim	0-3	Não	Unchecked	Unchecked
	2	Ensino médic	Sim	Não	0-3	Não	Unchecked	Unchecked
	4	Ensino funda	Sim	Não	4+	Não	Unchecked	Unchecked
	1	Ensino funda	Sim	Não	0-3	Sim	Checked	Checked
	1	Ensino funda	Não	Não	0-3	Não	Unchecked	Unchecked
	3	Ensino funda	Não	Sim	0-3	Não	Unchecked	Unchecked
		Ensino funda	Sim	Não	4+	Não	Unchecked	Unchecked
	3	Ensino médic	Sim	Não	0-3	Sim	Checked	Unchecked
	3	Ensino funda	Sim	Não	0-3	Sim	Unchecked	Unchecked
	2	Nenhum	Não	Não	0-3	Não	Unchecked	Unchecked
	3	Nenhum	Não sei	Sim	0-3	Sim	Checked	Unchecked
	2	Ensino médic	Não	Sim	0-3	Não	Unchecked	Unchecked
	4	Ensino funda	Não	Não	0-3	Sim	Unchecked	Checked
nimo		Nenhum	Sim	Sim	0-3	Sim	Unchecked	Checked
	4	Ensino médic	Sim	Não	0-3	Sim	Checked	Unchecked
	1	Ensino médic	Sim	Não	0-3	Recusou a re	Unchecked	Unchecked
	2	Ensino funda	Não	Sim	0-3	Sim	Unchecked	Checked
	3	Ensino funda	Não	Não	0-3	Sim	Checked	Unchecked
	3	Ensino funda	Recusou a	Sim	0-3	Sim	Unchecked	Checked
	4	Nível Técnico	Sim	Não	0-3	Não	Unchecked	Unchecked
	1	Ensino funda	Não sei	Não	0-3	Sim	Checked	Unchecked
	4	Ensino médic	Sim	Sim	4+	Não sei	Unchecked	Unchecked
	3	Ensino funda	Sim	Sim	0-3	Não sei	Unchecked	Unchecked
	4	Ensino funda	Sim	Não	0-3	Não	Unchecked	Unchecked
	1	Ensino funda	Não	Não	0-3	Sim	Checked	Checked
		Ensino funda	Sim	Não	4+	Não	Unchecked	Unchecked
nínimos		Ensino funda	Sim	Não	4+	Sim	Checked	Unchecked

3	Nenhum	Não sei	Não	0-3	Não	Unchecked	Unchecked
2	Ensino funda	Sim	Sim	0-3	Sim	Checked	Checked
3	Nenhum	Sim	Sim	0-3	Sim	Unchecked	Unchecked
5	Ensino médic	Sim	Sim	4+	Sim	Unchecked	Checked
1	Ensino médic	Não	Não	0-3	Sim	Unchecked	Checked
5	Ensino funda	Não	Sim	0-3	Sim	Unchecked	Checked
2	Ensino funda	Sim	Sim	0-3	Sim	Checked	Unchecked
2	Nenhum	Não	Sim	0-3	Não	Unchecked	Unchecked
1	Ensino funda	Não	Não	0-3	Não	Unchecked	Unchecked
2	Nenhum	Não sei	Sim	0-3	Não	Unchecked	Unchecked
	Ensino funda	Sim	Não	0-3	Sim	Checked	Unchecked
2	Ensino funda	Não	Sim	0-3	Sim	Checked	Unchecked
3	Ensino funda	Sim	Não	0-3	Sim	Checked	Unchecked
5	Ensino médic	Sim	Sim	0-3	Sim	Unchecked	Unchecked
3	Ensino funda	Sim	Sim	0-3	Não	Unchecked	Unchecked
3	Nenhum	Sim	Sim	0-3	Não	Unchecked	Unchecked
2	Ensino funda	Sim	Não	0-3	Sim	Checked	Checked
2	Ensino funda	Não	Sim	0-3	Não	Unchecked	Unchecked
4	Ensino funda	Recusou a	Não	4+	Sim	Checked	Checked
2	Pós-graduaçã	Sim	Sim	0-3	Sim	Checked	Unchecked
2	Ensino funda	Não	Sim	0-3	Sim	Unchecked	Checked
2	Nenhum	Sim	Sim	0-3	Não	Unchecked	Unchecked
3	Nenhum	Sim	Não	0-3	Sim	Checked	Unchecked
2	Ensino funda	Não sei	Sim	0-3	Sim	Unchecked	Unchecked
4	Ensino funda	Não	Sim	4+	Sim	Unchecked	Unchecked
2	Nenhum	Sim	Não	0-3	Sim	Checked	Unchecked
1	Ensino médic	Sim	Não	0-3	Não	Unchecked	Unchecked
5	Ensino funda	Sim	Sim	0-3	Sim	Checked	Unchecked
5	Ensino funda	Sim	Sim	0-3	Não sei	Unchecked	Unchecked
1	Ensino funda	Sim	Não	0-3	Não	Unchecked	Unchecked
2	Nenhum	Sim	Sim	0-3	Sim	Unchecked	Checked
1	Ensino funda	Não	Não	0-3	Não	Unchecked	Unchecked
4	Ensino funda	Sim	Sim	4+	Sim	Unchecked	Unchecked
	Ensino funda	Sim	Sim	0-3	Não	Unchecked	Unchecked
3	Ensino funda	Sim	Sim	0-3	Sim	Unchecked	Unchecked
3	Ensino funda	Sim	Sim	0-3	Sim	Unchecked	Checked
2	Ensino funda	Não	Sim	0-3	Não	Unchecked	Unchecked
4	Ensino funda	Sim	Sim	0-3	Sim	Checked	Unchecked
3	Ensino funda	Sim	Não	0-3	Sim	Checked	Unchecked
2	Ensino médic	Sim	Sim	0-3	Sim	Unchecked	Unchecked
3	Ensino funda	Sim	Não	0-3	Sim	Checked	Unchecked
1	Ensino funda	Sim	Não	0-3	Sim	Unchecked	Unchecked
3	Ensino funda	Sim	Não	0-3	Sim	Checked	Unchecked

3	Ensino funda	Não	Sim	0-3	Sim	Checked	Unchecked
1	Ensino funda	Não	Não	0-3	Sim	Checked	Unchecked
2	Ensino funda	Não	Sim	0-3	Sim	Unchecked	Unchecked
3	Ensino funda	Sim	Sim	0-3	Não	Unchecked	Unchecked
4	Ensino médic	Sim	Sim	4+	Não	Unchecked	Unchecked
2	Ensino funda	Não	Não	0-3	Não sei	Unchecked	Unchecked
1	Ensino funda	Sim	Não	0-3	Sim	Checked	Unchecked
4	Ensino funda	Sim	Sim	0-3	Não	Unchecked	Unchecked
3	Ensino médic	Sim	Sim	0-3	Sim	Checked	Checked
1	Nenhum	Não	Sim	0-3	Sim	Unchecked	Unchecked
1	Ensino funda	Sim	Não	0-3	Não	Unchecked	Unchecked
3	Recusou a re	Sim	Sim	0-3	Não	Unchecked	Unchecked
3	Ensino funda	Sim	Sim	0-3	Sim	Checked	Unchecked
3	Ensino funda	Sim	Sim	0-3	Sim	Checked	Unchecked
4	Ensino médic	Sim	Não	0-3	Sim	Unchecked	Checked
3	Ensino funda	Sim	Recusou a responder		Sim	Checked	Unchecked
2	Ensino médic	Sim	Sim	0-3	Sim	Unchecked	Unchecked
3	Ensino funda	Sim	Sim	0-3	Não	Unchecked	Unchecked
9	Nenhum	Não	Não	0-3	Sim	Checked	Unchecked
3	Ensino médic	Não	Sim	0-3	Sim	Unchecked	Checked
2	Ensino funda	Sim	Não	0-3	Sim	Checked	Unchecked
8	Ensino funda	Sim	Sim	0-3	Sim	Unchecked	Unchecked
2	Ensino funda	Sim	Não	4+	Sim	Checked	Checked
2	Ensino funda	Não	Não	0-3	Sim	Unchecked	Unchecked
3	Ensino funda	Sim	Não	0-3	Sim	Checked	Unchecked
5	Ensino funda	Sim	Não	4+	Sim	Checked	Checked
4	Ensino funda	Sim	Sim	0-3	Sim	Unchecked	Checked
1	Ensino funda	Sim	Não	0-3		Unchecked	Unchecked
2	Ensino funda	Não	Sim	0-3	Sim	Unchecked	Unchecked
3	Ensino médic	Sim	Sim	0-3	Não	Unchecked	Unchecked
4	Ensino funda	Sim	Não	4+	Sim	Checked	Checked
2	Ensino funda	Não	Não	Não sei	Sim	Unchecked	Checked
2	Nível superio	Sim	Sim	0-3	Não	Unchecked	Unchecked
3	Ensino funda	Sim	Sim	0-3	Sim	Unchecked	Checked
	Ensino funda	Sim	Não	4+	Não sei	Unchecked	Unchecked
2	Ensino funda	Não	Sim	0-3	Sim	Unchecked	Checked
9	Nível superio	Sim	Não	0-3	Sim	Checked	Unchecked
3	Ensino funda	Sim	Não	0-3	Não	Unchecked	Unchecked
1	Nenhum	Sim	Não	0-3	Sim	Unchecked	Unchecked
6	Ensino funda	Sim	Não	4+	Não sei	Unchecked	Unchecked
3	Ensino funda	Não	Sim	4+	Sim	Unchecked	Checked
3	Ensino médic	Sim	Sim	0-3	Sim	Unchecked	Checked
2	Ensino funda	Recusou a	Sim	0-3	Sim	Unchecked	Checked

	2	Ensino funda	Não	Não	0-3	Sim	Unchecked	Unchecked
	3	Ensino funda	Não	Não	Recusou a	Não	Unchecked	Unchecked
sponder		Ensino funda	Sim	Sim	0-3	Sim	Checked	Unchecked
	3	Ensino funda	Sim	Sim	0-3	Sim	Checked	Checked
	4	Ensino funda	Recusou a	Não	0-3	Não	Unchecked	Unchecked
	2	Ensino funda	Não	Não	0-3	Sim	Checked	Checked
	5	Nível superio	Sim	Sim	4+	Não	Unchecked	Unchecked
	7	Ensino funda	Sim	Sim	4+	Sim	Checked	Unchecked
	3	Ensino funda	Sim	Sim	0-3	Não sei	Unchecked	Unchecked
	3	Pós-graduaçã	Sim	Não	0-3	Sim	Checked	Checked
	2	Ensino funda	Não	Não	0-3	Sim	Unchecked	Checked
	4	Ensino médic	Sim	Não	0-3	Não	Unchecked	Unchecked
	4	Ensino funda	Sim	Não	0-3	Sim	Checked	Unchecked
	3	Ensino médic	Sim	Sim	0-3	Sim	Checked	Checked
	2	Ensino funda	Não	Sim	4+	Sim	Unchecked	Unchecked
	1	Ensino funda	Não	Não	0-3	Sim	Unchecked	Checked
	2	Ensino funda	Sim	Não	0-3	Sim	Unchecked	Checked
	2	Ensino funda	Não sei	Sim	0-3	Não	Unchecked	Unchecked
	3	Nenhum	Sim	Não	0-3	Sim	Checked	Unchecked
	3	Ensino médic	Não sei	Não	0-3	Não	Unchecked	Unchecked
	3	Nenhum	Não	Sim	0-3	Sim	Checked	Unchecked
	5	Ensino fundamental		Não	0-3	Não	Unchecked	Unchecked
	2	Ensino fundamental		Sim	0-3	Sim	Unchecked	Checked
	3	Nenhum	Sim	Não	0-3	Sim	Unchecked	Checked
	1	Ensino funda	Recusou a	Não	0-3	Sim	Unchecked	Unchecked
	1	Ensino funda	Sim	Não	0-3	Não	Unchecked	Unchecked
	2	Ensino funda	Sim	Sim	0-3	Sim	Unchecked	Checked
	1	Ensino funda	Sim	Não	0-3	Não	Unchecked	Unchecked
	1	Ensino médic	Não	Não	0-3	Sim	Unchecked	Unchecked
	2	Ensino funda	Recusou a	Sim	0-3	Sim	Unchecked	Unchecked
	3	Ensino funda	Não	Sim	0-3	Sim	Unchecked	Unchecked
	2	Ensino funda	Não	Não	0-3	Sim	Unchecked	Checked
	3	Ensino funda	Não	Sim	0-3	Sim	Checked	Checked
	1	Ensino funda	Sim	Não	0-3	Não sei	Unchecked	Unchecked
	3	Nenhum	Não	Sim	0-3	Sim	Checked	Unchecked
	4	Ensino médic	Sim	Sim	0-3	Sim	Unchecked	Unchecked
	6	Ensino funda	Não	Sim	0-3	Não	Unchecked	Unchecked
	2	Nenhum	Sim	Sim	0-3	Não	Unchecked	Unchecked
	5	Ensino funda	Recusou a	Não	4+	Não	Unchecked	Unchecked
	2	Ensino funda	Sim	Sim	0-3	Sim	Checked	Unchecked
	2	Ensino funda	Sim	Não	0-3	Não	Unchecked	Unchecked
	2	Ensino médic	Sim	Sim	0-3	Sim	Checked	Unchecked
	4	Nenhum	Sim	Não	4+	Não	Unchecked	Unchecked

	2	Ensino funda	Não	Não	0-3	Não sei	Unchecked	Unchecked
	2	Ensino funda	Recusou a	Não	0-3	Não	Unchecked	Unchecked
	3	Nível superio	Sim	Sim	0-3	Sim	Unchecked	Unchecked
	9	Ensino funda	Não	Sim	4+	Sim	Checked	Unchecked
	3	Ensino médic	Não	Sim	0-3	Não	Unchecked	Unchecked
	2	Ensino funda	Sim	Sim	0-3	Sim	Unchecked	Checked
	2	Nenhum	Sim	Não	0-3	Sim	Unchecked	Unchecked
sponder		Recusou a re	Sim	Não	0-3	Sim	Checked	Unchecked
	1	Ensino funda	Sim	Não	0-3	Sim	Checked	Unchecked
	2	Ensino fundamental		Sim	0-3	Sim	Unchecked	Checked
	3	Ensino médic	Sim	Sim	0-3	Não	Unchecked	Unchecked
	3	Ensino médic	Sim	Não	0-3	Sim	Checked	Unchecked
	6	Ensino médic	Sim	Sim	4+	Não	Unchecked	Unchecked
	2	Ensino funda	Não sei	Sim	0-3	Não	Unchecked	Unchecked
	2	Ensino funda	Sim	Sim	0-3	Sim	Checked	Unchecked
	3	Ensino funda	Sim	Não	0-3	Sim	Unchecked	Checked
	2	Ensino funda	Recusou a	Não	0-3	Sim	Unchecked	Unchecked
	2	Ensino funda	Não	Sim	0-3	Não	Unchecked	Unchecked
	4	Ensino médic	Sim	Sim	4+	Sim	Checked	Unchecked
	1	Ensino funda	Não sei	Não	0-3	Sim	Unchecked	Checked
	1	Ensino funda	Sim	Não	0-3	Sim	Checked	Checked
nínimos		Ensino funda	Não	Sim	0-3	Não	Unchecked	Unchecked
	3	Ensino funda	Não sei	Sim	0-3	Não	Unchecked	Unchecked
	2	Ensino funda	Sim	Não	0-3	Sim	Checked	Checked
	3	Ensino funda	Não sei	Não	0-3	Sim	Unchecked	Checked
	3	Pós-graduaçã	Sim	Não	0-3	Sim	Unchecked	Checked
	3	Ensino funda	Não sei	Sim	0-3	Sim	Unchecked	Unchecked
	3	Nível superio	Sim	Sim	0-3	Sim	Unchecked	Checked
	4	Ensino funda	Sim	Não	4+	Sim	Unchecked	Checked
	3	Ensino funda	Sim	Sim	0-3	Não	Unchecked	Unchecked
	6	Ensino funda	Sim	Não	4+	Sim	Unchecked	Unchecked
		Ensino funda	Sim	Não	0-3	Não sei	Unchecked	Unchecked
	3	Ensino médic	Sim	Não	0-3	Não sei	Unchecked	Unchecked
	4	Ensino médic	Sim	Sim	0-3	Sim	Checked	Checked
	4	Ensino funda	Não	Sim	0-3	Sim	Checked	Unchecked
	4	Ensino funda	Não	Não	4+	Não sei	Unchecked	Unchecked
	5	Ensino funda	Sim	Não	4+	Sim	Unchecked	Checked
	3	Ensino médic	Sim	Sim	0-3	Sim	Unchecked	Checked
	3	Nenhum	Sim	Não	0-3	Não	Unchecked	Unchecked
	3	Nenhum	Sim	Sim	0-3	Não	Unchecked	Unchecked
	2	Ensino médic	Sim	Sim	0-3	Não	Unchecked	Unchecked
	2	Ensino médic	Sim	Sim	0-3	Sim	Unchecked	Unchecked
	1	Ensino funda	Sim	Sim	0-3	Não	Unchecked	Unchecked

	2	Ensino funda	Sim	Sim	4+	Sim	Unchecked	Checked
	2	Ensino médic	Sim	Sim	0-3	Sim	Unchecked	Unchecked
	1	Ensino funda	Sim	Sim	0-3	Sim	Unchecked	Unchecked
	1	Ensino funda	Sim	Sim	0-3	Sim	Unchecked	Unchecked
	2	Nenhum	Não	Não	0-3	Sim	Unchecked	Unchecked
	2	Nenhum	Não	Não	0-3	Não	Unchecked	Unchecked
	4	Ensino médic	Não	Sim	0-3	Não	Unchecked	Unchecked
	2	Ensino funda	Recusou a	Não	0-3	Recusou a re	Unchecked	Unchecked
	3	Ensino funda	Não	Sim	0-3	Sim	Checked	Unchecked
	2	Ensino funda	Não	Sim	0-3	Não	Unchecked	Unchecked
ínimos		Nenhum	Não	Sim	0-3	Sim	Unchecked	Unchecked
	5	Ensino funda	Não	Não	0-3	Sim	Unchecked	Checked
	2	Ensino funda	Não	Não	0-3	Sim	Checked	Checked
	3	Ensino funda	Não	Sim	0-3	Sim	Checked	Unchecked
	4		Sim	Sim	0-3	Não	Unchecked	Unchecked
	4	Nenhum	Recusou a	Não	4+	Não	Unchecked	Unchecked
	2	Nenhum	Não	Não	0-3	Sim	Unchecked	Unchecked
	5	Ensino médic	Não	Não	4+	Sim	Unchecked	Checked
	4	Ensino médic	Recusou a	Sim	0-3	Sim	Unchecked	Unchecked
	2	Ensino funda	Sim	Sim	0-3	Sim	Checked	Checked
	2	Ensino médic	Sim	Sim	0-3	Não	Unchecked	Unchecked
	3	Ensino funda	Não	Sim	0-3	Não	Unchecked	Unchecked
	2	Ensino funda	Sim	Sim	0-3	Sim	Unchecked	Checked
	2	Ensino funda	Não	Sim	0-3	Sim	Unchecked	Checked
	3	Nenhum	Sim	Sim	0-3	Não	Unchecked	Unchecked
	3	Nenhum	Não	Não	0-3	Não	Unchecked	Unchecked
	4	Ensino funda	Sim	Não	0-3	Não	Unchecked	Unchecked
	2	Nenhum	Sim	Sim	4+	Sim	Unchecked	Unchecked
	4	Ensino funda	Sim	Não	0-3	Não	Unchecked	Unchecked
	2	Ensino funda	Sim	Não	0-3	Sim	Unchecked	Unchecked
	4	Ensino funda	Sim	Sim	4+	Sim	Checked	Unchecked
	4	Ensino funda	Sim	Não	4+	Não	Unchecked	Unchecked
	3	Nenhum	Sim	Sim	0-3	Sim	Checked	Unchecked
	6	Ensino funda	Não	Sim	0-3	Não	Unchecked	Unchecked
	2	Ensino funda	Sim	Sim	0-3	Sim	Checked	Checked
	2	Ensino funda	Não	Sim	0-3	Sim	Unchecked	Checked
sponder		Ensino funda	Não	Não	4+	Sim	Checked	Unchecked
	5	Ensino médic	Recusou a	Sim	0-3	Sim	Checked	Checked
	2	Nenhum	Não	Sim	0-3	Sim	Unchecked	Checked
	2	Nenhum	Não	Não	0-3	Não sei	Unchecked	Unchecked
sponder		Nenhum	Sim	Não	0-3	Não	Unchecked	Unchecked
sponder		Ensino funda	Recusou a	Não	0-3	Sim	Checked	Unchecked
	0	Ensino funda	Sim	Não	0-3	Sim	Unchecked	Unchecked

nínimos		Ensino funda	Sim	Sim	0-3	Sim	Unchecked	Unchecked
		Ensino funda	Sim	Sim	0-3	Sim	Checked	Unchecked
	1	Ensino médio		Sim	0-3	Sim	Checked	Unchecked
	3	Ensino médio		Sim	0-3	Sim	Checked	Unchecked
	1	Nenhum	Não	Sim	0-3	Não	Unchecked	Unchecked
	2	Ensino funda	Sim	Sim	0-3	Sim	Checked	Unchecked
	2	Ensino funda	Não	Sim	0-3	Sim	Checked	Unchecked
	2	Ensino médic	Não		0-3	Sim	Unchecked	Unchecked
	3	Ensino médic	Sim		0-3	Sim	Unchecked	Checked
	4	Ensino funda	Sim	Sim	0-3		Unchecked	Unchecked
	1	Ensino funda	Não	Não	0-3	Não sei	Unchecked	Unchecked
	1	Ensino funda	Sim	Não	0-3	Sim	Unchecked	Checked
		Nenhum		Sim	0-3	Sim	Checked	Unchecked
	1	Nenhum	Sim	Sim	0-3	Não sei	Unchecked	Unchecked
	2	Ensino funda	Não	Sim	0-3	Não sei	Unchecked	Unchecked
	2	Ensino funda	Sim	Não	0-3	Sim	Unchecked	Unchecked
	3	Nenhum	Não	Não	0-3	Não	Unchecked	Unchecked
	2	Ensino funda	Sim	Sim	0-3	Não sei	Unchecked	Unchecked
	2	Ensino funda	Sim	Não	0-3	Não	Unchecked	Unchecked
	1	Ensino médic	Não sei	Não	0-3	Sim	Checked	Checked
	2	Ensino médic	Sim	Não	0-3	Sim	Checked	Unchecked
	1	Ensino funda	Sim	Não	0-3	Não sei	Unchecked	Unchecked
	3	Ensino médic	Sim	Sim	0-3	Sim	Checked	Unchecked
	5	Ensino médic	Sim	Sim	0-3	Não sei	Unchecked	Unchecked
	2	Ensino funda	Não	Não	0-3	Sim	Unchecked	Checked
	2	Ensino funda	Sim	Sim	0-3	Sim	Unchecked	Checked
	1	Ensino funda	Sim	Sim	0-3	Sim	Checked	Unchecked
	4	Ensino funda	Sim	Sim	0-3	Sim	Checked	Unchecked
	2	Ensino funda	Não	Sim	0-3	Sim	Checked	Unchecked
	4	Ensino funda	Não	Não	4+	Não	Unchecked	Unchecked
	2	Ensino funda	Sim	Sim	0-3	Não sei	Unchecked	Unchecked
	2	Ensino funda	Sim	Sim	0-3	Não sei	Unchecked	Unchecked
	1	Ensino funda	Sim	Não	0-3	Não sei	Unchecked	Unchecked
	3	Nenhum	Não	Sim	0-3	Não	Unchecked	Unchecked
	2	Ensino funda	Sim	Sim	0-3	Não	Unchecked	Unchecked
	1	Ensino funda	Não	Não	0-3	Sim	Unchecked	Unchecked
	7	Ensino funda	Sim	Não	0-3	Sim	Checked	Checked
	4	Ensino médic	Sim	Não	0-3	Sim	Checked	Unchecked
	4	Ensino funda	Sim	Sim	0-3	Sim	Checked	Unchecked
	3	Ensino funda	Sim	Sim	0-3	Sim	Checked	Checked
	6	Ensino médic	Sim	Sim	0-3	Sim	Unchecked	Unchecked
	3	Ensino funda	Sim	Sim	0-3	Sim	Checked	Checked
	2	Ensino funda	Sim	Não	0-3	Sim	Checked	Unchecked

6	Ensino funda	Sim	Sim	0-3	Sim	Checked	Unchecked
4	Nenhum	Sim	Não	4+	Sim	Unchecked	Checked
2	Ensino funda	Não sei	Sim	0-3	Não	Unchecked	Unchecked
2	Ensino funda	Sim	Sim	0-3	Sim	Checked	Unchecked
2	Ensino funda	Sim	Sim	4+	Não	Unchecked	Unchecked
1	Ensino funda	Não	Não	0-3	Não	Unchecked	Unchecked
3	Ensino funda	Não sei	Sim	0-3	Não	Unchecked	Unchecked
2	Nenhum	Sim	Sim	0-3	Sim	Unchecked	Checked
4	Ensino médio	Não	Sim	0-3	Não	Unchecked	Unchecked
5	Ensino funda	Sim	Sim	0-3	Não	Unchecked	Unchecked
3	Ensino médio	Sim	Sim	0-3	Não	Unchecked	Unchecked
1	Nível Técnico	Sim	Sim	0-3	Sim	Unchecked	Unchecked
3	Ensino funda	Não	Sim	4+	Sim	Unchecked	Unchecked
1	Ensino médio	Sim	Sim	0-3	Não	Unchecked	Unchecked
2	Ensino funda	Não	Sim	0-3	Não sei	Unchecked	Unchecked
1	Ensino funda	Não	Não	0-3	Sim	Unchecked	Unchecked
3	Ensino funda	Não	Não	0-3	Não	Unchecked	Unchecked
2	Ensino funda	Não sei	Não	4+	Sim	Unchecked	Checked
1	Ensino funda	Não	Não	0-3	Sim	Unchecked	Checked
3	Ensino médio	Não	Não	4+	Não	Unchecked	Unchecked
2	Nenhum	Não	Não	0-3	Não	Unchecked	Unchecked
5	Ensino funda	Sim	Não	4+	Sim	Checked	Unchecked
1	Ensino funda	Não	Sim	0-3	Não	Unchecked	Unchecked
1	Ensino funda	Não	Sim	0-3	Sim	Unchecked	Unchecked
nimo	Ensino funda	Não	Sim	0-3	Sim	Checked	Unchecked
2	Nenhum	Não	Não	0-3	Não sei	Unchecked	Unchecked
3	Ensino funda	Sim	Sim	0-3	Sim	Checked	Unchecked
Não informac	Ensino funda	Sim	Sim	0-3	Sim	Checked	Checked
1	Ensino médio	Sim	Sim	0-3	Sim	Checked	Unchecked
2	Ensino funda	Sim	Sim	0-3	Sim	Checked	Checked
4	Nível superio	Sim	Sim	0-3	Não	Unchecked	Unchecked
4	Ensino médio	Sim	Sim	0-3	Não	Unchecked	Unchecked
2	Ensino médio	Sim	Sim	0-3	Sim	Checked	Checked
1	Ensino funda	Sim	Sim	0-3	Sim	Checked	Checked
2	Ensino funda	Sim	Sim	0-3	Sim	Unchecked	Unchecked
2	Ensino médio	Sim	Sim	0-3	Sim	Checked	Checked
3	Ensino médio	Sim	Sim	0-3	Não sei	Unchecked	Unchecked
2	Ensino funda	Não	Sim	0-3	Sim	Unchecked	Unchecked
2	Ensino funda	Sim	Não	0-3	Sim	Unchecked	Unchecked
1	Ensino funda	Não	Sim	0-3	Não	Unchecked	Unchecked
1	Nenhum	Sim	Não	0-3	Não	Unchecked	Unchecked
	Ensino funda	Não	Não	0-3	Sim	Checked	Unchecked
1	Ensino funda	Sim	Não	0-3	Sim	Unchecked	Unchecked

	2	Nenhum	Não	Não	0-3	Sim	Unchecked	Unchecked
	4	Ensino médic	Sim	Sim	4+	Não	Unchecked	Unchecked
	1	Ensino funda	Não	Não	0-3	Sim	Unchecked	Checked
	5	Ensino médic	Sim	Não	0-3	Não	Unchecked	Unchecked
nínimos		Ensino médic	Sim	Sim	0-3	Sim	Checked	Checked
	1	Nenhum	Não	Não	0-3	Sim	Checked	Unchecked
	2	Ensino médic	Não	Sim	0-3	Sim	Checked	Unchecked
	2	Ensino funda	Sim	Sim	0-3	Sim	Unchecked	Unchecked
	3	Ensino médic	Sim	Sim	0-3	Sim	Unchecked	Unchecked
	3	Ensino funda	Sim	Sim	0-3	Sim	Unchecked	Checked
	4	Ensino funda	Sim	Sim	0-3	Não sei	Unchecked	Unchecked
	3	Ensino funda	Não	Sim	4+	Sim	Checked	Unchecked
	2	Ensino funda	Não	Não	0-3	Não	Unchecked	Unchecked
	4	Ensino funda	Sim	Não	0-3	Não	Unchecked	Unchecked
	4	Ensino funda	Sim	Não	0-3	Sim	Unchecked	Unchecked
	4	Ensino funda	Sim	Sim	0-3	Sim	Checked	Unchecked
	4	Ensino funda	Não	Não	4+	Sim	Unchecked	Checked
	4	Ensino funda	Sim	Sim	4+	Não	Unchecked	Unchecked
	3	Nenhum	Não	Não	0-3	Sim	Checked	Unchecked
	3	Ensino funda	Sim	Sim	0-3	Não	Unchecked	Unchecked
	5	Ensino funda	Sim	Sim	4+	Não	Unchecked	Unchecked
	2	Ensino funda	Não	Sim	0-3	Não	Unchecked	Unchecked
	1	Ensino médic	Sim	Não	0-3	Não	Unchecked	Unchecked
	4	Ensino funda	Não	Sim	4+	Não	Unchecked	Unchecked
	2	Ensino funda	Não	Sim	4+	Sim	Unchecked	Unchecked
	1	Ensino funda	Sim	Não	0-3	Sim	Checked	Unchecked
	3	Nenhum	Não	Sim	0-3	Sim	Unchecked	Checked
	4	Ensino médic	Sim	Sim	0-3	Sim	Checked	Unchecked
	2	Ensino funda	Não	Sim	0-3	Não	Unchecked	Unchecked
	2	Ensino funda	Não	Sim	0-3	Sim	Unchecked	Checked
	3	Ensino médic	Sim	Não	4+	Não	Unchecked	Unchecked
	1	Ensino funda	Sim		4+	Não sei	Unchecked	Unchecked
	4	Ensino médic	Não	Sim	4+	Sim	Unchecked	Checked
	4	Ensino funda	Sim	Sim	0-3	Não	Unchecked	Unchecked
	3	Ensino funda	Não	Sim	0-3	Sim	Checked	Checked
		Ensino funda	Sim	Sim	0-3	Não	Unchecked	Unchecked
	4	Ensino médic	Sim	Não	0-3	Não sei	Unchecked	Unchecked
	4	Ensino funda	Sim	Sim	0-3	Sim	Checked	Unchecked
	4	Ensino médic	Sim	Não	4+	Sim	Checked	Unchecked
	1	Ensino funda	Não	Sim	0-3	Sim	Unchecked	Checked
	2	Ensino funda	Sim	Sim	0-3	Sim	Checked	Unchecked
nínimos		Ensino funda	Sim	Sim	0-3	Sim	Unchecked	Checked
	2	Ensino médic	Sim	Não	0-3	Sim	Checked	Unchecked

2	Ensino médic	Não	Não	0-3	Sim	Checked	Checked
5	Ensino médic	Sim	Sim	0-3	Não	Unchecked	Unchecked
4	Ensino médic	Sim	Não	4+	Sim	Checked	Unchecked
2	Ensino funda	Sim	Sim	0-3	Não sei	Unchecked	Unchecked
4	Nenhum	Sim	Sim	0-3	Sim	Checked	Unchecked
2	Ensino funda	Sim	Não	0-3	Não	Unchecked	Unchecked
4	Ensino funda	Sim	Sim	0-3	Não	Unchecked	Unchecked
2	Ensino médic	Sim	Não	0-3	Sim	Checked	Unchecked
3	Ensino funda	Sim	Sim	0-3	Sim	Unchecked	Checked
4	Ensino médic	Sim	Sim	0-3	Sim	Checked	Unchecked
3	Nível Técnico	Sim	Não	0-3	Não	Unchecked	Unchecked
2	Ensino médic	Sim	Sim	0-3	Sim	Checked	Unchecked
4	Ensino funda	Sim	Não	0-3	Sim	Checked	Unchecked
4	Ensino médic	Sim	Sim	0-3	Sim	Checked	Checked
4	Ensino funda	Sim	Sim	0-3	Sim	Unchecked	Checked
3	Ensino funda	Sim	Sim	0-3	Sim	Unchecked	Unchecked
2	Nenhum	Não	Sim	0-3	Não sei	Unchecked	Unchecked
4	Ensino funda	Sim	Sim	0-3	Sim	Unchecked	Checked
2	Ensino funda	Sim	Sim	0-3	Não sei	Unchecked	Unchecked
6	Ensino funda	Sim	Não	4+	Sim	Unchecked	Checked
4	Ensino funda	Sim	Sim	4+	Não	Unchecked	Unchecked
4	Ensino funda	Não	Sim	4+	Sim	Unchecked	Checked
3	Nível superio	Sim	Sim	0-3	Sim	Checked	Unchecked
1	Ensino médic	Sim	Sim	0-3	Não	Unchecked	Unchecked
1	Ensino funda	Sim	Não	0-3	Sim	Checked	Unchecked
1	Nível superio	Sim	Não	0-3	Sim	Unchecked	Checked
4	Ensino funda	Sim	Sim	0-3	Sim	Unchecked	Checked
1	Nenhum	Sim	Sim	0-3	Sim	Unchecked	Checked
4	Ensino funda	Sim	Não	4+	Sim	Unchecked	Checked
3	Pós-graduaçã	Sim	Sim	0-3	Sim	Unchecked	Checked
2	Nível Técnico	Sim	Sim	0-3	Sim	Unchecked	Unchecked
3	Nível superio	Sim	Sim	0-3	Sim	Unchecked	Unchecked
4	Ensino médic	Sim	Não	4+	Sim	Unchecked	Checked
4	Ensino médic	Sim	Não	4+	Não sei	Unchecked	Unchecked
3	Ensino funda	Sim	Sim	0-3	Sim	Unchecked	Checked
4	Ensino funda	Sim	Não	0-3	Sim	Unchecked	Checked
4	Ensino médic	Sim	Não	0-3	Sim	Unchecked	Unchecked
4	Ensino funda	Não	Não	0-3	Sim	Checked	Checked
2	Ensino funda	Não	Não	0-3	Sim	Unchecked	Unchecked
3	Nenhum	Sim	Sim	0-3	Sim	Unchecked	Unchecked
3	Nenhum	Sim	Sim	0-3	Sim	Unchecked	Unchecked
5	Ensino funda	Sim	Não	0-3	Não sei	Unchecked	Unchecked
2	Nenhum	Não	Não	0-3	Sim	Checked	Unchecked

	2	Ensino médic	Sim	Sim	0-3	Não	Unchecked	Unchecked
	3	Ensino médic	Sim	Não	0-3	Não	Unchecked	Unchecked
	2	Ensino funda	Não	Não	0-3	Não	Unchecked	Unchecked
	1	Ensino médic	Sim	Sim	0-3	Sim	Unchecked	Unchecked
	2	Ensino médic	Sim	Não	0-3	Não	Unchecked	Unchecked
	2	Ensino médic	Sim	Sim	0-3	Sim	Unchecked	Unchecked
	1	Nenhum	Não	Não	0-3	Não sei	Unchecked	Unchecked
	4	Ensino funda	Sim	Sim	0-3	Sim	Unchecked	Unchecked
		Ensino funda	Sim	Não	0-3	Não sei	Unchecked	Unchecked
	4	Ensino funda	Sim	Sim	0-3	Não	Unchecked	Unchecked
		Ensino funda	Sim	Sim	0-3	Não	Unchecked	Unchecked
	5	Ensino funda	Não	Sim	4+	Sim	Checked	Unchecked
	2	Ensino médic	Sim	Sim	0-3	Não	Unchecked	Unchecked
	4	Nenhum	Sim	Sim	0-3	Sim	Checked	Unchecked
		Nenhum	Sim	Sim	4+	Não	Unchecked	Unchecked
	2	Ensino funda	Sim	Sim	0-3	Não	Unchecked	Unchecked
	2	Ensino funda	Sim	Não	0-3	Não	Unchecked	Unchecked
	3	Ensino médic	Sim	Sim	0-3		Unchecked	Unchecked
	1	Nenhum	Sim	Não	0-3	Não	Unchecked	Unchecked
	5	Ensino funda	Sim	Sim	4+	Sim	Checked	Unchecked
	6	Ensino funda	Sim	Sim	4+	Não	Unchecked	Unchecked
	1	Ensino médic	Sim	Não	0-3	Sim	Unchecked	Checked
	1	Ensino funda	Não	Não	0-3	Sim	Checked	Unchecked
	2	Ensino funda	Não	Sim	0-3	Sim	Checked	Unchecked
	1	Ensino funda	Não	Não	0-3	Sim	Checked	Unchecked
	1	Nenhum	Não	Não	0-3	Não	Unchecked	Unchecked
nínimos		Nível Técnico	Sim	Não	0-3	Não	Unchecked	Unchecked
ínimos		Pós-graduaçã	Sim	Sim	0-3	Não	Unchecked	Unchecked
	1	Ensino médic	Sim	Não	0-3	Não	Unchecked	Unchecked
	2	Ensino médic	Não	Não	0-3	Não	Unchecked	Unchecked
	5	Nenhum	Não	Sim	0-3	Sim	Unchecked	Checked
	4	Ensino funda	Sim	Sim	0-3	Sim	Checked	Checked
	2	Ensino funda	Não	Não	0-3	Sim	Checked	Unchecked
	3	Nenhum	Sim	Sim	0-3	Não	Unchecked	Unchecked
	4	Ensino funda	Sim	Não	0-3	Não	Unchecked	Unchecked
nínimos		Nenhum	Não	Sim	0-3	Sim	Checked	Checked
	2	Ensino funda	Não	Sim	0-3	Sim	Checked	Checked
	3	Ensino funda	Não	Não	0-3	Sim	Checked	Unchecked
	3		Sim	Sim	0-3	Não sei	Unchecked	Unchecked
	4	Ensino funda	Sim	Sim	0-3	Sim	Unchecked	Unchecked
	4	Nenhum	Sim	Não	0-3	Sim	Checked	Unchecked
	5	Ensino funda	Sim	Sim	0-3	Sim	Checked	Unchecked
	4	Ensino funda	Sim	Sim	4+	Sim	Checked	Unchecked

1	Nenhum	Sim	Não	0-3	Não	Unchecked	Unchecked
7	Ensino funda	Sim	Sim	4+	Não sei	Unchecked	Unchecked
4	Ensino funda	Sim	Sim	0-3	Sim	Unchecked	Checked
1	Nenhum	Não	Não	0-3	Sim	Unchecked	Unchecked
6	Ensino funda	Sim	Sim	4+	Não	Unchecked	Unchecked
3	Ensino funda	Sim	Sim	0-3	Não	Unchecked	Unchecked
2	Nenhum	Sim	Não	0-3	Não sei	Unchecked	Unchecked
2	Ensino médic	Sim	Não	0-3	Sim	Checked	Checked
5	Ensino funda	Sim	Não	4+	Não	Unchecked	Unchecked
4	Ensino médic	Sim	Não	0-3	Sim	Checked	Unchecked
2	Nenhum	Não	Sim	0-3	Sim	Unchecked	Unchecked
	Ensino funda	Sim	Sim	0-3	Sim	Unchecked	Checked
2	Ensino médic	Sim	Sim	0-3	Sim	Unchecked	Checked
2	Ensino funda	Sim	Sim	0-3	Sim	Unchecked	Checked
4	Ensino funda	Não	Sim	4+	Sim	Checked	Unchecked
2	Ensino funda	Não	Sim	0-3	Sim	Checked	Unchecked
2	Ensino funda	Não	Sim	0-3	Não	Unchecked	Unchecked
7	Ensino médic	Sim	Sim	0-3	Sim	Unchecked	Checked
3	Nenhum	Não	Não	4+	Sim	Unchecked	Checked
3	Ensino médic	Sim	Sim	0-3	Sim	Checked	Unchecked
2	Ensino médic	Sim	Não	0-3	Sim	Unchecked	Unchecked
3	Ensino funda	Sim	Não	0-3	Sim	Checked	Unchecked
3	Ensino médic	Não	Não	0-3	Sim	Checked	Unchecked
2	Ensino médic	Sim	Não	0-3	Não sei	Unchecked	Unchecked
1	Nenhum	Sim	Não	0-3	Sim	Unchecked	Checked
2	Ensino médic	Sim	Sim	0-3	Sim	Unchecked	Unchecked
2	Ensino funda	Sim	Sim	0-3	Não	Unchecked	Unchecked
3	Ensino médic	Sim	Sim	0-3	Não	Unchecked	Unchecked
3	Ensino funda	Sim	Sim	0-3	Sim	Checked	Unchecked
5	Ensino funda	Sim	Sim	4+	Sim	Checked	Unchecked
3	Ensino funda	Sim	Sim	0-3	Sim	Unchecked	Unchecked
3	Ensino funda	Sim	Não	0-3	Sim	Checked	Checked
1	Ensino funda	Sim	Não	0-3	Não sei	Unchecked	Unchecked
2	Ensino funda	Sim	Sim	0-3	Não	Unchecked	Unchecked
2	Ensino médic	Sim	Sim	0-3	Sim	Checked	Unchecked
4	Ensino funda	Sim	Não	4+	Não	Unchecked	Unchecked
2	Ensino funda	Sim	Sim	0-3	Sim	Unchecked	Checked
3	Ensino funda	Sim	Sim	0-3	Sim	Checked	Unchecked
2	Nenhum	Sim	Não	0-3	Não	Unchecked	Unchecked
2	Ensino funda	Sim	Não	0-3	Sim	Checked	Unchecked
1	Ensino funda	Sim	Sim	0-3	Sim	Unchecked	Unchecked
2	Nenhum	Recusou a	Sim	0-3	Sim	Checked	Checked
4	Ensino funda	Sim	Sim	0-3	Não	Unchecked	Unchecked

1	Ensino funda	Não	Não	0-3	Sim	Unchecked	Checked
2	Ensino funda	Não	Sim		Sim	Unchecked	Checked
6	Ensino funda	Não	Não	0-3	Sim	Checked	Checked
2	Ensino funda	Não	Sim	0-3	Sim	Checked	Checked
2	Nenhum	Sim	Sim	0-3	Não	Unchecked	Unchecked
3	Nenhum	Sim	Sim	0-3	Sim	Checked	Unchecked
4	Nenhum	Sim	Não	0-3	Não	Unchecked	Unchecked
4	Recusou a re	Não	Sim	0-3	Sim	Checked	Checked
1	Nenhum	Não	Não	0-3	Não sei	Unchecked	Unchecked
2	Ensino funda	Sim	Não	0-3	Sim	Unchecked	Checked
1	Ensino funda	Não	Não	0-3	Sim	Unchecked	Unchecked
1	Ensino funda	Sim	Não	0-3	Não	Unchecked	Unchecked
6	Ensino médic	Sim	Sim	0-3	Sim	Unchecked	Unchecked
3	Ensino funda	Sim	Não	0-3	Sim	Checked	Checked
7	Nenhum	Não	Recusou a re	0-3	Sim	Checked	Unchecked
5	Ensino funda	Sim	Sim	4+	Sim	Unchecked	Unchecked
3	Ensino funda	Sim	Sim	4+	Não	Unchecked	Unchecked
3	Ensino funda	Sim	Sim	4+	Sim	Checked	Unchecked
3	Ensino funda	Não	Não	0-3	Não	Unchecked	Unchecked
1	Ensino médic	Sim	Não	0-3	Sim	Checked	Unchecked
3	Ensino funda	Sim	Sim	0-3	Sim	Checked	Unchecked
2	Ensino funda	Não	Não	0-3	Sim	Checked	Unchecked
3	Ensino funda	Sim	Não	0-3	Sim	Checked	Unchecked
2	Nenhum	Não	Não	0-3	Sim	Checked	Unchecked
3	Ensino funda	Sim	Sim	0-3	Sim	Checked	Unchecked
3	Ensino funda	Sim	Não	4+	Sim	Unchecked	Unchecked
4	Nenhum	Sim	Não	0-3	Sim	Checked	Checked
3	Ensino médic	Sim	Sim	0-3	Não	Unchecked	Unchecked
nínimos		Não sei	Não	4+	Não	Unchecked	Unchecked
3	Ensino funda	Sim	Sim	0-3	Não	Unchecked	Unchecked
2	Ensino funda	Sim	Sim	0-3	Sim	Checked	Checked
2	Ensino funda	Sim	Sim	0-3	Sim	Checked	Unchecked
4	Ensino funda	Sim	Sim	0-3	Sim	Unchecked	Unchecked
3	Ensino funda	Sim	Sim	0-3	Não	Unchecked	Unchecked
4	Ensino funda	Recusou a	Sim	4+	Sim	Checked	Unchecked
3	Ensino funda	Sim	Sim	0-3	Sim	Checked	Unchecked
3	Nenhum	Sim	Não	0-3	Não	Unchecked	Unchecked
1	Nenhum	Não	Não	0-3	Sim	Checked	Checked
2	Ensino funda	Sim	Sim	0-3	Sim	Unchecked	Unchecked
1	Nenhum	Não	Sim	0-3	Sim	Checked	Unchecked
3	Ensino funda	Sim	Sim	4+	Sim	Unchecked	Checked
4	Ensino funda	Sim	Não	4+	Não	Unchecked	Unchecked
4	Ensino médic	Sim	Sim	4+	Sim	Checked	Unchecked

	2	Ensino funda	Sim	Sim	0-3	Sim	Checked	Checked
	4	Ensino funda	Sim	Não	0-3	Sim	Unchecked	Unchecked
	4	Ensino médic	Sim	Sim	0-3	Sim	Unchecked	Unchecked
	4	Ensino médic	Sim	Sim	0-3	Sim	Unchecked	Unchecked
sponder		Ensino funda	Sim	Sim	0-3	Sim	Checked	Checked
	2	Ensino médic	Sim	Sim	0-3	Sim	Checked	Checked
	2	Ensino funda	Sim	Sim	0-3	Não	Unchecked	Unchecked
	4	Ensino funda	Não	Sim	0-3	Sim	Checked	Unchecked
	3	Ensino funda	Sim	Não	0-3	Sim	Unchecked	Unchecked
	4	Ensino médic	Sim	Sim	4+	Não sei	Unchecked	Unchecked
	4	Nível superio	Sim	Não	0-3	Sim	Checked	Unchecked
	4	Ensino funda	Sim	Não	0-3	Sim	Checked	Unchecked
	4	Ensino médic	Sim	Não	0-3	Sim	Checked	Unchecked
	7	Ensino médic	Sim	Não	0-3	Não	Unchecked	Unchecked
	7	Ensino funda	Sim	Não	0-3	Não	Unchecked	Unchecked
	3	Ensino fundamental		Sim	0-3	Não	Unchecked	Unchecked
	3	Ensino médic	Não sei	Sim	0-3	Sim	Checked	Unchecked
	4	Ensino funda	Sim	Sim	0-3	Sim	Unchecked	Unchecked
	4	Ensino funda	Sim	Não	0-3	Sim	Unchecked	Unchecked
	4	Nenhum	Sim	Não	0-3	Sim	Unchecked	Unchecked
	3	Nenhum	Não	Sim	0-3	Não	Unchecked	Unchecked
	3	Ensino funda	Não	Não	0-3	Sim	Unchecked	Checked
	2	Ensino funda	Não	Sim	0-3	Não	Unchecked	Unchecked
	3	Nenhum	Sim	Sim	0-3	Sim	Unchecked	Unchecked
	4	Ensino médic	Sim	Não	0-3	Não	Unchecked	Unchecked
	3	Ensino funda	Não sei	Não	0-3	Sim	Unchecked	Checked
	2	Nenhum	Sim	Sim	0-3	Sim	Checked	Checked
	1	Ensino funda	Sim	Não	0-3	Sim	Unchecked	Checked
	2	Nenhum	Não	Sim	0-3	Sim	Checked	Checked
	3	Ensino médic	Sim	Não	0-3	Sim	Unchecked	Unchecked
	3	Nenhum	Não	Não	0-3	Não	Unchecked	Unchecked
	3	Ensino funda	Sim	Sim	0-3	Sim	Unchecked	Checked
	3	Nenhum	Não	Sim	0-3	Sim	Checked	Checked
	4	Ensino funda	Não	Sim	0-3	Sim	Unchecked	Unchecked
	3	Ensino funda	Sim	Sim	0-3	Sim	Unchecked	Checked
	3	Nenhum	Sim	Não	0-3	Sim	Checked	Checked
	4	Ensino funda	Sim	Sim	0-3	Sim	Unchecked	Checked
	2	Ensino funda	Sim	Não	Não sei	Sim	Checked	Unchecked
	2	Ensino funda	Sim	Não	0-3	Sim	Checked	Unchecked
	2	Ensino funda	Sim	Não	0-3	Não sei	Unchecked	Unchecked
	2	Ensino médic	Não	Sim	0-3	Sim	Unchecked	Unchecked
	4	Ensino funda	Sim	Não	0-3	Sim	Unchecked	Checked
	4	Ensino médic	Sim	Sim	0-3	Sim	Checked	Unchecked

	2	Ensino médic	Sim	Sim	0-3	Sim	Checked	Checked
	5	Ensino funda	Sim	Não	0-3	Não	Unchecked	Unchecked
	5	Ensino médic	Sim	Não	0-3	Não	Unchecked	Unchecked
	1	Nenhum	Não	Não	0-3	Sim	Checked	Unchecked
	5	Ensino funda	Sim	Não	0-3	Não	Unchecked	Unchecked
	2	Nível superio	Sim	Não	0-3	Sim	Unchecked	Unchecked
	2	Nível superio	Sim	Sim	0-3	Sim	Unchecked	Checked
	1	Nenhum	Sim	Não	0-3	Sim	Checked	Unchecked
	2	Ensino funda	Sim	Não	0-3	Sim	Checked	Unchecked
	2	Ensino médic	Sim	Não	0-3	Não sei	Unchecked	Unchecked
	2	Nível superio	Sim	Não	0-3	Sim	Checked	Unchecked
	2	Ensino médic	Sim	Sim	0-3	Sim	Unchecked	Unchecked
	2	Nenhum	Não	Sim	0-3	Não sei	Unchecked	Unchecked
	1	Nível superio	Sim	Não	0-3	Sim	Unchecked	Unchecked
	5	Nenhum	Não	Sim	0-3	Sim	Unchecked	Checked
	2	Ensino médic	Sim	Sim	0-3	Sim	Unchecked	Unchecked
	5	Ensino médic	Sim	Não	0-3	Sim	Checked	Checked
	5	Nenhum	Não	Não	0-3	Sim	Unchecked	Checked
	4		Sim	Não	4+	Não	Unchecked	Unchecked
	4	Ensino funda	Não	Sim	0-3	Sim	Unchecked	Checked
	3	Ensino funda	Não	Não	0-3	Sim	Checked	Checked
	2	Ensino funda	Sim	Não	0-3	Sim	Checked	Unchecked
	2	Ensino funda	Sim	Não	0-3	Sim	Unchecked	Unchecked
	3	Nenhum		Sim	0-3	Sim	Checked	Unchecked
	3	Ensino funda	Sim	Sim	0-3	Sim	Unchecked	Unchecked
	1	Ensino funda	Sim	Não	0-3	Sim	Unchecked	Checked
	3	Nenhum	Sim	Sim	4+	Sim	Unchecked	Checked
	2	Ensino funda	Não	Sim	0-3	Não sei	Unchecked	Unchecked
	2	Nenhum	Sim	Sim	0-3	Sim	Unchecked	Unchecked
	1	Nenhum	Sim	Não	0-3	Sim	Checked	Unchecked
	2	Ensino funda	Sim	Sim	0-3	Não	Unchecked	Unchecked
	4	Ensino funda	Não	Não	0-3	Sim	Checked	Checked
	3	Nenhum	Sim	Não	0-3	Sim	Checked	Unchecked
nimo		Ensino funda	Sim	Não	0-3	Sim	Checked	Checked
	5	Ensino médic	Sim	Sim	0-3	Sim	Unchecked	Unchecked
	2	Ensino médic	Sim	Não	0-3	Sim	Unchecked	Unchecked
nínimos		Ensino funda	Sim	Sim	0-3	Não sei	Unchecked	Unchecked
	6	Ensino funda	Sim	Sim	4+	Sim	Checked	Checked
	4		Sim	Sim	4+	Não	Unchecked	Unchecked
	1	Nenhum	Não	Não	0-3	Sim	Checked	Unchecked
	3	Ensino funda	Sim	Não	0-3	Sim	Checked	Unchecked
	1	Ensino funda	Não	Não	0-3	Sim	Unchecked	Unchecked
	3	Ensino médic	Sim	Sim	0-3	Sim	Unchecked	Unchecked

2	Ensino funda	Sim	Sim	0-3	Não sei	Unchecked	Unchecked
2	Ensino funda	Sim	Sim	0-3	Sim	Unchecked	Unchecked
3	Ensino médic	Não sei	Sim	0-3	Sim	Unchecked	Unchecked
3	Ensino médic	Não sei	Não	0-3	Não	Unchecked	Unchecked
4	Ensino médic	Sim	Sim	4+	Sim	Unchecked	Unchecked
4	Ensino médic	Sim	Não	4+	Sim	Unchecked	Unchecked
4	Ensino funda	Sim	Não	4+	Não	Unchecked	Unchecked
3	Ensino funda	Não	Não	0-3	Não	Unchecked	Unchecked
3	Ensino funda	Sim	Sim	0-3	Não	Unchecked	Unchecked
2	Nenhum	Não	Sim	0-3	Sim	Unchecked	Checked
3	Ensino médic	Não	Não	0-3	Sim	Unchecked	Checked
1	Ensino funda	Sim	Sim	0-3	Sim	Checked	Unchecked
2	Nível superio	Sim	Sim	0-3	Sim	Checked	Checked
4	Nenhum	Não	Não	0-3	Não	Unchecked	Unchecked
2	Pós-graduaçã	Não	Sim	0-3	Sim	Checked	Checked
2	Nível superio	Sim	Não	0-3	Sim	Checked	Checked
3	Ensino médic	Sim	Não	0-3	Sim	Checked	Checked
5	Nenhum	Sim	Não	0-3	Sim	Checked	Unchecked
2	Ensino funda	Não	Não	0-3	Sim	Checked	Checked
3	Nível superio	Sim	Sim	0-3	Sim	Checked	Checked
3	Nível Técnicc	Sim	Não	0-3	Sim	Unchecked	Checked
4	Ensino funda	Sim	Sim	0-3	Sim	Checked	Unchecked
2	Ensino funda	Sim	Sim	0-3	Sim	Checked	Unchecked
3	Ensino funda	Não	Sim	0-3	Sim	Checked	Checked
3	Ensino funda	Sim	Não	0-3	Sim	Checked	Unchecked
2	Ensino funda	Sim	Não	0-3	Sim	Unchecked	Checked
4	Ensino funda	Sim	Não	0-3	Sim	Unchecked	Checked
4	Ensino funda	Sim	Sim	0-3	Sim	Checked	Unchecked
2	Ensino funda	Não	Sim	0-3	Sim	Unchecked	Checked
2	Ensino funda	Não	Sim	0-3	Sim	Unchecked	Checked
4	Ensino funda	Sim	Sim	0-3	Não sei	Unchecked	Unchecked
4	Ensino funda	Sim	Sim	0-3	Sim	Unchecked	Checked
2	Nenhum	Não	Sim	0-3	Sim	Checked	Unchecked
2	Nenhum	Sim	Sim	0-3	Sim	Checked	Checked
4	Nenhum	Sim	Sim	0-3	Sim	Unchecked	Checked
	Não sei	Sim	Não	0-3	Sim	Checked	Checked
4	Nenhum	Sim	Sim	0-3	Sim	Unchecked	Checked
2	Nenhum	Sim	Sim	0-3	Sim	Checked	Unchecked
4	Nenhum	Sim	Sim	0-3	Sim	Unchecked	Checked
4	Nenhum	Sim	Sim	0-3	Sim	Unchecked	Checked
2	Ensino funda	Não	Sim	0-3	Sim	Checked	Unchecked
4	Ensino funda	Sim	Sim	0-3	Não	Unchecked	Unchecked
5	Ensino funda	Sim	Sim	0-3	Sim	Checked	Checked

3	Ensino funda	Sim	Sim	0-3	Sim	Checked	Checked
2	Nenhum	Não	Sim	0-3	Sim	Checked	Unchecked
2	Nenhum	Sim	Sim	0-3	Sim	Checked	Unchecked
5	Ensino funda	Sim	Sim	0-3	Sim	Checked	Unchecked
1	Nenhum	Não	Não	0-3	Sim	Checked	Checked
2	Nenhum	Sim	Não	0-3	Sim	Checked	Checked
2	Ensino funda	Sim	Sim	0-3	Sim	Unchecked	Unchecked
2	Ensino funda	Sim	Sim	0-3	Sim	Checked	Checked
2	Ensino funda	Não	Não	0-3	Não	Unchecked	Unchecked
3	Nível superio	Sim	Não	0-3	Sim	Unchecked	Unchecked
3	Ensino funda	Sim	Sim	0-3	Não	Unchecked	Unchecked
3	Ensino funda	Não	Sim	0-3	Sim	Unchecked	Unchecked
2	Ensino médic	Sim	Sim	4+	Sim	Checked	Checked
2	Ensino funda	Não	Sim	0-3	Sim	Checked	Unchecked
1	Ensino médic	Sim	Não	0-3	Sim	Checked	Checked
4	Nível superio	Sim	Sim	0-3	Sim	Checked	Checked
1	Nenhum	Sim	Não	0-3	Sim	Unchecked	Unchecked
4	Ensino funda	Não	Sim	0-3	Não	Unchecked	Unchecked
4	Ensino funda	Sim	Sim	0-3	Não	Unchecked	Unchecked
4	Ensino médic	Sim	Sim	4+	Sim	Checked	Unchecked
4	Ensino médic	Sim	Sim	0-3	Sim	Unchecked	Unchecked
5	Ensino médic	Sim	Sim	0-3	Sim	Unchecked	Unchecked
5	Ensino funda	Sim	Não	0-3	Não	Unchecked	Unchecked
3	Ensino funda	Sim	Não	0-3	Sim	Unchecked	Checked
6	Ensino funda	Não	Sim	4+	Sim	Unchecked	Unchecked
6	Ensino funda	Sim	Não	4+	Sim	Unchecked	Unchecked
3	Ensino funda	Sim	Não	0-3	Sim	Checked	Unchecked
7	Ensino médic	Sim	Não	0-3	Sim	Unchecked	Unchecked
4	Ensino funda	Sim	Não	0-3	Não	Unchecked	Unchecked
4	Ensino médic	Sim	Sim	0-3	Sim	Checked	Checked
5	Ensino médic	Sim	Sim	0-3	Sim	Unchecked	Unchecked
4	Ensino médic	Sim	Sim	4+	Sim	Unchecked	Checked
5	Ensino funda	Sim	Sim	0-3	Sim	Unchecked	Unchecked
5	Ensino funda	Sim	Sim	0-3	Não	Unchecked	Unchecked
5	Ensino funda	Sim	Sim	0-3	Não	Unchecked	Unchecked
4	Ensino funda	Não	Não	0-3	Não	Unchecked	Unchecked
2	Ensino funda	Sim	Não	0-3	Não	Unchecked	Unchecked
3	Ensino funda	Sim	Sim	0-3	Sim	Unchecked	Unchecked
3	Ensino funda	Sim	Não	0-3	Não	Unchecked	Unchecked
3	Ensino médic	Sim	Sim	0-3	Não	Unchecked	Unchecked
3	Ensino funda	Sim	Não	0-3	Sim	Unchecked	Unchecked
6	Ensino médic	Sim	Sim	4+	Sim	Checked	Unchecked
5	Ensino funda	Sim	Sim	0-3	Não	Unchecked	Unchecked

	2	Ensino funda	Não	Sim	0-3	Sim	Unchecked	Checked
	2	Ensino funda	Não	Sim	0-3	Sim	Unchecked	Checked
	5	Ensino funda	Sim	Sim	4+	Não	Unchecked	Unchecked
	4	Nenhum	Sim	Sim	4+	Não	Unchecked	Unchecked
nimo			Sim	Sim	4+	Não	Unchecked	Unchecked
	5	Ensino funda	Sim	Não	0-3	Não	Unchecked	Unchecked
	5	Ensino funda	Sim	Não	0-3	Não	Unchecked	Unchecked
	4	Ensino médic	Sim	Sim	0-3	Sim	Unchecked	Unchecked
	5	Ensino funda	Sim	Não	0-3	Não	Unchecked	Unchecked
	4	Ensino funda	Sim	Não	0-3	Sim	Unchecked	Unchecked
	4	Ensino funda	Sim	Não	0-3	Sim	Unchecked	Unchecked
	5	Nenhum	Sim	Não	4+	Não	Unchecked	Unchecked
	2	Ensino funda	Sim	Não	0-3	Sim	Unchecked	Unchecked
	2	Ensino funda	Sim	Sim	0-3	Sim	Checked	Unchecked
	2	Ensino médic	Sim	Sim	0-3	Não	Unchecked	Unchecked
	2	Ensino médic	Sim	Não	0-3	Sim	Unchecked	Unchecked
	2	Ensino funda	Não	Sim	0-3	Sim	Unchecked	Checked
	6	Ensino médic	Sim	Não	0-3	Sim	Unchecked	Checked
	1	Ensino funda	Não	Não	0-3	Sim	Unchecked	Checked
	4	Pós-graduaçã	Sim	Não	4+	Sim	Unchecked	Unchecked
	4	Ensino funda	Sim	Não	4+	Sim	Unchecked	Unchecked
	2	Ensino médic	Sim	Sim	0-3	Sim	Checked	Unchecked
	3	Ensino funda	Sim	Sim	0-3	Não	Unchecked	Unchecked
	2	Ensino funda	Sim	Sim	0-3	Sim	Checked	Checked
	4	Ensino funda	Sim	Não	0-3	Sim	Unchecked	Unchecked
	2	Nenhum	Não	Sim	0-3	Sim	Unchecked	Checked
	1	Ensino funda	Não	Não	0-3	Sim	Unchecked	Checked
	1	Ensino funda	Sim	Não	0-3	Sim	Checked	Unchecked
	2	Ensino funda	Sim	Não	0-3	Sim	Checked	Unchecked
	5	Ensino médic	Sim	Sim	0-3	Não	Unchecked	Unchecked
		Ensino funda	Sim	Não	4+	Não	Unchecked	Unchecked
	2	Ensino funda	Sim	Não	0-3	Não sei	Unchecked	Unchecked
	2	Ensino funda	Sim	Sim	0-3	Sim	Unchecked	Checked
	2	Ensino funda	Sim	Não	0-3	Sim	Checked	Checked
	2	Ensino funda	Não	Sim	0-3	Não	Unchecked	Unchecked
	5	Ensino funda	Sim	Sim	4+	Não	Unchecked	Unchecked
	3	Ensino funda	Recusou a	Sim	0-3	Não	Unchecked	Unchecked
	4	Ensino funda	Sim	Sim	4+	Não	Unchecked	Unchecked
			Sim	Não	0-3	Não	Unchecked	Unchecked
	2	Ensino funda	Não	Não	0-3	Não	Unchecked	Unchecked
	2	Ensino funda	Sim	Não	0-3	Sim	Unchecked	Unchecked
	3	Ensino funda	Não	Não	0-3	Não	Unchecked	Unchecked
	2	Ensino funda	Sim	Não	0-3	Não	Unchecked	Unchecked

	2	Ensino funda	Não	Sim	0-3	Sim	Checked	Checked
	2	Ensino funda	Não	Não	0-3	Sim	Checked	Unchecked
	4	Ensino funda	Sim	Sim	0-3	Sim	Checked	Unchecked
	7	Ensino funda	Não	Não	0-3	Não	Unchecked	Unchecked
	2	Ensino funda	Não	Sim	0-3	Não	Unchecked	Unchecked
	5	Ensino funda	Não	Sim	4+	Sim	Unchecked	Checked
	2	Ensino funda	Sim	Sim	0-3	Sim	Unchecked	Unchecked
	1	Ensino funda	Não	Não	0-3	Sim	Checked	Checked
nínimos		Ensino funda	Não sei	Sim	4+	Sim	Unchecked	Checked
	1	Ensino funda	Não	Não	0-3	Sim	Checked	Checked
	4	Ensino funda	Não	Sim	4+	Não	Unchecked	Unchecked
	2	Nenhum	Sim	Sim	0-3	Não	Unchecked	Unchecked
	5	Nenhum	Não	Não	0-3	Não	Unchecked	Unchecked
	3	Ensino funda	Sim	Sim	0-3	Sim	Unchecked	Unchecked
	1	Ensino funda	Sim	Não	0-3	Não	Unchecked	Unchecked
	1	Ensino funda	Recusou a	Não	0-3	Não	Unchecked	Unchecked
	4	Ensino funda	Não	Sim	0-3	Sim	Checked	Checked
	5	Ensino funda	Sim	Sim	4+	Não	Unchecked	Unchecked
	3	Ensino funda	Não	Sim	0-3	Sim	Checked	Unchecked
	3	Nível superio	Não	Sim	0-3	Não	Unchecked	Unchecked
	3	Ensino funda	Sim	Sim	0-3	Sim	Checked	Unchecked
	2	Ensino médic	Sim	Não	0-3	Sim	Checked	Checked
	4	Nenhum	Sim	Não	0-3	Sim	Unchecked	Checked
	5	Ensino médic	Sim	Não	0-3	Não	Unchecked	Unchecked
	1	Ensino funda	Não	Não	0-3	Sim	Checked	Checked
	3	Ensino funda	Sim	Sim	0-3	Sim	Checked	Checked
	5	Ensino funda	Sim	Não	0-3	Sim	Checked	Unchecked
	3	Ensino funda	Não	Sim	0-3	Sim	Checked	Checked
	3	Ensino médic	Sim	Não	0-3	Não sei	Unchecked	Unchecked
	5	Ensino médic	Sim	Não	0-3	Sim	Unchecked	Checked
	2	Ensino funda	Sim	Não	0-3	Sim	Checked	Unchecked
	3	Ensino funda	Não	Sim	0-3	Sim	Checked	Checked
	5	Nenhum	Sim	Sim	0-3	Sim	Checked	Unchecked
	5	Ensino funda	Sim	Não	0-3	Sim	Checked	Unchecked
	5	Ensino funda	Sim	Não	0-3	Sim	Checked	Unchecked
	2	Nível Técnico	Sim	Sim	0-3	Sim	Unchecked	Checked
	2	Nenhum	Sim	Sim	0-3	Sim	Checked	Unchecked
	4	Ensino funda	Sim	Sim	0-3	Sim	Checked	Unchecked
	5	Ensino médic	Sim	Não	0-3	Sim	Checked	Unchecked
	4	Ensino fundamental		Não	0-3	Sim	Checked	Unchecked
	4	Ensino fundamental		Não	0-3	Sim	Checked	Unchecked
	4	Nenhum	Sim	Sim	0-3	Sim	Checked	Unchecked
	2	Nenhum	Sim	Sim	0-3	Sim	Checked	Unchecked

	2	Nenhum	Sim	Sim	0-3	Sim	Checked	Unchecked
	4	Nenhum	Sim	Sim	0-3		Unchecked	Unchecked
	1	Nenhum	Não sei	Não	0-3	Sim	Checked	Unchecked
	3	Ensino funda	Sim	Não	0-3	Sim	Checked	Unchecked
	3	Ensino funda	Sim	Não	0-3	Sim	Checked	Unchecked
	3	Ensino funda	Sim	Não	0-3	Sim	Checked	Unchecked
	3	Ensino funda	Sim	Não	0-3	Sim	Checked	Unchecked
	3	Ensino funda	Sim	Não	0-3	Sim	Checked	Unchecked
	3	Ensino funda	Sim	Não	0-3	Sim	Checked	Unchecked
	3	Ensino funda	Sim	Não	0-3	Sim	Checked	Unchecked
	3	Ensino funda	Sim	Não	0-3	Sim	Checked	Unchecked
	3	Ensino funda	Sim	Não	0-3	Sim	Checked	Unchecked
	3	Ensino funda	Sim	Não	0-3	Sim	Checked	Unchecked
	5	Ensino funda	Sim	Sim	0-3	Sim	Checked	Checked
	1	Ensino funda	Sim	Sim	0-3	Não sei	Unchecked	Unchecked
	4	Ensino funda	Sim	Não	4+	Não	Unchecked	Unchecked
	3	Ensino funda	Não sei	Não	0-3	Sim	Checked	Unchecked
	2	Ensino funda	Não	Não	0-3	Sim	Checked	Checked
	5	Ensino funda	Sim	Sim	4+	Não	Unchecked	Unchecked
	4	Ensino funda	Não	Sim	4+	Não	Unchecked	Unchecked
	5	Ensino funda	Sim	Sim	4+	Não	Unchecked	Unchecked
	3	Ensino funda	Sim	Não	0-3	Não	Unchecked	Unchecked
	2	Ensino funda	Sim	Não	0-3	Sim	Unchecked	Checked
	3	Ensino funda	Sim	Sim	0-3	Sim	Checked	Checked
nimo		Ensino funda	Sim	Não	4+	Não sei	Unchecked	Unchecked
	1	Ensino funda	Sim	Não	0-3	Não	Unchecked	Unchecked
	5	Ensino funda	Não	Não	0-3	Sim	Unchecked	Checked
	4	Nenhum	Não	Não	0-3	Sim	Unchecked	Checked
	1	Nenhum		Não	0-3	Não	Unchecked	Unchecked
	3	Ensino médic	Sim	Sim	0-3	Sim	Checked	Unchecked
	1	Ensino funda	Sim	Não	0-3	Sim	Unchecked	Unchecked
	22	Ensino funda	Sim	Não	0-3	Não	Unchecked	Unchecked
	2	Nenhum	Sim	Não	0-3	Não	Unchecked	Unchecked
	2	Ensino funda	Sim	Não	0-3	Sim	Unchecked	Unchecked
	3	Ensino médic	Sim	Sim	0-3	Não sei	Unchecked	Unchecked
	2	Ensino funda	Sim	Não	0-3	Sim	Checked	Unchecked
	2	Ensino funda	Sim	Não	0-3	Sim	Checked	Checked
	1	Ensino funda	Sim	Sim	0-3	Sim	Unchecked	Unchecked
	1	Ensino funda	Sim	Não	0-3	Não	Unchecked	Unchecked
	4	Ensino médic	Não	Não	4+	Não	Unchecked	Unchecked
	1	Ensino médic	Sim	Não	0-3	Não sei	Unchecked	Unchecked
	3	Nenhum	Sim	Não	0-3	Não sei	Unchecked	Unchecked
	2	Ensino funda	Sim	Sim	0-3	Não	Unchecked	Unchecked

	4	Ensino funda	Sim	Sim	4+	Não	Unchecked	Unchecked
	3	Ensino fundamental		Sim	0-3	Sim	Checked	Unchecked
	2	Ensino funda	Sim	Não	0-3	Sim	Checked	Unchecked
	2		Sim	Não	0-3	Não	Unchecked	Unchecked
	1	Nenhum	Não	Não	0-3	Sim	Unchecked	Unchecked
	4	Ensino funda	Sim	Não	4+	Não	Unchecked	Unchecked
	2	Ensino funda	Não	Não	0-3	Sim	Unchecked	Checked
	1	Não sei	Sim	Não	0-3	Não	Unchecked	Unchecked
	3	Nenhum	Não	Não	0-3	Não	Unchecked	Unchecked
	3	Nenhum	Não sei	Não	4+	Não sei	Unchecked	Unchecked
	2	Ensino funda	Sim	Não	0-3	Sim	Checked	Unchecked
	4	Nível superio	Sim	Sim	0-3	Sim	Unchecked	Unchecked
	4	Ensino funda	Sim	Não	0-3	Sim	Unchecked	Unchecked
	2	Ensino funda	Sim	Não	0-3	Não	Unchecked	Unchecked
	3	Ensino médic	Sim	Não	0-3	Não	Unchecked	Unchecked
	2	Ensino médic	Sim	Não	0-3	Não sei	Unchecked	Unchecked
	3	Ensino funda	Sim	Não	4+	Sim	Checked	Unchecked
nimo		Ensino médic	Sim	Não	4+	Não sei	Unchecked	Unchecked
	4	Ensino médic	Sim	Não	0-3	Sim	Unchecked	Unchecked
	1	Ensino funda	Não	Sim	0-3	Sim	Unchecked	Unchecked
	4	Ensino funda	Sim	Sim	0-3	Sim	Unchecked	Unchecked
	1	Ensino médic	Sim	Não	0-3	Não	Unchecked	Unchecked
	2	Ensino médic	Não	Não	0-3	Sim	Checked	Unchecked
	2	Ensino fundamental		Sim	0-3	Não	Unchecked	Unchecked
	3	Ensino funda	Sim	Não	0-3	Não	Unchecked	Unchecked
	2	Ensino funda	Sim	Não	0-3	Sim	Checked	Unchecked
	2	Ensino funda	Não sei	Sim	4+	Sim	Unchecked	Checked
	2	Ensino médic	Sim	Sim	0-3	Sim	Unchecked	Unchecked
	2	Ensino médic	Sim	Não	0-3	Não sei	Unchecked	Unchecked
	2	Ensino médic	Sim	Não	0-3	Não	Unchecked	Unchecked
	2	Ensino funda	Não	Não	0-3	Sim	Checked	Unchecked
	3	Ensino médic	Sim	Não	0-3	Não	Unchecked	Unchecked
	2	Ensino médic	Sim	Não	0-3	Sim	Unchecked	Unchecked
	3	Ensino funda	Sim	Não	0-3	Sim	Checked	Unchecked
	4	Nenhum	Sim	Sim	0-3	Não	Unchecked	Unchecked
	5	Ensino médic	Sim	Sim	4+	Não	Unchecked	Unchecked
	3	Ensino funda	Sim	Não	0-3	Não sei	Unchecked	Unchecked
	3	Ensino funda	Sim	Não	0-3	Não sei	Unchecked	Unchecked
	4	Ensino funda	Sim	Não	0-3	Não sei	Unchecked	Unchecked
	3	Recusou a re	Sim	Não	0-3	Sim	Unchecked	Unchecked
	3	Ensino funda	Não	Não	0-3	Sim	Unchecked	Unchecked
	2	Ensino funda	Não	Não	0-3	Sim	Checked	Checked
	4	Ensino médic	Sim	Sim	Recusou a	Não	Unchecked	Unchecked

	4	Ensino funda	Sim	Sim	0-3	Sim	Checked	Unchecked
	2	Ensino funda	Sim	Sim	0-3	Sim	Checked	Unchecked
	4	Ensino funda	Sim	Sim	0-3	Sim	Checked	Unchecked
	2	Ensino médio		Não	0-3	Sim	Checked	Unchecked
	2	Ensino médic	Sim	Sim		Não	Unchecked	Unchecked
	3	Ensino funda	Sim	Sim	0-3	Sim	Checked	Unchecked
	4	Ensino funda	Sim	Sim	0-3	Sim	Checked	Unchecked
	5	Ensino médic	Sim	Não	0-3	Não	Unchecked	Unchecked
	1	Ensino médic	Não	Não	0-3	Sim	Checked	Unchecked
	2	Ensino funda	Sim	Não	0-3	Sim	Checked	Checked
	7	Ensino funda	Sim	Não	4+	Sim	Checked	Checked
	2	Ensino médic	Sim	Sim	0-3	Não	Unchecked	Unchecked
	5	Ensino médic	Sim	Sim	0-3	Sim	Checked	Unchecked
	5	Ensino funda	Não	Não	0-3	Não	Unchecked	Unchecked
	5	Ensino funda	Sim	Não	0-3	Não	Unchecked	Unchecked
	4	Ensino médic	Sim	Sim	0-3	Não sei	Unchecked	Unchecked
	1	Ensino funda	Não	Não	0-3	Sim	Checked	Unchecked
	2	Ensino funda	Não	Sim	0-3	Não	Unchecked	Unchecked
	4	Ensino médic	Sim	Sim	0-3	Sim	Checked	Unchecked
	2	Ensino funda	Sim	Não	0-3	Não	Unchecked	Unchecked
	1	Nenhum	Não	Não	0-3	Sim	Unchecked	Checked
	1	Ensino funda	Sim	Não	0-3	Não	Unchecked	Unchecked
	3	Ensino funda	Sim	Sim	0-3	Não	Unchecked	Unchecked
	2	Ensino médic	Sim	Sim	0-3	Sim	Checked	Unchecked
	2	Ensino médic	Sim	Sim	0-3	Não	Unchecked	Unchecked
	2	Ensino funda	Sim	Não	0-3	Não	Unchecked	Unchecked
	1	Ensino funda	Sim	Sim	0-3	Sim	Unchecked	Unchecked
nínimos		Ensino funda	Sim	Não	0-3	Sim	Unchecked	Unchecked
	3	Recusou a re	Sim	Sim	0-3	Não	Unchecked	Unchecked
nimo		Ensino funda	Não	Recusou a re	Recusou a	Sim	Checked	Unchecked
	3	Ensino funda	Sim	Sim	0-3	Sim	Unchecked	Unchecked
	1	Ensino médic	Sim	Não	0-3	Não	Unchecked	Unchecked
	4	Ensino médic	Sim	Não	0-3	Não	Unchecked	Unchecked
	2	Ensino funda	Sim	Não	0-3	Não	Unchecked	Unchecked
	4	Ensino funda	Sim	Não	0-3	Não	Unchecked	Unchecked
	1	Não sei	Sim	Não	0-3	Não sei	Unchecked	Unchecked
	3	Ensino médic	Sim	Sim	0-3	Sim	Checked	Checked
	1	Ensino médic	Sim	Não	0-3	Não	Unchecked	Unchecked
	3	Ensino médic	Sim	Não	0-3	Sim	Checked	Unchecked
	3	Ensino funda	Sim	Sim	0-3	Sim	Unchecked	Unchecked
	3	Ensino médic	Sim	Não	0-3	Não	Unchecked	Unchecked
	3	Ensino médic	Sim	Não	0-3	Não sei	Unchecked	Unchecked
	1	Ensino funda	Sim	Não	0-3	Sim	Checked	Unchecked

	4	Ensino funda	Sim	Sim	0-3	Sim	Unchecked	Unchecked
	6	Ensino médic	Sim	Sim	0-3	Não	Unchecked	Unchecked
	4	Nível superio	Sim	Não	0-3	Não	Unchecked	Unchecked
	2	Ensino funda	Não	Não	0-3	Sim	Checked	Unchecked
	4	Ensino funda	Sim	Sim	0-3	Não	Unchecked	Unchecked
	2	Ensino funda	Sim	Sim	0-3	Não	Unchecked	Unchecked
	2	Ensino funda	Sim	Não	0-3	Sim	Checked	Unchecked
	2	Ensino funda	Sim	Sim	0-3	Sim	Unchecked	Unchecked
	3	Nível superio	Sim	Sim	0-3	Sim	Checked	Checked
	3	Nível Técnico	Sim	Não	0-3	Sim	Unchecked	Unchecked
	2	Ensino funda	Não	Não	0-3	Sim	Checked	Unchecked
	2	Ensino médio		Não	0-3	Sim	Checked	Unchecked
	2	Ensino funda	Não	Não	0-3	Não	Unchecked	Unchecked
	3	Ensino funda	Não	Não	0-3	Sim	Unchecked	Unchecked
	4	Ensino funda	Não	Sim	0-3	Sim	Checked	Unchecked
	1	Nenhum	Não	Não	0-3	Sim	Unchecked	Unchecked
	3	Ensino médic	Sim	Sim	0-3	Sim	Checked	Unchecked
	4	Ensino médic	Sim	Sim	0-3	Sim	Unchecked	Unchecked
	4	Ensino médic	Sim	Sim	0-3	Sim	Unchecked	Unchecked
	5	Ensino funda	Não	Sim	0-3	Sim	Unchecked	Unchecked
nínimos		Ensino funda	Sim	Sim	0-3	Sim	Unchecked	Unchecked
	6	Ensino funda	Sim	Sim	0-3	Sim	Checked	Unchecked
	6	Ensino médic	Sim	Sim	0-3	Sim	Checked	Unchecked
	4	Ensino médic	Sim	Sim	0-3	Sim	Unchecked	Unchecked
nimo		Ensino funda	Sim	Sim	0-3	Sim	Checked	Unchecked
	2	Ensino funda	Sim	Sim	0-3	Sim	Checked	Unchecked
	2	Ensino funda	Sim	Sim	0-3	Sim	Checked	Unchecked
	3	Ensino funda	Sim	Sim	0-3	Sim	Checked	Unchecked
	2	Ensino funda	Sim	Não	0-3	Sim	Checked	Checked
	1	Ensino funda	Não	Não	0-3	Não	Unchecked	Unchecked
	3	Ensino médic	Sim	Sim	0-3	Não	Unchecked	Unchecked
	6	Nível superio	Sim	Não	0-3	Sim	Unchecked	Unchecked
	5	Ensino funda	Sim	Não	0-3	Sim	Checked	Unchecked
	3	Ensino funda	Sim	Não	0-3	Sim	Checked	Unchecked
	3	Ensino funda	Não sei	Não	0-3	Sim	Unchecked	Unchecked
	2	Ensino funda	Sim	Não	0-3	Sim	Unchecked	Checked
	2	Ensino funda	Não	Sim	0-3	Sim	Unchecked	Unchecked
	2	Ensino funda	Sim	Sim	0-3	Sim	Unchecked	Unchecked
	2	Ensino funda	Sim	Sim	0-3	Não sei	Unchecked	Unchecked
	1	Ensino funda	Sim	Não	0-3	Sim	Checked	Checked
	2	Ensino funda	Sim	Não	0-3	Sim	Checked	Unchecked
	5	Ensino funda	Sim	Não	0-3	Sim	Unchecked	Unchecked
	2	Ensino funda	Não	Não	0-3	Sim	Checked	Unchecked

						Unchecked	Unchecked
						Unchecked	Unchecked
2	Nenhum	Não	Não	0-3	Sim	Checked	Checked
3	Ensino funda	Sim	Não	0-3	Sim	Unchecked	Unchecked
4	Ensino funda	Não	Sim	0-3	Sim	Checked	Unchecked
2	Ensino funda	Não	Sim	0-3	Sim	Checked	Checked
3	Ensino funda	Sim	Não	0-3	Sim	Unchecked	Unchecked
5	Ensino funda	Sim	Sim	0-3	Sim	Checked	Unchecked
4	Ensino médic	Sim	Não	4+	Sim	Unchecked	Unchecked
2	Ensino médic	Sim	Sim	0-3	Não	Unchecked	Unchecked
1	Ensino funda	Sim	Não	0-3	Não sei	Unchecked	Unchecked
3	Ensino funda	Não	Não	0-3	Sim	Checked	Unchecked
5	Nenhum	Sim	Sim	0-3	Não	Unchecked	Unchecked
2	Ensino funda	Sim	Não	0-3	Sim	Checked	Unchecked
3	Ensino médic	Não	Não	0-3	Sim	Checked	Unchecked
2	Ensino funda	Sim	Sim	0-3	Sim	Checked	Checked
2	Nenhum	Sim	Não	0-3	Sim	Checked	Checked
4	Recusou a re	Sim	Sim	0-3	Não	Unchecked	Unchecked
2	Nenhum	Não	Sim	0-3	Não	Unchecked	Unchecked
5	Nenhum	Não	Não	4+	Não	Unchecked	Unchecked
3	Ensino funda	Sim	Sim	0-3	Sim	Unchecked	Unchecked
1	Ensino funda	Sim	Sim	0-3	Sim	Unchecked	Unchecked
3	Ensino funda	Sim	Sim	0-3	Não	Unchecked	Unchecked
6	Nenhum	Sim	Não	0-3	Sim	Checked	Unchecked
2	Ensino funda	Não	Sim	0-3	Sim	Checked	Unchecked
1	Ensino funda	Não	Não	0-3	Não	Unchecked	Unchecked
3	Ensino médic	Sim	Sim	0-3	Não	Unchecked	Unchecked
3	Ensino médic	Sim	Sim	0-3	Sim	Checked	Unchecked
4	Nenhum	Sim	Não	0-3	Não	Unchecked	Unchecked
7	Ensino funda	Não	Não	4+	Não	Unchecked	Unchecked
7	Ensino funda	Sim	Não	0-3	Sim	Checked	Unchecked
3	Ensino funda	Sim	Não	0-3	Sim	Unchecked	Checked
6	Ensino funda	Sim	Sim	Recusou a	Sim	Checked	Unchecked
3	Ensino funda	Não sei	Sim	0-3	Sim	Unchecked	Unchecked
3	Ensino funda	Sim	Sim	0-3	Sim	Unchecked	Unchecked
1	Ensino funda	Sim	Não	0-3	Sim	Unchecked	Checked
6	Ensino médic	Sim	Não	0-3	Sim	Checked	Unchecked
1	Nenhum	Não	Não	0-3	Não	Unchecked	Unchecked
NÃO SOUBE I	Recusou a re	Sim	Não	0-3	Não sei	Unchecked	Unchecked
3	Nenhum	Sim	Sim	0-3	Não	Unchecked	Unchecked
1	Ensino médic	Não	Não	0-3	Sim	Unchecked	Unchecked
4	Ensino funda	Sim	Sim	0-3	Sim	Unchecked	Unchecked
2	Ensino médic	Sim	Não	0-3	Não	Unchecked	Unchecked

2	Ensino médic	Sim	Não	0-3	Sim	Unchecked	Unchecked
4	Ensino funda	Sim	Não	Não sei	Sim	Unchecked	Unchecked
3	Pós-graduaçã	Sim	Sim	0-3	Sim	Unchecked	Unchecked
3	Ensino funda	Sim	Não	0-3	Não	Unchecked	Unchecked
3	Ensino funda	Sim	Sim	0-3	Sim	Checked	Unchecked
4	Ensino funda	Não sei	Sim	0-3	Sim	Checked	Unchecked
5	Ensino médic	Sim	Sim	4+	Sim	Unchecked	Unchecked
3	Ensino médic	Sim	Não	0-3	Não sei	Unchecked	Unchecked
1	Nenhum	Não	Sim	0-3	Sim	Unchecked	Unchecked
1	Nenhum	Não	Sim	0-3	Sim	Checked	Checked
1	Ensino funda	Sim	Não	0-3	Sim	Checked	Unchecked
4	Nível Técnico	Sim	Não	4+	Não	Unchecked	Unchecked
6	Ensino médic	Sim	Sim	0-3	Não	Unchecked	Unchecked
3	Ensino médic	Sim	Sim	0-3	Não	Unchecked	Unchecked
5	Ensino médic	Sim	Sim	0-3	Sim	Checked	Unchecked
8	Nenhum	Não	Não	0-3	Não	Unchecked	Unchecked
8	Ensino funda	Sim	Sim	0-3	Sim	Checked	Unchecked
4	Ensino funda	Sim	Sim	0-3	Sim	Checked	Unchecked
3	Ensino funda	Sim	Não	0-3	Não	Unchecked	Unchecked
1	Nenhum	Sim	Não	0-3	Não	Unchecked	Unchecked
3	Ensino funda	Sim	Não	0-3	Sim	Checked	Unchecked
2	Nenhum	Sim	Não	0-3	Sim	Checked	Unchecked
2	Ensino funda	Sim	Não	0-3	Sim	Checked	Unchecked
2	Nenhum	Não	Não	0-3	Não	Unchecked	Unchecked
4	Ensino funda	Não	Não	4+	Não	Unchecked	Unchecked
1	Ensino funda	Sim	Sim	0-3	Sim	Checked	Unchecked
5	Ensino funda	Sim	Sim	0-3	Não	Unchecked	Unchecked
3	Nenhum	Não	Não	0-3	Não	Unchecked	Unchecked
3	Ensino médic	Sim	Não	0-3	Sim	Checked	Unchecked
1	Ensino funda	Sim	Não	0-3	Sim	Checked	Unchecked
2	Nenhum	Sim	Sim	0-3	Sim	Checked	Unchecked
2	Ensino funda	Não sei	Não	0-3	Não	Unchecked	Unchecked
3	Ensino funda	Não	Sim	0-3	Sim	Unchecked	Checked
2	Ensino médic	Sim	Não	0-3	Sim	Checked	Unchecked
4	Ensino funda	Sim	Não	4+	Não	Unchecked	Unchecked
2	Ensino médic	Sim	Não	0-3	Não	Unchecked	Unchecked
1	Ensino funda	Sim	Sim	0-3	Sim	Unchecked	Checked
7	Ensino funda	Sim	Sim	4+	Sim	Checked	Unchecked
4	Nenhum	Sim	Sim	4+	Não	Unchecked	Unchecked
1	Ensino funda	Sim	Não	0-3	Sim	Checked	Unchecked
4	Ensino funda	Sim	Sim	0-3	Sim	Unchecked	Unchecked
3	Ensino funda	Sim	Não	0-3	Não	Unchecked	Unchecked
4	Nenhum	Não	Sim	0-3	Sim	Checked	Checked

	1	Ensino médic	Sim	Não	0-3	Não	Unchecked	Unchecked
	5	Ensino funda	Sim	Sim	0-3	Não	Unchecked	Unchecked
	3	Ensino funda	Não	Não	0-3	Sim	Unchecked	Checked
	11	Ensino funda	Sim	Sim	0-3	Sim	Checked	Unchecked
	1	Nenhum	Sim	Não	0-3	Sim	Checked	Unchecked
	2	Ensino funda	Sim	Não	0-3	Sim	Checked	Unchecked
	2	Ensino funda	Sim	Não	0-3	Não sei	Unchecked	Unchecked
	7	Não sei	Sim	Sim	0-3	Não sei	Unchecked	Unchecked
	2	Ensino funda	Não	Não	0-3	Sim	Unchecked	Unchecked
	2	Ensino funda	Sim	Não	0-3	Não sei	Unchecked	Unchecked
	5	Ensino funda	Não	Sim	0-3	Sim	Unchecked	Checked
	1	Nenhum	Sim	Não	0-3	Não	Unchecked	Unchecked
	1	Ensino funda	Sim	Sim	0-3	Não sei	Unchecked	Unchecked
	4	Nenhum	Sim	Sim	0-3	Sim	Checked	Unchecked
	1	Ensino funda	Sim	Não	0-3	Sim	Checked	Unchecked
	2	Nenhum	Não	Sim	0-3	Sim	Unchecked	Unchecked
	4	Recusou a re	Sim	Não	0-3	Sim	Unchecked	Unchecked
	2	Ensino funda	Sim	Sim	0-3	Não	Unchecked	Unchecked
	2	Ensino funda	Não	Sim	0-3	Sim	Unchecked	Unchecked
	2	Ensino funda	Sim	Sim	0-3	Não sei	Unchecked	Unchecked
	3	Ensino funda	Não	Sim	0-3		Unchecked	Unchecked
	2	Ensino funda	Não	Sim	0-3	Não	Unchecked	Unchecked
	2	Ensino funda	Sim	Sim	0-3	Não	Unchecked	Unchecked
	3	Nenhum	Sim	Não	0-3	Não sei	Unchecked	Unchecked
	2	Ensino médic	Sim	Sim	0-3	Sim	Checked	Unchecked
	2	Ensino funda	Não	Sim	0-3	Sim	Checked	Unchecked
	2	Nenhum	Não	Sim	0-3	Sim	Checked	Unchecked
	4	Ensino funda	Não sei	Não	0-3	Não	Unchecked	Unchecked
	2	Ensino médic	Não	Sim	0-3	Não	Unchecked	Unchecked
	3	Pós-graduaçã	Sim	Sim	0-3	Sim	Unchecked	Unchecked
	3	Ensino funda	Sim	Sim	0-3	Sim	Unchecked	Unchecked
	3	Nível superio	Sim	Sim	0-3	Não	Unchecked	Unchecked
	4	Nível Técnico	Sim	Sim	0-3	Não	Unchecked	Unchecked
	2	Nível superio	Sim	Não	0-3	Sim	Checked	Unchecked
	1	Nenhum	Sim	Sim	0-3	Sim	Unchecked	Unchecked
	3	Nenhum	Não	Não	0-3	Sim	Checked	Checked
	4	Nenhum	Sim	Sim	0-3	Sim	Checked	Checked
	1	Ensino funda	Sim	Não	0-3	Sim	Checked	Checked
	2	Nenhum	Sim	Sim	0-3	Sim	Checked	Checked
sponder		Recusou a re	Recusou a	Recusou a re	Recusou a	Recusou a re	Unchecked	Unchecked
	1	Nenhum	Sim	Não	0-3	Sim	Checked	Unchecked
	4	Ensino médic	Sim	Sim	0-3	Sim	Checked	Unchecked
sponder		Recusou a re	Recusou a	Recusou a re	Recusou a	Recusou a re	Unchecked	Unchecked

sponder		Recusou a re	Não sei	Recusou a re	Recusou a	Recusou a re	Unchecked	Unchecked
sponder		Recusou a re	Não sei	Recusou a re	Recusou a	Recusou a re	Unchecked	Unchecked
sponder		Recusou a re	Recusou a	Recusou a re	Recusou a	Recusou a re	Unchecked	Unchecked
sponder		Recusou a re	Recusou a	Recusou a re	Recusou a	Recusou a re	Unchecked	Unchecked
sponder		Recusou a re	Não sei	Recusou a re	Recusou a	Recusou a re	Unchecked	Unchecked
sponder		Recusou a re	Recusou a	Recusou a re	Recusou a	Recusou a re	Unchecked	Unchecked
sponder		Recusou a re	Recusou a	Recusou a re	Recusou a	Recusou a re	Unchecked	Unchecked
sponder		Recusou a re	Não sei	Recusou a re	Recusou a	Recusou a re	Unchecked	Unchecked
		Ensino funda	Sim	Não sei	Não sei	Não sei	Unchecked	Unchecked
sponder		Recusou a re	Não sei	Recusou a re	Não sei	Não sei	Unchecked	Unchecked
sponder		Recusou a re	Não sei	Recusou a re	Recusou a	Não sei	Unchecked	Unchecked
sponder		Recusou a re	Não sei	Recusou a re	Não sei	Não sei	Unchecked	Unchecked
sponder		Não sei	Não sei	Não sei	Recusou a	Não sei	Unchecked	Unchecked
sponder		Recusou a re	Não sei	Recusou a re	Recusou a	Não sei	Unchecked	Unchecked
		Ensino funda	Sim	Não sei	Não sei	Não sei	Unchecked	Unchecked
	4	Ensino funda	Não sei	Recusou a re	0-3	Não sei	Unchecked	Unchecked
sponder		Recusou a re	Não sei	Recusou a re	Não sei	Não sei	Unchecked	Unchecked
sponder		Recusou a re	Recusou a	Recusou a re	Recusou a	Não sei	Unchecked	Unchecked
sponder		Recusou a re	Não sei	Recusou a re	Recusou a	Não sei	Unchecked	Unchecked
sponder		Recusou a re	Não sei	Recusou a re	Recusou a	Não sei	Unchecked	Unchecked
sponder		Recusou a re	Recusou a	Recusou a re	Recusou a	Recusou a re	Unchecked	Unchecked
sponder		Recusou a re	Não sei	Recusou a re	Recusou a	Não sei	Unchecked	Unchecked
sponder		Recusou a re	Recusou a	Recusou a re	Recusou a	Recusou a re	Unchecked	Unchecked
sponder		Recusou a re	Não sei	Recusou a re	Recusou a	Não sei	Unchecked	Unchecked
sponder		Ensino funda	Sim	Recusou a re	Recusou a	Não sei	Unchecked	Unchecked
sponder		Recusou a re	Não sei	Recusou a re	Recusou a	Recusou a re	Unchecked	Unchecked

Se sim, você	Se sim, você	Se sim, você	Se sim, você	Se sim, você	Se sim, você	Se sim, você
Unchecked	Unchecked	Unchecked	Unchecked	Unchecked	Unchecked	Unchecked
Unchecked	Unchecked	Unchecked	Unchecked	Unchecked	Unchecked	Unchecked
Unchecked	Unchecked	Unchecked	Unchecked	Checked	Unchecked	Unchecked
Unchecked	Unchecked	Unchecked	Unchecked	Unchecked	Unchecked	Unchecked
Unchecked	Unchecked	Checked	Unchecked	Checked	Unchecked	Unchecked
Unchecked	Checked	Unchecked	Unchecked	Unchecked	Unchecked	Unchecked
Unchecked	Unchecked	Unchecked	Unchecked	Unchecked	Unchecked	Unchecked
Unchecked	Unchecked	Unchecked	Unchecked	Unchecked	Unchecked	Unchecked
Unchecked	Unchecked	Unchecked	Unchecked	Unchecked	Unchecked	Unchecked
Unchecked	Unchecked	Unchecked	Unchecked	Unchecked	Unchecked	Unchecked
Unchecked	Unchecked	Unchecked	Unchecked	Unchecked	Unchecked	Unchecked
Unchecked	Unchecked	Unchecked	Unchecked	Unchecked	Unchecked	Unchecked
Unchecked	Unchecked	Unchecked	Unchecked	Unchecked	Unchecked	Unchecked
Unchecked	Unchecked	Unchecked	Unchecked	Unchecked	Unchecked	Unchecked
Unchecked	Unchecked	Unchecked	Unchecked	Unchecked	Unchecked	Unchecked
Unchecked	Unchecked	Unchecked	Unchecked	Unchecked	Unchecked	Unchecked
Unchecked	Unchecked	Unchecked	Unchecked	Unchecked	Unchecked	Unchecked
Unchecked	Unchecked	Unchecked	Unchecked	Unchecked	Unchecked	Unchecked
Unchecked	Unchecked	Unchecked	Unchecked	Unchecked	Unchecked	Unchecked
Unchecked	Unchecked	Unchecked	Unchecked	Unchecked	Unchecked	Unchecked
Unchecked	Unchecked	Checked	Unchecked	Unchecked	Unchecked	Unchecked
Unchecked	Unchecked	Unchecked	Unchecked	Unchecked	Unchecked	Unchecked
Unchecked	Unchecked	Unchecked	Unchecked	Unchecked	Unchecked	Unchecked
Unchecked	Unchecked	Unchecked	Unchecked	Unchecked	Unchecked	Unchecked
Unchecked	Unchecked	Checked	Unchecked	Unchecked	Unchecked	Unchecked
Unchecked	Unchecked	Checked	Unchecked	Checked	Unchecked	Unchecked
Unchecked	Unchecked	Unchecked	Unchecked	Unchecked	Unchecked	Unchecked
Unchecked	Unchecked	Checked	Unchecked	Unchecked	Unchecked	Unchecked
Unchecked	Unchecked	Checked	Unchecked	Unchecked	Unchecked	Unchecked
Unchecked	Unchecked	Unchecked	Unchecked	Unchecked	Unchecked	Unchecked
Unchecked	Unchecked	Unchecked	Unchecked	Checked	Unchecked	Unchecked
Unchecked	Unchecked	Unchecked	Unchecked	Unchecked	Unchecked	Unchecked
Unchecked	Unchecked	Unchecked	Unchecked	Unchecked	Unchecked	Unchecked
Unchecked	Unchecked	Unchecked	Unchecked	Checked	Unchecked	Unchecked
Unchecked	Unchecked	Unchecked	Unchecked	Unchecked	Unchecked	Unchecked
Unchecked	Unchecked	Unchecked	Unchecked	Unchecked	Unchecked	Unchecked
Unchecked	Unchecked	Unchecked	Unchecked	Unchecked	Unchecked	Unchecked
Unchecked	Unchecked	Unchecked	Unchecked	Checked	Unchecked	Unchecked
Unchecked	Unchecked	Unchecked	Unchecked	Unchecked	Unchecked	Unchecked
Unchecked	Unchecked	Unchecked	Unchecked	Unchecked	Unchecked	Unchecked
Unchecked	Unchecked	Unchecked	Unchecked	Unchecked	Unchecked	Unchecked
Unchecked	Unchecked	Unchecked	Unchecked	Unchecked	Unchecked	Unchecked

Unchecked	Unchecked	Unchecked	Unchecked	Checked	Unchecked	Unchecked
Unchecked	Unchecked	Unchecked	Unchecked	Unchecked	Unchecked	Unchecked
Unchecked	Unchecked	Unchecked	Unchecked	Unchecked	Unchecked	Unchecked
Unchecked	Unchecked	Unchecked	Unchecked	Unchecked	Unchecked	Unchecked
Unchecked	Unchecked	Unchecked	Unchecked	Unchecked	Unchecked	Unchecked
Unchecked	Unchecked	Unchecked	Unchecked	Unchecked	Unchecked	Unchecked
Unchecked	Unchecked	Unchecked	Unchecked	Unchecked	Unchecked	Unchecked
Unchecked	Unchecked	Unchecked	Unchecked	Unchecked	Unchecked	Unchecked
Unchecked	Unchecked	Unchecked	Unchecked	Checked	Unchecked	Unchecked
Unchecked	Unchecked	Unchecked	Unchecked	Unchecked	Unchecked	Unchecked
Unchecked	Unchecked	Unchecked	Unchecked	Unchecked	Unchecked	Unchecked
Unchecked	Unchecked	Unchecked	Unchecked	Unchecked	Unchecked	Unchecked
Unchecked	Unchecked	Unchecked	Unchecked	Unchecked	Unchecked	Unchecked
Unchecked	Unchecked	Unchecked	Unchecked	Checked	Unchecked	Unchecked
Unchecked	Unchecked	Unchecked	Unchecked	Unchecked	Unchecked	Unchecked
Unchecked	Unchecked	Unchecked	Unchecked	Unchecked	Unchecked	Unchecked
Unchecked	Unchecked	Unchecked	Unchecked	Unchecked	Unchecked	Unchecked
Unchecked	Unchecked	Unchecked	Unchecked	Unchecked	Unchecked	Unchecked
Unchecked	Unchecked	Unchecked	Unchecked	Unchecked	Unchecked	Unchecked
Unchecked	Unchecked	Unchecked	Unchecked	Unchecked	Unchecked	Unchecked
Unchecked	Checked	Unchecked	Unchecked	Unchecked	Unchecked	Unchecked
Unchecked	Unchecked	Unchecked	Unchecked	Checked	Unchecked	Unchecked
Unchecked	Unchecked	Unchecked	Unchecked	Unchecked	Unchecked	Unchecked
Unchecked	Unchecked	Unchecked	Unchecked	Unchecked	Unchecked	Unchecked
Unchecked	Unchecked	Unchecked	Unchecked	Unchecked	Unchecked	Unchecked
Unchecked	Unchecked	Unchecked	Unchecked	Unchecked	Unchecked	Unchecked
Unchecked	Unchecked	Unchecked	Unchecked	Unchecked	Unchecked	Unchecked
Unchecked	Unchecked	Unchecked	Unchecked	Unchecked	Unchecked	Unchecked
Unchecked	Unchecked	Unchecked	Unchecked	Unchecked	Unchecked	Unchecked
Unchecked	Unchecked	Unchecked	Unchecked	Unchecked	Unchecked	Unchecked
Unchecked	Unchecked	Unchecked	Unchecked	Unchecked	Unchecked	Unchecked
Unchecked	Unchecked	Unchecked	Unchecked	Checked	Unchecked	Unchecked
Unchecked	Unchecked	Unchecked	Unchecked	Unchecked	Unchecked	Unchecked
Unchecked	Unchecked	Unchecked	Unchecked	Unchecked	Unchecked	Unchecked
Unchecked	Unchecked	Unchecked	Unchecked	Unchecked	Unchecked	Unchecked
Unchecked	Unchecked	Unchecked	Unchecked	Checked	Unchecked	Unchecked
Unchecked	Unchecked	Unchecked	Unchecked	Unchecked	Unchecked	Unchecked
Unchecked	Unchecked	Unchecked	Unchecked	Unchecked	Unchecked	Unchecked
Unchecked	Unchecked	Unchecked	Unchecked	Unchecked	Unchecked	Unchecked
Unchecked	Unchecked	Unchecked	Unchecked	Unchecked	Unchecked	Unchecked
Unchecked	Unchecked	Unchecked	Unchecked	Unchecked	Unchecked	Unchecked
Unchecked	Unchecked	Unchecked	Unchecked	Unchecked	Unchecked	Unchecked
Unchecked	Unchecked	Unchecked	Unchecked	Unchecked	Unchecked	Unchecked

Unchecked	Unchecked	Unchecked	Unchecked	Unchecked	Unchecked	Unchecked
Unchecked	Unchecked	Unchecked	Unchecked	Unchecked	Unchecked	Unchecked
Unchecked	Unchecked	Unchecked	Checked	Unchecked	Unchecked	Unchecked
Unchecked	Unchecked	Unchecked	Unchecked	Checked	Unchecked	Unchecked
Unchecked	Unchecked	Unchecked	Unchecked	Unchecked	Unchecked	Unchecked
Unchecked	Unchecked	Unchecked	Unchecked	Unchecked	Unchecked	Unchecked
Unchecked	Unchecked	Unchecked	Unchecked	Unchecked	Unchecked	Unchecked
Unchecked	Unchecked	Unchecked	Unchecked	Checked	Unchecked	Unchecked
Unchecked	Unchecked	Unchecked	Unchecked	Unchecked	Unchecked	Unchecked
Unchecked	Unchecked	Unchecked	Unchecked	Checked	Unchecked	Unchecked
Unchecked	Unchecked	Unchecked	Unchecked	Unchecked	Unchecked	Unchecked
Unchecked	Unchecked	Unchecked	Unchecked	Checked	Unchecked	Unchecked
Unchecked	Unchecked	Unchecked	Unchecked	Unchecked	Unchecked	Unchecked
Unchecked	Unchecked	Unchecked	Unchecked	Unchecked	Unchecked	Unchecked
Unchecked	Unchecked	Unchecked	Unchecked	Unchecked	Unchecked	Unchecked
Unchecked	Unchecked	Unchecked	Unchecked	Unchecked	Unchecked	Unchecked
Unchecked	Unchecked	Unchecked	Unchecked	Unchecked	Unchecked	Unchecked
Unchecked	Unchecked	Unchecked	Unchecked	Unchecked	Unchecked	Unchecked
Unchecked	Unchecked	Checked	Unchecked	Unchecked	Unchecked	Unchecked
Unchecked	Unchecked	Unchecked	Unchecked	Unchecked	Unchecked	Unchecked
Unchecked	Unchecked	Unchecked	Unchecked	Unchecked	Unchecked	Unchecked
Unchecked	Unchecked	Unchecked	Unchecked	Unchecked	Unchecked	Unchecked
Unchecked	Unchecked	Checked	Unchecked	Unchecked	Unchecked	Unchecked
Unchecked	Unchecked	Unchecked	Unchecked	Unchecked	Unchecked	Unchecked
Unchecked	Unchecked	Unchecked	Unchecked	Checked	Unchecked	Unchecked
Unchecked	Unchecked	Unchecked	Unchecked	Unchecked	Unchecked	Unchecked
Unchecked	Unchecked	Unchecked	Unchecked	Unchecked	Unchecked	Unchecked
Unchecked	Unchecked	Unchecked	Unchecked	Unchecked	Unchecked	Unchecked
Unchecked	Unchecked	Unchecked	Unchecked	Unchecked	Unchecked	Unchecked
Unchecked	Unchecked	Unchecked	Unchecked	Unchecked	Unchecked	Unchecked
Unchecked	Unchecked	Unchecked	Unchecked	Unchecked	Unchecked	Unchecked
Unchecked	Unchecked	Checked	Unchecked	Unchecked	Unchecked	Unchecked
Unchecked	Unchecked	Unchecked	Unchecked	Unchecked	Unchecked	Unchecked
Unchecked	Unchecked	Checked	Unchecked	Unchecked	Unchecked	Unchecked
Unchecked	Unchecked	Unchecked	Checked	Unchecked	Unchecked	Unchecked
Unchecked	Unchecked	Checked	Unchecked	Unchecked	Unchecked	Unchecked
Unchecked	Unchecked	Checked	Unchecked	Unchecked	Unchecked	Unchecked
Unchecked	Checked	Unchecked	Unchecked	Unchecked	Unchecked	Unchecked
Unchecked	Unchecked	Unchecked	Unchecked	Unchecked	Unchecked	Unchecked
Unchecked	Unchecked	Unchecked	Unchecked	Checked	Unchecked	Unchecked
Unchecked	Unchecked	Unchecked	Unchecked	Unchecked	Unchecked	Unchecked
Unchecked	Unchecked	Checked	Unchecked	Unchecked	Unchecked	Unchecked
Unchecked	Unchecked	Unchecked	Unchecked	Unchecked	Unchecked	Unchecked

Unchecked	Unchecked	Unchecked	Unchecked	Unchecked	Unchecked	Unchecked
Unchecked	Unchecked	Unchecked	Unchecked	Unchecked	Unchecked	Unchecked
Unchecked	Unchecked	Unchecked	Unchecked	Unchecked	Unchecked	Unchecked
Unchecked	Unchecked	Unchecked	Unchecked	Unchecked	Unchecked	Unchecked
Unchecked	Unchecked	Unchecked	Unchecked	Unchecked	Unchecked	Unchecked
Unchecked	Checked	Unchecked	Unchecked	Unchecked	Unchecked	Unchecked
Unchecked	Unchecked	Unchecked	Unchecked	Checked	Unchecked	Unchecked
Unchecked	Unchecked	Unchecked	Unchecked	Unchecked	Unchecked	Unchecked
Unchecked	Unchecked	Unchecked	Unchecked	Unchecked	Unchecked	Unchecked
Unchecked	Unchecked	Unchecked	Unchecked	Unchecked	Unchecked	Unchecked
Unchecked	Unchecked	Unchecked	Unchecked	Unchecked	Unchecked	Unchecked
Unchecked	Unchecked	Unchecked	Unchecked	Unchecked	Unchecked	Unchecked
Unchecked	Unchecked	Unchecked	Unchecked	Unchecked	Unchecked	Unchecked
Unchecked	Unchecked	Unchecked	Unchecked	Unchecked	Unchecked	Unchecked
Unchecked	Unchecked	Unchecked	Unchecked	Unchecked	Unchecked	Unchecked
Unchecked	Unchecked	Unchecked	Unchecked	Unchecked	Unchecked	Unchecked
Unchecked	Unchecked	Unchecked	Unchecked	Unchecked	Unchecked	Unchecked
Unchecked	Unchecked	Unchecked	Unchecked	Checked	Unchecked	Unchecked
Unchecked	Unchecked	Unchecked	Unchecked	Unchecked	Unchecked	Unchecked
Unchecked	Unchecked	Unchecked	Unchecked	Unchecked	Unchecked	Unchecked
Unchecked	Unchecked	Unchecked	Unchecked	Unchecked	Unchecked	Unchecked
Unchecked	Unchecked	Unchecked	Unchecked	Unchecked	Unchecked	Unchecked
Unchecked	Unchecked	Unchecked	Unchecked	Unchecked	Unchecked	Unchecked
Unchecked	Checked	Unchecked	Unchecked	Unchecked	Unchecked	Unchecked
Unchecked	Unchecked	Unchecked	Unchecked	Unchecked	Unchecked	Unchecked
Unchecked	Unchecked	Unchecked	Unchecked	Checked	Unchecked	Unchecked
Unchecked	Unchecked	Unchecked	Unchecked	Unchecked	Unchecked	Unchecked
Unchecked	Unchecked	Unchecked	Unchecked	Checked	Unchecked	Unchecked
Unchecked	Unchecked	Unchecked	Unchecked	Checked	Unchecked	Unchecked
Unchecked	Unchecked	Unchecked	Unchecked	Unchecked	Unchecked	Unchecked
Unchecked	Unchecked	Unchecked	Unchecked	Unchecked	Unchecked	Unchecked
Unchecked	Unchecked	Unchecked	Unchecked	Unchecked	Unchecked	Unchecked
Unchecked	Unchecked	Unchecked	Unchecked	Unchecked	Unchecked	Unchecked
Unchecked	Unchecked	Unchecked	Unchecked	Unchecked	Unchecked	Unchecked
Unchecked	Unchecked	Unchecked	Unchecked	Unchecked	Unchecked	Unchecked
Unchecked	Unchecked	Unchecked	Unchecked	Unchecked	Unchecked	Unchecked
Unchecked	Unchecked	Checked	Unchecked	Unchecked	Unchecked	Unchecked
Unchecked	Unchecked	Unchecked	Unchecked	Unchecked	Unchecked	Unchecked
Unchecked	Unchecked	Checked	Unchecked	Checked	Unchecked	Unchecked
Unchecked	Unchecked	Unchecked	Unchecked	Unchecked	Unchecked	Unchecked
Unchecked	Unchecked	Unchecked	Unchecked	Unchecked	Unchecked	Unchecked
Unchecked	Checked	Unchecked	Unchecked	Unchecked	Unchecked	Unchecked
Unchecked	Unchecked	Unchecked	Unchecked	Unchecked	Unchecked	Unchecked

Unchecked	Unchecked	Unchecked	Unchecked	Unchecked	Unchecked	Unchecked
Unchecked	Unchecked	Unchecked	Unchecked	Unchecked	Unchecked	Unchecked
Unchecked	Unchecked	Checked	Unchecked	Checked	Unchecked	Unchecked
Unchecked	Unchecked	Checked	Unchecked	Unchecked	Unchecked	Unchecked
Unchecked	Unchecked	Unchecked	Unchecked	Unchecked	Unchecked	Unchecked
Unchecked	Unchecked	Unchecked	Unchecked	Checked	Unchecked	Unchecked
Unchecked	Unchecked	Unchecked	Unchecked	Unchecked	Unchecked	Unchecked
Unchecked	Unchecked	Unchecked	Unchecked	Unchecked	Unchecked	Unchecked
Unchecked	Checked	Checked	Unchecked	Unchecked	Unchecked	Unchecked
Unchecked	Unchecked	Unchecked	Unchecked	Unchecked	Unchecked	Unchecked
Checked	Unchecked	Unchecked	Checked	Checked	Unchecked	Unchecked
Unchecked	Unchecked	Unchecked	Unchecked	Unchecked	Unchecked	Unchecked
Unchecked	Unchecked	Unchecked	Unchecked	Unchecked	Unchecked	Unchecked
Unchecked	Unchecked	Unchecked	Unchecked	Unchecked	Unchecked	Unchecked
Unchecked	Unchecked	Unchecked	Unchecked	Unchecked	Unchecked	Unchecked
Unchecked	Unchecked	Unchecked	Unchecked	Unchecked	Unchecked	Unchecked
Unchecked	Unchecked	Unchecked	Unchecked	Unchecked	Unchecked	Unchecked
Unchecked	Unchecked	Unchecked	Unchecked	Unchecked	Unchecked	Unchecked
Unchecked	Unchecked	Unchecked	Unchecked	Unchecked	Unchecked	Unchecked
Unchecked	Unchecked	Unchecked	Unchecked	Unchecked	Unchecked	Unchecked
Unchecked	Unchecked	Unchecked	Unchecked	Unchecked	Unchecked	Unchecked
Unchecked	Unchecked	Unchecked	Unchecked	Unchecked	Unchecked	Unchecked
Unchecked	Unchecked	Unchecked	Unchecked	Unchecked	Unchecked	Unchecked
Unchecked	Unchecked	Unchecked	Unchecked	Unchecked	Unchecked	Unchecked
Unchecked	Unchecked	Checked	Unchecked	Unchecked	Unchecked	Unchecked
Unchecked	Unchecked	Unchecked	Unchecked	Unchecked	Unchecked	Unchecked
Unchecked	Unchecked	Unchecked	Unchecked	Unchecked	Unchecked	Unchecked
Unchecked	Unchecked	Unchecked	Unchecked	Unchecked	Unchecked	Unchecked
Unchecked	Unchecked	Unchecked	Unchecked	Unchecked	Unchecked	Unchecked
Unchecked	Unchecked	Unchecked	Unchecked	Unchecked	Unchecked	Unchecked
Unchecked	Unchecked	Unchecked	Unchecked	Unchecked	Unchecked	Unchecked
Unchecked	Unchecked	Unchecked	Unchecked	Unchecked	Unchecked	Unchecked
Unchecked	Unchecked	Unchecked	Unchecked	Unchecked	Unchecked	Unchecked
Unchecked	Unchecked	Unchecked	Unchecked	Unchecked	Checked	Unchecked
Unchecked	Unchecked	Unchecked	Unchecked	Unchecked	Unchecked	Unchecked
Unchecked	Unchecked	Unchecked	Unchecked	Unchecked	Unchecked	Unchecked
Unchecked	Unchecked	Unchecked	Unchecked	Checked	Unchecked	Unchecked
Unchecked	Unchecked	Unchecked	Unchecked	Unchecked	Unchecked	Unchecked
Unchecked	Unchecked	Unchecked	Unchecked	Unchecked	Unchecked	Unchecked
Unchecked	Unchecked	Unchecked	Unchecked	Unchecked	Unchecked	Unchecked
Unchecked	Unchecked	Unchecked	Unchecked	Unchecked	Unchecked	Unchecked
Unchecked	Unchecked	Unchecked	Unchecked	Unchecked	Unchecked	Unchecked
Unchecked	Unchecked	Unchecked	Unchecked	Unchecked	Unchecked	Unchecked

Unchecked	Unchecked	Unchecked	Unchecked	Unchecked	Unchecked	Unchecked
Unchecked	Unchecked	Checked	Unchecked	Unchecked	Unchecked	Unchecked
Unchecked	Unchecked	Unchecked	Unchecked	Unchecked	Unchecked	Unchecked
Unchecked	Unchecked	Unchecked	Unchecked	Unchecked	Unchecked	Unchecked
Unchecked	Unchecked	Unchecked	Unchecked	Unchecked	Unchecked	Unchecked
Unchecked	Unchecked	Unchecked	Unchecked	Unchecked	Unchecked	Unchecked
Unchecked	Unchecked	Unchecked	Unchecked	Unchecked	Unchecked	Unchecked
Unchecked	Unchecked	Unchecked	Unchecked	Unchecked	Unchecked	Unchecked
Unchecked	Unchecked	Unchecked	Unchecked	Unchecked	Unchecked	Unchecked
Unchecked	Unchecked	Unchecked	Unchecked	Unchecked	Unchecked	Unchecked
Unchecked	Unchecked	Unchecked	Unchecked	Unchecked	Unchecked	Unchecked
Unchecked	Checked	Unchecked	Unchecked	Unchecked	Unchecked	Unchecked
Unchecked	Unchecked	Unchecked	Unchecked	Unchecked	Unchecked	Unchecked
Unchecked	Unchecked	Unchecked	Unchecked	Unchecked	Unchecked	Unchecked
Unchecked	Unchecked	Unchecked	Unchecked	Unchecked	Unchecked	Unchecked
Unchecked	Unchecked	Unchecked	Unchecked	Unchecked	Unchecked	Unchecked
Unchecked	Unchecked	Unchecked	Unchecked	Unchecked	Unchecked	Unchecked
Unchecked	Unchecked	Unchecked	Unchecked	Unchecked	Unchecked	Unchecked
Unchecked	Checked	Checked	Unchecked	Unchecked	Unchecked	Unchecked
Unchecked	Unchecked	Unchecked	Unchecked	Unchecked	Unchecked	Unchecked
Unchecked	Unchecked	Unchecked	Unchecked	Unchecked	Unchecked	Unchecked
Unchecked	Unchecked	Unchecked	Unchecked	Unchecked	Unchecked	Unchecked
Unchecked	Unchecked	Checked	Unchecked	Unchecked	Unchecked	Unchecked
Unchecked	Unchecked	Unchecked	Unchecked	Unchecked	Unchecked	Unchecked
Unchecked	Checked	Unchecked	Checked	Checked	Unchecked	Unchecked
Unchecked	Unchecked	Unchecked	Unchecked	Unchecked	Unchecked	Unchecked
Unchecked	Unchecked	Unchecked	Unchecked	Unchecked	Unchecked	Unchecked
Unchecked	Unchecked	Unchecked	Unchecked	Unchecked	Unchecked	Unchecked
Unchecked	Unchecked	Unchecked	Unchecked	Unchecked	Unchecked	Unchecked
Unchecked	Unchecked	Unchecked	Unchecked	Unchecked	Unchecked	Unchecked
Unchecked	Unchecked	Unchecked	Unchecked	Unchecked	Unchecked	Unchecked
Unchecked	Unchecked	Unchecked	Unchecked	Unchecked	Unchecked	Unchecked
Unchecked	Checked	Unchecked	Unchecked	Unchecked	Unchecked	Unchecked
Unchecked	Unchecked	Unchecked	Unchecked	Unchecked	Unchecked	Unchecked
Unchecked	Unchecked	Unchecked	Unchecked	Unchecked	Unchecked	Unchecked
Unchecked	Unchecked	Checked	Unchecked	Checked	Unchecked	Unchecked
Unchecked	Unchecked	Unchecked	Unchecked	Unchecked	Unchecked	Unchecked
Unchecked	Unchecked	Unchecked	Unchecked	Unchecked	Unchecked	Unchecked
Unchecked	Unchecked	Unchecked	Unchecked	Unchecked	Unchecked	Unchecked
Unchecked	Unchecked	Unchecked	Checked	Checked	Unchecked	Unchecked
Unchecked	Unchecked	Unchecked	Unchecked	Unchecked	Unchecked	Unchecked
Unchecked	Unchecked	Checked	Unchecked	Unchecked	Unchecked	Unchecked
Unchecked	Unchecked	Checked	Unchecked	Unchecked	Unchecked	Unchecked

Unchecked	Unchecked	Unchecked	Unchecked	Unchecked	Unchecked	Unchecked
Unchecked	Unchecked	Unchecked	Unchecked	Unchecked	Unchecked	Unchecked
Unchecked	Unchecked	Unchecked	Unchecked	Checked	Unchecked	Unchecked
Unchecked	Unchecked	Unchecked	Unchecked	Unchecked	Unchecked	Unchecked
Unchecked	Unchecked	Unchecked	Unchecked	Unchecked	Unchecked	Unchecked
Unchecked	Unchecked	Unchecked	Unchecked	Unchecked	Unchecked	Unchecked
Unchecked	Unchecked	Unchecked	Unchecked	Unchecked	Unchecked	Unchecked
Unchecked	Unchecked	Unchecked	Unchecked	Unchecked	Unchecked	Unchecked
Unchecked	Unchecked	Unchecked	Unchecked	Checked	Unchecked	Unchecked
Unchecked	Unchecked	Unchecked	Unchecked	Unchecked	Unchecked	Unchecked
Unchecked	Unchecked	Unchecked	Unchecked	Unchecked	Unchecked	Unchecked
Unchecked	Unchecked	Unchecked	Unchecked	Unchecked	Unchecked	Unchecked
Unchecked	Unchecked	Unchecked	Unchecked	Unchecked	Unchecked	Unchecked
Unchecked	Unchecked	Unchecked	Unchecked	Unchecked	Unchecked	Unchecked
Unchecked	Unchecked	Unchecked	Unchecked	Unchecked	Unchecked	Unchecked
Unchecked	Unchecked	Unchecked	Unchecked	Unchecked	Unchecked	Unchecked
Unchecked	Unchecked	Unchecked	Checked	Unchecked	Unchecked	Unchecked
Unchecked	Unchecked	Unchecked	Unchecked	Unchecked	Unchecked	Unchecked
Unchecked	Unchecked	Unchecked	Unchecked	Unchecked	Unchecked	Unchecked
Unchecked	Unchecked	Unchecked	Unchecked	Unchecked	Unchecked	Unchecked
Unchecked	Unchecked	Unchecked	Unchecked	Unchecked	Unchecked	Unchecked
Unchecked	Unchecked	Unchecked	Unchecked	Checked	Unchecked	Unchecked
Unchecked	Unchecked	Unchecked	Unchecked	Unchecked	Checked	Unchecked
Unchecked	Unchecked	Unchecked	Unchecked	Unchecked	Unchecked	Unchecked
Unchecked	Unchecked	Unchecked	Unchecked	Unchecked	Unchecked	Unchecked
Unchecked	Unchecked	Unchecked	Unchecked	Unchecked	Unchecked	Unchecked
Unchecked	Unchecked	Unchecked	Unchecked	Unchecked	Unchecked	Unchecked
Unchecked	Unchecked	Unchecked	Unchecked	Unchecked	Unchecked	Unchecked
Unchecked	Unchecked	Checked	Unchecked	Unchecked	Unchecked	Unchecked
Unchecked	Unchecked	Unchecked	Unchecked	Unchecked	Unchecked	Unchecked
Unchecked	Unchecked	Unchecked	Unchecked	Checked	Unchecked	Unchecked
Unchecked	Unchecked	Unchecked	Unchecked	Unchecked	Unchecked	Unchecked
Unchecked	Unchecked	Unchecked	Unchecked	Unchecked	Unchecked	Unchecked
Unchecked	Unchecked	Unchecked	Unchecked	Unchecked	Unchecked	Unchecked
Unchecked	Unchecked	Unchecked	Unchecked	Unchecked	Unchecked	Unchecked
Unchecked	Unchecked	Unchecked	Unchecked	Unchecked	Unchecked	Unchecked
Unchecked	Unchecked	Unchecked	Unchecked	Unchecked	Unchecked	Unchecked
Unchecked	Unchecked	Unchecked	Unchecked	Unchecked	Unchecked	Unchecked
Unchecked	Unchecked	Unchecked	Unchecked	Unchecked	Unchecked	Unchecked
Unchecked	Unchecked	Unchecked	Unchecked	Unchecked	Unchecked	Unchecked
Unchecked	Unchecked	Unchecked	Unchecked	Unchecked	Unchecked	Unchecked
Unchecked	Unchecked	Unchecked	Unchecked	Unchecked	Unchecked	Unchecked
Unchecked	Unchecked	Unchecked	Unchecked	Unchecked	Unchecked	Unchecked

Unchecked	Unchecked	Checked	Unchecked	Unchecked	Checked	Unchecked
Unchecked	Unchecked	Unchecked	Unchecked	Unchecked	Unchecked	Unchecked
Unchecked	Unchecked	Unchecked	Unchecked	Unchecked	Unchecked	Unchecked
Unchecked	Checked	Unchecked	Unchecked	Checked	Unchecked	Unchecked
Unchecked	Unchecked	Unchecked	Unchecked	Unchecked	Unchecked	Unchecked
Unchecked	Unchecked	Unchecked	Unchecked	Checked	Unchecked	Unchecked
Unchecked	Unchecked	Unchecked	Unchecked	Unchecked	Unchecked	Unchecked
Unchecked	Unchecked	Unchecked	Unchecked	Unchecked	Unchecked	Unchecked
Unchecked	Unchecked	Unchecked	Unchecked	Unchecked	Unchecked	Unchecked
Unchecked	Unchecked	Unchecked	Unchecked	Unchecked	Unchecked	Unchecked
Unchecked	Unchecked	Unchecked	Unchecked	Unchecked	Unchecked	Unchecked
Unchecked	Unchecked	Unchecked	Unchecked	Unchecked	Unchecked	Unchecked
Unchecked	Unchecked	Unchecked	Unchecked	Unchecked	Unchecked	Unchecked
Unchecked	Unchecked	Unchecked	Unchecked	Checked	Unchecked	Unchecked
Unchecked	Unchecked	Unchecked	Unchecked	Unchecked	Unchecked	Unchecked
Unchecked	Unchecked	Unchecked	Unchecked	Unchecked	Unchecked	Unchecked
Unchecked	Checked	Unchecked	Unchecked	Unchecked	Unchecked	Unchecked
Unchecked	Unchecked	Unchecked	Unchecked	Unchecked	Unchecked	Unchecked
Unchecked	Unchecked	Unchecked	Unchecked	Unchecked	Unchecked	Unchecked
Unchecked	Unchecked	Unchecked	Unchecked	Unchecked	Unchecked	Unchecked
Unchecked	Checked	Unchecked	Unchecked	Unchecked	Unchecked	Unchecked
Unchecked	Unchecked	Unchecked	Unchecked	Unchecked	Unchecked	Unchecked
Unchecked	Unchecked	Unchecked	Unchecked	Unchecked	Unchecked	Unchecked
Unchecked	Unchecked	Unchecked	Unchecked	Unchecked	Unchecked	Unchecked
Unchecked	Checked	Unchecked	Unchecked	Unchecked	Unchecked	Unchecked
Unchecked	Unchecked	Unchecked	Unchecked	Unchecked	Unchecked	Unchecked
Unchecked	Unchecked	Unchecked	Checked	Unchecked	Unchecked	Unchecked
Unchecked	Unchecked	Unchecked	Unchecked	Unchecked	Unchecked	Unchecked
Unchecked	Checked	Unchecked	Unchecked	Unchecked	Unchecked	Unchecked
Unchecked	Unchecked	Checked	Unchecked	Checked	Unchecked	Unchecked
Unchecked	Unchecked	Unchecked	Unchecked	Unchecked	Unchecked	Unchecked
Unchecked	Checked	Unchecked	Unchecked	Unchecked	Unchecked	Unchecked
Unchecked	Checked	Unchecked	Unchecked	Unchecked	Unchecked	Unchecked
Unchecked	Unchecked	Unchecked	Unchecked	Unchecked	Unchecked	Unchecked
Unchecked	Unchecked	Unchecked	Unchecked	Unchecked	Unchecked	Unchecked
Unchecked	Unchecked	Unchecked	Unchecked	Unchecked	Unchecked	Unchecked
Unchecked	Unchecked	Unchecked	Unchecked	Unchecked	Unchecked	Unchecked
Unchecked	Unchecked	Unchecked	Unchecked	Unchecked	Unchecked	Unchecked
Unchecked	Unchecked	Unchecked	Unchecked	Unchecked	Unchecked	Unchecked
Unchecked	Unchecked	Unchecked	Unchecked	Unchecked	Unchecked	Unchecked
Unchecked	Unchecked	Unchecked	Unchecked	Unchecked	Unchecked	Unchecked
Unchecked	Unchecked	Unchecked	Unchecked	Unchecked	Unchecked	Unchecked
Unchecked	Unchecked	Unchecked	Unchecked	Unchecked	Unchecked	Unchecked

Unchecked	Unchecked	Unchecked	Unchecked	Unchecked	Unchecked	Unchecked
Unchecked	Unchecked	Unchecked	Unchecked	Unchecked	Unchecked	Unchecked
Unchecked	Unchecked	Unchecked	Unchecked	Checked	Unchecked	Unchecked
Unchecked	Unchecked	Unchecked	Unchecked	Unchecked	Unchecked	Unchecked
Unchecked	Unchecked	Unchecked	Unchecked	Unchecked	Unchecked	Unchecked
Unchecked	Unchecked	Unchecked	Unchecked	Unchecked	Unchecked	Unchecked
Unchecked	Unchecked	Checked	Unchecked	Unchecked	Unchecked	Unchecked
Unchecked	Unchecked	Unchecked	Unchecked	Unchecked	Unchecked	Unchecked
Unchecked	Unchecked	Unchecked	Unchecked	Unchecked	Unchecked	Unchecked
Unchecked	Unchecked	Unchecked	Unchecked	Unchecked	Unchecked	Unchecked
Unchecked	Unchecked	Unchecked	Unchecked	Unchecked	Unchecked	Unchecked
Unchecked	Unchecked	Unchecked	Unchecked	Unchecked	Unchecked	Unchecked
Unchecked	Unchecked	Unchecked	Unchecked	Unchecked	Unchecked	Unchecked
Unchecked	Unchecked	Unchecked	Unchecked	Unchecked	Unchecked	Unchecked
Unchecked	Checked	Unchecked	Unchecked	Unchecked	Unchecked	Unchecked
Unchecked	Unchecked	Unchecked	Unchecked	Checked	Unchecked	Unchecked
Unchecked	Unchecked	Unchecked	Unchecked	Unchecked	Unchecked	Unchecked
Unchecked	Unchecked	Unchecked	Unchecked	Unchecked	Unchecked	Unchecked
Unchecked	Unchecked	Unchecked	Unchecked	Unchecked	Unchecked	Unchecked
Unchecked	Unchecked	Unchecked	Unchecked	Unchecked	Unchecked	Unchecked
Unchecked	Unchecked	Unchecked	Unchecked	Unchecked	Unchecked	Unchecked
Unchecked	Unchecked	Unchecked	Unchecked	Unchecked	Unchecked	Unchecked
Unchecked	Unchecked	Unchecked	Unchecked	Unchecked	Unchecked	Unchecked
Unchecked	Unchecked	Unchecked	Unchecked	Unchecked	Unchecked	Unchecked
Unchecked	Unchecked	Unchecked	Unchecked	Unchecked	Unchecked	Unchecked
Unchecked	Unchecked	Unchecked	Unchecked	Unchecked	Unchecked	Unchecked
Unchecked	Checked	Unchecked	Unchecked	Unchecked	Unchecked	Unchecked
Unchecked	Unchecked	Unchecked	Unchecked	Unchecked	Unchecked	Unchecked
Unchecked	Unchecked	Unchecked	Unchecked	Unchecked	Unchecked	Unchecked
Unchecked	Unchecked	Unchecked	Unchecked	Unchecked	Unchecked	Unchecked
Unchecked	Unchecked	Unchecked	Unchecked	Checked	Unchecked	Unchecked
Unchecked	Unchecked	Unchecked	Unchecked	Unchecked	Unchecked	Unchecked
Unchecked	Unchecked	Unchecked	Unchecked	Unchecked	Unchecked	Unchecked
Unchecked	Unchecked	Unchecked	Unchecked	Unchecked	Unchecked	Unchecked
Unchecked	Unchecked	Unchecked	Unchecked	Unchecked	Unchecked	Unchecked
Unchecked	Unchecked	Unchecked	Unchecked	Unchecked	Unchecked	Unchecked
Unchecked	Unchecked	Checked	Unchecked	Unchecked	Unchecked	Unchecked
Unchecked	Unchecked	Unchecked	Unchecked	Unchecked	Unchecked	Unchecked
Unchecked	Unchecked	Unchecked	Unchecked	Unchecked	Unchecked	Unchecked
Unchecked	Unchecked	Unchecked	Unchecked	Unchecked	Unchecked	Unchecked
Unchecked	Unchecked	Unchecked	Unchecked	Unchecked	Unchecked	Unchecked
Unchecked	Unchecked	Unchecked	Unchecked	Checked	Unchecked	Unchecked
Unchecked	Unchecked	Unchecked	Unchecked	Unchecked	Unchecked	Unchecked

Unchecked	Unchecked	Unchecked	Unchecked	Unchecked	Unchecked	Unchecked
Unchecked	Unchecked	Unchecked	Unchecked	Unchecked	Unchecked	Unchecked
Unchecked	Unchecked	Unchecked	Unchecked	Unchecked	Unchecked	Unchecked
Unchecked	Checked	Checked	Unchecked	Unchecked	Unchecked	Unchecked
Unchecked	Unchecked	Unchecked	Unchecked	Unchecked	Unchecked	Unchecked
Unchecked	Unchecked	Unchecked	Unchecked	Unchecked	Unchecked	Unchecked
Unchecked	Unchecked	Unchecked	Unchecked	Unchecked	Unchecked	Unchecked
Unchecked	Unchecked	Unchecked	Unchecked	Unchecked	Unchecked	Unchecked
Unchecked	Unchecked	Unchecked	Unchecked	Unchecked	Unchecked	Unchecked
Unchecked	Unchecked	Unchecked	Unchecked	Unchecked	Unchecked	Unchecked
Checked	Unchecked	Unchecked	Unchecked	Unchecked	Unchecked	Unchecked
Unchecked	Unchecked	Unchecked	Unchecked	Unchecked	Unchecked	Unchecked
Unchecked	Unchecked	Unchecked	Unchecked	Checked	Unchecked	Unchecked
Unchecked	Unchecked	Unchecked	Unchecked	Unchecked	Unchecked	Unchecked
Unchecked	Unchecked	Unchecked	Unchecked	Unchecked	Unchecked	Unchecked
Unchecked	Unchecked	Unchecked	Unchecked	Unchecked	Unchecked	Unchecked
Unchecked	Checked	Unchecked	Unchecked	Unchecked	Unchecked	Unchecked
Unchecked	Unchecked	Unchecked	Unchecked	Unchecked	Unchecked	Unchecked
Unchecked	Unchecked	Unchecked	Unchecked	Unchecked	Unchecked	Unchecked
Unchecked	Unchecked	Unchecked	Unchecked	Unchecked	Unchecked	Unchecked
Unchecked	Unchecked	Unchecked	Unchecked	Unchecked	Unchecked	Unchecked
Unchecked	Unchecked	Unchecked	Unchecked	Unchecked	Unchecked	Unchecked
Unchecked	Unchecked	Unchecked	Unchecked	Unchecked	Unchecked	Unchecked
Unchecked	Unchecked	Unchecked	Unchecked	Unchecked	Unchecked	Unchecked
Unchecked	Unchecked	Unchecked	Unchecked	Unchecked	Unchecked	Unchecked
Unchecked	Unchecked	Unchecked	Unchecked	Unchecked	Unchecked	Unchecked
Unchecked	Unchecked	Unchecked	Unchecked	Unchecked	Unchecked	Unchecked
Unchecked	Unchecked	Unchecked	Unchecked	Unchecked	Unchecked	Unchecked
Unchecked	Unchecked	Unchecked	Unchecked	Unchecked	Unchecked	Unchecked
Unchecked	Unchecked	Unchecked	Unchecked	Checked	Unchecked	Unchecked
Unchecked	Unchecked	Unchecked	Unchecked	Unchecked	Unchecked	Unchecked
Unchecked	Unchecked	Unchecked	Unchecked	Unchecked	Unchecked	Unchecked
Unchecked	Unchecked	Unchecked	Unchecked	Unchecked	Unchecked	Unchecked
Unchecked	Unchecked	Unchecked	Unchecked	Unchecked	Unchecked	Unchecked
Unchecked	Checked	Checked	Unchecked	Checked	Unchecked	Unchecked
Unchecked	Unchecked	Unchecked	Unchecked	Unchecked	Unchecked	Unchecked
Unchecked	Unchecked	Unchecked	Unchecked	Unchecked	Unchecked	Unchecked
Unchecked	Unchecked	Unchecked	Unchecked	Unchecked	Unchecked	Unchecked
Unchecked	Unchecked	Checked	Unchecked	Checked	Unchecked	Unchecked
Unchecked	Unchecked	Unchecked	Unchecked	Unchecked	Unchecked	Unchecked
Unchecked	Unchecked	Unchecked	Unchecked	Unchecked	Unchecked	Unchecked
Unchecked	Unchecked	Unchecked	Unchecked	Unchecked	Unchecked	Unchecked
Unchecked	Unchecked	Checked	Unchecked	Unchecked	Unchecked	Unchecked

Unchecked	Unchecked	Checked	Unchecked	Unchecked	Unchecked	Unchecked
Unchecked	Unchecked	Unchecked	Unchecked	Unchecked	Unchecked	Unchecked
Unchecked	Unchecked	Unchecked	Unchecked	Unchecked	Unchecked	Unchecked
Unchecked	Unchecked	Unchecked	Unchecked	Unchecked	Unchecked	Unchecked
Unchecked	Unchecked	Unchecked	Unchecked	Unchecked	Unchecked	Unchecked
Unchecked	Unchecked	Unchecked	Unchecked	Unchecked	Unchecked	Unchecked
Unchecked	Unchecked	Unchecked	Unchecked	Unchecked	Unchecked	Unchecked
Unchecked	Unchecked	Unchecked	Unchecked	Unchecked	Unchecked	Unchecked
Unchecked	Unchecked	Unchecked	Unchecked	Unchecked	Unchecked	Unchecked
Unchecked	Unchecked	Unchecked	Unchecked	Unchecked	Unchecked	Unchecked
Unchecked	Unchecked	Unchecked	Unchecked	Unchecked	Unchecked	Unchecked
Unchecked	Unchecked	Checked	Unchecked	Unchecked	Unchecked	Unchecked
Unchecked	Unchecked	Unchecked	Unchecked	Checked	Unchecked	Unchecked
Unchecked	Unchecked	Unchecked	Unchecked	Unchecked	Unchecked	Unchecked
Unchecked	Unchecked	Unchecked	Unchecked	Unchecked	Unchecked	Unchecked
Unchecked	Unchecked	Checked	Unchecked	Unchecked	Unchecked	Unchecked
Unchecked	Unchecked	Unchecked	Unchecked	Unchecked	Unchecked	Unchecked
Unchecked	Unchecked	Unchecked	Unchecked	Unchecked	Unchecked	Unchecked
Unchecked	Unchecked	Unchecked	Unchecked	Unchecked	Unchecked	Unchecked
Unchecked	Unchecked	Checked	Unchecked	Checked	Unchecked	Unchecked
Unchecked	Unchecked	Checked	Unchecked	Unchecked	Unchecked	Unchecked
Unchecked	Unchecked	Unchecked	Unchecked	Unchecked	Unchecked	Unchecked
Unchecked	Unchecked	Unchecked	Unchecked	Unchecked	Unchecked	Unchecked
Unchecked	Unchecked	Unchecked	Unchecked	Unchecked	Unchecked	Unchecked
Unchecked	Unchecked	Unchecked	Unchecked	Unchecked	Unchecked	Unchecked
Unchecked	Unchecked	Unchecked	Unchecked	Unchecked	Unchecked	Unchecked
Unchecked	Unchecked	Unchecked	Unchecked	Unchecked	Unchecked	Unchecked
Unchecked	Unchecked	Unchecked	Unchecked	Unchecked	Unchecked	Unchecked
Unchecked	Unchecked	Unchecked	Unchecked	Unchecked	Unchecked	Unchecked
Unchecked	Unchecked	Unchecked	Unchecked	Unchecked	Unchecked	Unchecked
Unchecked	Unchecked	Unchecked	Unchecked	Unchecked	Unchecked	Unchecked
Unchecked	Unchecked	Unchecked	Unchecked	Unchecked	Unchecked	Unchecked
Unchecked	Unchecked	Unchecked	Unchecked	Unchecked	Unchecked	Unchecked
Unchecked	Unchecked	Unchecked	Unchecked	Unchecked	Unchecked	Unchecked
Unchecked	Unchecked	Unchecked	Unchecked	Unchecked	Unchecked	Unchecked
Unchecked	Unchecked	Checked	Unchecked	Unchecked	Unchecked	Unchecked
Unchecked	Unchecked	Checked	Unchecked	Checked	Unchecked	Unchecked
Unchecked	Unchecked	Unchecked	Unchecked	Unchecked	Unchecked	Unchecked
Unchecked	Checked	Unchecked	Unchecked	Unchecked	Unchecked	Unchecked
Unchecked	Unchecked	Unchecked	Unchecked	Unchecked	Unchecked	Unchecked
Unchecked	Unchecked	Unchecked	Unchecked	Unchecked	Unchecked	Unchecked
Unchecked	Unchecked	Unchecked	Checked	Unchecked	Unchecked	Unchecked
Unchecked	Unchecked	Unchecked	Unchecked	Unchecked	Unchecked	Unchecked

Unchecked	Unchecked	Unchecked	Unchecked	Unchecked	Unchecked	Unchecked
Unchecked	Unchecked	Unchecked	Unchecked	Unchecked	Unchecked	Unchecked
Unchecked	Unchecked	Unchecked	Unchecked	Unchecked	Unchecked	Unchecked
Unchecked	Unchecked	Unchecked	Unchecked	Unchecked	Unchecked	Unchecked
Unchecked	Unchecked	Unchecked	Unchecked	Unchecked	Unchecked	Unchecked
Unchecked	Unchecked	Unchecked	Unchecked	Unchecked	Unchecked	Unchecked
Unchecked	Unchecked	Unchecked	Unchecked	Unchecked	Unchecked	Unchecked
Unchecked	Unchecked	Unchecked	Unchecked	Unchecked	Unchecked	Unchecked
Unchecked	Unchecked	Unchecked	Unchecked	Unchecked	Unchecked	Unchecked
Unchecked	Unchecked	Unchecked	Unchecked	Unchecked	Unchecked	Unchecked
Unchecked	Unchecked	Unchecked	Unchecked	Unchecked	Unchecked	Unchecked
Checked	Unchecked	Unchecked	Checked	Checked	Unchecked	Unchecked
Unchecked	Unchecked	Unchecked	Checked	Unchecked	Unchecked	Unchecked
Unchecked	Unchecked	Unchecked	Unchecked	Unchecked	Unchecked	Unchecked
Unchecked	Unchecked	Unchecked	Unchecked	Unchecked	Unchecked	Unchecked
Unchecked	Unchecked	Unchecked	Unchecked	Unchecked	Unchecked	Unchecked
Unchecked	Unchecked	Unchecked	Unchecked	Unchecked	Unchecked	Unchecked
Unchecked	Unchecked	Unchecked	Unchecked	Checked	Unchecked	Unchecked
Unchecked	Unchecked	Unchecked	Unchecked	Unchecked	Unchecked	Unchecked
Unchecked	Unchecked	Unchecked	Unchecked	Unchecked	Unchecked	Unchecked
Unchecked	Unchecked	Unchecked	Unchecked	Unchecked	Unchecked	Unchecked
Unchecked	Unchecked	Unchecked	Unchecked	Unchecked	Unchecked	Unchecked
Unchecked	Unchecked	Unchecked	Unchecked	Unchecked	Unchecked	Unchecked
Unchecked	Unchecked	Unchecked	Unchecked	Checked	Unchecked	Unchecked
Unchecked	Unchecked	Unchecked	Unchecked	Checked	Unchecked	Unchecked
Unchecked	Unchecked	Unchecked	Unchecked	Unchecked	Unchecked	Unchecked
Unchecked	Unchecked	Unchecked	Unchecked	Unchecked	Unchecked	Unchecked
Unchecked	Unchecked	Unchecked	Unchecked	Unchecked	Unchecked	Unchecked
Unchecked	Unchecked	Unchecked	Unchecked	Unchecked	Unchecked	Unchecked
Unchecked	Unchecked	Checked	Unchecked	Unchecked	Unchecked	Unchecked
Unchecked	Unchecked	Unchecked	Unchecked	Unchecked	Unchecked	Unchecked
Unchecked	Unchecked	Unchecked	Unchecked	Unchecked	Unchecked	Unchecked
Unchecked	Unchecked	Unchecked	Unchecked	Unchecked	Unchecked	Unchecked
Unchecked	Unchecked	Unchecked	Checked	Unchecked	Unchecked	Unchecked
Unchecked	Unchecked	Unchecked	Unchecked	Unchecked	Unchecked	Unchecked
Unchecked	Unchecked	Checked	Unchecked	Unchecked	Unchecked	Unchecked
Unchecked	Unchecked	Unchecked	Unchecked	Unchecked	Unchecked	Unchecked
Unchecked	Unchecked	Unchecked	Checked	Unchecked	Unchecked	Unchecked
Unchecked	Unchecked	Unchecked	Unchecked	Unchecked	Unchecked	Unchecked
Unchecked	Unchecked	Unchecked	Unchecked	Unchecked	Unchecked	Unchecked
Unchecked	Unchecked	Unchecked	Unchecked	Unchecked	Unchecked	Unchecked
Unchecked	Unchecked	Unchecked	Unchecked	Unchecked	Unchecked	Unchecked
Unchecked	Unchecked	Checked	Unchecked	Unchecked	Unchecked	Unchecked

Unchecked	Checked	Checked	Unchecked	Unchecked	Unchecked	Unchecked
Unchecked	Unchecked	Unchecked	Unchecked	Unchecked	Unchecked	Unchecked
Unchecked	Unchecked	Checked	Checked	Checked	Unchecked	Unchecked
Unchecked	Unchecked	Unchecked	Unchecked	Unchecked	Unchecked	Unchecked
Unchecked	Checked	Checked	Unchecked	Unchecked	Unchecked	Unchecked
Unchecked	Unchecked	Unchecked	Unchecked	Unchecked	Unchecked	Unchecked
Unchecked	Unchecked	Unchecked	Unchecked	Checked	Unchecked	Unchecked
Unchecked	Unchecked	Unchecked	Unchecked	Checked	Unchecked	Unchecked
Unchecked	Unchecked	Unchecked	Unchecked	Checked	Unchecked	Unchecked
Unchecked	Unchecked	Unchecked	Unchecked	Unchecked	Unchecked	Unchecked
Unchecked	Unchecked	Unchecked	Unchecked	Unchecked	Unchecked	Unchecked
Unchecked	Unchecked	Unchecked	Unchecked	Unchecked	Unchecked	Unchecked
Unchecked	Unchecked	Unchecked	Unchecked	Unchecked	Unchecked	Unchecked
Unchecked	Unchecked	Unchecked	Unchecked	Unchecked	Unchecked	Unchecked
Unchecked	Unchecked	Unchecked	Unchecked	Unchecked	Unchecked	Unchecked
Unchecked	Unchecked	Unchecked	Unchecked	Unchecked	Unchecked	Unchecked
Unchecked	Unchecked	Unchecked	Unchecked	Unchecked	Unchecked	Unchecked
Unchecked	Unchecked	Unchecked	Unchecked	Unchecked	Unchecked	Unchecked
Unchecked	Unchecked	Unchecked	Unchecked	Unchecked	Unchecked	Unchecked
Unchecked	Unchecked	Unchecked	Unchecked	Unchecked	Unchecked	Unchecked
Unchecked	Unchecked	Unchecked	Unchecked	Unchecked	Unchecked	Unchecked
Unchecked	Unchecked	Unchecked	Unchecked	Unchecked	Unchecked	Unchecked
Unchecked	Unchecked	Unchecked	Unchecked	Unchecked	Unchecked	Unchecked
Unchecked	Unchecked	Unchecked	Unchecked	Unchecked	Unchecked	Unchecked
Unchecked	Unchecked	Unchecked	Unchecked	Unchecked	Unchecked	Unchecked
Unchecked	Unchecked	Unchecked	Unchecked	Unchecked	Unchecked	Unchecked
Unchecked	Unchecked	Unchecked	Unchecked	Unchecked	Unchecked	Unchecked
Unchecked	Unchecked	Unchecked	Unchecked	Unchecked	Unchecked	Unchecked
Unchecked	Unchecked	Unchecked	Unchecked	Unchecked	Unchecked	Unchecked
Unchecked	Unchecked	Unchecked	Unchecked	Unchecked	Unchecked	Unchecked
Unchecked	Unchecked	Unchecked	Unchecked	Unchecked	Unchecked	Unchecked
Unchecked	Unchecked	Unchecked	Unchecked	Unchecked	Unchecked	Unchecked
Unchecked	Unchecked	Unchecked	Unchecked	Unchecked	Unchecked	Unchecked
Unchecked	Unchecked	Unchecked	Unchecked	Unchecked	Unchecked	Unchecked
Unchecked	Unchecked	Unchecked	Unchecked	Checked	Unchecked	Unchecked
Unchecked	Unchecked	Unchecked	Unchecked	Unchecked	Unchecked	Unchecked
Unchecked	Unchecked	Unchecked	Unchecked	Unchecked	Unchecked	Unchecked
Unchecked	Unchecked	Checked	Unchecked	Unchecked	Unchecked	Unchecked
Unchecked	Unchecked	Unchecked	Unchecked	Checked	Unchecked	Unchecked
Unchecked	Unchecked	Unchecked	Unchecked	Checked	Unchecked	Unchecked
Unchecked	Unchecked	Unchecked	Unchecked	Unchecked	Unchecked	Unchecked
Unchecked	Unchecked	Unchecked	Unchecked	Unchecked	Unchecked	Unchecked
Unchecked	Unchecked	Unchecked	Unchecked	Unchecked	Unchecked	Unchecked

Unchecked	Unchecked	Unchecked	Unchecked	Unchecked	Unchecked	Unchecked
Unchecked	Unchecked	Unchecked	Unchecked	Unchecked	Unchecked	Unchecked
Unchecked	Unchecked	Unchecked	Unchecked	Unchecked	Unchecked	Unchecked
Unchecked	Unchecked	Unchecked	Unchecked	Unchecked	Unchecked	Unchecked
Unchecked	Unchecked	Unchecked	Unchecked	Unchecked	Unchecked	Unchecked
Unchecked	Unchecked	Unchecked	Unchecked	Unchecked	Unchecked	Unchecked
Unchecked	Unchecked	Unchecked	Unchecked	Unchecked	Unchecked	Unchecked
Unchecked	Unchecked	Unchecked	Unchecked	Unchecked	Unchecked	Unchecked
Unchecked	Unchecked	Unchecked	Unchecked	Unchecked	Unchecked	Unchecked
Unchecked	Unchecked	Unchecked	Unchecked	Unchecked	Unchecked	Unchecked
Unchecked	Unchecked	Unchecked	Unchecked	Unchecked	Unchecked	Unchecked
Unchecked	Unchecked	Unchecked	Unchecked	Unchecked	Unchecked	Unchecked
Unchecked	Unchecked	Unchecked	Unchecked	Unchecked	Unchecked	Unchecked
Unchecked	Unchecked	Unchecked	Unchecked	Unchecked	Unchecked	Unchecked
Unchecked	Unchecked	Unchecked	Unchecked	Unchecked	Unchecked	Unchecked
Unchecked	Unchecked	Unchecked	Unchecked	Unchecked	Unchecked	Unchecked
Unchecked	Unchecked	Unchecked	Unchecked	Unchecked	Unchecked	Unchecked
Unchecked	Unchecked	Unchecked	Unchecked	Unchecked	Unchecked	Unchecked
Unchecked	Unchecked	Unchecked	Unchecked	Unchecked	Unchecked	Unchecked
Unchecked	Unchecked	Checked	Unchecked	Unchecked	Unchecked	Unchecked
Unchecked	Unchecked	Unchecked	Unchecked	Unchecked	Unchecked	Unchecked
Unchecked	Unchecked	Unchecked	Unchecked	Unchecked	Unchecked	Unchecked
Unchecked	Unchecked	Unchecked	Unchecked	Unchecked	Unchecked	Unchecked
Unchecked	Unchecked	Unchecked	Unchecked	Unchecked	Unchecked	Unchecked
Unchecked	Unchecked	Unchecked	Unchecked	Unchecked	Unchecked	Unchecked
Unchecked	Unchecked	Unchecked	Unchecked	Unchecked	Unchecked	Unchecked
Unchecked	Unchecked	Unchecked	Unchecked	Unchecked	Unchecked	Unchecked
Unchecked	Unchecked	Unchecked	Unchecked	Unchecked	Unchecked	Unchecked
Unchecked	Unchecked	Unchecked	Unchecked	Unchecked	Unchecked	Unchecked
Unchecked	Unchecked	Unchecked	Unchecked	Unchecked	Checked	Unchecked
Unchecked	Unchecked	Unchecked	Unchecked	Unchecked	Unchecked	Unchecked
Unchecked	Unchecked	Unchecked	Unchecked	Checked	Unchecked	Unchecked
Unchecked	Unchecked	Unchecked	Unchecked	Unchecked	Unchecked	Unchecked
Unchecked	Unchecked	Unchecked	Unchecked	Unchecked	Unchecked	Unchecked
Unchecked	Unchecked	Unchecked	Unchecked	Unchecked	Unchecked	Unchecked
Unchecked	Unchecked	Unchecked	Unchecked	Unchecked	Unchecked	Unchecked
Unchecked	Unchecked	Unchecked	Unchecked	Unchecked	Unchecked	Unchecked
Unchecked	Unchecked	Checked	Unchecked	Unchecked	Unchecked	Unchecked
Unchecked	Unchecked	Checked	Unchecked	Checked	Unchecked	Unchecked
Unchecked	Unchecked	Checked	Unchecked	Unchecked	Unchecked	Unchecked
Unchecked	Unchecked	Checked	Unchecked	Unchecked	Unchecked	Unchecked
Unchecked	Unchecked	Unchecked	Unchecked	Unchecked	Unchecked	Unchecked
Unchecked	Unchecked	Unchecked	Unchecked	Unchecked	Unchecked	Unchecked

Unchecked	Unchecked	Unchecked	Unchecked	Unchecked	Unchecked	Unchecked
Unchecked	Unchecked	Unchecked	Unchecked	Unchecked	Unchecked	Unchecked
Unchecked	Unchecked	Unchecked	Unchecked	Unchecked	Unchecked	Unchecked
Unchecked	Unchecked	Unchecked	Checked	Unchecked	Unchecked	Unchecked
Unchecked	Unchecked	Unchecked	Unchecked	Unchecked	Unchecked	Unchecked
Unchecked	Unchecked	Unchecked	Checked	Unchecked	Unchecked	Unchecked
Unchecked	Unchecked	Unchecked	Unchecked	Unchecked	Unchecked	Unchecked
Unchecked	Unchecked	Unchecked	Checked	Unchecked	Unchecked	Unchecked
Unchecked	Unchecked	Unchecked	Unchecked	Unchecked	Unchecked	Unchecked
Unchecked	Unchecked	Unchecked	Unchecked	Unchecked	Unchecked	Unchecked
Unchecked	Unchecked	Unchecked	Unchecked	Unchecked	Unchecked	Unchecked
Unchecked	Unchecked	Unchecked	Unchecked	Unchecked	Unchecked	Unchecked
Unchecked	Unchecked	Unchecked	Unchecked	Unchecked	Unchecked	Unchecked
Unchecked	Unchecked	Unchecked	Unchecked	Unchecked	Unchecked	Unchecked
Unchecked	Unchecked	Unchecked	Unchecked	Unchecked	Unchecked	Unchecked
Unchecked	Unchecked	Unchecked	Unchecked	Unchecked	Unchecked	Unchecked
Unchecked	Unchecked	Unchecked	Unchecked	Unchecked	Unchecked	Unchecked
Unchecked	Unchecked	Unchecked	Unchecked	Unchecked	Unchecked	Unchecked
Unchecked	Unchecked	Unchecked	Unchecked	Unchecked	Unchecked	Unchecked
Unchecked	Unchecked	Unchecked	Unchecked	Unchecked	Unchecked	Unchecked
Unchecked	Unchecked	Unchecked	Unchecked	Unchecked	Unchecked	Unchecked
Unchecked	Unchecked	Unchecked	Unchecked	Checked	Unchecked	Unchecked
Unchecked	Unchecked	Unchecked	Unchecked	Unchecked	Unchecked	Unchecked
Checked	Unchecked	Checked	Checked	Unchecked	Unchecked	Unchecked
Unchecked	Unchecked	Unchecked	Unchecked	Unchecked	Unchecked	Unchecked
Unchecked	Unchecked	Unchecked	Unchecked	Unchecked	Unchecked	Unchecked
Unchecked	Unchecked	Unchecked	Unchecked	Unchecked	Unchecked	Unchecked
Unchecked	Unchecked	Unchecked	Unchecked	Unchecked	Unchecked	Unchecked
Unchecked	Unchecked	Unchecked	Unchecked	Unchecked	Unchecked	Unchecked
Unchecked	Unchecked	Unchecked	Unchecked	Unchecked	Unchecked	Unchecked
Unchecked	Unchecked	Unchecked	Unchecked	Unchecked	Unchecked	Unchecked
Unchecked	Unchecked	Unchecked	Unchecked	Unchecked	Unchecked	Unchecked
Unchecked	Checked	Unchecked	Unchecked	Unchecked	Unchecked	Unchecked
Unchecked	Unchecked	Unchecked	Unchecked	Unchecked	Unchecked	Unchecked
Unchecked	Unchecked	Unchecked	Unchecked	Unchecked	Unchecked	Unchecked
Unchecked	Unchecked	Unchecked	Checked	Unchecked	Unchecked	Unchecked
Unchecked	Unchecked	Unchecked	Unchecked	Checked	Unchecked	Unchecked
Unchecked	Unchecked	Checked	Unchecked	Unchecked	Unchecked	Unchecked
Unchecked	Unchecked	Unchecked	Unchecked	Unchecked	Unchecked	Unchecked
Unchecked	Checked	Unchecked	Unchecked	Unchecked	Unchecked	Unchecked
Unchecked	Unchecked	Unchecked	Unchecked	Unchecked	Unchecked	Unchecked
Unchecked	Unchecked	Unchecked	Unchecked	Unchecked	Unchecked	Unchecked
Unchecked	Unchecked	Unchecked	Unchecked	Unchecked	Unchecked	Unchecked

Unchecked	Unchecked	Unchecked	Unchecked	Unchecked	Unchecked	Unchecked
Unchecked	Unchecked	Unchecked	Unchecked	Unchecked	Unchecked	Unchecked
Unchecked	Unchecked	Unchecked	Unchecked	Unchecked	Unchecked	Unchecked
Unchecked	Unchecked	Unchecked	Unchecked	Unchecked	Unchecked	Unchecked
Unchecked	Unchecked	Unchecked	Unchecked	Unchecked	Unchecked	Unchecked
Unchecked	Unchecked	Unchecked	Unchecked	Unchecked	Unchecked	Unchecked
Unchecked	Unchecked	Unchecked	Unchecked	Unchecked	Unchecked	Unchecked
Unchecked	Unchecked	Unchecked	Unchecked	Checked	Unchecked	Unchecked
Unchecked	Unchecked	Unchecked	Unchecked	Unchecked	Unchecked	Unchecked
Unchecked	Unchecked	Unchecked	Unchecked	Unchecked	Unchecked	Unchecked
Unchecked	Unchecked	Checked	Unchecked	Unchecked	Unchecked	Unchecked
Unchecked	Unchecked	Unchecked	Unchecked	Unchecked	Unchecked	Unchecked
Unchecked	Unchecked	Unchecked	Unchecked	Unchecked	Unchecked	Unchecked
Unchecked	Unchecked	Unchecked	Unchecked	Unchecked	Unchecked	Unchecked
Unchecked	Unchecked	Checked	Unchecked	Unchecked	Unchecked	Unchecked
Unchecked	Unchecked	Unchecked	Unchecked	Unchecked	Unchecked	Unchecked
Unchecked	Unchecked	Unchecked	Unchecked	Unchecked	Unchecked	Unchecked
Unchecked	Unchecked	Unchecked	Unchecked	Unchecked	Unchecked	Unchecked
Unchecked	Unchecked	Unchecked	Unchecked	Checked	Unchecked	Unchecked
Unchecked	Unchecked	Unchecked	Unchecked	Unchecked	Unchecked	Unchecked
Unchecked	Unchecked	Checked	Unchecked	Unchecked	Unchecked	Unchecked
Unchecked	Unchecked	Unchecked	Unchecked	Unchecked	Unchecked	Unchecked
Unchecked	Unchecked	Checked	Unchecked	Unchecked	Unchecked	Unchecked
Unchecked	Unchecked	Unchecked	Unchecked	Unchecked	Unchecked	Unchecked
Unchecked	Unchecked	Unchecked	Unchecked	Unchecked	Unchecked	Unchecked
Unchecked	Unchecked	Unchecked	Checked	Unchecked	Unchecked	Unchecked
Unchecked	Unchecked	Unchecked	Unchecked	Unchecked	Unchecked	Unchecked
Unchecked	Unchecked	Unchecked	Unchecked	Unchecked	Unchecked	Unchecked
Unchecked	Unchecked	Unchecked	Unchecked	Unchecked	Unchecked	Unchecked
Unchecked	Unchecked	Checked	Unchecked	Unchecked	Unchecked	Unchecked
Unchecked	Checked	Unchecked	Unchecked	Unchecked	Unchecked	Unchecked
Unchecked	Unchecked	Checked	Unchecked	Unchecked	Unchecked	Unchecked
Unchecked	Unchecked	Unchecked	Unchecked	Unchecked	Unchecked	Unchecked
Unchecked	Unchecked	Unchecked	Unchecked	Unchecked	Unchecked	Unchecked
Unchecked	Unchecked	Unchecked	Unchecked	Unchecked	Unchecked	Unchecked
Unchecked	Unchecked	Unchecked	Unchecked	Unchecked	Unchecked	Unchecked
Unchecked	Unchecked	Unchecked	Unchecked	Checked	Unchecked	Unchecked
Unchecked	Checked	Unchecked	Unchecked	Unchecked	Unchecked	Unchecked
Unchecked	Unchecked	Unchecked	Unchecked	Unchecked	Unchecked	Unchecked
Unchecked	Unchecked	Unchecked	Unchecked	Unchecked	Unchecked	Unchecked
Unchecked	Unchecked	Unchecked	Unchecked	Checked	Unchecked	Unchecked
Unchecked	Unchecked	Unchecked	Unchecked	Unchecked	Unchecked	Unchecked
Unchecked	Unchecked	Unchecked	Unchecked	Unchecked	Unchecked	Unchecked

Unchecked	Unchecked	Unchecked	Unchecked	Unchecked	Checked	Unchecked
Unchecked	Unchecked	Unchecked	Unchecked	Unchecked	Unchecked	Unchecked
Unchecked	Unchecked	Unchecked	Unchecked	Unchecked	Unchecked	Unchecked
Unchecked	Unchecked	Unchecked	Unchecked	Unchecked	Unchecked	Unchecked
Unchecked	Unchecked	Unchecked	Unchecked	Unchecked	Unchecked	Unchecked
Unchecked	Unchecked	Unchecked	Unchecked	Unchecked	Unchecked	Unchecked
Unchecked	Unchecked	Unchecked	Unchecked	Unchecked	Unchecked	Unchecked
Unchecked	Checked	Unchecked	Checked	Unchecked	Unchecked	Unchecked
Unchecked	Unchecked	Unchecked	Unchecked	Unchecked	Unchecked	Unchecked
Unchecked	Unchecked	Unchecked	Unchecked	Unchecked	Unchecked	Unchecked
Unchecked	Checked	Unchecked	Unchecked	Unchecked	Unchecked	Unchecked
Unchecked	Unchecked	Unchecked	Unchecked	Unchecked	Unchecked	Unchecked
Unchecked	Unchecked	Unchecked	Unchecked	Checked	Unchecked	Unchecked
Unchecked	Unchecked	Unchecked	Unchecked	Unchecked	Unchecked	Unchecked
Unchecked	Unchecked	Unchecked	Unchecked	Unchecked	Unchecked	Unchecked
Unchecked	Unchecked	Unchecked	Unchecked	Unchecked	Unchecked	Unchecked
Unchecked	Unchecked	Unchecked	Unchecked	Unchecked	Unchecked	Unchecked
Unchecked	Unchecked	Unchecked	Unchecked	Unchecked	Unchecked	Unchecked
Unchecked	Unchecked	Unchecked	Unchecked	Unchecked	Unchecked	Unchecked
Unchecked	Unchecked	Unchecked	Unchecked	Unchecked	Unchecked	Unchecked
Unchecked	Unchecked	Unchecked	Unchecked	Unchecked	Unchecked	Unchecked
Unchecked	Unchecked	Unchecked	Unchecked	Unchecked	Unchecked	Unchecked
Unchecked	Unchecked	Unchecked	Checked	Unchecked	Unchecked	Unchecked
Unchecked	Unchecked	Unchecked	Unchecked	Unchecked	Unchecked	Unchecked
Unchecked	Unchecked	Unchecked	Unchecked	Unchecked	Unchecked	Unchecked
Unchecked	Unchecked	Unchecked	Unchecked	Checked	Unchecked	Unchecked
Unchecked	Unchecked	Unchecked	Unchecked	Unchecked	Unchecked	Unchecked
Unchecked	Unchecked	Unchecked	Unchecked	Unchecked	Unchecked	Unchecked
Unchecked	Unchecked	Unchecked	Unchecked	Unchecked	Unchecked	Unchecked
Unchecked	Unchecked	Unchecked	Unchecked	Unchecked	Unchecked	Unchecked
Unchecked	Unchecked	Unchecked	Unchecked	Checked	Unchecked	Unchecked
Unchecked	Unchecked	Unchecked	Unchecked	Unchecked	Unchecked	Unchecked
Unchecked	Unchecked	Unchecked	Unchecked	Checked	Unchecked	Unchecked
Unchecked	Unchecked	Unchecked	Unchecked	Unchecked	Unchecked	Unchecked
Unchecked	Unchecked	Unchecked	Unchecked	Unchecked	Unchecked	Unchecked
Unchecked	Unchecked	Unchecked	Unchecked	Unchecked	Unchecked	Unchecked
Unchecked	Unchecked	Unchecked	Unchecked	Unchecked	Unchecked	Unchecked
Unchecked	Unchecked	Unchecked	Unchecked	Unchecked	Unchecked	Unchecked
Unchecked	Unchecked	Unchecked	Unchecked	Unchecked	Unchecked	Unchecked
Unchecked	Unchecked	Unchecked	Unchecked	Unchecked	Unchecked	Unchecked
Unchecked	Unchecked	Unchecked	Unchecked	Unchecked	Unchecked	Unchecked
Unchecked	Unchecked	Unchecked	Unchecked	Unchecked	Unchecked	Unchecked
Unchecked	Unchecked	Checked	Unchecked	Unchecked	Unchecked	Unchecked

Unchecked	Unchecked	Unchecked	Unchecked	Unchecked	Unchecked	Unchecked
Unchecked	Unchecked	Checked	Unchecked	Checked	Unchecked	Unchecked
Unchecked	Unchecked	Unchecked	Unchecked	Checked	Unchecked	Unchecked
Unchecked	Unchecked	Unchecked	Unchecked	Unchecked	Unchecked	Unchecked
Checked	Unchecked	Unchecked	Unchecked	Checked	Unchecked	Unchecked
Unchecked	Unchecked	Unchecked	Checked	Checked	Unchecked	Unchecked
Unchecked	Unchecked	Unchecked	Unchecked	Unchecked	Unchecked	Unchecked
Unchecked	Unchecked	Checked	Unchecked	Unchecked	Unchecked	Unchecked
Unchecked	Unchecked	Unchecked	Checked	Unchecked	Unchecked	Unchecked
Unchecked	Unchecked	Unchecked	Unchecked	Unchecked	Unchecked	Unchecked
Unchecked	Unchecked	Checked	Unchecked	Unchecked	Unchecked	Unchecked
Unchecked	Unchecked	Unchecked	Unchecked	Unchecked	Unchecked	Unchecked
Unchecked	Unchecked	Checked	Unchecked	Unchecked	Unchecked	Unchecked
Unchecked	Unchecked	Unchecked	Unchecked	Unchecked	Unchecked	Unchecked
Unchecked	Unchecked	Unchecked	Unchecked	Unchecked	Unchecked	Unchecked
Unchecked	Unchecked	Unchecked	Unchecked	Unchecked	Unchecked	Unchecked
Unchecked	Unchecked	Unchecked	Unchecked	Unchecked	Unchecked	Unchecked
Unchecked	Unchecked	Checked	Unchecked	Unchecked	Unchecked	Unchecked
Unchecked	Unchecked	Checked	Unchecked	Unchecked	Unchecked	Unchecked
Unchecked	Unchecked	Checked	Unchecked	Unchecked	Unchecked	Unchecked
Unchecked	Unchecked	Unchecked	Unchecked	Unchecked	Unchecked	Unchecked
Unchecked	Unchecked	Unchecked	Unchecked	Unchecked	Unchecked	Unchecked
Unchecked	Unchecked	Unchecked	Unchecked	Unchecked	Unchecked	Unchecked
Unchecked	Unchecked	Unchecked	Unchecked	Unchecked	Unchecked	Unchecked
Unchecked	Unchecked	Unchecked	Unchecked	Unchecked	Unchecked	Unchecked
Unchecked	Unchecked	Unchecked	Unchecked	Unchecked	Unchecked	Unchecked
Unchecked	Unchecked	Unchecked	Unchecked	Unchecked	Unchecked	Unchecked
Unchecked	Unchecked	Unchecked	Unchecked	Unchecked	Unchecked	Unchecked
Unchecked	Unchecked	Unchecked	Unchecked	Checked	Unchecked	Unchecked
Unchecked	Unchecked	Unchecked	Checked	Unchecked	Unchecked	Unchecked
Unchecked	Unchecked	Unchecked	Unchecked	Unchecked	Unchecked	Unchecked
Unchecked	Unchecked	Unchecked	Checked	Unchecked	Unchecked	Unchecked
Unchecked	Checked	Checked	Unchecked	Unchecked	Unchecked	Unchecked
Unchecked	Unchecked	Unchecked	Unchecked	Unchecked	Unchecked	Checked
Unchecked	Unchecked	Unchecked	Unchecked	Unchecked	Unchecked	Unchecked
Checked	Unchecked	Unchecked	Unchecked	Checked	Unchecked	Unchecked
Unchecked	Unchecked	Unchecked	Unchecked	Unchecked	Unchecked	Unchecked
Unchecked	Unchecked	Unchecked	Unchecked	Unchecked	Unchecked	Unchecked
Unchecked	Unchecked	Unchecked	Unchecked	Unchecked	Unchecked	Unchecked
Unchecked	Unchecked	Unchecked	Unchecked	Unchecked	Unchecked	Unchecked
Unchecked	Unchecked	Unchecked	Checked	Checked	Unchecked	Unchecked
Unchecked	Checked	Unchecked	Unchecked	Unchecked	Unchecked	Unchecked
Unchecked	Unchecked	Unchecked	Unchecked	Unchecked	Unchecked	Unchecked

Unchecked	Unchecked	Unchecked	Checked	Checked	Unchecked	Unchecked
Unchecked	Unchecked	Unchecked	Unchecked	Unchecked	Unchecked	Unchecked
Unchecked	Unchecked	Unchecked	Unchecked	Unchecked	Unchecked	Unchecked
Unchecked	Unchecked	Unchecked	Unchecked	Unchecked	Unchecked	Unchecked
Unchecked	Unchecked	Unchecked	Unchecked	Unchecked	Unchecked	Unchecked
Unchecked	Checked	Unchecked	Checked	Checked	Unchecked	Unchecked
Unchecked	Unchecked	Unchecked	Unchecked	Unchecked	Unchecked	Unchecked
Unchecked	Unchecked	Unchecked	Unchecked	Unchecked	Unchecked	Unchecked
Checked	Unchecked	Checked	Unchecked	Unchecked	Unchecked	Unchecked
Unchecked	Unchecked	Unchecked	Unchecked	Unchecked	Unchecked	Unchecked
Unchecked	Unchecked	Unchecked	Unchecked	Unchecked	Unchecked	Unchecked
Unchecked	Unchecked	Unchecked	Unchecked	Unchecked	Unchecked	Unchecked
Unchecked	Unchecked	Unchecked	Unchecked	Unchecked	Unchecked	Unchecked
Unchecked	Unchecked	Unchecked	Unchecked	Unchecked	Unchecked	Unchecked
Unchecked	Unchecked	Unchecked	Unchecked	Unchecked	Unchecked	Unchecked
Unchecked	Unchecked	Unchecked	Unchecked	Unchecked	Unchecked	Unchecked
Unchecked	Unchecked	Unchecked	Unchecked	Unchecked	Unchecked	Unchecked
Unchecked	Unchecked	Unchecked	Unchecked	Unchecked	Unchecked	Unchecked
Unchecked	Unchecked	Unchecked	Unchecked	Unchecked	Unchecked	Unchecked
Unchecked	Unchecked	Unchecked	Unchecked	Unchecked	Unchecked	Unchecked
Unchecked	Checked	Unchecked	Unchecked	Unchecked	Unchecked	Unchecked
Unchecked	Unchecked	Unchecked	Unchecked	Unchecked	Unchecked	Unchecked
Unchecked	Unchecked	Unchecked	Unchecked	Unchecked	Unchecked	Unchecked
Unchecked	Unchecked	Unchecked	Unchecked	Checked	Unchecked	Unchecked
Unchecked	Checked	Unchecked	Unchecked	Unchecked	Unchecked	Unchecked
Unchecked	Unchecked	Unchecked	Unchecked	Unchecked	Unchecked	Unchecked
Checked	Checked	Checked	Unchecked	Unchecked	Unchecked	Unchecked
Unchecked	Unchecked	Unchecked	Unchecked	Unchecked	Unchecked	Unchecked
Unchecked	Unchecked	Unchecked	Unchecked	Unchecked	Unchecked	Unchecked
Unchecked	Unchecked	Unchecked	Unchecked	Unchecked	Unchecked	Unchecked
Unchecked	Unchecked	Unchecked	Unchecked	Unchecked	Unchecked	Unchecked
Unchecked	Unchecked	Unchecked	Unchecked	Unchecked	Unchecked	Unchecked
Unchecked	Unchecked	Unchecked	Unchecked	Unchecked	Unchecked	Unchecked
Unchecked	Unchecked	Unchecked	Unchecked	Unchecked	Unchecked	Unchecked
Unchecked	Unchecked	Unchecked	Unchecked	Unchecked	Unchecked	Unchecked
Unchecked	Unchecked	Checked	Unchecked	Unchecked	Unchecked	Unchecked
Unchecked	Unchecked	Unchecked	Unchecked	Unchecked	Unchecked	Unchecked
Unchecked	Unchecked	Unchecked	Unchecked	Checked	Unchecked	Unchecked
Unchecked	Unchecked	Unchecked	Unchecked	Unchecked	Unchecked	Unchecked
Unchecked	Unchecked	Unchecked	Unchecked	Unchecked	Unchecked	Unchecked
Unchecked	Checked	Unchecked	Unchecked	Unchecked	Unchecked	Unchecked
Unchecked	Unchecked	Unchecked	Unchecked	Unchecked	Unchecked	Unchecked
Unchecked	Unchecked	Unchecked	Unchecked	Unchecked	Unchecked	Unchecked

Unchecked	Unchecked	Unchecked	Unchecked	Unchecked	Unchecked	Unchecked
Unchecked	Unchecked	Checked	Unchecked	Unchecked	Unchecked	Unchecked
Unchecked	Unchecked	Unchecked	Unchecked	Checked	Unchecked	Unchecked
Unchecked	Unchecked	Unchecked	Unchecked	Unchecked	Unchecked	Unchecked
Unchecked	Unchecked	Unchecked	Unchecked	Unchecked	Unchecked	Unchecked
Unchecked	Unchecked	Unchecked	Unchecked	Checked	Unchecked	Unchecked
Unchecked	Unchecked	Unchecked	Unchecked	Unchecked	Unchecked	Unchecked
Unchecked	Unchecked	Unchecked	Unchecked	Unchecked	Unchecked	Unchecked
Unchecked	Unchecked	Unchecked	Unchecked	Unchecked	Unchecked	Unchecked
Unchecked	Checked	Unchecked	Unchecked	Unchecked	Unchecked	Unchecked
Unchecked	Unchecked	Unchecked	Unchecked	Unchecked	Unchecked	Unchecked
Unchecked	Unchecked	Unchecked	Unchecked	Unchecked	Unchecked	Unchecked
Unchecked	Unchecked	Unchecked	Checked	Unchecked	Unchecked	Unchecked
Unchecked	Unchecked	Unchecked	Unchecked	Unchecked	Unchecked	Unchecked
Unchecked	Checked	Unchecked	Checked	Checked	Unchecked	Unchecked
Unchecked	Unchecked	Unchecked	Unchecked	Unchecked	Unchecked	Unchecked
Unchecked	Unchecked	Unchecked	Unchecked	Unchecked	Unchecked	Unchecked
Unchecked	Unchecked	Unchecked	Unchecked	Unchecked	Unchecked	Unchecked
Unchecked	Unchecked	Unchecked	Unchecked	Unchecked	Unchecked	Unchecked
Unchecked	Unchecked	Checked	Unchecked	Unchecked	Unchecked	Unchecked
Unchecked	Unchecked	Unchecked	Unchecked	Unchecked	Unchecked	Unchecked
Unchecked	Unchecked	Unchecked	Unchecked	Unchecked	Unchecked	Unchecked
Unchecked	Unchecked	Unchecked	Checked	Unchecked	Unchecked	Unchecked
Unchecked	Checked	Checked	Unchecked	Checked	Unchecked	Unchecked
Unchecked	Unchecked	Unchecked	Unchecked	Unchecked	Unchecked	Unchecked
Unchecked	Unchecked	Unchecked	Unchecked	Unchecked	Unchecked	Unchecked
Unchecked	Checked	Unchecked	Unchecked	Unchecked	Unchecked	Unchecked
Unchecked	Unchecked	Unchecked	Unchecked	Unchecked	Unchecked	Unchecked
Unchecked	Unchecked	Unchecked	Unchecked	Unchecked	Unchecked	Unchecked
Unchecked	Unchecked	Unchecked	Unchecked	Unchecked	Unchecked	Unchecked
Unchecked	Unchecked	Unchecked	Unchecked	Unchecked	Unchecked	Unchecked
Unchecked	Unchecked	Unchecked	Unchecked	Unchecked	Unchecked	Unchecked
Unchecked	Unchecked	Unchecked	Unchecked	Unchecked	Unchecked	Unchecked
Unchecked	Unchecked	Unchecked	Unchecked	Unchecked	Unchecked	Unchecked
Unchecked	Unchecked	Unchecked	Unchecked	Unchecked	Unchecked	Unchecked
Unchecked	Unchecked	Unchecked	Unchecked	Unchecked	Unchecked	Unchecked
Unchecked	Unchecked	Unchecked	Unchecked	Unchecked	Unchecked	Unchecked
Unchecked	Unchecked	Unchecked	Unchecked	Unchecked	Unchecked	Unchecked
Unchecked	Unchecked	Unchecked	Unchecked	Unchecked	Unchecked	Unchecked
Unchecked	Unchecked	Unchecked	Unchecked	Unchecked	Unchecked	Unchecked
Unchecked	Unchecked	Unchecked	Unchecked	Unchecked	Unchecked	Unchecked
Unchecked	Unchecked	Unchecked	Unchecked	Unchecked	Unchecked	Unchecked
Unchecked	Unchecked	Unchecked	Unchecked	Unchecked	Unchecked	Unchecked

Unchecked	Unchecked	Unchecked	Unchecked	Unchecked	Unchecked	Unchecked
Unchecked	Unchecked	Unchecked	Unchecked	Unchecked	Unchecked	Unchecked
Unchecked	Unchecked	Unchecked	Unchecked	Unchecked	Unchecked	Unchecked
Unchecked	Unchecked	Unchecked	Unchecked	Unchecked	Unchecked	Unchecked
Unchecked	Checked	Checked	Unchecked	Unchecked	Unchecked	Unchecked
Unchecked	Checked	Unchecked	Unchecked	Unchecked	Unchecked	Unchecked
Unchecked	Unchecked	Unchecked	Unchecked	Unchecked	Unchecked	Unchecked
Checked	Checked	Unchecked	Unchecked	Unchecked	Unchecked	Unchecked
Unchecked	Unchecked	Unchecked	Unchecked	Unchecked	Unchecked	Unchecked
Unchecked	Unchecked	Unchecked	Unchecked	Unchecked	Unchecked	Unchecked
Unchecked	Unchecked	Unchecked	Unchecked	Unchecked	Unchecked	Unchecked
Unchecked	Unchecked	Checked	Unchecked	Checked	Unchecked	Unchecked
Unchecked	Unchecked	Unchecked	Unchecked	Unchecked	Unchecked	Unchecked
Unchecked	Checked	Unchecked	Checked	Checked	Unchecked	Unchecked
Unchecked	Checked	Checked	Checked	Unchecked	Unchecked	Unchecked
Unchecked	Unchecked	Unchecked	Checked	Unchecked	Unchecked	Unchecked
Unchecked	Unchecked	Unchecked	Unchecked	Unchecked	Unchecked	Unchecked
Unchecked	Unchecked	Unchecked	Unchecked	Unchecked	Unchecked	Unchecked
Unchecked	Unchecked	Unchecked	Unchecked	Unchecked	Unchecked	Unchecked
Unchecked	Unchecked	Unchecked	Unchecked	Checked	Unchecked	Unchecked
Unchecked	Unchecked	Unchecked	Unchecked	Unchecked	Checked	Unchecked
Unchecked	Unchecked	Unchecked	Unchecked	Unchecked	Unchecked	Unchecked
Unchecked	Unchecked	Unchecked	Unchecked	Unchecked	Unchecked	Unchecked
Unchecked	Unchecked	Unchecked	Unchecked	Unchecked	Unchecked	Unchecked
Unchecked	Unchecked	Unchecked	Unchecked	Unchecked	Unchecked	Unchecked
Unchecked	Unchecked	Unchecked	Unchecked	Unchecked	Unchecked	Unchecked
Unchecked	Unchecked	Unchecked	Unchecked	Unchecked	Unchecked	Unchecked
Unchecked	Unchecked	Unchecked	Unchecked	Unchecked	Unchecked	Unchecked
Unchecked	Unchecked	Unchecked	Unchecked	Unchecked	Unchecked	Unchecked
Unchecked	Unchecked	Unchecked	Unchecked	Unchecked	Unchecked	Unchecked
Unchecked	Unchecked	Checked	Unchecked	Unchecked	Unchecked	Unchecked
Unchecked	Unchecked	Unchecked	Unchecked	Unchecked	Unchecked	Unchecked
Unchecked	Unchecked	Unchecked	Unchecked	Unchecked	Unchecked	Unchecked
Unchecked	Unchecked	Unchecked	Unchecked	Unchecked	Unchecked	Unchecked
Unchecked	Unchecked	Unchecked	Unchecked	Unchecked	Unchecked	Unchecked
Unchecked	Unchecked	Unchecked	Unchecked	Unchecked	Unchecked	Unchecked
Unchecked	Unchecked	Unchecked	Unchecked	Unchecked	Unchecked	Unchecked
Unchecked	Unchecked	Unchecked	Unchecked	Checked	Unchecked	Unchecked
Unchecked	Unchecked	Unchecked	Unchecked	Unchecked	Unchecked	Unchecked
Unchecked	Unchecked	Unchecked	Unchecked	Unchecked	Unchecked	Unchecked
Unchecked	Unchecked	Unchecked	Unchecked	Unchecked	Unchecked	Unchecked
Unchecked	Unchecked	Unchecked	Unchecked	Unchecked	Unchecked	Unchecked
Unchecked	Unchecked	Unchecked	Unchecked	Unchecked	Unchecked	Unchecked

Unchecked	Unchecked	Unchecked	Unchecked	Checked	Checked	Unchecked
Unchecked	Unchecked	Unchecked	Unchecked	Checked	Checked	Unchecked
Unchecked	Unchecked	Unchecked	Unchecked	Unchecked	Unchecked	Unchecked
Unchecked	Unchecked	Unchecked	Unchecked	Unchecked	Unchecked	Unchecked
Unchecked	Unchecked	Unchecked	Unchecked	Unchecked	Unchecked	Unchecked
Unchecked	Unchecked	Unchecked	Unchecked	Unchecked	Unchecked	Unchecked
Unchecked	Unchecked	Unchecked	Unchecked	Unchecked	Unchecked	Unchecked
Unchecked	Unchecked	Unchecked	Unchecked	Unchecked	Unchecked	Unchecked
Unchecked	Unchecked	Unchecked	Unchecked	Unchecked	Unchecked	Unchecked
Unchecked	Unchecked	Checked	Unchecked	Unchecked	Unchecked	Unchecked
Unchecked	Unchecked	Unchecked	Unchecked	Unchecked	Unchecked	Unchecked
Unchecked	Unchecked	Unchecked	Unchecked	Unchecked	Unchecked	Unchecked
Unchecked	Unchecked	Checked	Unchecked	Unchecked	Unchecked	Unchecked
Unchecked	Checked	Checked	Unchecked	Checked	Unchecked	Unchecked
Unchecked	Unchecked	Unchecked	Unchecked	Unchecked	Unchecked	Unchecked
Unchecked	Unchecked	Unchecked	Checked	Unchecked	Unchecked	Unchecked
Unchecked	Unchecked	Unchecked	Unchecked	Unchecked	Unchecked	Unchecked
Unchecked	Unchecked	Unchecked	Unchecked	Unchecked	Unchecked	Unchecked
Unchecked	Unchecked	Unchecked	Unchecked	Unchecked	Unchecked	Unchecked
Unchecked	Unchecked	Checked	Checked	Unchecked	Unchecked	Unchecked
Unchecked	Unchecked	Unchecked	Checked	Checked	Unchecked	Unchecked
Unchecked	Unchecked	Unchecked	Unchecked	Unchecked	Unchecked	Unchecked
Unchecked	Unchecked	Unchecked	Unchecked	Unchecked	Unchecked	Unchecked
Checked	Checked	Checked	Unchecked	Unchecked	Unchecked	Unchecked
Unchecked	Checked	Unchecked	Unchecked	Unchecked	Unchecked	Unchecked
Unchecked	Unchecked	Unchecked	Unchecked	Unchecked	Unchecked	Unchecked
Unchecked	Unchecked	Unchecked	Unchecked	Unchecked	Unchecked	Unchecked
Unchecked	Checked	Unchecked	Unchecked	Checked	Unchecked	Unchecked
Unchecked	Unchecked	Unchecked	Unchecked	Unchecked	Unchecked	Unchecked
Unchecked	Unchecked	Unchecked	Unchecked	Unchecked	Unchecked	Unchecked
Unchecked	Unchecked	Unchecked	Unchecked	Unchecked	Unchecked	Unchecked
Unchecked	Unchecked	Unchecked	Unchecked	Unchecked	Unchecked	Unchecked
Unchecked	Unchecked	Unchecked	Unchecked	Unchecked	Unchecked	Unchecked
Unchecked	Unchecked	Unchecked	Unchecked	Unchecked	Unchecked	Unchecked
Unchecked	Unchecked	Unchecked	Unchecked	Unchecked	Unchecked	Unchecked
Unchecked	Unchecked	Unchecked	Unchecked	Unchecked	Unchecked	Unchecked
Unchecked	Unchecked	Unchecked	Unchecked	Unchecked	Unchecked	Unchecked
Unchecked	Unchecked	Unchecked	Unchecked	Unchecked	Unchecked	Unchecked
Unchecked	Unchecked	Unchecked	Unchecked	Unchecked	Unchecked	Unchecked
Unchecked	Unchecked	Unchecked	Unchecked	Unchecked	Unchecked	Unchecked
Unchecked	Unchecked	Unchecked	Unchecked	Unchecked	Unchecked	Unchecked
Unchecked	Unchecked	Unchecked	Unchecked	Unchecked	Unchecked	Unchecked
Unchecked	Unchecked	Unchecked	Unchecked	Unchecked	Unchecked	Unchecked

Unchecked	Unchecked	Unchecked	Unchecked	Unchecked	Unchecked	Unchecked
Unchecked	Unchecked	Unchecked	Unchecked	Unchecked	Unchecked	Unchecked
Unchecked	Unchecked	Unchecked	Unchecked	Unchecked	Unchecked	Unchecked
Unchecked	Unchecked	Unchecked	Unchecked	Unchecked	Unchecked	Unchecked
Unchecked	Unchecked	Unchecked	Unchecked	Unchecked	Unchecked	Unchecked
Unchecked	Unchecked	Checked	Unchecked	Unchecked	Unchecked	Unchecked
Unchecked	Unchecked	Checked	Unchecked	Unchecked	Unchecked	Unchecked
Unchecked	Unchecked	Unchecked	Unchecked	Unchecked	Unchecked	Unchecked
Unchecked	Unchecked	Unchecked	Unchecked	Unchecked	Unchecked	Unchecked
Unchecked	Checked	Unchecked	Unchecked	Unchecked	Unchecked	Unchecked
Unchecked	Unchecked	Unchecked	Unchecked	Unchecked	Unchecked	Unchecked
Unchecked	Unchecked	Unchecked	Unchecked	Unchecked	Unchecked	Unchecked
Unchecked	Unchecked	Unchecked	Unchecked	Unchecked	Unchecked	Unchecked
Unchecked	Checked	Unchecked	Unchecked	Unchecked	Unchecked	Unchecked
Unchecked	Unchecked	Unchecked	Unchecked	Unchecked	Unchecked	Unchecked
Unchecked	Unchecked	Unchecked	Unchecked	Unchecked	Unchecked	Unchecked
Unchecked	Checked	Unchecked	Unchecked	Unchecked	Unchecked	Unchecked
Unchecked	Unchecked	Unchecked	Unchecked	Unchecked	Unchecked	Unchecked
Unchecked	Checked	Unchecked	Unchecked	Unchecked	Unchecked	Unchecked
Unchecked	Unchecked	Unchecked	Unchecked	Unchecked	Unchecked	Unchecked
Unchecked	Unchecked	Unchecked	Unchecked	Unchecked	Unchecked	Unchecked
Unchecked	Unchecked	Unchecked	Unchecked	Unchecked	Unchecked	Unchecked
Unchecked	Unchecked	Unchecked	Unchecked	Unchecked	Unchecked	Unchecked
Unchecked	Unchecked	Unchecked	Unchecked	Unchecked	Unchecked	Unchecked
Unchecked	Unchecked	Unchecked	Checked	Unchecked	Unchecked	Unchecked
Unchecked	Unchecked	Checked	Unchecked	Unchecked	Unchecked	Unchecked
Unchecked	Unchecked	Unchecked	Unchecked	Unchecked	Unchecked	Unchecked
Checked	Checked	Checked	Unchecked	Unchecked	Unchecked	Unchecked
Unchecked	Unchecked	Unchecked	Unchecked	Unchecked	Unchecked	Unchecked
Unchecked	Unchecked	Unchecked	Unchecked	Unchecked	Unchecked	Unchecked
Unchecked	Unchecked	Unchecked	Unchecked	Unchecked	Unchecked	Unchecked
Unchecked	Unchecked	Checked	Unchecked	Unchecked	Unchecked	Unchecked
Unchecked	Unchecked	Unchecked	Unchecked	Unchecked	Unchecked	Unchecked
Unchecked	Unchecked	Unchecked	Unchecked	Unchecked	Unchecked	Unchecked
Unchecked	Unchecked	Unchecked	Unchecked	Unchecked	Unchecked	Unchecked
Unchecked	Unchecked	Unchecked	Unchecked	Unchecked	Unchecked	Unchecked
Unchecked	Unchecked	Unchecked	Unchecked	Unchecked	Unchecked	Unchecked
Unchecked	Unchecked	Unchecked	Unchecked	Unchecked	Unchecked	Unchecked
Unchecked	Unchecked	Unchecked	Unchecked	Unchecked	Unchecked	Unchecked
Unchecked	Unchecked	Unchecked	Unchecked	Unchecked	Unchecked	Unchecked
Unchecked	Unchecked	Unchecked	Unchecked	Unchecked	Unchecked	Unchecked
Unchecked	Unchecked	Unchecked	Unchecked	Unchecked	Unchecked	Unchecked
Unchecked	Unchecked	Unchecked	Unchecked	Unchecked	Unchecked	Unchecked

Unchecked	Unchecked	Unchecked	Unchecked	Unchecked	Unchecked	Unchecked
Unchecked	Unchecked	Unchecked	Unchecked	Unchecked	Unchecked	Unchecked
Unchecked	Unchecked	Unchecked	Unchecked	Unchecked	Unchecked	Unchecked
Unchecked	Unchecked	Unchecked	Unchecked	Unchecked	Unchecked	Unchecked
Unchecked	Unchecked	Unchecked	Unchecked	Unchecked	Unchecked	Unchecked
Unchecked	Unchecked	Unchecked	Unchecked	Unchecked	Unchecked	Unchecked
Unchecked	Unchecked	Unchecked	Unchecked	Unchecked	Unchecked	Unchecked
Unchecked	Unchecked	Unchecked	Unchecked	Unchecked	Unchecked	Unchecked
Unchecked	Unchecked	Unchecked	Unchecked	Unchecked	Unchecked	Unchecked
Unchecked	Unchecked	Unchecked	Unchecked	Unchecked	Unchecked	Unchecked
Unchecked	Unchecked	Unchecked	Unchecked	Unchecked	Unchecked	Unchecked
Unchecked	Unchecked	Unchecked	Unchecked	Unchecked	Unchecked	Unchecked
Unchecked	Unchecked	Unchecked	Unchecked	Unchecked	Unchecked	Unchecked
Unchecked	Checked	Unchecked	Unchecked	Unchecked	Unchecked	Unchecked
Unchecked	Unchecked	Unchecked	Unchecked	Unchecked	Unchecked	Unchecked
Unchecked	Unchecked	Unchecked	Unchecked	Unchecked	Unchecked	Unchecked
Unchecked	Unchecked	Unchecked	Unchecked	Checked	Unchecked	Unchecked
Unchecked	Unchecked	Unchecked	Checked	Unchecked	Unchecked	Unchecked
Unchecked	Unchecked	Unchecked	Unchecked	Unchecked	Unchecked	Unchecked
Unchecked	Unchecked	Unchecked	Unchecked	Unchecked	Unchecked	Unchecked
Unchecked	Unchecked	Unchecked	Unchecked	Unchecked	Unchecked	Unchecked
Unchecked	Unchecked	Unchecked	Unchecked	Unchecked	Unchecked	Unchecked
Unchecked	Unchecked	Unchecked	Unchecked	Unchecked	Unchecked	Unchecked
Unchecked	Unchecked	Unchecked	Unchecked	Unchecked	Unchecked	Unchecked
Unchecked	Unchecked	Unchecked	Unchecked	Unchecked	Unchecked	Unchecked
Unchecked	Unchecked	Unchecked	Unchecked	Unchecked	Unchecked	Unchecked
Unchecked	Unchecked	Unchecked	Unchecked	Unchecked	Unchecked	Unchecked
Unchecked	Unchecked	Unchecked	Unchecked	Unchecked	Unchecked	Unchecked
Unchecked	Unchecked	Unchecked	Unchecked	Unchecked	Unchecked	Unchecked
Unchecked	Checked	Unchecked	Checked	Unchecked	Unchecked	Unchecked
Unchecked	Unchecked	Unchecked	Checked	Checked	Unchecked	Unchecked
Unchecked	Unchecked	Unchecked	Unchecked	Unchecked	Unchecked	Unchecked
Unchecked	Unchecked	Unchecked	Unchecked	Unchecked	Unchecked	Unchecked
Unchecked	Unchecked	Unchecked	Unchecked	Unchecked	Unchecked	Unchecked
Unchecked	Unchecked	Unchecked	Unchecked	Unchecked	Unchecked	Unchecked
Unchecked	Unchecked	Unchecked	Unchecked	Unchecked	Unchecked	Unchecked
Unchecked	Unchecked	Unchecked	Unchecked	Unchecked	Unchecked	Unchecked
Unchecked	Unchecked	Unchecked	Checked	Unchecked	Unchecked	Unchecked
Unchecked	Unchecked	Unchecked	Unchecked	Unchecked	Unchecked	Unchecked
Unchecked	Unchecked	Unchecked	Unchecked	Unchecked	Unchecked	Unchecked
Unchecked	Unchecked	Unchecked	Unchecked	Unchecked	Unchecked	Unchecked
Unchecked	Unchecked	Unchecked	Unchecked	Unchecked	Unchecked	Unchecked
Unchecked	Unchecked	Unchecked	Unchecked	Unchecked	Unchecked	Unchecked

Unchecked	Unchecked	Unchecked	Unchecked	Unchecked	Unchecked	Unchecked
Unchecked	Unchecked	Unchecked	Unchecked	Unchecked	Unchecked	Unchecked
Unchecked	Unchecked	Unchecked	Unchecked	Unchecked	Unchecked	Unchecked
Unchecked	Unchecked	Unchecked	Unchecked	Unchecked	Unchecked	Unchecked
Unchecked	Unchecked	Unchecked	Unchecked	Unchecked	Unchecked	Unchecked
Unchecked	Unchecked	Unchecked	Unchecked	Unchecked	Unchecked	Unchecked
Unchecked	Unchecked	Unchecked	Checked	Checked	Unchecked	Unchecked
Unchecked	Unchecked	Unchecked	Unchecked	Unchecked	Unchecked	Unchecked
Unchecked	Unchecked	Unchecked	Unchecked	Unchecked	Unchecked	Unchecked
Unchecked	Unchecked	Unchecked	Unchecked	Unchecked	Unchecked	Unchecked
Unchecked	Unchecked	Unchecked	Unchecked	Unchecked	Unchecked	Unchecked
Unchecked	Unchecked	Checked	Checked	Checked	Unchecked	Unchecked
Unchecked	Checked	Checked	Unchecked	Unchecked	Unchecked	Unchecked
Unchecked	Unchecked	Unchecked	Unchecked	Unchecked	Unchecked	Unchecked
Unchecked	Unchecked	Unchecked	Unchecked	Unchecked	Unchecked	Unchecked
Unchecked	Unchecked	Unchecked	Unchecked	Unchecked	Unchecked	Unchecked
Unchecked	Unchecked	Unchecked	Unchecked	Unchecked	Unchecked	Unchecked
Unchecked	Unchecked	Unchecked	Unchecked	Unchecked	Unchecked	Unchecked
Unchecked	Unchecked	Checked	Unchecked	Checked	Unchecked	Unchecked
Unchecked	Unchecked	Unchecked	Unchecked	Checked	Unchecked	Unchecked
Unchecked	Unchecked	Checked	Unchecked	Checked	Unchecked	Unchecked
Unchecked	Unchecked	Unchecked	Unchecked	Unchecked	Unchecked	Unchecked
Unchecked	Unchecked	Unchecked	Unchecked	Unchecked	Unchecked	Unchecked
Unchecked	Unchecked	Unchecked	Unchecked	Unchecked	Unchecked	Unchecked
Unchecked	Unchecked	Unchecked	Unchecked	Unchecked	Unchecked	Unchecked
Unchecked	Unchecked	Unchecked	Unchecked	Unchecked	Unchecked	Unchecked
Unchecked	Unchecked	Unchecked	Checked	Unchecked	Unchecked	Unchecked
Unchecked	Unchecked	Unchecked	Unchecked	Unchecked	Unchecked	Unchecked
Unchecked	Unchecked	Unchecked	Unchecked	Unchecked	Unchecked	Unchecked
Unchecked	Unchecked	Unchecked	Unchecked	Unchecked	Unchecked	Unchecked
Unchecked	Unchecked	Unchecked	Unchecked	Unchecked	Unchecked	Unchecked
Unchecked	Unchecked	Unchecked	Unchecked	Unchecked	Unchecked	Unchecked
Unchecked	Unchecked	Unchecked	Checked	Unchecked	Unchecked	Unchecked
Unchecked	Unchecked	Unchecked	Unchecked	Checked	Unchecked	Unchecked
Unchecked	Unchecked	Unchecked	Unchecked	Unchecked	Unchecked	Unchecked
Unchecked	Unchecked	Unchecked	Unchecked	Unchecked	Unchecked	Unchecked
Unchecked	Unchecked	Unchecked	Unchecked	Unchecked	Unchecked	Unchecked
Unchecked	Unchecked	Unchecked	Unchecked	Unchecked	Unchecked	Unchecked
Unchecked	Unchecked	Unchecked	Unchecked	Unchecked	Unchecked	Unchecked
Unchecked	Unchecked	Unchecked	Unchecked	Unchecked	Checked	Unchecked
Unchecked	Unchecked	Checked	Checked	Checked	Unchecked	Unchecked
Unchecked	Unchecked	Unchecked	Unchecked	Unchecked	Unchecked	Unchecked
Unchecked	Unchecked	Unchecked	Unchecked	Unchecked	Unchecked	Unchecked

Unchecked	Unchecked	Checked	Unchecked	Unchecked	Unchecked	Unchecked
Unchecked	Unchecked	Unchecked	Unchecked	Unchecked	Unchecked	Unchecked
Unchecked	Unchecked	Unchecked	Unchecked	Unchecked	Unchecked	Unchecked
Unchecked	Unchecked	Unchecked	Unchecked	Unchecked	Unchecked	Unchecked
Unchecked	Unchecked	Unchecked	Unchecked	Unchecked	Unchecked	Unchecked
Unchecked	Unchecked	Unchecked	Checked	Unchecked	Unchecked	Unchecked
Unchecked	Unchecked	Unchecked	Unchecked	Unchecked	Unchecked	Unchecked
Unchecked	Unchecked	Unchecked	Unchecked	Unchecked	Unchecked	Unchecked
Unchecked	Unchecked	Unchecked	Unchecked	Unchecked	Unchecked	Unchecked
Unchecked	Unchecked	Unchecked	Unchecked	Unchecked	Unchecked	Unchecked
Unchecked	Unchecked	Unchecked	Unchecked	Unchecked	Unchecked	Unchecked
Unchecked	Unchecked	Unchecked	Unchecked	Unchecked	Unchecked	Unchecked
Unchecked	Unchecked	Unchecked	Unchecked	Unchecked	Unchecked	Unchecked
Unchecked	Unchecked	Unchecked	Unchecked	Unchecked	Unchecked	Unchecked
Unchecked	Unchecked	Unchecked	Unchecked	Unchecked	Unchecked	Unchecked
Unchecked	Unchecked	Unchecked	Unchecked	Unchecked	Unchecked	Unchecked
Unchecked	Unchecked	Unchecked	Unchecked	Unchecked	Unchecked	Unchecked
Unchecked	Unchecked	Unchecked	Unchecked	Unchecked	Unchecked	Unchecked
Unchecked	Unchecked	Unchecked	Unchecked	Unchecked	Unchecked	Unchecked
Unchecked	Unchecked	Unchecked	Unchecked	Unchecked	Unchecked	Unchecked
Unchecked	Unchecked	Unchecked	Unchecked	Unchecked	Unchecked	Unchecked
Unchecked	Unchecked	Unchecked	Unchecked	Unchecked	Unchecked	Unchecked
Unchecked	Unchecked	Unchecked	Unchecked	Unchecked	Unchecked	Unchecked
Unchecked	Unchecked	Unchecked	Unchecked	Unchecked	Unchecked	Unchecked
Unchecked	Unchecked	Unchecked	Unchecked	Unchecked	Unchecked	Unchecked
Unchecked	Unchecked	Unchecked	Unchecked	Unchecked	Unchecked	Unchecked
Unchecked	Unchecked	Unchecked	Unchecked	Unchecked	Unchecked	Unchecked
Unchecked	Unchecked	Unchecked	Unchecked	Checked	Checked	Unchecked
Unchecked	Unchecked	Unchecked	Unchecked	Unchecked	Unchecked	Unchecked
Unchecked	Unchecked	Unchecked	Unchecked	Unchecked	Unchecked	Unchecked
Unchecked	Unchecked	Checked	Unchecked	Checked	Unchecked	Unchecked
Unchecked	Unchecked	Unchecked	Unchecked	Unchecked	Unchecked	Unchecked
Unchecked	Unchecked	Unchecked	Unchecked	Unchecked	Unchecked	Unchecked
Unchecked	Unchecked	Unchecked	Unchecked	Unchecked	Unchecked	Unchecked
Unchecked	Unchecked	Unchecked	Unchecked	Unchecked	Unchecked	Unchecked
Unchecked	Unchecked	Unchecked	Unchecked	Unchecked	Unchecked	Unchecked
Unchecked	Unchecked	Unchecked	Unchecked	Unchecked	Unchecked	Unchecked
Unchecked	Unchecked	Unchecked	Unchecked	Unchecked	Unchecked	Unchecked
Unchecked	Unchecked	Unchecked	Checked	Unchecked	Unchecked	Unchecked
Unchecked	Unchecked	Checked	Unchecked	Unchecked	Unchecked	Unchecked
Unchecked	Unchecked	Unchecked	Unchecked	Unchecked	Unchecked	Unchecked
Unchecked	Unchecked	Unchecked	Unchecked	Unchecked	Unchecked	Unchecked
Unchecked	Unchecked	Unchecked	Unchecked	Unchecked	Unchecked	Unchecked

Unchecked	Unchecked	Checked	Unchecked	Unchecked	Unchecked	Unchecked
Unchecked	Unchecked	Unchecked	Unchecked	Unchecked	Unchecked	Unchecked
Unchecked	Unchecked	Unchecked	Unchecked	Unchecked	Unchecked	Unchecked
Unchecked	Unchecked	Unchecked	Checked	Unchecked	Unchecked	Unchecked
Unchecked	Unchecked	Unchecked	Unchecked	Unchecked	Unchecked	Unchecked
Unchecked	Unchecked	Unchecked	Unchecked	Unchecked	Unchecked	Unchecked
Unchecked	Unchecked	Unchecked	Checked	Checked	Unchecked	Unchecked
Unchecked	Unchecked	Unchecked	Unchecked	Unchecked	Unchecked	Unchecked
Unchecked	Checked	Unchecked	Unchecked	Checked	Unchecked	Unchecked
Unchecked	Unchecked	Unchecked	Unchecked	Checked	Unchecked	Unchecked
Unchecked	Unchecked	Unchecked	Unchecked	Unchecked	Unchecked	Unchecked
Unchecked	Unchecked	Unchecked	Unchecked	Unchecked	Unchecked	Unchecked
Unchecked	Unchecked	Unchecked	Unchecked	Unchecked	Unchecked	Unchecked
Unchecked	Unchecked	Unchecked	Unchecked	Unchecked	Unchecked	Unchecked
Unchecked	Unchecked	Unchecked	Unchecked	Unchecked	Unchecked	Unchecked
Unchecked	Unchecked	Checked	Unchecked	Unchecked	Unchecked	Unchecked
Unchecked	Unchecked	Unchecked	Unchecked	Unchecked	Unchecked	Unchecked
Unchecked	Unchecked	Unchecked	Unchecked	Unchecked	Unchecked	Unchecked
Unchecked	Unchecked	Unchecked	Unchecked	Checked	Unchecked	Unchecked
Unchecked	Checked	Unchecked	Unchecked	Unchecked	Unchecked	Unchecked
Unchecked	Unchecked	Unchecked	Unchecked	Unchecked	Unchecked	Unchecked
Unchecked	Unchecked	Unchecked	Unchecked	Unchecked	Unchecked	Unchecked
Unchecked	Unchecked	Unchecked	Unchecked	Unchecked	Unchecked	Unchecked
Unchecked	Unchecked	Checked	Unchecked	Unchecked	Unchecked	Unchecked
Unchecked	Unchecked	Unchecked	Unchecked	Unchecked	Unchecked	Unchecked
Unchecked	Unchecked	Unchecked	Unchecked	Unchecked	Unchecked	Unchecked
Unchecked	Unchecked	Unchecked	Unchecked	Unchecked	Unchecked	Unchecked
Unchecked	Unchecked	Unchecked	Unchecked	Unchecked	Unchecked	Unchecked
Unchecked	Unchecked	Unchecked	Unchecked	Unchecked	Unchecked	Unchecked
Unchecked	Unchecked	Unchecked	Unchecked	Unchecked	Unchecked	Unchecked
Unchecked	Unchecked	Unchecked	Unchecked	Unchecked	Unchecked	Unchecked
Unchecked	Unchecked	Checked	Unchecked	Unchecked	Unchecked	Unchecked
Unchecked	Unchecked	Unchecked	Unchecked	Unchecked	Unchecked	Unchecked
Unchecked	Unchecked	Unchecked	Checked	Unchecked	Unchecked	Unchecked
Unchecked	Checked	Unchecked	Unchecked	Unchecked	Unchecked	Unchecked
Unchecked	Unchecked	Unchecked	Unchecked	Unchecked	Unchecked	Unchecked
Unchecked	Unchecked	Unchecked	Unchecked	Unchecked	Unchecked	Unchecked
Unchecked	Unchecked	Checked	Unchecked	Unchecked	Unchecked	Unchecked
Unchecked	Unchecked	Unchecked	Unchecked	Unchecked	Unchecked	Unchecked
Unchecked	Unchecked	Unchecked	Unchecked	Unchecked	Unchecked	Unchecked
Unchecked	Unchecked	Unchecked	Unchecked	Unchecked	Unchecked	Unchecked
Unchecked	Unchecked	Checked	Unchecked	Unchecked	Unchecked	Unchecked
Unchecked	Unchecked	Unchecked	Unchecked	Unchecked	Unchecked	Unchecked

Unchecked	Unchecked	Unchecked	Unchecked	Unchecked	Unchecked	Unchecked
Unchecked	Unchecked	Unchecked	Unchecked	Unchecked	Unchecked	Unchecked
Checked	Unchecked	Unchecked	Unchecked	Unchecked	Unchecked	Unchecked
Unchecked	Unchecked	Checked	Unchecked	Unchecked	Unchecked	Unchecked
Unchecked	Unchecked	Unchecked	Unchecked	Unchecked	Unchecked	Unchecked
Unchecked	Unchecked	Unchecked	Unchecked	Unchecked	Unchecked	Unchecked
Unchecked	Unchecked	Unchecked	Unchecked	Checked	Unchecked	Unchecked
Unchecked	Unchecked	Unchecked	Unchecked	Unchecked	Unchecked	Unchecked
Unchecked	Unchecked	Unchecked	Unchecked	Checked	Unchecked	Unchecked
Unchecked	Unchecked	Unchecked	Unchecked	Unchecked	Unchecked	Unchecked
Unchecked	Unchecked	Unchecked	Unchecked	Unchecked	Unchecked	Unchecked
Unchecked	Checked	Unchecked	Unchecked	Unchecked	Unchecked	Unchecked
Unchecked	Unchecked	Unchecked	Unchecked	Unchecked	Unchecked	Unchecked
Unchecked	Unchecked	Unchecked	Unchecked	Unchecked	Unchecked	Unchecked
Unchecked	Unchecked	Unchecked	Unchecked	Unchecked	Unchecked	Unchecked
Unchecked	Unchecked	Unchecked	Unchecked	Unchecked	Unchecked	Unchecked
Unchecked	Unchecked	Unchecked	Unchecked	Unchecked	Unchecked	Unchecked
Unchecked	Unchecked	Unchecked	Unchecked	Unchecked	Unchecked	Unchecked
Unchecked	Unchecked	Unchecked	Unchecked	Unchecked	Unchecked	Unchecked
Unchecked	Unchecked	Unchecked	Unchecked	Unchecked	Unchecked	Unchecked
Unchecked	Unchecked	Unchecked	Unchecked	Checked	Unchecked	Unchecked
Unchecked	Unchecked	Unchecked	Unchecked	Unchecked	Unchecked	Unchecked
Unchecked	Unchecked	Unchecked	Unchecked	Unchecked	Unchecked	Unchecked
Unchecked	Unchecked	Unchecked	Unchecked	Unchecked	Unchecked	Unchecked
Unchecked	Unchecked	Unchecked	Unchecked	Unchecked	Unchecked	Unchecked
Unchecked	Unchecked	Unchecked	Unchecked	Unchecked	Unchecked	Unchecked
Unchecked	Unchecked	Unchecked	Unchecked	Unchecked	Unchecked	Unchecked
Unchecked	Unchecked	Unchecked	Unchecked	Unchecked	Unchecked	Unchecked
Unchecked	Unchecked	Unchecked	Unchecked	Unchecked	Unchecked	Unchecked
Unchecked	Unchecked	Unchecked	Unchecked	Unchecked	Unchecked	Unchecked
Unchecked	Unchecked	Unchecked	Unchecked	Unchecked	Unchecked	Unchecked
Unchecked	Unchecked	Unchecked	Unchecked	Unchecked	Unchecked	Unchecked
Unchecked	Unchecked	Unchecked	Unchecked	Unchecked	Unchecked	Unchecked
Unchecked	Unchecked	Unchecked	Unchecked	Checked	Unchecked	Unchecked
Unchecked	Unchecked	Unchecked	Unchecked	Unchecked	Unchecked	Unchecked
Unchecked	Unchecked	Unchecked	Unchecked	Unchecked	Unchecked	Unchecked
Unchecked	Unchecked	Unchecked	Unchecked	Unchecked	Unchecked	Unchecked
Unchecked	Unchecked	Unchecked	Unchecked	Unchecked	Unchecked	Unchecked
Unchecked	Unchecked	Unchecked	Unchecked	Unchecked	Unchecked	Unchecked
Unchecked	Unchecked	Unchecked	Unchecked	Unchecked	Unchecked	Unchecked
Unchecked	Unchecked	Checked	Unchecked	Unchecked	Unchecked	Unchecked
Unchecked	Unchecked	Unchecked	Unchecked	Checked	Unchecked	Unchecked
Unchecked	Unchecked	Unchecked	Unchecked	Unchecked	Unchecked	Unchecked

Unchecked	Unchecked	Unchecked	Unchecked	Unchecked	Unchecked	Unchecked
Unchecked	Unchecked	Checked	Unchecked	Unchecked	Unchecked	Unchecked
Unchecked	Unchecked	Unchecked	Unchecked	Checked	Unchecked	Unchecked
Unchecked	Unchecked	Unchecked	Unchecked	Unchecked	Unchecked	Unchecked
Unchecked	Unchecked	Unchecked	Unchecked	Unchecked	Unchecked	Unchecked
Unchecked	Unchecked	Unchecked	Unchecked	Unchecked	Unchecked	Unchecked
Unchecked	Unchecked	Unchecked	Unchecked	Unchecked	Unchecked	Unchecked
Unchecked	Unchecked	Unchecked	Unchecked	Unchecked	Unchecked	Unchecked
Unchecked	Unchecked	Unchecked	Unchecked	Unchecked	Unchecked	Unchecked
Unchecked	Unchecked	Unchecked	Unchecked	Unchecked	Unchecked	Unchecked
Unchecked	Unchecked	Unchecked	Unchecked	Unchecked	Unchecked	Unchecked
Unchecked	Unchecked	Unchecked	Unchecked	Unchecked	Unchecked	Unchecked
Unchecked	Unchecked	Unchecked	Unchecked	Unchecked	Unchecked	Unchecked
Unchecked	Unchecked	Unchecked	Unchecked	Unchecked	Unchecked	Unchecked
Unchecked	Unchecked	Unchecked	Unchecked	Unchecked	Unchecked	Unchecked
Unchecked	Unchecked	Unchecked	Unchecked	Unchecked	Unchecked	Unchecked
Unchecked	Unchecked	Unchecked	Unchecked	Unchecked	Unchecked	Unchecked
Unchecked	Unchecked	Unchecked	Unchecked	Unchecked	Unchecked	Unchecked
Unchecked	Unchecked	Unchecked	Unchecked	Unchecked	Unchecked	Unchecked
Unchecked	Unchecked	Unchecked	Unchecked	Unchecked	Unchecked	Unchecked
Unchecked	Unchecked	Unchecked	Unchecked	Unchecked	Unchecked	Unchecked
Unchecked	Unchecked	Unchecked	Unchecked	Unchecked	Unchecked	Unchecked
Unchecked	Unchecked	Unchecked	Unchecked	Unchecked	Unchecked	Unchecked
Unchecked	Unchecked	Unchecked	Unchecked	Unchecked	Unchecked	Unchecked
Unchecked	Unchecked	Unchecked	Unchecked	Unchecked	Unchecked	Unchecked
Unchecked	Unchecked	Checked	Unchecked	Unchecked	Unchecked	Unchecked
Unchecked	Unchecked	Unchecked	Unchecked	Unchecked	Unchecked	Unchecked
Unchecked	Unchecked	Unchecked	Unchecked	Unchecked	Unchecked	Unchecked
Unchecked	Unchecked	Unchecked	Unchecked	Unchecked	Unchecked	Unchecked
Unchecked	Unchecked	Unchecked	Unchecked	Unchecked	Unchecked	Unchecked
Unchecked	Unchecked	Unchecked	Unchecked	Unchecked	Unchecked	Unchecked
Unchecked	Unchecked	Unchecked	Unchecked	Unchecked	Unchecked	Unchecked
Unchecked	Unchecked	Unchecked	Unchecked	Unchecked	Unchecked	Unchecked
Unchecked	Unchecked	Unchecked	Unchecked	Unchecked	Unchecked	Unchecked
Unchecked	Unchecked	Unchecked	Unchecked	Unchecked	Unchecked	Unchecked
Unchecked	Unchecked	Unchecked	Unchecked	Unchecked	Unchecked	Unchecked
Unchecked	Unchecked	Unchecked	Unchecked	Unchecked	Unchecked	Unchecked
Unchecked	Unchecked	Unchecked	Unchecked	Unchecked	Unchecked	Unchecked
Unchecked	Unchecked	Unchecked	Unchecked	Unchecked	Unchecked	Unchecked
Unchecked	Unchecked	Unchecked	Unchecked	Unchecked	Unchecked	Unchecked
Unchecked	Unchecked	Unchecked	Unchecked	Checked	Unchecked	Unchecked
Unchecked	Unchecked	Unchecked	Unchecked	Unchecked	Unchecked	Unchecked
Unchecked	Checked	Unchecked	Unchecked	Unchecked	Unchecked	Unchecked

Unchecked	Unchecked	Unchecked	Unchecked	Unchecked	Unchecked	Unchecked
Unchecked	Unchecked	Unchecked	Unchecked	Unchecked	Unchecked	Unchecked
Unchecked	Unchecked	Unchecked	Unchecked	Unchecked	Unchecked	Unchecked
Unchecked	Unchecked	Unchecked	Checked	Checked	Unchecked	Unchecked
Unchecked	Unchecked	Unchecked	Unchecked	Checked	Unchecked	Unchecked
Unchecked	Unchecked	Unchecked	Unchecked	Unchecked	Unchecked	Unchecked
Unchecked	Unchecked	Unchecked	Unchecked	Unchecked	Unchecked	Unchecked
Unchecked	Unchecked	Unchecked	Unchecked	Unchecked	Unchecked	Unchecked
Unchecked	Unchecked	Checked	Unchecked	Unchecked	Unchecked	Unchecked
Unchecked	Unchecked	Unchecked	Unchecked	Unchecked	Unchecked	Unchecked
Unchecked	Unchecked	Unchecked	Unchecked	Unchecked	Unchecked	Unchecked
Unchecked	Unchecked	Unchecked	Unchecked	Unchecked	Unchecked	Unchecked
Unchecked	Unchecked	Unchecked	Unchecked	Unchecked	Unchecked	Unchecked
Unchecked	Unchecked	Unchecked	Unchecked	Unchecked	Unchecked	Unchecked
Unchecked	Unchecked	Unchecked	Unchecked	Unchecked	Unchecked	Unchecked
Unchecked	Unchecked	Unchecked	Unchecked	Unchecked	Unchecked	Unchecked
Unchecked	Unchecked	Unchecked	Unchecked	Unchecked	Unchecked	Unchecked
Unchecked	Unchecked	Unchecked	Unchecked	Unchecked	Unchecked	Unchecked
Unchecked	Unchecked	Checked	Unchecked	Unchecked	Unchecked	Unchecked
Unchecked	Unchecked	Unchecked	Unchecked	Unchecked	Unchecked	Unchecked
Unchecked	Unchecked	Unchecked	Unchecked	Unchecked	Unchecked	Unchecked
Unchecked	Unchecked	Unchecked	Unchecked	Unchecked	Unchecked	Unchecked
Unchecked	Unchecked	Unchecked	Unchecked	Unchecked	Unchecked	Unchecked
Unchecked	Unchecked	Unchecked	Unchecked	Unchecked	Unchecked	Unchecked
Unchecked	Unchecked	Unchecked	Unchecked	Unchecked	Unchecked	Unchecked
Unchecked	Checked	Checked	Unchecked	Unchecked	Unchecked	Unchecked
Unchecked	Checked	Unchecked	Unchecked	Unchecked	Unchecked	Unchecked
Unchecked	Unchecked	Unchecked	Unchecked	Unchecked	Unchecked	Unchecked
Unchecked	Unchecked	Unchecked	Unchecked	Unchecked	Unchecked	Unchecked
Unchecked	Unchecked	Unchecked	Checked	Unchecked	Unchecked	Unchecked
Unchecked	Unchecked	Unchecked	Unchecked	Unchecked	Unchecked	Unchecked
Unchecked	Unchecked	Unchecked	Unchecked	Unchecked	Unchecked	Unchecked
Unchecked	Unchecked	Unchecked	Unchecked	Unchecked	Unchecked	Unchecked
Unchecked	Unchecked	Unchecked	Unchecked	Unchecked	Unchecked	Unchecked
Unchecked	Checked	Unchecked	Unchecked	Unchecked	Unchecked	Unchecked
Unchecked	Checked	Unchecked	Unchecked	Unchecked	Unchecked	Unchecked
Unchecked	Unchecked	Unchecked	Unchecked	Unchecked	Unchecked	Unchecked
Unchecked	Unchecked	Unchecked	Unchecked	Unchecked	Unchecked	Unchecked
Unchecked	Checked	Unchecked	Unchecked	Checked	Unchecked	Unchecked
Unchecked	Unchecked	Unchecked	Unchecked	Unchecked	Unchecked	Unchecked
Unchecked	Unchecked	Unchecked	Unchecked	Unchecked	Unchecked	Unchecked
Unchecked	Unchecked	Unchecked	Unchecked	Unchecked	Unchecked	Unchecked
Unchecked	Unchecked	Unchecked	Unchecked	Unchecked	Unchecked	Unchecked

Unchecked	Unchecked	Unchecked	Unchecked	Unchecked	Unchecked	Unchecked
Unchecked	Unchecked	Unchecked	Unchecked	Unchecked	Unchecked	Unchecked
Unchecked	Unchecked	Unchecked	Unchecked	Unchecked	Unchecked	Unchecked
Unchecked	Unchecked	Unchecked	Unchecked	Unchecked	Unchecked	Unchecked
Unchecked	Unchecked	Unchecked	Unchecked	Unchecked	Unchecked	Unchecked
Unchecked	Unchecked	Unchecked	Unchecked	Unchecked	Unchecked	Unchecked
Unchecked	Unchecked	Unchecked	Unchecked	Unchecked	Unchecked	Unchecked
Unchecked	Unchecked	Unchecked	Unchecked	Unchecked	Unchecked	Unchecked
Unchecked	Unchecked	Unchecked	Unchecked	Unchecked	Unchecked	Unchecked
Unchecked	Unchecked	Unchecked	Unchecked	Unchecked	Unchecked	Unchecked
Unchecked	Unchecked	Unchecked	Unchecked	Unchecked	Unchecked	Unchecked
Unchecked	Unchecked	Unchecked	Unchecked	Unchecked	Unchecked	Unchecked
Unchecked	Unchecked	Unchecked	Unchecked	Unchecked	Unchecked	Unchecked
Unchecked	Unchecked	Unchecked	Unchecked	Unchecked	Unchecked	Unchecked
Unchecked	Unchecked	Unchecked	Unchecked	Unchecked	Unchecked	Unchecked
Unchecked	Unchecked	Unchecked	Unchecked	Unchecked	Unchecked	Unchecked
Unchecked	Unchecked	Unchecked	Unchecked	Unchecked	Unchecked	Unchecked
Unchecked	Unchecked	Unchecked	Unchecked	Unchecked	Unchecked	Unchecked
Unchecked	Unchecked	Unchecked	Unchecked	Unchecked	Unchecked	Unchecked
Unchecked	Unchecked	Unchecked	Unchecked	Unchecked	Unchecked	Unchecked
Unchecked	Unchecked	Unchecked	Unchecked	Unchecked	Unchecked	Unchecked
Unchecked	Unchecked	Unchecked	Unchecked	Unchecked	Unchecked	Unchecked
Unchecked	Unchecked	Unchecked	Unchecked	Unchecked	Unchecked	Unchecked
Unchecked	Unchecked	Unchecked	Unchecked	Unchecked	Unchecked	Unchecked
Unchecked	Unchecked	Unchecked	Unchecked	Unchecked	Unchecked	Unchecked
Unchecked	Unchecked	Unchecked	Unchecked	Unchecked	Unchecked	Unchecked

Se sim, você :	Outros: Othe	Em caso afirr	Você já foi dia	Se sim, quando? If yes, when?
Unchecked		Sim	Não	
Checked		Sim	Não	
Unchecked		Sim	Não	
Unchecked			Não sei	
Unchecked		Sim	Não	
Unchecked		Sim	Não	
Unchecked		Sim	Não	
Unchecked			Não	
Unchecked			Não	
Unchecked			Não	
Unchecked		Sim	Não	
Unchecked		Não	Não	
Checked		Sim	Não	
Unchecked			Não	
Unchecked		Sim	Não	
Unchecked			Não	
Unchecked		Sim	Não	
Unchecked			Não	
Unchecked			Sim	SOBRINHO
Unchecked		Sim	Não	
Unchecked		Sim	Não sei	
Checked		Sim	Não sei	
Checked		Sim		
Unchecked			Não	
Unchecked		Sim		
Unchecked		Sim	Não	
Unchecked		Sim		
Unchecked		Sim		
Unchecked		Sim		
Unchecked				
Unchecked		Sim		
Unchecked		Eu não sei	Não	
Unchecked			Não	
Unchecked		Sim	Não	
Unchecked			Não	
Unchecked		Sim	Não	
Unchecked		Sim	Não	
Unchecked		Sim	Não	
Unchecked			Não	
Unchecked			Não	
Unchecked		Sim	Não	
Unchecked			Não sei	

Unchecked	Sim	Não	
Checked	Eu não sei	Não	
Unchecked	Não	Não	
Unchecked	Sim	Não	
Checked	Sim	Não	
Checked	Sim	Não	
Unchecked	Sim	Não	
Unchecked			
Unchecked	Sim	Não	
Unchecked		Não	
Unchecked		Não	
Unchecked		Não	
Unchecked	Sim	Não	
Unchecked	Sim	Não	
Checked	Sim	Não	
Unchecked		Não	
Unchecked		Não	
Unchecked		Sim	NÃO LEMBRA
Unchecked		Não	
Unchecked	Sim	Não	
Unchecked	Sim	Não	
Unchecked	Sim	Não	
Unchecked		Não	
Unchecked		Não	
Unchecked	Sim	Não	
Unchecked		Não	
Unchecked	Sim	Não	
Unchecked		Não	
Unchecked		Não	
Unchecked		Não	
Unchecked	Sim	Não	
Unchecked	Sim	Não	
Unchecked		Não	
Unchecked	Sim	Sim	HÁ 5 ANOS
Unchecked		Não	
Unchecked	Sim	Não	
Unchecked		Não	
Unchecked	Sim	Não	
Unchecked	Sim	Não	
Unchecked		Não	
Unchecked		Sim	HÁ MUITOS ANOS (NÃO LEMBRA AO CER
Unchecked		Não	
Unchecked	Sim	Não	

Unchecked		Não	
Unchecked		Não	
Unchecked	Sim	Não	
Unchecked	Sim	Não	
Unchecked	Sim	Não	
Unchecked		Não	
Unchecked		Não	
Unchecked	Sim	Não	
Checked	Sim	Não	
Unchecked	Sim	Não	
Unchecked		Não	
Unchecked	Sim	Não	
Unchecked		Não sei	
Checked	Sim	Não sei	
Unchecked	Sim	Não sei	
Unchecked		Não sei	
Unchecked		Não sei	
Unchecked	Sim	Não sei	
Unchecked	Sim	Não sei	
Unchecked	Sim	Não sei	
Unchecked	Sim	Não sei	
Unchecked			
Unchecked	Sim	Não sei	
Unchecked	Não	Não sei	
Unchecked	Sim	Não	
Unchecked	Sim	Não sei	
Checked	Sim	Não sei	
Unchecked	Sim	Não	
Unchecked		Não sei	
Unchecked	Sim		
Unchecked	Sim	Não sei	
Unchecked	Não	Não	
Unchecked		Não sei	
Unchecked	Sim	Não sei	
Unchecked	Sim	Não sei	
Unchecked	Sim	Não sei	
Unchecked	Sim	Sim	há 10 anos
Unchecked	Sim	Não	
Unchecked		Não	
Unchecked	Sim	Não	
Unchecked		Não	
Unchecked	Sim	Não	
Checked	Sim	Sim	Com 10 anos

Unchecked	Sim	Não	
Unchecked	Sim	Não	
Unchecked	Sim	Não	
Unchecked	Sim	Não	
Unchecked	Sim	Não	
Unchecked	Sim	Não	
Unchecked	Sim	Não	
Unchecked	Sim	Sim	2009
Unchecked	Sim	Não	
Unchecked	Sim	Não	
Unchecked	Sim	Não	
Unchecked	Sim	Não	
Unchecked		Não	
Unchecked	Sim	Não	
Unchecked	Sim	Não	
Unchecked		Não	
Unchecked	Sim	Não	
Unchecked	Sim	Não	
Unchecked	Sim	Não	
Unchecked		Não	
Unchecked	Sim	Não	
Unchecked	Sim	Não	
Unchecked	Sim	Não	
Unchecked	Sim	Não	
Unchecked	Sim	Não	
Unchecked	Sim	Não	
Unchecked	Sim	Não	
Unchecked	Sim	Não	
Unchecked	Sim	Não	
Unchecked	Sim	Não	
Unchecked	Sim	Não	
Unchecked	Não	Não	
Unchecked		Não	
Unchecked	Sim	Não	
Checked	Sim	Não	
Unchecked		Não	
Unchecked	Sim	Não	
Unchecked		Não	
Unchecked	Sim	Não	
Unchecked	Sim	Não	
Unchecked	Sim	Não sei	
Unchecked	Sim	Não	
Unchecked	Sim	Não	

Unchecked		Sim	Não	
Unchecked		Sim	Não	
Unchecked		Sim	Não	
Unchecked		Sim	Não	
Unchecked		Sim	Não	
Unchecked		Sim	Não	
Unchecked		Sim	Não	
Checked		Eu não sei	Não	
Unchecked		Sim	Não	
Unchecked		Sim	Não	
Unchecked		Sim	Não	
Unchecked		Sim	Não	
Unchecked			Não	
Unchecked		Sim	Recusou a responder	
Checked		Eu não sei	Não	
Unchecked		Sim	Não	
Unchecked			Sim	NÃO INFORMA - ESTÁ COM MANCHAS N
Unchecked			Não	
Unchecked			Não	
Unchecked		Sim	Sim	NÃO RESPONDEU
Unchecked			Não	
Unchecked			Não	
Unchecked			Não	
Unchecked		Sim	Não	
Unchecked		Sim	Sim	QUANDO BEBÊ
Unchecked			Sim	HÁ MUITOS ANOS (NÃO ESPECIFICOU)
Unchecked		Sim	Não	
Unchecked			Não	
Unchecked		Sim	Recusou a responder	
Unchecked		Sim	Não	
Unchecked		Sim	Recusou a responder	
Unchecked			Não	
Unchecked		Sim	Não	
Unchecked	LEISHMANIO	Sim	Sim	HÁ 3 ANOS
Unchecked		Não	Sim	NÃO RESPONDEU
Unchecked			Recusou a responder	
Unchecked		Sim	Não	
Unchecked			Não	
Unchecked			Não	
Unchecked			Não	
Unchecked		Sim	Não	
Unchecked			Não	
Unchecked		Sim		

Unchecked		
Unchecked	Sim	Não
Checked	Sim	Não
Unchecked	Sim	Não
Unchecked	Sim	Não
Unchecked	Sim	Não
Unchecked	Sim	Não
Unchecked		Não
Unchecked		Não
Unchecked		Não
Unchecked	Sim	Não sei
Unchecked	Sim	Não
Unchecked	Sim	Não
Checked	Sim	Não
Unchecked		Não
Unchecked		Não
Unchecked	Sim	Recusou a responder
Unchecked		Não
Unchecked	Sim	Não
Unchecked	Sim	Não sei
Unchecked	Sim	Não
Unchecked		Não sei
Unchecked	Sim	Não sei
Checked	Eu não sei	Não sei
Unchecked	Sim	Não sei
Unchecked	Sim	Não sei
Unchecked		Não
Unchecked	Sim	Não
Unchecked		
Unchecked		Não sei
Unchecked	Sim	Não
Unchecked		Não
Unchecked	Sim	Não sei
Unchecked		Não sei
Checked	Não	Não sei
Unchecked	Sim	Não sei
Unchecked		Não sei
Unchecked	Sim	Não sei
Unchecked	Sim	Não sei
Unchecked	Sim	Não sei
Unchecked	Sim	Não sei
Unchecked	Sim	Não sei
Unchecked	Sim	Não sei

Unchecked		Sim	Não sei	
Unchecked		Sim	Não sei	
Unchecked		Sim	Não sei	
Unchecked			Não sei	
Unchecked			Não sei	
Unchecked			Sim	NÃO RELATA
Unchecked		Sim	Não sei	
Unchecked			Não	
Unchecked		Sim	Não sei	
Checked		Sim	Não sei	
Unchecked			Não sei	
Unchecked			Não sei	
Unchecked		Sim	Não sei	
Unchecked		Sim	Não sei	
Unchecked		Sim	Não sei	
Unchecked		Sim	Não	
Unchecked		Sim	Não	
Unchecked			Não	
Unchecked		Sim	Não sei	
Unchecked		Sim	Sim	11 ANOS
Unchecked		Sim	Não sei	
Unchecked		Não	Não	
Unchecked	LEICHIMANI(	Não	Não	
Checked		Sim	Não	
Unchecked		Sim	Não sei	
Unchecked		Sim	Não	
Unchecked		Sim	Não	
Unchecked				
Unchecked		Sim	Não	
Unchecked			Não	
Unchecked		Sim	Não	
Unchecked		Sim	Não sei	
Unchecked			Não	
Unchecked		Sim	Não	
Unchecked			Não	
Unchecked		Sim	Não	
Unchecked		Sim	Não	
Unchecked			Não sei	
Checked		Sim	Não	
Unchecked			Não	
Unchecked		Sim	Não	
Unchecked		Sim	Não	
Unchecked		Sim	Não	

Unchecked	INFECÇÃO D	Sim	Não
Unchecked			Não
Unchecked		Sim	Não
Unchecked		Sim	Não
Unchecked			Não
Unchecked		Sim	Não
Unchecked			Não sei
Unchecked		Sim	Não sei
Unchecked			Não sei
Unchecked		Sim	Não
Unchecked		Sim	Não
Unchecked			Não
Unchecked		Sim	Não
Unchecked		Sim	Não
Checked		Sim	Não
Unchecked		Sim	Não
Unchecked		Sim	Não
Unchecked			Não
Unchecked		Sim	Não
Unchecked			Não
Unchecked		Sim	Não
Unchecked			Não
Unchecked		Sim	Não
Unchecked		Sim	Não
Unchecked		Sim	Recusou a responder
Unchecked			Recusou a responder
Unchecked		Sim	Não
Unchecked			Não
Unchecked		Sim	
Unchecked		Sim	Não
Checked		Sim	Recusou a responder
Unchecked		Sim	Não sei
Unchecked		Sim	Não
Unchecked			
Unchecked		Sim	Não
Checked		Sim	Não sei
Unchecked			Recusou a responder
Unchecked			Não sei
Unchecked			Não sei
Unchecked		Sim	Não sei
Unchecked			Não sei
Unchecked		Sim	Não sei
Unchecked			Não sei

Unchecked		Não	
Unchecked		Não	
Unchecked	Sim	Não	
Unchecked	Sim	Não sei	
Unchecked		Não	
Unchecked	Sim	Não sei	
Unchecked	Sim	Não	
Unchecked	Sim	Recusou a responder	
Unchecked	Sim	Não	
Unchecked	Sim	Não	
Unchecked		Não sei	
Unchecked	Sim	Recusou a responder	
Unchecked		Não	
Unchecked		Não	
Unchecked	Sim	Sim	NÃO RESPONDEU
Unchecked	Sim	Não	
Checked	Sim	Não sei	
Unchecked		Não	
Unchecked	Sim	Sim	NÃO RESPONDEU
Unchecked	Sim	Não	
Unchecked	Sim	Não	
Unchecked		Não	
Unchecked		Não	
Unchecked	Sim	Não	
Unchecked	Sim	Não	
Unchecked	Sim	Não	
Unchecked	Sim	Não	
Unchecked	Sim	Não	
Unchecked	Sim	Não	
Unchecked		Não	
Unchecked	Sim	Não	
Unchecked		Não	
Unchecked		Não sei	
Unchecked	Sim	Não sei	
Unchecked	Sim	Não sei	
Unchecked		Não sei	
Unchecked	Eu não sei	Não sei	
Unchecked	Sim	Não sei	
Unchecked		Não sei	
Unchecked		Não sei	
Unchecked		Não sei	
Unchecked	Sim	Não sei	
Unchecked		Não sei	

Unchecked	Sim	Não sei	
Checked	Sim	Não sei	
Checked	Sim	Não sei	
Unchecked	Sim	Não sei	
Checked	Recusou-se	Recusou a responder	
Unchecked		Não	
Unchecked		Não	
Unchecked		Recusou a responder	
Unchecked	Sim	Não	
Unchecked		Não	
Unchecked	Sim	Não	
Unchecked	Sim	Recusou a responder	
Unchecked	Sim	Não	
Unchecked	Não	Não	
Unchecked		Não sei	
Unchecked		Recusou a responder	
Unchecked	Sim	Recusou a responder	
Unchecked	Sim	Não	
Checked	Não	Não	
Unchecked	Sim	Não	
Unchecked			
Unchecked		Sim	HÁ 20 ANOS ATRÁS
Unchecked	Sim		
Unchecked	Sim	Não sei	
Unchecked		Não sei	
Unchecked		Não	
Unchecked		Não	
Checked	Sim		
Unchecked		Não sei	
Unchecked	Sim	Não	
Unchecked	Sim	Sim	Há 25 anos ou mais
Unchecked		Não	
Unchecked	Sim	Não sei	
Unchecked		Não sei	
Unchecked	Sim	Não	
Unchecked	Sim	Não	
Unchecked	Sim	Recusou a responder	
Unchecked	Sim	Não	
Unchecked	Sim	Não	
Unchecked		Recusou a responder	
Unchecked		Recusou a responder	
Unchecked	Sim	Não	
Unchecked	Sim	Não sei	

Unchecked	Sim	Não sei
Unchecked	Sim	Sim
Unchecked	Sim	Não sei
Unchecked	Sim	Não sei
Unchecked		Sim
Unchecked	Sim	Não
Unchecked	Sim	
Checked		Não sei
Unchecked	Sim	Não sei
Unchecked		
Unchecked		Não
Unchecked	Sim	
Unchecked	Sim	Não sei
Unchecked		Não sei
Unchecked		Não sei
Unchecked	Sim	Não sei
Unchecked		Não
Unchecked		Não sei
Unchecked		Não
Unchecked	Sim	Não sei
Unchecked	Sim	Não
Unchecked		Não sei
Unchecked	Sim	Não
Unchecked		Não sei
Unchecked	Sim	Não sei
Unchecked	Sim	Não
Unchecked	Sim	Não
Unchecked	Sim	Não sei
Unchecked	Sim	Não sei
Unchecked		Não sei
Unchecked		
Unchecked		Não
Unchecked		Não sei
Unchecked		Não sei
Unchecked		Não
Unchecked	Sim	Não sei
Unchecked	Sim	Não
Unchecked	Sim	Não
Unchecked	Sim	Não
Unchecked	Sim	Não
Checked		Não sei
Unchecked	Sim	Não sei
Unchecked	Sim	Não sei

Unchecked	Sim	Não sei	
Unchecked	Sim	Não sei	
Unchecked		Não sei	
Unchecked	Sim	Não sei	
Unchecked		Não sei	
Unchecked		Não	
Unchecked		Não sei	
Unchecked	Sim	Sim	
Unchecked		Não sei	
Unchecked		Não sei	
Unchecked		Não sei	
Unchecked	Sim	Não sei	
Unchecked	Sim	Não sei	
Unchecked		Não sei	
Unchecked		Não sei	
Checked	Sim	Não sei	
Unchecked		Não sei	
Unchecked	Sim	Não sei	
Unchecked	Sim	Não sei	
Unchecked		Não sei	
Unchecked		Não sei	
Unchecked	Sim	Não sei	
Unchecked			
Unchecked	Sim	Não sei	
Unchecked	Sim	Não sei	
Unchecked		Sim	Há mais de 20 anos
Unchecked	Sim	Não	
Unchecked	Sim	Não sei	
Unchecked	Sim		
Unchecked	Sim	Não	
Unchecked		Não	
Unchecked		Não	
Unchecked	Sim	Não	
Unchecked	Sim	Não	
Checked	Sim	Não	
Unchecked	Sim	Não	
Unchecked		Não	
Unchecked	Sim	Não sei	
Checked	Sim	Não sei	
Unchecked		Não sei	
Unchecked		Não	
Unchecked	Sim	Não sei	
Unchecked	Sim	Não sei	

Unchecked	Não	Não	
Unchecked		Não sei	
Unchecked	Não	Não sei	
Unchecked		Não sei	
Unchecked	Sim	Não sei	
Unchecked	Sim	Não sei	
Unchecked	Sim	Não	
Unchecked	Sim	Não sei	
Unchecked	Sim	Não sei	
Unchecked	Sim	Não sei	
Unchecked		Não sei	
Unchecked	Sim	Não sei	
Unchecked		Não sei	
Unchecked		Não sei	
Checked	Não	Não sei	
Unchecked	Sim	Não sei	
Unchecked	Não	Não	
Unchecked		Não sei	
Unchecked	Sim	Não sei	
Unchecked		Não sei	
Unchecked			
Unchecked		Não sei	
Unchecked		Não sei	
Unchecked		Não sei	
Checked	Sim	Não sei	
Unchecked	Sim	Não sei	
Unchecked	Sim	Não	
Unchecked	Sim	Sim	2010
Unchecked		Não sei	
Unchecked	Sim	Não sei	
Unchecked		Não sei	
Unchecked		Não sei	
Unchecked	Sim	Não sei	
Unchecked		Não sei	
Unchecked	Sim	Não sei	
Unchecked		Não sei	
Unchecked		Não sei	
Unchecked	Sim	Não	
Unchecked		Não	
Unchecked	Sim	Não	
Unchecked	Sim	Não	
Unchecked	Sim	Não	
Unchecked	Sim	Não	

Unchecked		Sim	Não	
Unchecked			Não sei	
Unchecked		Sim	Não sei	
Unchecked				
Unchecked		Sim	Não sei	
Unchecked			Não	
Unchecked			Não	
Unchecked		Sim	Não	
Unchecked		Sim	Não	
Unchecked		Sim	Não	
Unchecked			Não	
Unchecked		Sim	Não	
Unchecked		Sim	Não	
Unchecked		Sim	Não	
Unchecked		Não	Não	
Checked		Sim	Não	
Unchecked			Não	
Unchecked		Sim	Não	
Unchecked			Não	
Unchecked		Sim	Não	
Unchecked			Não	
Unchecked		Sim	Não	
Unchecked		Sim	Não	
Unchecked			Não	
Unchecked		Sim	Não	
Unchecked		Sim	Não	
Unchecked		Sim	Não	
Unchecked		Sim	Não	
Unchecked		Sim	Não	
Unchecked	huminolepes	Sim	Não	
Checked		Sim	Não	
Unchecked		Sim	Não	
Unchecked		Sim	Não	
Unchecked			Não	
Unchecked		Sim	Não	
Unchecked		Sim	Não	
Checked		Eu não sei	Não	
Unchecked		Sim	Não	
Unchecked		Sim	Não	
Unchecked		Sim	Não	
Unchecked		Sim	Sim	tratou no creden-pes
Unchecked			Não	
Unchecked		Eu não sei	Sim	Não lembra, tratou somente com duas ca

Unchecked		Não	
Unchecked		Não	
Unchecked		Não	
Unchecked	Sim	Sim	Há mais de 10 anos
Unchecked		Não	
Unchecked	Sim	Não	
Unchecked		Não	
Unchecked	Sim	Não	
Unchecked		Não	
Unchecked		Não	
Unchecked		Não	
Unchecked	Sim	Não	
Unchecked		Não	
Unchecked	Sim	Não	
Unchecked		Não	
Unchecked		Não	
Unchecked		Não sei	
Unchecked		Não	
Unchecked		Não	
Unchecked	Sim	Não	
Unchecked		Não	
Unchecked	Sim	Não	
Unchecked	Sim	Não	
Unchecked	Não	Não	
Unchecked	Sim		
Unchecked		Não	
Unchecked		Não	
Unchecked			
Unchecked			
Unchecked		Não	
Unchecked	Sim	Não	
Unchecked	Sim	Não	
Unchecked	Sim	Não	
Unchecked		Não	
Unchecked		Não	
Unchecked	Sim	Não	
Unchecked		Não	
Unchecked	Sim	Não	
Unchecked		Não	
Unchecked	Sim	Não	
Unchecked	Sim	Não	
Unchecked	Sim	Não	
Unchecked	Eu não sei	Não	

Unchecked		Não	
Unchecked		Não	
Unchecked	Sim	Não	
Checked	Sim	Sim	Esta fazendo tratamento
Unchecked		Não	
Unchecked		Não	
Unchecked		Não	
Unchecked	Sim	Não	
Unchecked		Não	
Unchecked	Sim	Não	
Unchecked			
Unchecked			
Unchecked	Sim	Sim	Há 8 anos
Unchecked	Sim	Não	
Unchecked	Sim	Não	
Unchecked	Sim	Não	
Unchecked		Sim	Há mais de 10 anos
Unchecked	Sim	Não	
Unchecked	Sim	Não	
Unchecked	Sim	Não	
Unchecked	Sim	Não	
Unchecked	Sim	Não	
Unchecked	Sim	Não	
Unchecked		Não	
Unchecked	Sim	Não	
Unchecked	Sim	Não sei	
Unchecked		Não	
Unchecked		Não	
Unchecked	Eu não sei	Não	
Unchecked	Sim	Não	
Unchecked	Sim	Não	
Unchecked	Sim	Não	
Unchecked		Não sei	
Unchecked		Não	
Unchecked	Sim	Não	
Unchecked		Não	
Unchecked	Sim	Não	
Unchecked	Sim	Não	
Unchecked		Não	
Unchecked	Sim	Não	
Unchecked	Sim	Não	
Unchecked	Sim	Não	
Unchecked		Não	

Unchecked	Ancilostomíd	Sim	Sim	Há 23 anos
Unchecked		Sim	Não	
Unchecked		Sim	Sim	Há 20 anos
Unchecked		Sim	Não	
Unchecked			Não	
Unchecked		Sim	Recusou a responder	
Unchecked			Não	
Unchecked		Sim	Recusou a responder	
Unchecked			Sim	Há mais de 30 anos.
Unchecked		Sim	Não sei	
Unchecked		Sim	Não	
Unchecked			Não	
Unchecked		Sim	Não	
Unchecked		Sim	Não	
Unchecked		Não	Recusou a responder	
Checked		Não	Não	
Unchecked			Não	
Unchecked		Sim	Não	
Unchecked			Não	
Unchecked		Sim	Sim	Há 15 anos atrás
Unchecked		Sim	Não	
Unchecked		Sim	Não	
Unchecked		Sim	Não	
Unchecked		Sim	Não	
Unchecked		Sim	Não	
Unchecked		Sim	Não	
Unchecked		Sim	Não	
Unchecked			Não	
Unchecked			Não	
Unchecked			Não	
Unchecked		Sim	Não	
Unchecked		Não	Não	
Unchecked		Sim	Não	
Unchecked			Não	
Unchecked		Sim	Não	
Unchecked		Sim	Não	
Unchecked			Não	
Unchecked		Sim	Não	
Checked		Sim	Não	
Unchecked		Não	Sim	ATUALMENTE É PORTADOR DA DOENÇA
Unchecked		Sim	Não	
Unchecked			Não	
Unchecked		Sim	Não	

Unchecked	Sim	Não	
Unchecked	Sim	Não	
Unchecked	Sim	Não	
Checked	Sim	Não	
Unchecked	Sim	Não	
Unchecked	Sim	Não	
Unchecked		Recusou a responder	
Unchecked	Sim	Não	
Unchecked	Sim	Não	
Unchecked		Recusou a responder	
Unchecked	Sim	Não	
Unchecked	Sim	Não	
Unchecked	Sim	Não	
Unchecked		Não	
Unchecked		Não	
Unchecked		Não	
Unchecked	Eu não sei	Não	
Unchecked	Sim	Não	
Unchecked	Sim	Não	
Unchecked	Sim	Não	
Unchecked		Não	
Unchecked	Sim	Não	
Unchecked		Não	
Checked	Sim	Não	
Unchecked		Não	
Unchecked	Sim	Não	
Unchecked	Sim	Sim	INICIOU O TRATAMENTO
Unchecked	Sim	Não	
Unchecked	Sim	Não	
Unchecked	Sim	Não	
Unchecked		Não	
Unchecked	Sim	Não	
Unchecked	Sim	Não	
Unchecked	Sim	Sim	10 ANOS
Unchecked	Sim	Não	
Unchecked	Sim	Não	
Unchecked	Sim	Não	
Unchecked	Eu não sei	Não	
Unchecked	Eu não sei	Não	
Unchecked		Não	
Unchecked	Sim	Não	
Unchecked	Não	Não	
Unchecked	Sim	Não	

Unchecked	Sim	Não	
Unchecked		Sim	11 ANOS
Unchecked		Não	
Unchecked	Sim	Não	
Unchecked		Não	
Unchecked	Sim	Não	
Unchecked	Sim	Não	
Unchecked	Sim	Não	
Unchecked	Sim	Não	
Unchecked		Não	
Unchecked	Sim	Não	
Checked	Sim	Não	
Unchecked		Sim	HÁ 7 ANOS
Checked	Sim	Não	
Unchecked	Não	Não	
Checked	Sim	Não	
Unchecked	Sim	Não	
Unchecked	Sim	Não	
Unchecked		Não	
Unchecked	Sim	Não	
Unchecked	Sim	Não	
Unchecked	Sim	Não	
Checked	Sim	Não	
Unchecked	Sim	Não	
Unchecked	Sim	Não	
Unchecked	Sim	Não	
Unchecked	Sim	Não sei	
Unchecked		Não	
Checked	Sim	Não	
Unchecked	Sim	Não	
Unchecked			
Unchecked	Sim	Não	
Unchecked	Sim	Não	
Unchecked	Sim	Não	
Checked	Sim	Não	
Unchecked	Sim	Não	
Unchecked			
Unchecked	Sim	Não	
Unchecked			
Unchecked	Sim	Não	
Unchecked	Sim	Não	
Checked	Não	Não	
Checked	Sim	Não	

Unchecked		Não	
Unchecked	Sim	Não	
Unchecked	Sim	Não	
Unchecked		Não	
Checked	Sim	Não	
Unchecked	Sim	Não	
Unchecked		Não	
Unchecked		Não	
Unchecked		Não	
Unchecked	Sim	Sim	HÁ 10 ANOS
Unchecked	Sim	Não	
Unchecked	Sim	Não	
Unchecked	Sim	Não	
Unchecked		Não	
Unchecked	Sim	Não	
Unchecked	Sim	Não	
Unchecked	Sim	Não	
Unchecked	Sim	Não	
Unchecked	Sim	Sim	HÁ 3 ANOS
Unchecked	Sim	Não	
Unchecked	Sim	Não	
Unchecked	Sim	Não	
Unchecked	Sim	Não	
Unchecked	Sim	Não	
Unchecked	Eu não sei	Não	
Unchecked	Sim	Não	
Unchecked	Sim	Não	
Unchecked	Não	Não	
Unchecked	Sim	Não	
Unchecked	Sim	Não	
Unchecked		Não	
Unchecked	Sim	Não	
Unchecked	Sim	Não	
Unchecked	Sim	Não	
Unchecked	Sim	Não	
Unchecked	Sim	Sim	NÃO RESPONDEU
Unchecked	Eu não sei	Não	
Unchecked	Sim	Não	
Unchecked	Sim	Não	
Unchecked	Sim	Não	
Unchecked	Sim	Não	
Unchecked		Não	
Unchecked	Sim	Sim	HÁ 5 ANOS

Unchecked		Sim	Sim	NÃO RESPONDEU
Unchecked		Sim	Sim	EM TRATAMENTO - FORMA CLÍNICA VIRC
Unchecked		Sim	Não	
Unchecked		Sim	Não	
Unchecked		Sim	Não	
Unchecked		Sim	Não	
Checked		Sim	Não sei	
Unchecked		Sim	Não	
Unchecked			Não	
Checked		Sim	Não	
Unchecked			Não	
Unchecked		Sim	Não	
Unchecked		Sim	Não	
Unchecked		Sim	Não	
Unchecked		Sim	Não	
Unchecked		Sim	Não	
Checked		Sim	Não	
Unchecked			Não	
Unchecked			Não	
Unchecked		Sim	Não	
Unchecked	TOXOPLASM(	Não	Não	
Checked		Sim	Não	
Unchecked			Não	
Unchecked		Sim	Não	
Checked		Não		
Checked		Sim		
Unchecked		Sim	Não	
Checked		Sim	Não	
Unchecked			Não	
Unchecked		Sim	Não	
Unchecked		Sim	Não	
Unchecked		Sim	Não	
Checked		Sim	Não	
Unchecked			Não	
Unchecked			Não	
Unchecked			Não	
Unchecked			Não	
Unchecked		Sim	Não	
Unchecked			Não	
Unchecked			Não	
Checked		Sim	Não	
Unchecked		Sim	Não sei	
Unchecked			Não	

Unchecked	estrongiloidía	Sim	Não	
Unchecked	estrongiloidía	Sim	Não	
Unchecked				
Unchecked			Não	
Unchecked				
Unchecked			Não	
Unchecked			Não	
Checked		Sim	Não	
Unchecked			Não	
Unchecked		Sim	Não	
Checked		Sim	Não	
Unchecked			Não	
Unchecked		Sim	Não	
Unchecked		Não	Sim	não informado
Unchecked			Não	
Unchecked		Sim		
Unchecked		Sim	Não	
Unchecked		Sim	Não	
Unchecked		Sim	Não	
Unchecked		Sim	Não	
Unchecked		Sim	Não	
Unchecked		Sim	Não	
Unchecked			Não	
Unchecked		Sim	Não	
Unchecked		Sim	Não	
Unchecked		Sim	Sim	HÁ 5 ANOS APROXIMADAMENTE
Unchecked		Sim	Não	
Unchecked		Sim	Não	
Unchecked		Sim	Não	
Unchecked			Não	
Unchecked			Não	
Unchecked			Não sei	
Unchecked		Sim	Não	
Unchecked		Sim	Não	
Unchecked			Não	
Unchecked			Não	
Unchecked			Não	
Unchecked			Não	
Unchecked			Não	
Unchecked			Não	
Checked		Eu não sei	Sim	HÁ UNS 20 ANOS
Unchecked			Não	
Unchecked			Não	

Unchecked	Recusou-se	Sim	TEVE ALTA EM 2018
Unchecked	Sim	Não	
Unchecked	Sim	Não	
Unchecked		Não	
Unchecked		Não	
Unchecked	Sim	Não	
Unchecked	Sim	Não	
Unchecked	Sim	Não	
Unchecked	Sim	Não	
Unchecked	Sim	Não	
Unchecked		Não	
Unchecked		Não	
Unchecked		Não	
Unchecked	Não	Não	
Unchecked		Não	
Unchecked		Não	
Unchecked	Sim	Não	
Unchecked		Não	
Unchecked	Sim	Não	
Unchecked		Não	
Unchecked	Sim	Não	
Unchecked	Sim	Não	
Unchecked	Não	Não	
Unchecked		Não	
Unchecked	Sim	Sim	HÁ 16 ANOS
Unchecked	Sim	Não	
Unchecked	Sim	Não	
Unchecked	Sim	Não	
Unchecked		Não sei	
Unchecked	Eu não sei	Não	
Unchecked	Sim	Não	
Unchecked	Sim	Não	
Unchecked	Sim	Não	
Unchecked	Sim	Não	
Unchecked	Sim	Não	
Unchecked	Não	Não	
Unchecked	Sim	Não	
Unchecked	Sim	Não	
Unchecked	Sim	Não	
Unchecked	Sim	Sim	Não informado
Unchecked	Sim		
Unchecked	Sim	Não	
Unchecked	Sim	Não	

Unchecked	Sim	Não	
Unchecked		Sim	Não informado
Unchecked	Eu não sei	Não	
Unchecked	Sim	Sim	Há 7 anos
Unchecked	Sim	Sim	Há 7 anos
Unchecked	Sim	Não	
Unchecked	Sim	Sim	Há 5 anos
Unchecked	Sim	Não	
Unchecked	Sim	Não	
Unchecked	Sim	Não	
Unchecked	Sim	Não	
Unchecked		Sim	Há 5 anos
Unchecked	Sim	Não	
Unchecked	Sim	Não	
Unchecked		Não	
Unchecked		Não	
Unchecked	Sim	Não	
Unchecked		Não	
Unchecked		Não	
Unchecked		Não	
Unchecked		Não	
Unchecked		Não	
Unchecked	Sim	Não	
Unchecked	Sim	Não	
Unchecked		Não	
Unchecked		Não	
Unchecked	Sim		
Unchecked	Sim	Não	
Unchecked		Sim	Há 4 anos
Unchecked	Sim	Sim	NÃO CONSTA
Unchecked	Sim	Sim	HÁ 20 ANOS
Unchecked		Não	
Unchecked		Não	
Checked	Sim	Não	
Unchecked		Não sei	
Unchecked	Sim	Não	
Unchecked	Sim	Não	
Unchecked	Sim	Não	
Unchecked		Não	
Unchecked		Não	
Unchecked		Não	
Unchecked		Não	
Unchecked		Não	

Unchecked			Não	
Unchecked		Sim	Não	
Unchecked		Sim	Sim	30 anos atrás
Unchecked			Sim	30 anos atrás
Checked		Sim	Não	
Unchecked			Não	
Unchecked		Sim	Não	
Unchecked			Não	
Unchecked			Não	
Unchecked			Não	
Unchecked		Sim	Não	
Unchecked		Não	Não	
Unchecked		Sim	Não	
Unchecked			Não	
Unchecked			Não	
Unchecked			Não	
Unchecked		Sim	Não	
Unchecked			Não	
Unchecked		Sim	Recusou a responder	
Unchecked		Sim	Não	
Unchecked		Sim	Sim	tratamento 3 meses
Unchecked			Não	
Unchecked		Sim	Não	
Unchecked			Não	
Unchecked			Não	
Unchecked		Sim	Não	
Unchecked		Sim	Não	
Checked		Sim	Não	
Unchecked			Não	
Unchecked			Não	
Unchecked		Sim	Não	
Unchecked			Não	
Unchecked		Sim	Não	
Unchecked		Sim	Não	
Unchecked			Não	
Unchecked			Não	
Unchecked			Não	
Unchecked			Não	
Unchecked			Não	
Unchecked	Tirote	Sim	Não	
Unchecked		Sim	Não	
Unchecked		Sim	Não	
Unchecked			Não	

Unchecked		Eu não sei	Não	
Unchecked		Sim	Não	
Unchecked		Sim	Não	
Unchecked		Sim	Não	
Unchecked			Não	
Unchecked		Sim	Não	
Unchecked		Sim	Não	
Unchecked			Não	
Unchecked		Sim	Não	
Unchecked		Sim	Sim	não respondeu
Unchecked		Sim	Sim	quando era pequeño?
Unchecked			Não	
Unchecked		Sim	Não	
Unchecked			Não	
Unchecked			Não	
Unchecked			Não	
Unchecked		Sim	Não	
Unchecked			Não	
Unchecked		Sim	Não	
Unchecked			Não	
Unchecked		Sim	Não	
Unchecked			Não	
Unchecked			Não	
Unchecked		Sim	Não	
Unchecked			Não	
Unchecked			Não	
Checked		Sim	Não	
Unchecked	não sabe	Sim	Não	
Unchecked			Não	
Unchecked		Sim	Não	
Unchecked		Sim	Não	
Unchecked			Não	
Unchecked			Não	
Unchecked			Não	
Unchecked			Não	
Unchecked			Não	
Unchecked		Sim	Não	
Unchecked			Não	
Unchecked		Sim	Não	
Unchecked		Sim	Não	
Unchecked			Não	
Unchecked			Não	
Unchecked		Sim	Não	

Unchecked	Sim	Não	
Unchecked		Não	
Unchecked		Não	
Unchecked	Sim	Não	
Unchecked		Não	
Unchecked		Não	
Unchecked	Sim	Não	
Unchecked	Sim	Não	
Unchecked	Sim	Não	
Unchecked	Sim	Não	
Unchecked	Sim	Não	
Unchecked	Sim	Não	
Unchecked		Não	
Checked	Sim	Não	
Unchecked	Sim	Não	
Unchecked	Sim	Sim	há 10 anos
Unchecked	Sim	Não	
Checked	Sim	Não	
Unchecked	Sim	Não	
Unchecked	Não		
Checked	Não	Não	
Unchecked	Sim	Não	
Unchecked	Sim	Não	
Unchecked	Sim	Não	
Unchecked	Sim	Não	
Unchecked	Sim	Não	
Unchecked	Sim	Não	
Unchecked	Sim	Não	
Unchecked	Sim	Não	
Unchecked		Não	
Unchecked		Não	
Unchecked	Sim	Não	
Unchecked	Sim	Não	
Unchecked	Sim	Não	
Unchecked	Sim	Não	
Unchecked	Sim	Não	
Checked	Sim	Não	
Unchecked	Sim	Não	
Unchecked		Não	
Unchecked	Sim	Não	
Unchecked	Sim	Não	
Unchecked	Sim	Não sei	
Unchecked	Sim	Não	

Unchecked		
Unchecked		
Unchecked	Sim	Não
Unchecked	Sim	Não
Unchecked	Sim	Não
Unchecked	Sim	Não
Unchecked	Sim	Não
Unchecked	Sim	Não
Unchecked	Sim	Não
Unchecked		Não
Unchecked		Não
Unchecked	Sim	Não
Unchecked		Não sei
Unchecked	Eu não sei	Não
Unchecked	Sim	Não
Unchecked	Sim	Não
Unchecked	Sim	Não
Unchecked		Não
Unchecked		Não
Unchecked		Não
Unchecked	Sim	Não
Checked	Não	Não
Unchecked		Não
Unchecked	Sim	Não
Unchecked	Sim	Não
Unchecked		Não
Unchecked		Não
Unchecked	Eu não sei	Não
Unchecked		Não
Unchecked		Não
Unchecked	Sim	Não
Unchecked	Sim	Não
Unchecked	Sim	Não
Unchecked	Sim	Não
Checked	Não	Não
Unchecked	Sim	Não
Unchecked	Sim	Não
Unchecked		Não
Unchecked		Não sei
Unchecked		Não
Unchecked	Sim	Não
Unchecked	Sim	Não
Unchecked		Não

Checked	Sim	Não	
Unchecked	Sim	Não	
Unchecked	Sim	Não	
Unchecked		Não	
Unchecked	Sim	Sim	HÁ MAIS DE 10 ANOS
Unchecked	Sim	Não	
Checked	Sim	Não	
Unchecked		Não	
Checked	Sim	Não	
Unchecked	Sim	Sim	ESTÁ EM TRATAMENTO (PACIENTE DO CF
Unchecked	Sim	Não	
Unchecked		Não	
Unchecked		Sim	SIM, COM 18 ANOS
Unchecked		Não	
Unchecked	Sim	Não	
Unchecked		Não	
Unchecked	Não	Não	
Unchecked	Eu não sei	Não	
Unchecked		Não	
Unchecked		Não	
Unchecked	Sim	Não	
Unchecked	Sim	Não	
Unchecked	Sim	Não	
Unchecked		Não	
Unchecked		Não	
Unchecked	Sim	Não	
Unchecked		Não	
Unchecked		Não sei	
Unchecked	Sim	Não	
Unchecked	Sim	Não	
Unchecked	Sim	Não	
Unchecked		Não	
Unchecked	Sim	Não	
Unchecked	Sim	Não	
Unchecked		Não	
Unchecked		Não	
Unchecked	Sim	Não	
Unchecked	Sim	Não	
Unchecked		Não	
Unchecked	Não	Não	
Unchecked	Sim	Sim	HA 10 ANOS
Unchecked		Não	
Unchecked	Sim	Não	

Unchecked		Não sei	
Unchecked		Não	
Unchecked	Sim	Não	
Unchecked	Sim	Não	
Unchecked	Sim	Não	
Unchecked	Sim	Não	
Unchecked		Não	
Unchecked		Não	
Unchecked	Sim	Não	
Unchecked		Não	
Unchecked	Sim	Não	
Unchecked		Não	
Unchecked		Não sei	
Unchecked	Não	Não	
Unchecked	Sim	Não	
Checked	Sim	Não	
Checked	Eu não sei	Não	
Unchecked		Não	
Unchecked	Sim	Não	
Unchecked		Não	
Unchecked		Não	
Unchecked		Não	
Unchecked		Sim	10 ANOS
Unchecked		Não	
Unchecked	Sim	Não	
Unchecked	Sim	Não	
Unchecked	Sim	Não	
Unchecked		Não	
Unchecked		Não	
Unchecked	Sim	Não	
Checked	Não	Não	
Unchecked		Não	
Unchecked		Não	
Unchecked	Sim	Não	
Unchecked		Sim	HÁ 40 ANOS
Unchecked	Sim	Sim	HÁ 40 ANOS
Unchecked	Sim	Não	
Unchecked	Sim	Não	
Unchecked	Sim	Não	
Unchecked		Recusou a responder	
Unchecked	Sim	Não	
Unchecked	Sim	Não	
Unchecked		Recusou a responder	

Unchecked	Recusou a responder
Unchecked	Recusou a responder
Unchecked	Recusou a responder
Unchecked	Recusou a responder
Unchecked	Recusou a responder
Unchecked	Recusou a responder
Unchecked	Recusou a responder
Unchecked	Não sei
Unchecked	Não sei
Unchecked	Não sei
Unchecked	Não sei
Unchecked	Não sei
Unchecked	Não sei
Unchecked	Não sei
Unchecked	Não sei
Unchecked	Não sei
Unchecked	Não sei
Unchecked	Não sei
Unchecked	Não sei
Unchecked	Não sei
Unchecked	Recusou a responder
Unchecked	Não sei
Unchecked	Recusou a responder
Unchecked	Não sei
Unchecked	Não sei
Unchecked	Recusou a responder

TO)

O CORPO

artelas verde

REDEN-PES) - PESQUISA MARCOS
