# Supplementary material for: Mycobacterium leprae infection, Hansen’s disease, and helminth infections: A cross-sectional study in southeastern Brazil
Source: PLoS Negl Trop Dis. 2026 Oct 1;20(10):e0013285. doi: 10.1371/journal.pntd.0013285 (PMC13693981; doi:10.1371/journal.pntd.0013285)
Supplement: Supplementary file 2 [file pntd.0013285.s001.docx]

**SUPPLEMENTARY TABLES**

**S1 Table: Multivariate Logistic Regression with POSITIVE ANTI-LID1 and HISTORY OF HANSEN’S DISEASE against co-infection (serological results and *past parasitic infection*) and demographic risk factors**

=====================================================================================

**Pos Anti-LID-1	History of HD**

aOR (95% CI)	aOR (95% CI)

------------------------------------------------------------------------------------------------------------------------------------------

*S. mansoni* antibody positive (anti-SEA)	0.63 (0.28, 1.41)	1.05 (0.50, 2.21)

*S. sterocralis* antibody positive (anti-NIE)	1.64 (0.72, 3.74)	0.45 (0.11, 1.94)

Past parasitic infection	0.97 (0.60, 1.57)	1.50 (0.80, 2.83)

Race (non-white)	1.15 (0.65, 2.02)

Adult age (15 or older)	6.31 (0.85, 46.65)

Sex as female	0.64 (0.39, 1.07)

Rural residence	**1.86* (1.12, 3.07)	1.84* (1.06, 3.17**)

=====================================================================================

Note: Adjusted odds ratio listed with confidence intervals (in parentheses) of exposures involving positive

serological results of helminth infections, reporting ‘yes’ to past parasitic infection, and best fit

demographic variables. *p<0.05
