# Supplementary material for: Mycobacterium leprae infection, Hansen’s disease, and helminth infections: A cross-sectional study in southeastern Brazil
Source: PLoS Negl Trop Dis. 2026 Oct 1;20(10):e0013285. doi: 10.1371/journal.pntd.0013285 (PMC13693981; doi:10.1371/journal.pntd.0013285)
Supplement: Supplementary file 3 [file pntd.0013285.s002.docx]

**SUPPLEMENTARY TABLES**

**S2 Table: MLR with POSITIVE ANTI-LID1 and HISTORY OF HANSEN’S DISEASE against co-infection (serological results and *all individual parasites*) and demographic risk factors**

=====================================================================================

**Pos Anti-LID-1	History of HD**

aOR (95% CI)	aOR (95% CI)

------------------------------------------------------------------------------------------------------------------------------------------

*S. mansoni* antibody positive (anti-SEA)	0.63 (0.28, 1.40)	1.09 (0.52, 2.31)

*S. sterocralis* antibody positive (anti-NIE)	1.63 (0.71, 3.76)	0.41 (0.09, 1.82)

Self-reported roundworm	0.97 (0.61, 1.53)	1.50 (0.90, 2.51)

Self-reported schistosomiasis	0.89 (0.52, 1.51)	0.92 (0.52, 1.63)

Self-reported hookworm	3.46 (0.82, 14.58)	0.79 (0.09, 6.82)

Self-reported tapeworm	1.88 (0.86, 4.11)	1.36 (0.54, 3.44)

Self-reported pinworm	0.58 (0.24, 1.45)	1.15 (0.46, 2.87)

Self-reported giardia	1.00 (0.34, 2.95)	1.39 (0.47, 4.16)

Self-reported amoeba	0.63 (0.26, 1.52)	0.49 (0.17, 1.41)

Self-reported other	0 (0, Inf)	3.51 (0.39, 33.45)

Race (non-white)	1.15 (0.69, 2.03)

Adult age (15 or older)	6.85 (0.92, 50.85)

Sex as female	0.64 (0.378, 1.07)

Rural residence	**1.75* (1.03, 2.96)	1.84* (1.05, 3.25)**

=====================================================================================

Note: Adjusted odds ratio listed with confidence intervals (in parentheses) of exposures involving positive

serological results of helminth infections, self-reported past or present infections, and best fit

demographic variables. * p<0.05
