# Supplementary material for: Mycobacterium leprae infection, Hansen’s disease, and helminth infections: A cross-sectional study in southeastern Brazil
Source: PLoS Negl Trop Dis. 2026 Oct 1;20(10):e0013285. doi: 10.1371/journal.pntd.0013285 (PMC13693981; doi:10.1371/journal.pntd.0013285)
Supplement: Supplementary file 4 [file pntd.0013285.s003.docx]

**SUPPLEMENTARY TABLES**

**S3 Table: Checking effects size change among different model permutations for the**

**outcome of POSITIVE ANTI-LID1 (aOR)**

**Both A & SR	A Only	SR Only**

-----------------------------------------------------------------------------------------------------------------------------------------

*S. mansoni* antibody positive (anti-SEA)	0.78	0.78

*S. sterocralis* antibody positive (anti-NIE)	1.57	1.54	1.57

Self-reported schistosomiasis	1.00	0.99

Self reported helminth infection (other than *S. mansoni*)	1.14	1.15	1.14

Self-reported protozoa	0.96	0.95	0.96

Race (non-white)	1.21	1.21	1.21

Rural Residence	1.81**	1.79**	1.81**

===================================================================================

Note: ‘A’ stands for antibody result for schistosomiasis. ‘SR’ is self report for schistomiasis. **p<0.05.
