# Supplementary material for: Mycobacterium leprae infection, Hansen’s disease, and helminth infections: A cross-sectional study in southeastern Brazil
Source: PLoS Negl Trop Dis. 2026 Oct 1;20(10):e0013285. doi: 10.1371/journal.pntd.0013285 (PMC13693981; doi:10.1371/journal.pntd.0013285)
Supplement: Supplementary file 5 [file pntd.0013285.s004.docx]

**SUPPLEMENTARY TABLES**

**S4 Table: Checking effects size change among different model permutations for the**

**outcome of HISTORY OF HANSEN’S DISEASE (aOR)**

**Both A & SR	A Only	SR Only**

-----------------------------------------------------------------------------------------------------------------------------------------

*S. mansoni* antibody positive (anti-SEA)	1.25	1.25

*S. sterocralis* antibody positive (anti-NIE)	0.40	0.40	0.40

Self-reported schistosomiasis	0.87	0.87

Self reported helminth infection (other then S. mansoni)	1.77**	1.75**	1.74**

Self-reported protozoa	0.84	0.82	0.84

Adult age (15 or older)	7.75**	7.47**	7.97**

Sex as female	0.64*	0.64*	0.63*

Rural Residence	1.97**	1.91**	1.99**

===================================================================================

Note: ‘A’ stands for antibody result for schistosomiasis. ‘SR’ is self-report for schistomiasis. **p<0.05.
